# MinION Analysis and Reference Consortium: Phase 2 data release and analysis of R9.0 chemistry

**DOI:** 10.12688/f1000research.11354.1

**Published:** 2017-05-31

**Authors:** Miten Jain, John R. Tyson, Matthew Loose, Camilla L.C. Ip, David A. Eccles, Justin O'Grady, Sunir Malla, Richard M. Leggett, Ola Wallerman, Hans J. Jansen, Vadim Zalunin, Ewan Birney, Bonnie L. Brown, Terrance P. Snutch, Hugh E. Olsen, MinION Analysis and Reference Consortium

**Affiliations:** 1 University of California at Santa Cruz, Santa Cruz, CA, USA; 2 Michael Smith Laboratories and Djavad Mowfaghian Centre for Brain Health, University of British Columbia, Vancouver, Canada; 3 School of Life Sciences, University of Nottingham, Nottingham, UK; 4 Wellcome Trust Centre for Human Genetics, University of Oxford, Oxford, UK; 5 Peter Medawar Building for Pathogen Research, University of Oxford, Oxford, UK; 6 Malaghan Institute of Medical Research, Wellington, New Zealand; 7 Norwich Medical School, University of East Anglia, Norwich, UK; 8 Earlham Institute, Norwich Research Park, Norwich, UK; 9 Science for Life Laboratory, IGP, Uppsala University, Uppsala, Sweden; 10 ZF-screens B.V., Leiden, Netherlands; 11 European Molecular Biology Laboratory (EMBL), European Bioinformatics Institute, Wellcome Trust Genome Campus, Hinxton, UK; 12 Virginia Commonwealth University, Richmond, VA, USA

**Keywords:** MinION, nanopore sequencing, R9 chemistry, CsgG, data release, long reads, minoTour, marginAlign, NanoOK, third-generation sequencing

## Abstract

Background: Long-read sequencing is rapidly evolving and reshaping the suite of opportunities for genomic analysis. For the MinION in particular, as both the platform and chemistry develop, the user community requires reference data to set performance expectations and maximally exploit third-generation sequencing. We performed an analysis of MinION data derived from whole genome sequencing of *Escherichia* *coli* K-12 using the R9.0 chemistry, comparing the results with the older R7.3 chemistry.

Methods: We computed the error-rate estimates for insertions, deletions, and mismatches in MinION reads.

Results: Run-time characteristics of the flow cell and run scripts for R9.0 were similar to those observed for R7.3 chemistry, but with an 8-fold increase in bases per second (from 30 bps in R7.3 and SQK-MAP005 library preparation, to 250 bps in R9.0) processed by individual nanopores, and less drop-off in yield over time. The 2-dimensional (“2D”) N50 read length was unchanged from the prior chemistry. Using the proportion of alignable reads as a measure of base-call accuracy, 99.9% of “pass” template reads from 1-dimensional (“1D”)  experiments were mappable and ~97% from 2D experiments. The median identity of reads was ~89% for 1D and ~94% for 2D experiments. The total error rate (miscall + insertion + deletion ) decreased for 2D “pass” reads from 9.1% in R7.3 to 7.5% in R9.0 and for template “pass” reads from 26.7% in R7.3 to 14.5% in R9.0.

Conclusions: These Phase 2 MinION experiments serve as a baseline by providing estimates for read quality, throughput, and mappability. The datasets further enable the development of bioinformatic tools tailored to the new R9.0 chemistry and the design of novel biological applications for this technology.

Abbreviations: K: thousand, Kb: kilobase (one thousand base pairs), M: million, Mb: megabase (one million base pairs), Gb: gigabase (one billion base pairs).

## Introduction

The Oxford Nanopore Technologies (ONT) MinION Access Programme (MAP) released the MinION™ nanopore sequencer to early access users in June 2014. The MinION Analysis and Reference Consortium (MARC) was formed by a subset of MAP participants to perform independent evaluation of the platform, share standard protocols, collaboratively produce reference data for the nanopore community, and to address biological questions. The Phase 1 MARC analysis of October 2015 ^1^ was an evaluation of the library preparation chemistry version SQK–MAP005, R7.3 flow cell chemistry, and a base-calling algorithm derived from a Markov model (HMM) using a 5-mer model. The R9.0 chemistry and protocol, ( https://www.youtube.com/watch?v=nizGyutn6v4) was made available to users in June 2016 ( https://londoncallingconf.co.uk/lc/2016-plenary#168687629). This substantial upgrade to the platform included the CsgG membrane protein for the pore and a recurrent neural network (RNN) for base-calling. In part, ONT claimed these changes made substantial improvements to data yields and quality, to the extent that 1-dimensional (“1D”) reads, without a hairpin, could be used for analyses in many use-cases.

Before embarking on further analyses, MARC performed “bridging experiments” to evaluate the effect of the R9.0 changes on data yield, quality, and accuracy. To capture variability and reproducibility among experiments using R9.0 chemistry, two labs concurrently sequenced *Escherichia coli* strain K-12 substrain MG1655, the same strain used for MARC Phase 1 ^1^. Sequencing was performed using both the 2-dimensional (“2D”) “ligation” kit and the newer 1D “rapid” kit. The analyses performed included characterizing throughput, read quality, and accuracy. This work also marks the release of MinION Phase 2 data for both sequencing modes with the R9.0 chemistry. Although the newer R9.4 flow cell chemistry has become available to the community since the Phase 2 experiments were performed in late July and early August 2016, ONT have stated that R9.4 flow cell chemistry has similar base-calling characteristics compared to R9.0, as it uses the same pore and base-calling strategy. Thus, this data release and analysis is of interest as it describes the major changes introduced with the R9 chemistry. It is a resource to aid further developments in nanopore informatics as well as the development of biological applications using the MinION.

## Materials and methods

Two laboratories each performed a 1D and a 2D experiment using the protocol described in MARC Phase 1 ^1^ to obtain total genomic DNA from freshly grown cells ( Supplementary File 1) and slightly modified protocols for 1D “rapid” and 2D “ligation” library preparation and sequencing.

### Cell culture and DNA extraction of the *E. coli* K-12 target sample

*E. coli* cells were cultured and DNA was extracted using the protocol described in MARC Phase 1 ( Supplementary File 1).

### 2D sequencing library preparation

Sequencing libraries were prepared according to the ONT recommended 2D protocol (SQK-NSK007 kits), which included addition of the lambda control sample, with the following changes:

(i) genomic DNA was sheared to ~10 kb; and

(ii) both labs performed a 0.4x AMPureXP cleanup post-FFPE treatment.

### 1D sequencing library preparation

Sequencing libraries were prepared according to the ONT recommended 1D protocol (SQK-RAD001 kits, referred to as 1D “rapid” sequencing) with the following changes:

(i) a 0.4x AMPureXP cleanup was performed prior to 1D library preparation;

(ii) an unsheared input DNA sample of 400 ng was used for the library;

(iii) 0.4 μl Blunt/TA Ligase was added; and

(iv) a 10 min incubation was used in the final step.

Note that this protocol does not include addition of the lambda control sample DNA.

### Sequencer configuration and sequencing run conditions

All sequencing runs used MinKNOW (version 1.0.3) and Metrichor Desktop Agent. The experiments are henceforth referred to as P2-Lab6-R1-2D, P2-Lab7-R1-2D, P2-Lab6-R1-1D and P2-Lab7-R1-1D following a “phase-lab-replicate-kit” format. All flow cells used for sequencing underwent the standard MinION Platform QC for analysis of overall quality and number of functional pores. This was followed by the recommended priming step, after which the prepared library was loaded onto an R9.0 flow cell. Final library volume for the 1D runs was 11.2 μl, which was loaded once with running buffer at the start of the experiment. A 500 μl flush with running buffer alone was performed at 24 hrs on the P2-Lab6-R1-1D run. The final volume of 2D libraries was 25 μl, of which 12 μl was loaded with running buffer at the start of the sequencing run followed by addition of another 12 μl library aliquot 16 hours into the run. All sequencing runs were performed on MinION Mk1b devices using the standard MinKNOW 48-hour sequencing protocol (NC_48Hr_Sequencing_Run_FLO-MIN104).

### Base-calling and data formats

The sequencing data for 1D MinION runs were base-called using the Metrichor 1D Base-calling RNN for the SQK-RAD001 (v1.107) workflow. This workflow classified base-called sequence data into “pass” and “fail” categories based on the mean Phred-scaled quality score for that read. The threshold for a read to be categorized as “pass” was a Q-value of 6. The sequencing data for 2D MinION runs were base-called using the Metrichor 2D Base-calling RNN for the SQK-NSK007 (v1.107) workflow. Similarly, this workflow classified reads into “pass” and “fail” with a Q-value threshold of 9 required for pass reads.

### European Nucleotide Archive data pre-processing pipeline

As in Phase 1, the base-called FAST5 files and meta-data were collated on a server at the European Nucleotide Archive (ENA). These data were then processed using several tools. The base-calls in FASTQ format were extracted using poretools (version 0.5.1) ^2^ and then aligned against the *E. coli* K-12 reference genome ( NCBI RefSeq, accession NC_000913.1) using BWA-MEM (version 0.7.12-41044), parameter “-x ont2d” ^3^ and LAST (version 460) ^4^, parameters “-s 2 -T 0 -Q 0 -a 1” as recommended by 5. Both alignments were then improved with marginAlign (version 0.1) ^6^, and were statistics computed using marginStats ^6^.

### Data analyses

The R9.0 data were characterized by collating statistics for a typical run from MARC Phase 1 (P1b-Lab2-R2, hereafter referred to as P1b-Lab2-R2-2D for consistency with the Phase 2 experiment naming convention) and the four Phase 2 experiments. In keeping with the MARC Phase 1 analyses ^1^, we computed alignments and error-rate measurements using BWA-MEM and LAST, followed by re-alignment using marginAlign ^6^. Real-time evaluation of the runs was performed by minoTour ^7^ (more information available from: http://minotour.github.io/minoTour), run locally at the two experimental laboratories. The “pass” and “fail” reads from each experiment were evaluated with NanoOK (version 0.95) ^8^ using bwa alignments. Additional metrics and analyses were performed with bespoke Python and R scripts, (available at https://github.com/camilla-ip/marcp2) ^9^.

## Results

### Experimental conditions

The MARC Phase 2 experiments were performed by two laboratories ( Supplementary File 1) between 27 July and 2 August 2016 ( Table 1). The total number of functional g1 pores prior to sequencing on R9.0 flow cells was ~94%, an improvement from ~88% for R7.3 ( Table 1). The operating ASIC (chip) temperature on the R9.0 flow cell ranged from 30 to 34°C, and the temperature regulation of the flow cell heat sink was a uniform 34°C across all flow cells ( Table 1). All experiments ran for at least 40 hours of the 48 hour run script. However, experiment P2-Lab6-R1-2D crashed when the controlling computer’s hard-drive reached capacity; it was restarted ~42 hours after the initial experiment start time using modified recipe scripts, but produced few further reads. Experiment P2-Lab7-R1-2D was terminated after ~44 hours. Experiment P2-Lab7-R1-1D was restarted twice between 24 and 32 hours and terminated at 41.5 hours ( Table 1).

**Table 1.** Experimental conditions. P1 refers to a typical R7.3 run from MARC Phase 1 ^1^. P2 refers to the MARC Phase 2 R9.0 data presented in this study. NA: not available.

	P1b-Lab2-R2-2D	P2-Lab6-R1-2D	P2-Lab7-R1-2D	P2-Lab6-R1-1D	P2-Lab7-R1-1D
Library & base-call type	2D	2D	2D	1D	1D
Flow cell version	R7.3	R9	R9	R9	R9
MinION device	Initial version	Mk1b	Mk1b	Mk1b	Mk1b
Experiment start date	2015-07-25	2016-07-27	2016-07-27	2016-08-02	2016-07-29
Active g1 pores (% of 512)	87.9	94	NA	94	NA
Active g2 pores (% of 512)	60.7	77	NA	69	NA
Mean ASIC temperature (°C)	24.4	30.5	33.7	31.9	33.8
Mean heat-sink temp (°C)	37.1	34.0	34.0	34.0	34.0
Experiment run time (h)	48.0	41.5	44.0	48.0	48.0
Experimental notes	Full run	Hard drive filled up at ~41.5 h	Terminated early ~44h as no more data generated	Full run; no lambda control sample	Two restarts between 24 and 32 h, no lambda control sample

### Data format and experimental constants

One challenge of MinION data analysis is referencing the proper data format after major upgrades, such as the switch from an HMM to an RNN base-caller. The new or superseded fields in the resulting table after introduction of R9 chemistry are shown in Supplementary File 2 ^9^.

### Base yield and read lengths

**Figure 1.**
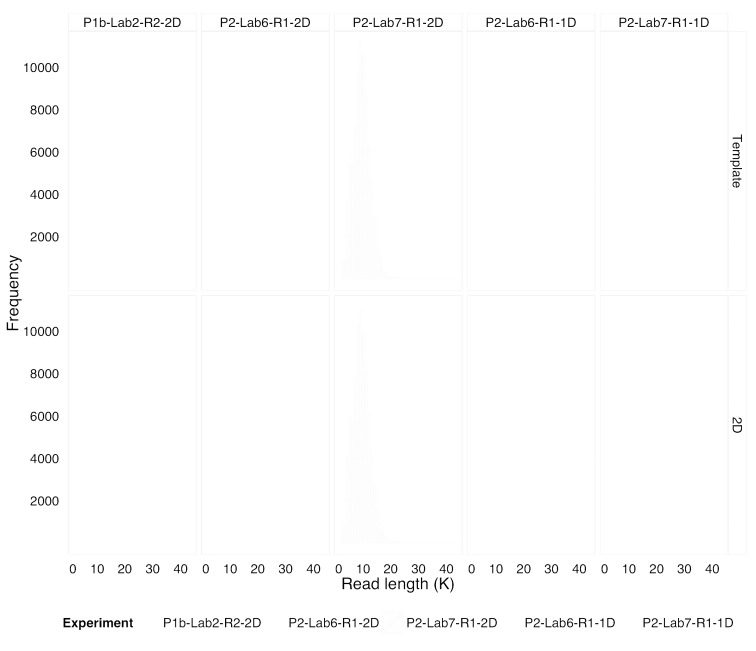
Read length distribution for template and 2D “pass” reads. The distribution of template (“1D”) read lengths for experiments based on 1D “rapid” libraries (P2-Lab6-R1-1D and P2-Lab7-R1-1D) was skewed toward shorter read lengths due to enzymatic, rather than mechanical, DNA fragmentation. The long tails of the distributions were truncated at 40,000 bases for clarity.

The read count, base yield, and read lengths of the 2D and 1D R9.0 experiments compared to a typical R7.3 experiment ( Table 2 and Table 3, and Figure 1) were inferred from NanoOK reports ( Supplementary File 3) and bespoke scripts ^9^. There was considerable variability between the quantity of data produced by the two 2D experiments and the two 1D experiments, but overall, the R9.0 chemistry showed an increase in data yield and read length when compared with a typical Phase 1 R7.3 experiment.

**Table 2.** Read counts and base yields. (“-”) indicates not applicable.

	P1b-Lab2-R1-2D	P2-Lab6-R1-2D	P2-Lab7-R1-2D	P2-Lab6-R1-1D	P2-Lab7-R1-1D
Read count (K) files - total pass fail template - total pass fail comp - total pass fail 2D - total pass fail	48.5 (100%) 14.8 (30.5%) 33.7 (69.5%) 48.0 (99.0%) 14.8 (30.8%) 33.2 (69.2%) 34.2 (70.5%) 14.8 (43.3%) 19.4 (56.7%) 21.4 (44.1%) 14.8 (69.2%) 6.6 (30.8%)	126.8 (100%) 41.7 (32.9%) 85.1 (67.1%) 126.7 (99.9%) 41.7 (32.9%) 85.0 (67.1%) 89.2 (70.3%) 41.7 (46.7%) 47.4 (53.1%) 63.6 (50.2%) 41.7 (65.6%) 21.9 (34.4%)	216.5 (100%) 80.6 (37.2%) 135.9 (62.8%) 216.5 (100%) 80.6 (37.2%) 135.8 (62.7%) 146.6 (67.7%) 80.6 (55.0%) 66.0 (45.0%) 111.4 (51.5%) 80.6 (72.4%) 30.8 (27.6%)	96.2 (100%) 56.9 (56.1%) 39.3 (40.9%) 96.2 (100%) 56.9 (59.1%) 39.3 (40.9%) - -	57.4 (100%) 35.3 (60.5%) 22.1 (38.5%) 57.4 (100%) 35.3 (60.5%) 22.1 (38.5%) - -
Base yield (Mb) template - total pass fail comp - total pass fail 2D - total pass fail	242.4 (100%) 92.1 (38.0%) 150.3 (62.0%) 180.3 (74.4%) 87.7 (48.7%) 92.5 (51.3%) 128.2 (52.9%) 94.1 (73.4%) 34.2 (26.6%)	790.5 (100%) 330.5 (41.8%) 460.0 (58.2%) 437.6 (55.6%) 286.2 (65.4%) 151.4 (34.6%) 414.9 (52.5%) 320.8 (77.3%) 94.1 (22.7%)	1268.8 (100%) 546.3 (43.1%) 722.5 (56.9%) 681.1 (53.7%) 481.3 (70.7%) 199.8 (29.3%) 665.6 (52.5%) 533.8 (80.2%) 131.8 (19.8%)	829.7 (100%) 526.3 (63.4%) 303.3 (36.6%) - -	410.6 (100%) 276.3 (67.3%) 134.3 (32.7%) - -

**Table 3.** Read lengths. (“-”) indicates not applicable.

	P1b-Lab2-R1-2D		P2-Lab6-R1-2D		P2-Lab7-R1-2D		P2-Lab6-R1-1D		P2-Lab7-R1-1D	
	total	pass	total	pass	total	pass	total	pass	total	pass
Mean length (Kb) template complement 2D	5.0 5.3 6.0	6.2 5.9 6.4	6.2 4.9 6.5	7.9 6.9 7.7	5.9 4.6 6.0	6.8 6.0 6.6	8.6 - -	9.2 - -	7.2 - -	7.8 - -
**Longest read (Kb) template complement 2D	244.5 50.2 35.2	36.1 33.9 35.2	119.0 403.7 50.9	50.3 47.0 50.9	110.8 253.0 40.1	34.6 34.9 35.8	478.8 - -	141.2 - -	353.1 - -	151.2 - -
Longest aligned read (> 75% length aligned) (Kb) template complement 2D	35.6 33.9 35.2	34.6 33.9 35.2	56.4 47.0 50.9	50.3 47.0 50.9	42.7 32.2 33.6	33.3 32.2 33.6	141.2 - -	141.2 - -	151.2 - -	151.2 - -
N50 length (Kb) template complement 2D	6.9 6.8 7.4	7.5 7.1 7.6	9.4 8.0 9.1	10.0 8.8 9.8	7.6 6.5 7.3	7.9 7.0 7.8	15.7 - -	16.2 - -	13.1 - -	13.6 - -

** : Longest read here is pre-alignment.

Improvements in base yield and read length were observed for the 2D R9.0 experiments compared with a typical R7.3 experiment ( Table 2 and Table 3). The 2D R9.0 experiments sequenced 127–217 K molecules (compared with ~49 K molecules for the typical Phase 1 R7.3 experiment). Of these, ~50% resulted in 2D reads (an improvement from ~44% for the typical R7.3 experiment) and a total of 64–111 K 2D pass reads (compared with 21 K for the typical R7.3 experiment). The proportion of “pass” reads with a Q-value threshold of 9 was 66% to 72%, about the same as that observed for the typical R7.3 experiment, with a base quality threshold of 9.0. Average read lengths of “pass” 2D base-calls were higher at 6.6–7.7 Kb (compared with 6.4 Kb for the typical R7.3 experiment), and for “all” 2D base-calls at 6.0–6.5 Kb (compared with 6.0 Kb for the typical R7.3 experiment). The longest 2D reads observed in R9.0 (50.9 Kb, Table 3) were comparable to those observed in R7.3 experiments (59.7 Kb) ^1^. However, the longest 2D aligned read observed increased to 50.9 Kb (from 35.2 Kb in the typical R7.3 experiment) ( Table 3). The increase in N50 read length to 7.3–9.1 Kb for all 2D reads in the R9.0 experiments (compared with 7.4 Kb for the typical R7.3 experiment) and 7.8–9.8 Kb for “pass” R9.0 reads (compared with 7.6 Kb for the R7.3 experiment) indicates, as for the 1D data, an overall increase in the proportion of longer 2D base-called reads.

The 1D R9.0 experiments sequenced 57–96 K molecules (compared with 49 K for the typical Phase 1 R7.3 experiment), resulting in a total template base yield of 410–830 Mb (compared with 242 Mb for the typical R7.3 experiment), of which ~60% were higher-quality “pass” reads with a Q-value threshold of 6.0 (compared with ~31% for the typical Phase 1 experiment classified with 2D base quality threshold of 9.0) ( Table 2). Read lengths also improved, with the mean template length for “pass” reads increasing to 7.2–8.6 Kb (from 5.0 for R7.3) and increasing to 7.8–9.4 for “fail” reads (from 6.2 for the R7.3 experiment). The longest mappable template read observed across all of the R9 runs was 151.2 kb and the artefactually long reads, detectable by a discrepancy between the longest read lengths and the longest mappable read lengths, were comparably rare ( Table 3). Read length N50 increased to 13.1–15.7 Kb for “pass” reads (compared with 6.9 Kb for the typical R7.3) and 13.6–16.2 Kb (compared with 7.5 Kb for the typical R7.3), indicating that more of the base-calls were contained in longer reads.

We observed that the speed and convenience of the 1D “rapid” library protocol came at a cost. The distribution of template “pass” read lengths was skewed toward shorter reads peaking closer to 1 Kb rather than the ~6.5 Kb obtained through the 2D “ligation” library protocol. However, one benefit was that a greater proportion of longer reads was also produced ( Figure 1). The addition of the lambda control sample in the 2D library protocol resulted in a variable ratio of “target” to “control” sample reads, evident in the relative sizes of the bimodal read length distributions for the 2D library experiments ( Figure 1).

### Alignment identity and accuracy

The proportion of alignable reads is a measure of the accuracy of the base-calls. For template reads from both 1D and 2D experiments, 99.9% of “pass” reads were alignable from both 1D and 2D experiments, and 60% and 83% for “fail” reads from 1D and 2D experiments, respectively ( Table 4).

**Table 4.** Per-read accuracy metrics for target E. coli sample. (“-”) indicates the metrics were not applicable for that experiment. NA: not available.

	P1b-Lab2-R1-2D		P2-Lab6-R1-2D		P2-Lab7-R1-2D		P2-Lab6-R1-1D		P2-Lab7-R1-1D	
	pass	fail	pass	fail	pass	fail	pass	fail	pass	fail
Identity % template complement 2D	77.9 79.7 92.4	74.6 76.8 82.2	89.4 88.5 93.5	85.0 83.1 69.9	89.7 88.9 94.0	85.6 83.4 69.1	88.0 - -	76.0 - -	88.8 NA NA	75.6 - -
Reads mapped % template complement 2D	96.3 96.1 96.7	47.5 59.8 84.0	95.9 95.6 95.9	72.7 61.5 83.7	98.0 97.8 98.0	77.2 63.0 82.4	99.9 - -	62.6 - -	99.9 - -	57.5 - -
Longest perfectly aligned subsequence template complement 2D	87 75 333	85 75 333	275 228 713	248 228 713	274 203 750	271 203 750	235 - -	235 - -	273 - -	273 - -
Total error % template complement 2D	26.7 27.9 9.1	32.8 32.4 19.7	14.5 17.4 7.8	19.2 23.0 25.4	14.0 16.7 7.2	18.5 22.6 25.4	15.3 - -	30.5 - -	14.5 - -	31.3 - -
Miscall % template complement 2D	10.3 9.9 1.9	12.2 10.6 5.2	6.1 6.5 2.1	8.0 8.6 7.2	5.9 6.4 2.0	8.0 8.7 7.2	6.4 - -	15.1 - -	5.9 - -	15.7 - -
Insertion % template complement 2D	6.5 6.3 3.1	7.6 7.6 6.5	2.7 3.0 2.0	3.8 4.5 7.9	2.6 2.9 1.9	3.6 4.4 8.1	3.2 - -	5.8 - -	3.0 - -	5.9 - -
Deletion % template complement 2D	9.9 11.8 4.1	13.0 14.2 8.0	5.8 7.9 3.7	7.4 9.9 10.3	5.5 7.4 3.3	6.9 9.5 10.2	5.7 - -	9.7 - -	5.6 - -	9.8 - -

The median identity of reads from 1D and 2D experiments ( Table 4) was similar to that observed for the R7.3 chemistry in MARC Phase 1. The median identity for 1D template reads was ~88% and ~76%, for “pass” and “fail”, respectively (compared with 78% and 75% for the typical R7.3 experiment). For the 2D experiments, the read identity was ~89% and ~85%, for “pass” and “fail”, respectively (compared with ~92% and ~82%, respectively, for the typical R7.3 experiment).

Another metric of overall error, the longest perfectly aligned subsequence, showed improvement associated with the R9.0 chemistry. The longest perfectly aligned subsequences in the R9.0 1D runs were 235 and 273 bases (compared with 87 in the typical R7.3 experiment), and in the 2D runs were 713 and 750 bases (compared with 333 bases in the typical R7.3 experiment).

### Miscall, insertion, and deletion rates

The total error of “pass” reads in the 1D sequencing experiments reduced from 26.7% in R7.3 to 15.0% in R9.0 (miscalls 6.2%, insertions 3.1%, deletions 5.7%) ( Table 4). Little change was observed for the “fail” template reads, between the 32.8% observed for a typical R7.3 experiment and the 31.1% for the R9.0 experiments (miscalls 15.4%, insertions 5.9%, deletions 9.8%) ( Table 4).

Total error of the 2D reads was reduced from 9.1% in R7.3 to 7.3% in R9.0 for “pass” reads, whereas the total error increased for “fail” reads from 19.7% in R7.3 to 25.4% in R9.0 ( Table 4).

### Sequencing performance over time

**Figure 2.**
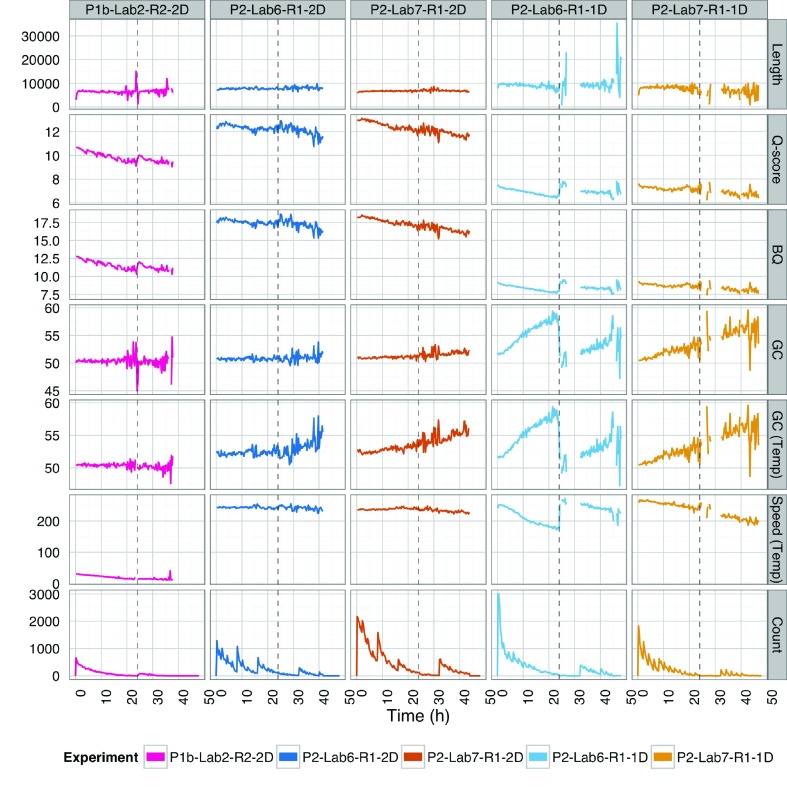
Sequencing performance over time. The mean read length (kb), Q-score, base quality (BQ), and GC%, speed (bases per second), and throughput (count) for each experiment, computed from “pass” reads that mapped to the *E. coli* reference, were plotted for 15 minute intervals. The values for template reads (“1D”) are plotted for the 1D libraries (P2-Lab6-R1-1D and P2-Lab7-R1-1D) whereas the values for 2D reads were plotted for the 2D libraries (P1b-Lab2-R2-2D, P2-Lab6-R1-2D, and P2-Lab7-R1-2D).

In the MARC Phase 1 analysis of R7.3 chemistry experiments, the quantity and quality of data produced during an experiment varied as material passed from one side of the membrane to the other. This was punctuated by periodic changes in voltage every 4 hours, and a switch to the group 2 pores at 24 hours ^1^. To enable a direct comparison between the performance of the R7.3 and R9.0 chemistry, key metrics were plotted for 15 minute windows over the course of the 48 hour experiment for the typical R7.3 experiment (P1b-Lab2-R2-2D) and the four R9.0 experiments on the same scale ( Figure 2). The mean of each time window was computed from “pass” reads that mapped to the *E. coli* reference genome, to remove irregularities due to poor quality reads. The metrics computed from template base-called reads were plotted for the 1D library experiments, and those from 2D base-called reads for the 2D library experiments.

The plots show some irregularities due to lower throughput before the pore group switch at 24 hours, towards the end of the runs, during run script restarts (in P2-Lab7-R1-1D and P2-Lab6-R1-2D), and at the early termination (P2-Lab7-R1-2D). However, in general, the read lengths and GC% varied around a constant value over time and the Q-score and base quality dropped at a similar rate ( Figure 2) This was despite sequencing speed increasing (measured in bases per second) from about 30 bps to 250 bps. Differences in the Lab6 and Lab7 1D “rapid” run plots around the 24hr point can be attributed to flushing of the flowcell with 500μl of fresh running buffer in the case of Lab6. This appears to be of benefit for speed and quality, but would require further investigation on a chemistry no longer in use. This procedure may be worth bearing in mind going forward, however, for possible beneficial effects with newer chemistries.

We noticed an increase in the GC content of the template reads from the 1D “rapid” library experiments and to a lesser extent for the 2D reads from the 2D experiments ( Figure 2). These plots should have shown stochastic variation throughout the run around the mean GC of 50.8% for the *E. coli* sample. We considered a number of possible factors that could account for this artefact including: (i) low data density; (ii) an over-representation of poorer-quality “fail” reads; (iii) an over-representation of unmappable reads; or (iv) high-GC repetitive motifs. We found a negative correlation for the R9.0 1D data between %GC and average QV scores and also a decrease in base qualities over time. This was particularly pronounced for 1D “fail” reads (Q 3–10), but persisted even for 2D reads, likely due to 1D consensus follow through. The current report is for the initial R9.0 chemistry, and the GC-bias seems to be less pronounced with the improved version of the R9 pore (R9.4 data not shown).

## Discussion

The MARC Phase 2 experiments were performed with the MinION Mk1b device to provide an independent evaluation of the performance, data yield, and data quality of the R9.0 chemistry and scripts. By comparing the data from four R9.0 experiments on the same *E. coli* isolate sequenced with R7.3 chemistry in MARC Phase 1 ^1^, we have established new benchmarks for data from the 1D “rapid” and 2D “ligation” protocols and kits available in late July 2016. ( Table 1).

We have verified that the MinION Mk1b device reliably maintains the R9.0 flow cell at an appropriate temperature ( Table 1). The R9.0 flow cells improve overall data yield through provision of a higher proportion of available functional pores during an experiment, with 94% functional group 1 pores observed in this study ( Table 1). With higher yields comes an increased chance of experiment failure as the file system accepting the data is likely to reach capacity during a run ( Table 1). This suggests scripts should be deployed routinely to move the data from the file system during the sequencing run. The FAST5 data format continues to evolve and improve ( Supplementary File 2) to store more comprehensive metadata in a more logical internal structure, and is now beginning to be documented on the MAP Community Forum (available via https://nanoporetech.com).

In the 12 months between the MARC Phase 1 and Phase 2 experiments ( Table 1), we observed that for 2D base-calls, the distribution of read lengths remained the same ( Figure 1, Table 3). The yield of higher-quality “pass” base-calls increased from ~100 Mb to ~450 Mb per flow cell ( Table 2), and the total error of the “pass” base-calls reduced from 9.1% to 7.5% ( Table 4). The read length and GC% over the course of the experiment remained uniform ( Figure 2). The initial mean Q-scores increased from ~11 to over 12. The initial mean base qualities increased from ~12.5 to over 17.5, and both decreased gradually over the course of an experiment as observed previously ( Figure 2). Finally, the proportion of mappable reads remained comparable, between 96 and 98% ( Table 4) despite the sequencing speed increasing from 50 to 250 bases per second ( Supplementary File 2). The yield improvements are a result of higher speeds and proportion of available pores, and the increase in data quality is attributed to the newer RNN basecaller.

The new 1D “rapid” library protocol, which sequences a single DNA strand, has the potential to query twice as many molecules during the lifetime of a flow cell. We found that this technique is a viable alternative to 2D library chemistry for use-cases where rapid scanning of the population of library molecules is important. The higher total error of 15.3% for “pass” template base-calls, compared with 7.5% for “pass” 2D base-calls ( Table 4), is an acceptable trade off.

We confirm that the yield and quality of MinION data continues to improve. The data released in this study provide a benchmark to compare the newer R9.4 chemistry to and can be used to develop bioinformatic tools tailored to the newer chemistry. The updated reports of achievable data yield and quality, along with the characteristics of data production during the lifetime of a flow cell, will enable the design of new biological applications for this third-generation sequencing technology. Although a newer R9.4 chemistry has recently become available, ONT has emphasized that R9 platforms that use the CsgG nanopore will be backward compatible. This study provides the first comprehensive description of data from R9.0 flow cells and RNN base-calling software. We anticipate that it will serve as a framework for evaluating changes resulting from subsequent R9-based chemistries.

## Data and software availability

All data presented in this study are available via ENA with accession PRJEB18053.

Archived source code as at the time of publication: http://dx.doi.org/10.5281/zenodo.582311 ^10^

License: CC BY 4.0

## Supplementary materials

Supplementary File 1. Laboratories. List of laboratories that generated data for this study.

Supplementary File 2. Experimental constants. Table of metadata fields and values shared across experiments.

Supplementary File 3. NanoOK experiment reports. NanoOK PDF reports for three sets of reads (pass only, fail only, and both pass and fail) for each experiment.
