# Supplementary material for: MinION Analysis and Reference Consortium: Phase 2 data release and analysis of R9.0 chemistry
Source: F1000Res. 2017 May 31;6:760. [Version 1] doi: 10.12688/f1000research.11354.1 (PMC5538040; doi:10.12688/f1000research.11354.1)
Supplement: Supplementary file 3 [file f1000research-6-12257-s0002.tgz › 5d2560fe-dd7a-44d1-b265-e3522e742149.zip!/SI File 3. NanoOK experiment reports/P1b-Lab2-R2_2D_nanook_bwa_passonly.pdf]

NanoOK report for UCSC_MARC_Phase_Ib_Run_2

Pass and fail counts

Type	Pass	Fail
Template	14800	0
Complement	14800	0
2D	14800	0

Read lengths

Type	NumReads	TotalBases	Mean	Longest	Shortest	N50	N50Count	N90	N90Count
Template	14800	92094831	6222.62	36112	209	7480	4707	4109	10968
Complement	14800	87742236	5928.53	33891	186	7112	4703	3900	10962
2D	14800	94094963	6357.77	35194	199	7648	4715	4200	10961

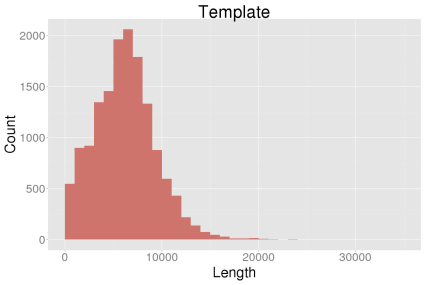

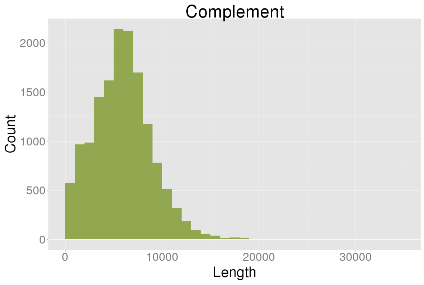

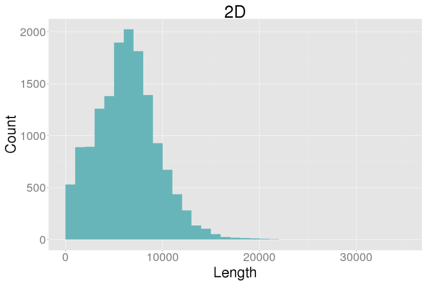

Template alignments

Number of reads	14800	
Number of reads with alignments	14723	(99.48%)
Number of reads without alignments	77	(0.52%)

ID	Size	Number of Reads	% of Reads	Mean read length	Aligned bases	Mean coverage	Longest Perf Kmer
Control sequence	3560	465	3.14	3081.82	1528170	429.26	64
Escherichia coli	4641652	14258	96.34	6354.28	98084004	21.13	85

Complement alignments

Number of reads	14800	
Number of reads with alignments	14681	(99.20%)
Number of reads without alignments	119	(0.80%)

ID	Size	Number of Reads	% of Reads	Mean read length	Aligned bases	Mean coverage	Longest Perf Kmer
Control sequence	3560	466	3.15	2886.96	1434536	402.96	52
Escherichia coli	4641652	14215	96.05	6071.83	95561670	20.59	75

2D alignments

Number of reads	14800	
Number of reads with alignments	14778	(99.85%)
Number of reads without alignments	22	(0.15%)

ID	Size	Number of Reads	% of Reads	Mean read length	Aligned bases	Mean coverage	Longest Perf Kmer
Control sequence	3560	470	3.18	3084.69	1500163	421.39	233
Escherichia coli	4641652	14308	96.68	6473.64	96083224	20.70	333

Control sequence error analysis

	Template	Complement	2D
Overall base identity (excluding indels)	75.74%	74.18%	90.65%
Aligned base identity (excluding indels)	82.74%	83.26%	95.21%
Identical bases per 100 aligned bases (including indels)	71.03%	69.57%	87.61%
Inserted bases per 100 aligned bases (including indels)	5.27%	3.81%	2.97%
Deleted bases per 100 aligned bases (including indels)	8.89%	12.64%	5.01%
Substitutions per 100 aligned bases (including indels)	14.81%	13.98%	4.41%
Mean insertion size	1.59	1.49	1.49
Mean deletion size	1.59	1.77	1.60

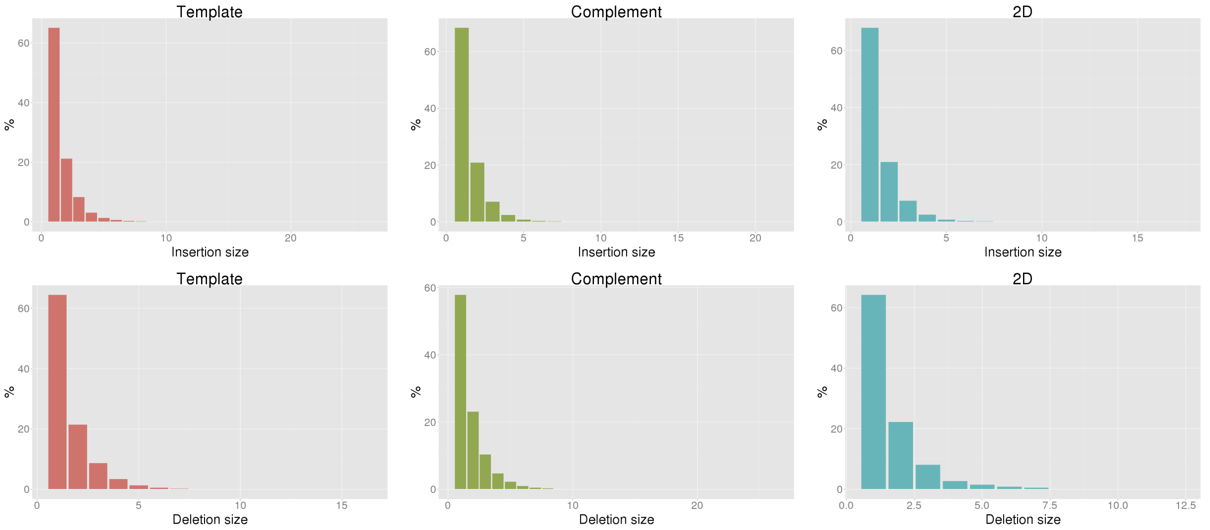

Control sequence read identity

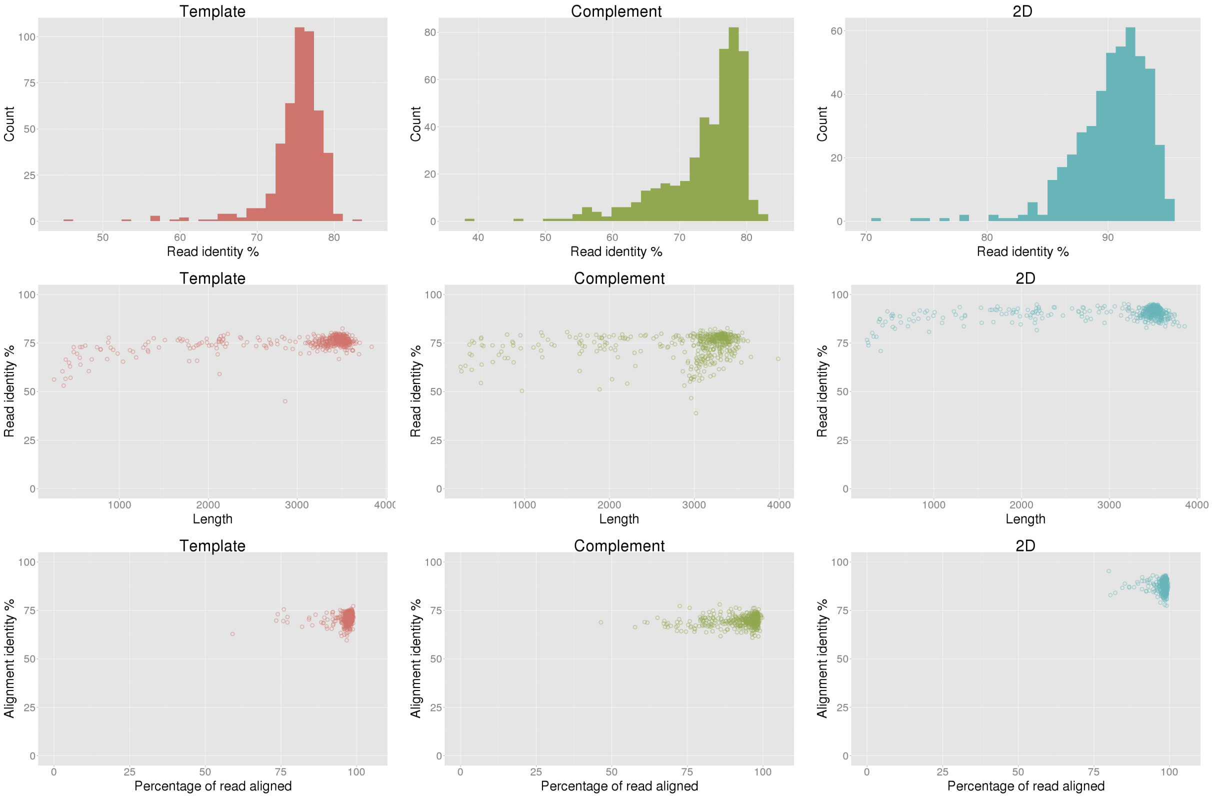

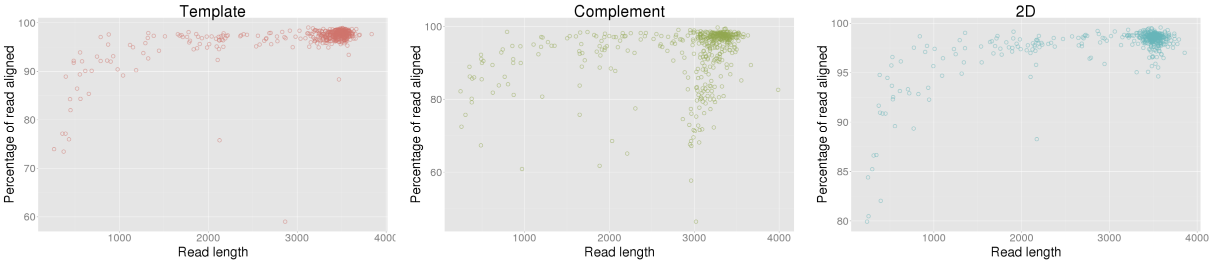

Control sequence perfect kmers

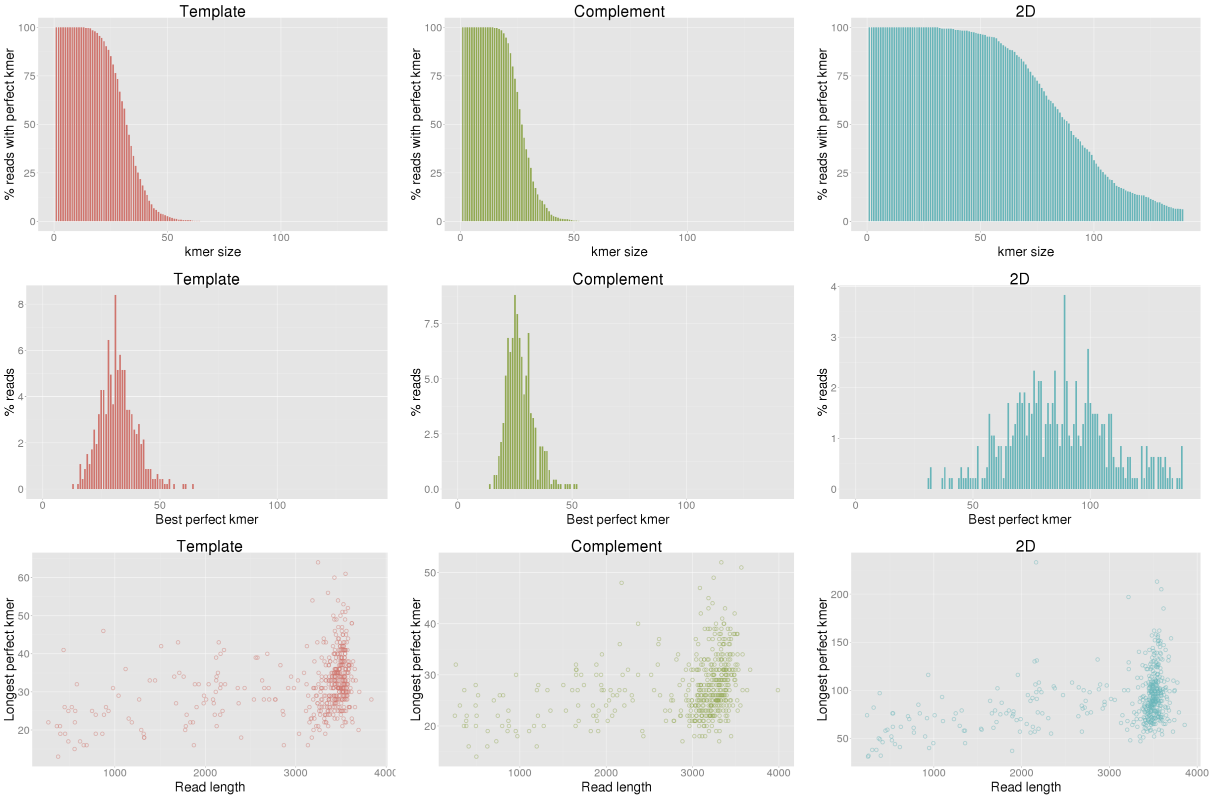

Control sequence coverage

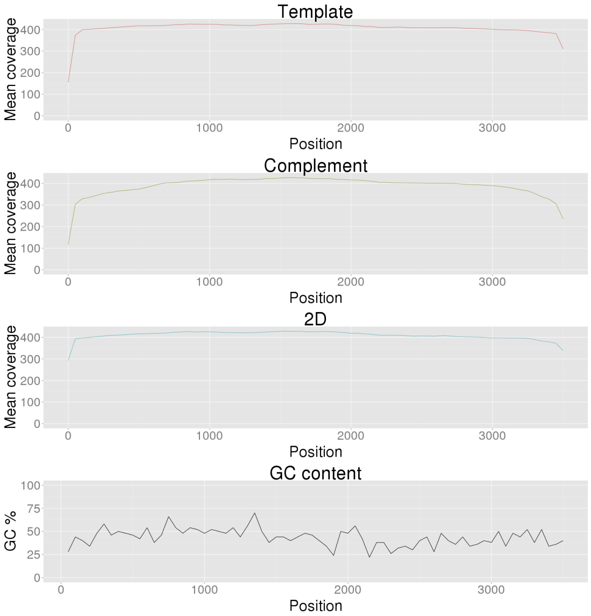

Control sequence 5-mer analysis

Under-represented 5-mers

	Template				Complement				2D			
Rank	kmer	Ref %	Read %	Diff %	kmer	Ref %	Read %	Diff %	kmer	Ref %	Read %	Diff %
1	TTTTT	0.759	0.138	-0.620	TTTTT	0.759	0.089	-0.670	TTTTT	0.759	0.070	-0.689
2	AAAAA	0.478	0.112	-0.365	AAAAA	0.478	0.054	-0.423	AAAAA	0.478	0.091	-0.386
3	TGATG	0.393	0.145	-0.249	AAAAC	0.337	0.145	-0.192	TGATG	0.393	0.189	-0.205
4	GATGT	0.309	0.104	-0.205	GTCAG	0.281	0.117	-0.164	CTGAT	0.309	0.150	-0.159
5	AAAAC	0.337	0.136	-0.201	TGAAG	0.253	0.089	-0.164	GATGT	0.309	0.155	-0.155
6	CTGAT	0.309	0.120	-0.189	GCAAT	0.309	0.146	-0.163	GCTGA	0.281	0.132	-0.149
7	GCTGA	0.281	0.119	-0.162	ACACG	0.197	0.036	-0.160	GATGC	0.281	0.148	-0.133
8	GCAAT	0.309	0.148	-0.161	AACAA	0.281	0.122	-0.159	CTTTT	0.253	0.121	-0.132
9	AATAT	0.309	0.148	-0.161	GATGT	0.309	0.152	-0.157	TGTGA	0.225	0.096	-0.129
10	AACAA	0.281	0.123	-0.158	TTATC	0.309	0.161	-0.148	TTATC	0.309	0.185	-0.124

Over-represented 5-mers

	Template				Complement				2D			
Rank	kmer	Ref %	Read %	Diff %	kmer	Ref %	Read %	Diff %	kmer	Ref %	Read %	Diff %
1	CTTTG	0.028	0.156	0.128	GGAAT	0.112	0.240	0.127	TCAGC	0.028	0.172	0.144
2	CATCT	0.000	0.123	0.123	TATAC	0.000	0.121	0.121	CATCA	0.112	0.257	0.144
3	TCTAC	0.000	0.117	0.117	CTTTG	0.028	0.146	0.117	ATCAG	0.056	0.191	0.135
4	ATCTA	0.000	0.113	0.113	TGCGA	0.028	0.138	0.110	AACCA	0.028	0.158	0.130
5	TACTT	0.000	0.105	0.105	ACCCC	0.000	0.104	0.104	CATCT	0.000	0.116	0.116
6	GCTCC	0.000	0.103	0.103	GAGGA	0.000	0.103	0.103	GCATC	0.084	0.200	0.116
7	AACCA	0.028	0.129	0.101	GGCTT	0.056	0.156	0.100	CAGCA	0.056	0.168	0.112
8	ACCCC	0.000	0.098	0.098	CCCCA	0.000	0.100	0.100	ACCAA	0.028	0.138	0.110
9	TCAGC	0.028	0.126	0.098	TGCTT	0.084	0.184	0.100	TCACA	0.028	0.138	0.110
10	GCGAA	0.028	0.124	0.096	GATTC	0.028	0.127	0.099	TACAT	0.028	0.134	0.106

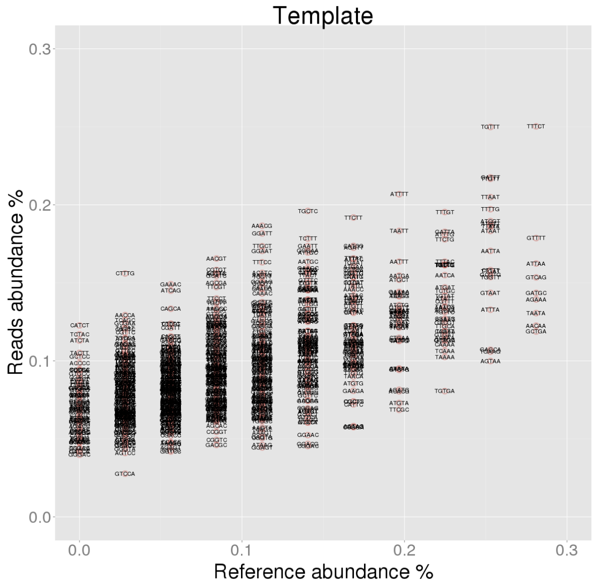

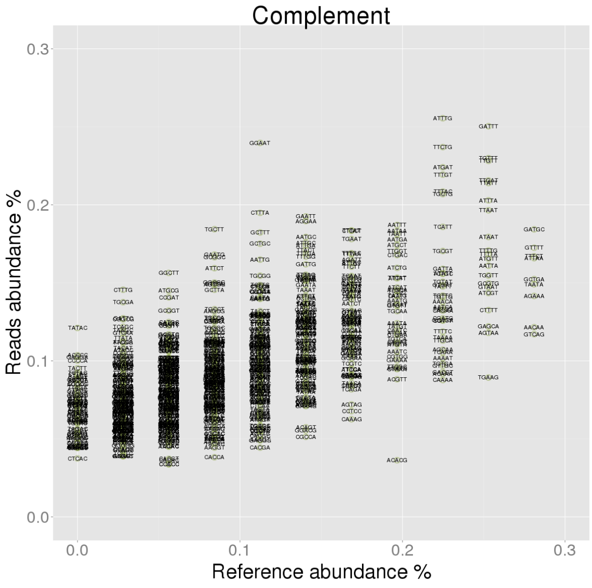

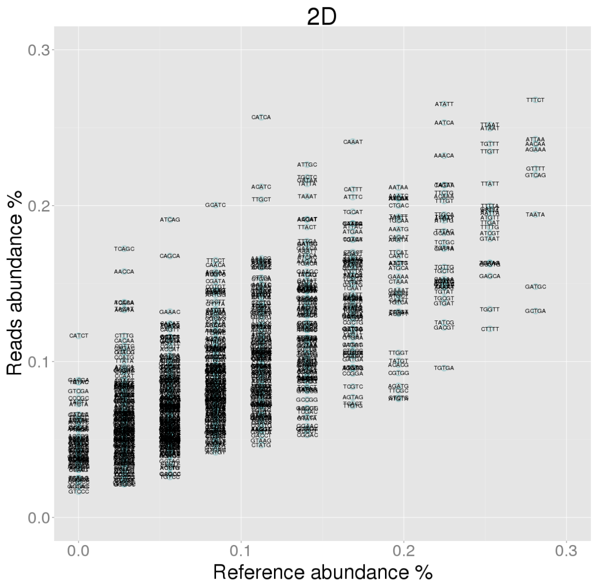

Control sequence GC content

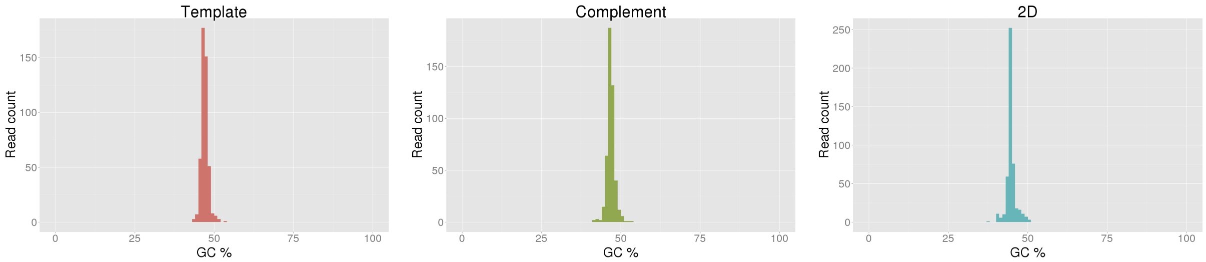

Escherichia coli error analysis

	Template	Complement	2D
Overall base identity (excluding indels)	76.23%	76.42%	91.21%
Aligned base identity (excluding indels)	82.35%	82.66%	95.21%
Identical bases per 100 aligned bases (including indels)	70.42%	69.02%	87.93%
Inserted bases per 100 aligned bases (including indels)	5.32%	4.26%	3.18%
Deleted bases per 100 aligned bases (including indels)	9.18%	12.25%	4.46%
Substitutions per 100 aligned bases (including indels)	15.09%	14.47%	4.42%
Mean insertion size	1.60	1.52	1.53
Mean deletion size	1.61	1.74	1.53

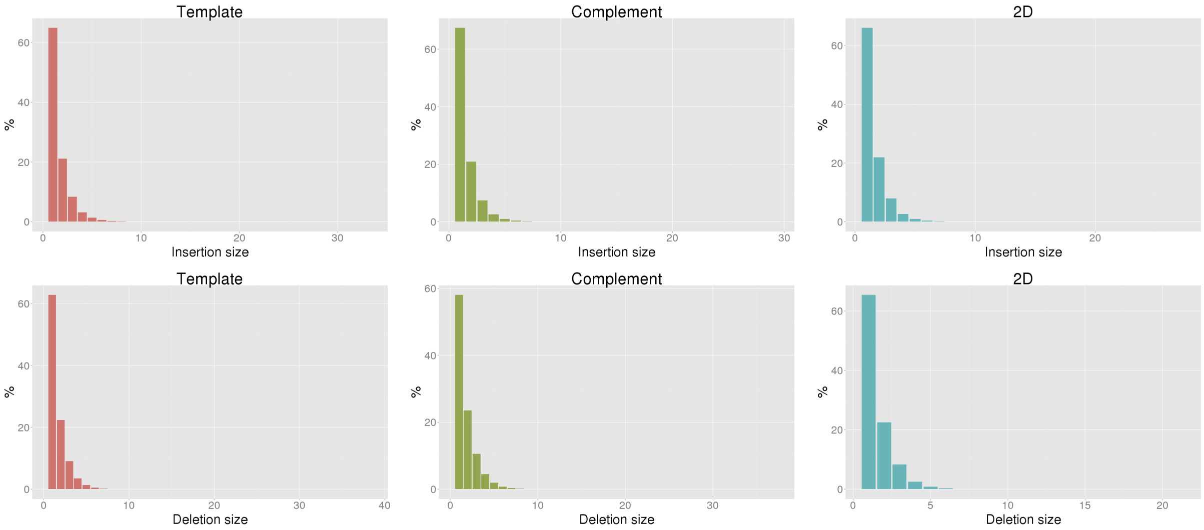

Escherichia coli read identity

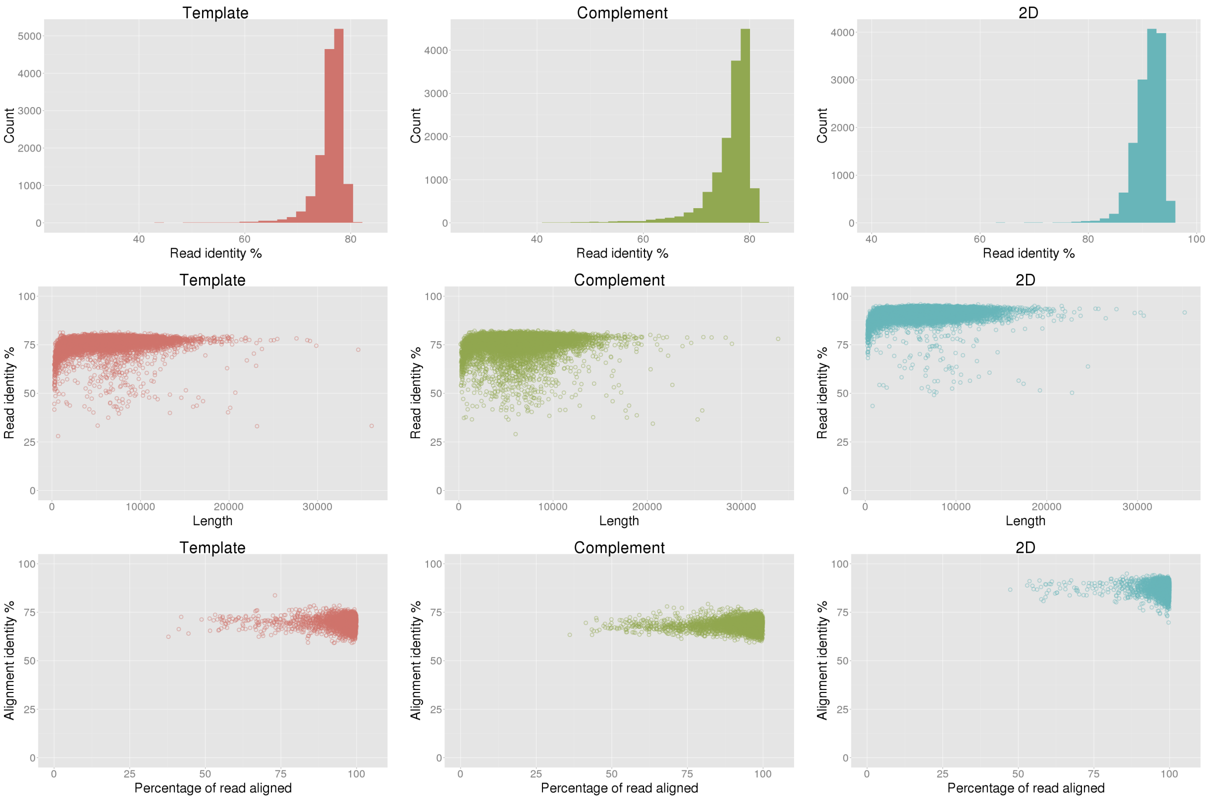

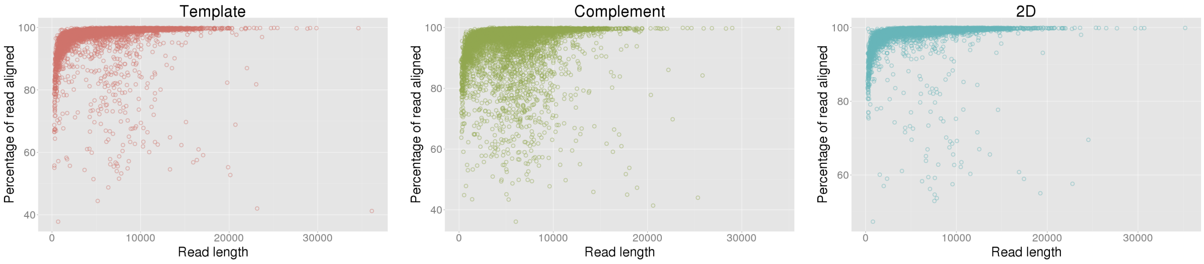

Escherichia coli perfect kmers

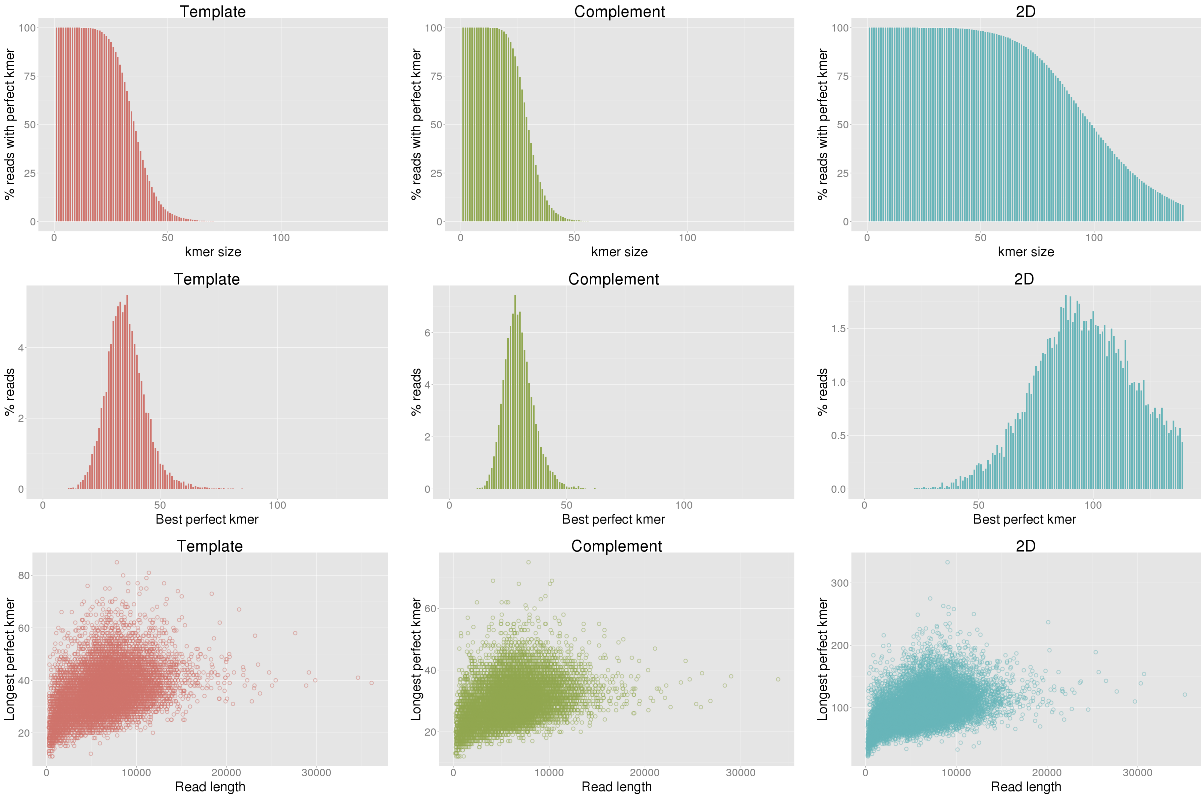

Escherichia coli coverage

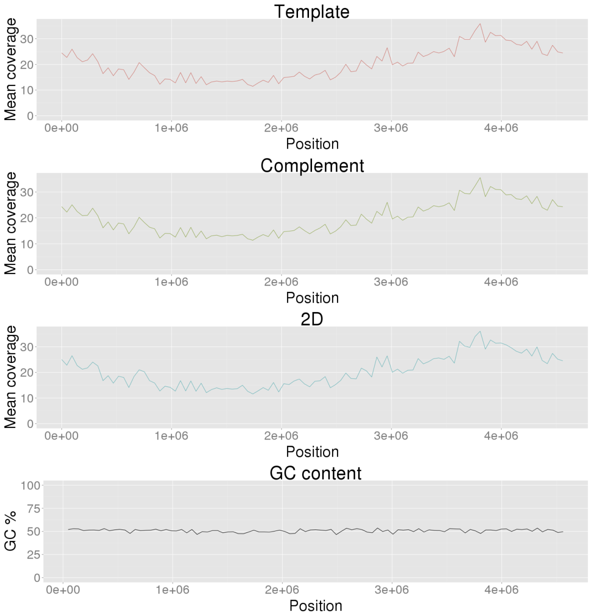

Escherichia coli 5-mer analysis

Under-represented 5-mers

	Template				Complement				2D			
Rank	kmer	Ref %	Read %	Diff %	kmer	Ref %	Read %	Diff %	kmer	Ref %	Read %	Diff %
1	AAAAA	0.247	0.079	-0.168	CGCCA	0.288	0.088	-0.200	TTTTT	0.251	0.046	-0.205
2	TTTTT	0.251	0.085	-0.166	AAAAA	0.247	0.048	-0.199	AAAAA	0.247	0.059	-0.188
3	CGCTG	0.259	0.107	-0.152	TTTTT	0.251	0.059	-0.192	AAAAT	0.195	0.134	-0.061
4	GCTGG	0.279	0.153	-0.127	CACCA	0.184	0.052	-0.132	CAAAA	0.169	0.108	-0.061
5	CGCCA	0.288	0.167	-0.121	CCAGC	0.289	0.160	-0.129	CGCCA	0.288	0.227	-0.061
6	CCAGC	0.289	0.181	-0.108	GCCAG	0.280	0.156	-0.124	CGCTG	0.259	0.202	-0.057
7	GCCAG	0.280	0.176	-0.104	CAGCA	0.261	0.139	-0.122	GCCAG	0.280	0.226	-0.054
8	CTGGC	0.278	0.182	-0.096	CGCTG	0.259	0.138	-0.121	TGGTG	0.185	0.131	-0.054
9	CAGCA	0.261	0.169	-0.093	CTGGC	0.278	0.158	-0.120	GCTGG	0.279	0.227	-0.052
10	CGGCA	0.222	0.137	-0.085	GCGCA	0.202	0.086	-0.115	AAAAG	0.132	0.080	-0.052

Over-represented 5-mers

	Template				Complement				2D			
Rank	kmer	Ref %	Read %	Diff %	kmer	Ref %	Read %	Diff %	kmer	Ref %	Read %	Diff %
1	ACCCC	0.040	0.130	0.090	ACCCC	0.040	0.137	0.097	CAAAT	0.105	0.153	0.048
2	CCCCG	0.055	0.140	0.085	CCCCG	0.055	0.127	0.072	GGGGT	0.039	0.072	0.033
3	CCCCC	0.033	0.113	0.080	CTGAG	0.050	0.112	0.063	CCCAA	0.047	0.076	0.029
4	CCTAG	0.003	0.078	0.075	CCTAG	0.003	0.066	0.063	CGGGG	0.054	0.083	0.029
5	CCCCA	0.064	0.131	0.068	TCCTA	0.013	0.075	0.062	GGGTC	0.040	0.068	0.028
6	TCTAC	0.048	0.114	0.066	TACCC	0.073	0.135	0.061	CTCGT	0.042	0.069	0.026
7	GCCCC	0.062	0.128	0.066	TAGGA	0.012	0.073	0.061	GAAGG	0.094	0.120	0.026
8	CTCCC	0.040	0.103	0.063	CCTAA	0.026	0.086	0.060	CGTGA	0.102	0.128	0.026
9	TAGGA	0.012	0.074	0.062	TACCT	0.062	0.121	0.059	ACCGT	0.123	0.149	0.026
10	CCCCT	0.039	0.101	0.062	GAACC	0.075	0.134	0.059	TCCCA	0.056	0.081	0.025

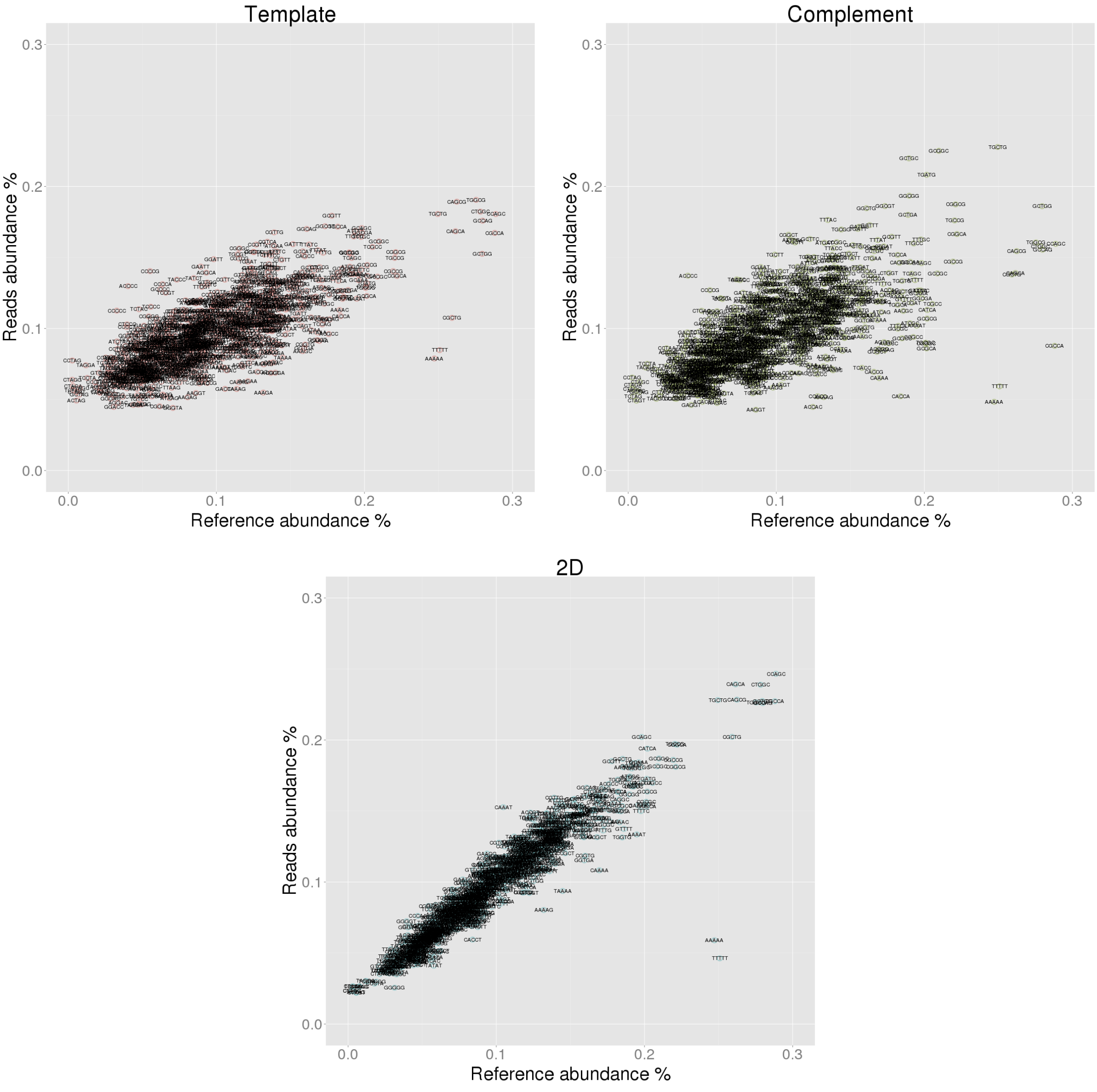

Escherichia coli GC content

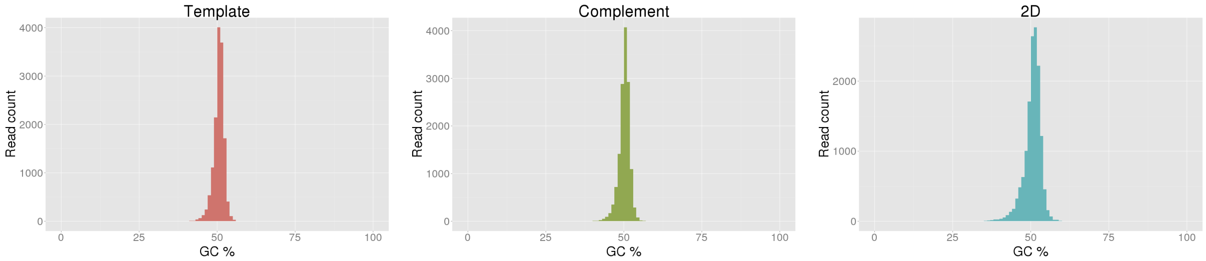

All reference 21mer analysis

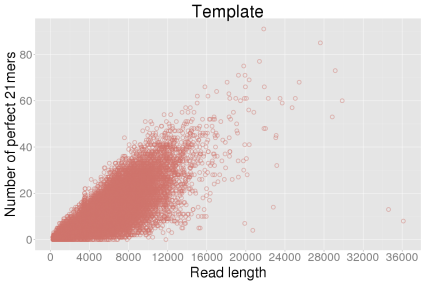

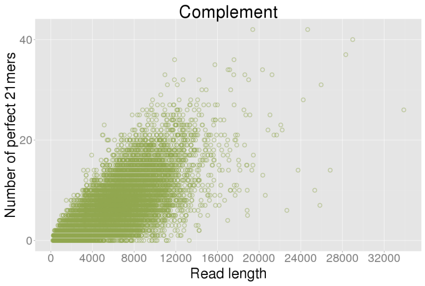

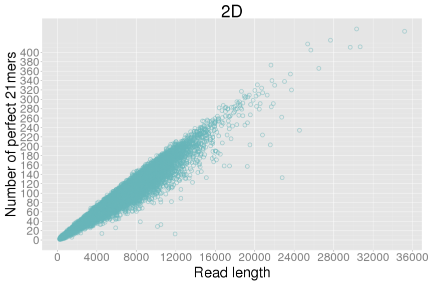

All reference substitutions

		Template substituted %				Complement substituted %				2D substituted %			
		a	c	g	t	a	c	g	t	a	c	g	t
Reference	A	0.00	8.59	9.51	4.78	0.00	8.75	8.81	5.06	0.00	7.94	8.46	4.03
	C	8.29	0.00	9.12	9.80	9.14	0.00	8.70	9.47	9.03	0.00	11.20	9.41
	G	9.82	9.11	0.00	8.25	9.38	8.72	0.00	9.18	9.40	11.16	0.00	9.05
	T	4.78	9.44	8.51	0.00	5.07	8.88	8.83	0.00	3.99	8.41	7.90	0.00

Kmer motifs before errors

3-mer error motif analysis

	Template			Complement			2D			
Rank	Insertion	Deletion	Substitution	Insertion	Deletion	Substitution	Insertion	Deletion	Substitution	
1	TTC (3.13%)	AAA (3.73%)	AAA (3.82%)	TGC (2.95%)	AAA (3.70%)	AAA (3.91%)	AAA (3.00%)	AAA (6.08%)	GCA (3.68%)	Most common
2	AAA (2.97%)	TTC (3.14%)	TTC (3.79%)	AAA (2.85%)	TTT (2.90%)	GCA (3.59%)	GCA (2.87%)	TTT (5.30%)	AAA (3.63%)	
3	TGC (2.70%)	TTT (3.05%)	GCA (3.28%)	GCA (2.73%)	GAA (2.81%)	GAA (3.34%)	TTC (2.58%)	GCG (2.64%)	GAA (3.23%)	
4	GCA (2.67%)	TGC (2.88%)	GAA (2.83%)	CAG (2.56%)	TGC (2.74%)	TTC (2.99%)	GAA (2.53%)	GAA (2.58%)	TTC (2.94%)	
5	TTT (2.53%)	GCC (2.63%)	TGC (2.56%)	GAA (2.56%)	GGC (2.71%)	TGC (2.56%)	CAG (2.52%)	GCC (2.51%)	TTT (2.80%)	
6	TCA (2.51%)	GCA (2.62%)	TTT (2.48%)	TTC (2.51%)	GCA (2.50%)	TTT (2.56%)	GCC (2.51%)	GCA (2.25%)	GCC (2.50%)	
7	GCC (2.45%)	GCG (2.35%)	GCC (2.35%)	TTT (2.36%)	GCC (2.34%)	TCA (2.43%)	TTT (2.46%)	TGC (2.15%)	GCG (2.45%)	
8	ATC (2.42%)	TCA (2.30%)	TCA (2.28%)	TCA (2.29%)	TTC (2.32%)	ATC (2.34%)	TGC (2.39%)	CGC (2.13%)	ATC (2.32%)	
9	CAG (2.21%)	GAA (2.19%)	ATC (2.25%)	GGC (2.29%)	GCG (2.26%)	GGC (2.21%)	TCA (2.36%)	GGC (2.04%)	TCA (2.31%)	
10	GAA (2.17%)	GGC (2.15%)	GGC (2.20%)	GCC (2.23%)	CAG (2.24%)	GCC (2.18%)	GCG (2.33%)	TTC (2.03%)	GTT (2.22%)	
	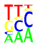	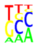	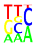	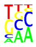	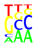	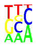	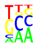	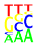	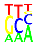	
-10	CTC (1.01%)	TGT (0.96%)	CCT (0.96%)	AGT (0.99%)	CGA (0.97%)	CCC (0.91%)	AGG (1.04%)	AGT (0.96%)	ACT (0.86%)	Least common
-9	GGG (0.93%)	GTA (0.96%)	GGG (0.92%)	AGA (0.98%)	GTG (0.97%)	CTT (0.90%)	GTA (1.04%)	GAG (0.92%)	TAT (0.85%)	
-8	AGT (0.89%)	CTT (0.90%)	AGG (0.82%)	GTG (0.96%)	GGG (0.96%)	AGT (0.88%)	TAT (1.01%)	GTA (0.87%)	TGA (0.82%)	
-7	TGT (0.88%)	AGT (0.86%)	AGT (0.81%)	CTC (0.94%)	GTA (0.95%)	AGG (0.84%)	TGT (1.00%)	CGA (0.74%)	CGA (0.75%)	
-6	GGA (0.82%)	CGA (0.81%)	AGA (0.77%)	GGA (0.88%)	CCT (0.92%)	CCT (0.80%)	AGT (0.95%)	AGA (0.73%)	CTT (0.73%)	
-5	AGA (0.80%)	GGA (0.77%)	CTT (0.74%)	AGG (0.79%)	ACT (0.92%)	GGG (0.77%)	GAG (0.90%)	ACT (0.67%)	GAG (0.69%)	
-4	AGG (0.76%)	GAG (0.73%)	TGT (0.72%)	GGG (0.79%)	CTC (0.82%)	ACT (0.71%)	AGA (0.79%)	CCT (0.65%)	AGA (0.49%)	
-3	GAG (0.71%)	AGA (0.70%)	GAG (0.58%)	GAG (0.78%)	GAG (0.78%)	GAG (0.59%)	GGA (0.67%)	TAG (0.58%)	TAG (0.48%)	
-2	CTA (0.48%)	TAG (0.53%)	CTA (0.38%)	CTA (0.57%)	CTA (0.52%)	CTA (0.47%)	CTA (0.56%)	CTA (0.58%)	GGA (0.46%)	
-1	TAG (0.44%)	CTA (0.47%)	TAG (0.38%)	TAG (0.46%)	TAG (0.51%)	TAG (0.38%)	TAG (0.53%)	GGA (0.56%)	CTA (0.44%)	
	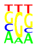	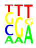	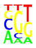	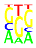	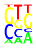	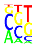	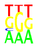	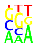	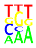	

Kmer space for 3-mers: 64 Random chance for any given 3-mer: 1.56%

4-mer error motif analysis

Rank	Template Insertion	Template Deletion	Template Substitution	Complement Insertion	Complement Deletion	Complement Substitution	2D Insertion	2D Deletion	2D Substitution	
1	TTTC (0.98%)	AAAA (1.46%)	AAAA (1.29%)	CAGC (0.93%)	AAAA (1.10%)	AAAA (1.05%)	CGCC (0.95%)	AAAA (2.54%)	GGCA (1.17%)	Most common
2	AAAA (0.95%)	TTTT (1.27%)	TTTC (1.23%)	CTGC (0.91%)	CAAA (1.08%)	CAAA (1.03%)	CCAG (0.91%)	TTTT (2.46%)	AAAA (1.12%)	
3	ATCA (0.89%)	TGCC (1.01%)	GAAA (1.10%)	ATCA (0.87%)	TGGC (0.97%)	TGAA (0.98%)	ATCA (0.86%)	CAAA (1.48%)	TTTT (1.00%)	
4	GAAA (0.87%)	TTTC (0.96%)	GGCA (0.98%)	TTGC (0.85%)	CAGC (0.95%)	ATCA (0.97%)	CAAA (0.83%)	TAAA (1.20%)	TGAA (0.95%)	
5	TGCC (0.86%)	GAAA (0.93%)	GTTC (0.92%)	CAAA (0.84%)	CGGC (0.94%)	GGCA (0.93%)	GGCA (0.81%)	ATTT (1.06%)	CGCC (0.91%)	
6	TTTT (0.84%)	GTTT (0.91%)	TGCC (0.90%)	CCAG (0.84%)	TTTT (0.94%)	AGCA (0.91%)	TGCC (0.80%)	GAAA (1.03%)	GAAA (0.89%)	
7	CGCC (0.84%)	TTGC (0.86%)	GCAA (0.88%)	CGGC (0.80%)	ATTT (0.88%)	GAAA (0.91%)	GAAA (0.77%)	GTTT (1.02%)	GGAA (0.88%)	
8	TTCA (0.81%)	TTCC (0.85%)	CTTC (0.84%)	TGGC (0.79%)	TAAA (0.87%)	TAAA (0.88%)	CGCA (0.73%)	CTTT (0.94%)	TTTC (0.88%)	
9	GGCA (0.80%)	CAAA (0.83%)	TTTT (0.84%)	GAAA (0.75%)	TGAA (0.84%)	AGAA (0.86%)	AAAA (0.73%)	CGCC (0.89%)	CGCA (0.87%)	
10	TTGC (0.79%)	CGCC (0.83%)	GGAA (0.83%)	TGAA (0.74%)	TTGC (0.84%)	TTTC (0.82%)	CAGC (0.73%)	TGCC (0.80%)	TGCC (0.85%)	
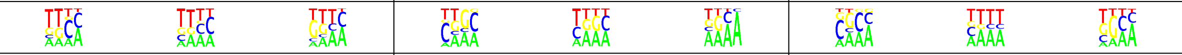										
-10	GTAG (0.13%)	CTAT (0.13%)	TTAG (0.12%)	CTAT (0.11%)	ACCT (0.12%)	TTAG (0.11%)	CCCT (0.14%)	CGGA (0.13%)	TCTA (0.11%)	Least common
-9	TTAG (0.13%)	CGGA (0.13%)	ACTA (0.11%)	CTAA (0.10%)	GTGT (0.12%)	ACCT (0.11%)	GGGA (0.13%)	CCGA (0.13%)	CTAA (0.11%)	
-8	TCTA (0.12%)	GGAC (0.12%)	TAGT (0.10%)	GTGT (0.10%)	GGAC (0.12%)	GGAC (0.10%)	TCTA (0.13%)	ACCT (0.13%)	CCCT (0.10%)	
-7	TAGT (0.11%)	TCTA (0.12%)	CGAG (0.10%)	TAGA (0.09%)	CGAG (0.12%)	GTGT (0.10%)	CTAT (0.13%)	GGGA (0.12%)	CGGA (0.10%)	
-6	CTAA (0.10%)	TAGT (0.12%)	GGAC (0.09%)	GAGG (0.09%)	CTAT (0.11%)	CTAT (0.09%)	TAGT (0.12%)	GACT (0.12%)	GGGA (0.10%)	
-5	GGAC (0.10%)	CTAA (0.11%)	TCTA (0.09%)	GGAC (0.09%)	CCTC (0.11%)	CGAG (0.09%)	CTAA (0.12%)	CTAA (0.12%)	CTAT (0.09%)	
-4	TAGA (0.07%)	TAGG (0.09%)	TAGA (0.07%)	CCCT (0.08%)	TAGG (0.09%)	TAGG (0.07%)	TAGG (0.08%)	CCCT (0.09%)	TAGG (0.08%)	
-3	CCTA (0.06%)	TAGA (0.06%)	TAGG (0.07%)	CCTA (0.07%)	CCCT (0.08%)	CCCT (0.06%)	TAGA (0.08%)	TAGA (0.08%)	TAGA (0.05%)	
-2	TAGG (0.06%)	CCTA (0.05%)	CCTA (0.05%)	TAGG (0.06%)	CCTA (0.06%)	CCTA (0.06%)	CCTA (0.07%)	CCTA (0.06%)	CCTA (0.04%)	
-1	CTAG (0.01%)	CTAG (0.01%)	CTAG (0.01%)	CTAG (0.01%)	CTAG (0.01%)	CTAG (0.01%)	CTAG (0.02%)	CTAG (0.02%)	CTAG (0.01%)	
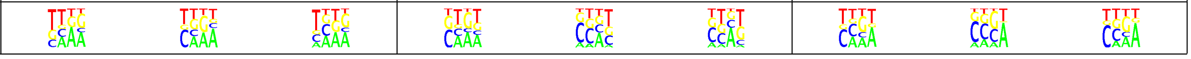										

Kmer space for 4-mers: 256 Random chance for any given 4-mer: 0.39%

5-mer error motif analysis

Rank	Template Insertion	Template Deletion	Template Substitution	Complement Insertion	Complement Deletion	Complement Substitution	2D Insertion	2D Deletion	2D Substitution	
1	CAGCA (0.34%)	GAAAA (0.46%)	GAAAA (0.41%)	CAGCA (0.42%)	GAAAA (0.40%)	CAGCA (0.54%)	CAGCA (0.37%)	ATTTT (0.92%)	CAGCA (0.45%)	Most common
2	CAAAA (0.33%)	ATTTT (0.45%)	CAGCA (0.40%)	GCTGC (0.34%)	CAGCA (0.39%)	CGGCA (0.38%)	GCCAG (0.35%)	GAAAA (0.79%)	CGGCA (0.43%)	
3	CATCA (0.32%)	CAAAA (0.44%)	CAAAA (0.37%)	CATCA (0.33%)	GCAAA (0.36%)	CATCA (0.37%)	CGGCA (0.30%)	CAAAA (0.74%)	TGGCA (0.37%)	
4	GCCAG (0.32%)	CGTTT (0.36%)	CGTTC (0.33%)	CGGCA (0.31%)	CTGGC (0.34%)	ATAAA (0.36%)	GCAAA (0.30%)	CTTTT (0.69%)	GAAAA (0.36%)	
5	CTGGC (0.31%)	TTGCC (0.34%)	TGTTC (0.33%)	CCAGC (0.31%)	ATAAA (0.34%)	GCAAA (0.33%)	CGCCA (0.30%)	TAAAA (0.69%)	ATTTT (0.34%)	
6	CGCCA (0.29%)	TAAAA (0.34%)	CGGCA (0.32%)	TTTGC (0.29%)	ATTTT (0.34%)	CAGAA (0.33%)	TTGCC (0.27%)	GTTTT (0.53%)	CGCCA (0.31%)	
7	GAAAA (0.29%)	CAGCA (0.34%)	GCCAG (0.32%)	CTGGC (0.29%)	GCGGC (0.32%)	GAAAA (0.33%)	TGGCG (0.27%)	GCAAA (0.48%)	CAAAA (0.31%)	
8	ATTTT (0.29%)	CTGGC (0.32%)	TGGCA (0.32%)	GCAAA (0.28%)	CCAGC (0.31%)	AATCA (0.32%)	CATCA (0.26%)	ATAAA (0.45%)	TTGCC (0.30%)	
9	GTTTT (0.29%)	CTTTT (0.32%)	ATTTC (0.32%)	GCCAG (0.27%)	GCTGC (0.30%)	AAGAA (0.32%)	TGGCA (0.26%)	TTTTT (0.39%)	TGGCG (0.29%)	
10	ATTTC (0.28%)	GCAAA (0.30%)	TTGCC (0.31%)	GCGGC (0.27%)	AAGAA (0.29%)	TGGCA (0.31%)	GCGCC (0.26%)	ACAAA (0.39%)	GCAAA (0.29%)	
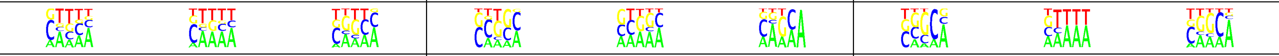										
-10	GGACC (0.01%)	TAGGA (0.01%)	ACCTA (0.01%)	CTAGC (0.01%)	ACCTA (0.01%)	CCCCT (0.01%)	CCCTA (0.01%)	TCCTA (0.01%)	CCCTA (0.01%)	Least common
-9	CCCTA (0.01%)	CCCTA (0.01%)	CCCTA (0.01%)	CCCTA (0.01%)	CTAGC (0.01%)	CCCTA (0.01%)	GCTAG (0.01%)	GCTAG (0.01%)	TAGGA (0.01%)	
-8	ACTAG (0.00%)	ACTAG (0.00%)	ACTAG (0.00%)	ACCTA (0.01%)	CCCTA (0.01%)	CTAGC (0.01%)	TAGGA (0.01%)	TAGGA (0.01%)	CTAGC (0.01%)	
-7	GCTAG (0.00%)	GCTAG (0.00%)	GCTAG (0.00%)	CTAGT (0.01%)	GCTAG (0.01%)	GCTAG (0.01%)	CTAGC (0.01%)	CTAGC (0.01%)	GCTAG (0.00%)	
-6	CTAGC (0.00%)	CTAGC (0.00%)	CTAGC (0.00%)	GCTAG (0.01%)	CTAGT (0.00%)	CTAGT (0.00%)	ACTAG (0.01%)	ACTAG (0.01%)	CTAGT (0.00%)	
-5	CCTAG (0.00%)	CTAGT (0.00%)	CTAGT (0.00%)	ACTAG (0.00%)	ACTAG (0.00%)	CTAGG (0.00%)	CTAGT (0.00%)	CTAGG (0.00%)	ACTAG (0.00%)	
-4	CTAGG (0.00%)	CTAGG (0.00%)	CTAGG (0.00%)	TCTAG (0.00%)	CTAGG (0.00%)	CTAGA (0.00%)	CCTAG (0.00%)	CTAGT (0.00%)	CTAGG (0.00%)	
-3	CTAGT (0.00%)	TCTAG (0.00%)	TCTAG (0.00%)	CTAGA (0.00%)	CTAGA (0.00%)	ACTAG (0.00%)	TCTAG (0.00%)	TCTAG (0.00%)	CCTAG (0.00%)	
-2	CTAGA (0.00%)	CCTAG (0.00%)	CCTAG (0.00%)	CTAGG (0.00%)	TCTAG (0.00%)	TCTAG (0.00%)	CTAGG (0.00%)	CTAGA (0.00%)	TCTAG (0.00%)	
-1	TCTAG (0.00%)	CTAGA (0.00%)	CTAGA (0.00%)	CCTAG (0.00%)	CCTAG (0.00%)	CCTAG (0.00%)	CTAGA (0.00%)	CCTAG (0.00%)	CTAGA (0.00%)	
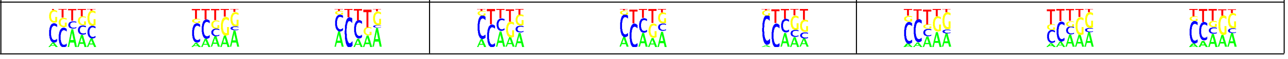										

Kmer space for 5-mers: 1024 Random chance for any given 5-mer: 0.10%
