# Supplementary material for: MinION Analysis and Reference Consortium: Phase 2 data release and analysis of R9.0 chemistry
Source: F1000Res. 2017 May 31;6:760. [Version 1] doi: 10.12688/f1000research.11354.1 (PMC5538040; doi:10.12688/f1000research.11354.1)
Supplement: Supplementary file 3 [file f1000research-6-12257-s0002.tgz › 5d2560fe-dd7a-44d1-b265-e3522e742149.zip!/SI File 3. NanoOK experiment reports/P1b-Lab2-R2-2D_nanook_bwa_failonly.pdf]

NanoOK report for UCSC_MARC_Phase_Ib_Run_2

Pass and fail counts

Type	Pass	Fail
Template	0	33237
Complement	0	19426
2D	0	6569

Read lengths

Type	NumReads	TotalBases	Mean	Longest	Shortest	N50	N50Count	N90	N90Count
Template	33237	150287956	4521.71	244454	5	6563	8088	2911	20696
Complement	19426	92548425	4764.15	50218	10	6424	5253	3130	12941
2D	6569	34153331	5199.17	26972	115	6862	1888	3361	4516

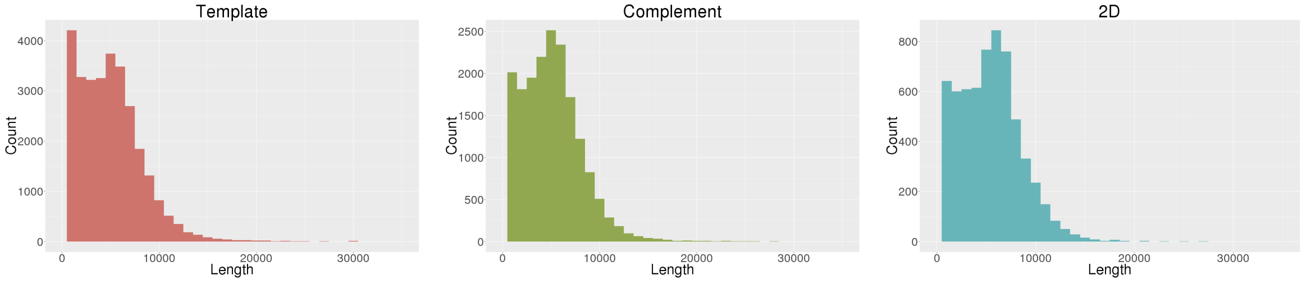

Template alignments

Number of reads	33237	
Number of reads with alignments	15484	(46.59%)
Number of reads without alignments	17753	(53.41%)

ID	Size	Number of Reads	% of Reads	Mean read length	Aligned bases	Mean coverage	Longest Perf Kmer
Control sequence	3560	478	1.44	2859.81	1274411	357.98	49
Escherichia coli	4641652	15006	45.15	6133.36	84870084	18.28	87

Complement alignments

Number of reads	19426	
Number of reads with alignments	11406	(58.72%)
Number of reads without alignments	8020	(41.28%)

ID	Size	Number of Reads	% of Reads	Mean read length	Aligned bases	Mean coverage	Longest Perf Kmer
Control sequence	3560	294	1.51	2643.07	671913	188.74	41
Escherichia coli	4641652	11112	57.20	5813.81	58178568	12.53	57

2D alignments

Number of reads	6569	
Number of reads with alignments	5660	(86.16%)
Number of reads without alignments	909	(13.84%)

ID	Size	Number of Reads	% of Reads	Mean read length	Aligned bases	Mean coverage	Longest Perf Kmer
Control sequence	3560	193	2.94	2661.64	526022	147.76	109
Escherichia coli	4641652	5467	83.22	5692.65	32042968	6.90	230

Control sequence error analysis

	Template	Complement	2D
Overall base identity (excluding indels)	62.50%	56.46%	78.19%
Aligned base identity (excluding indels)	80.10%	80.03%	87.56%
Identical bases per 100 aligned bases (including indels)	67.04%	65.30%	76.36%
Inserted bases per 100 aligned bases (including indels)	4.96%	4.40%	5.04%
Deleted bases per 100 aligned bases (including indels)	11.35%	14.01%	7.76%
Substitutions per 100 aligned bases (including indels)	16.65%	16.29%	10.85%
Mean insertion size	1.56	1.51	1.54
Mean deletion size	1.71	1.86	1.63

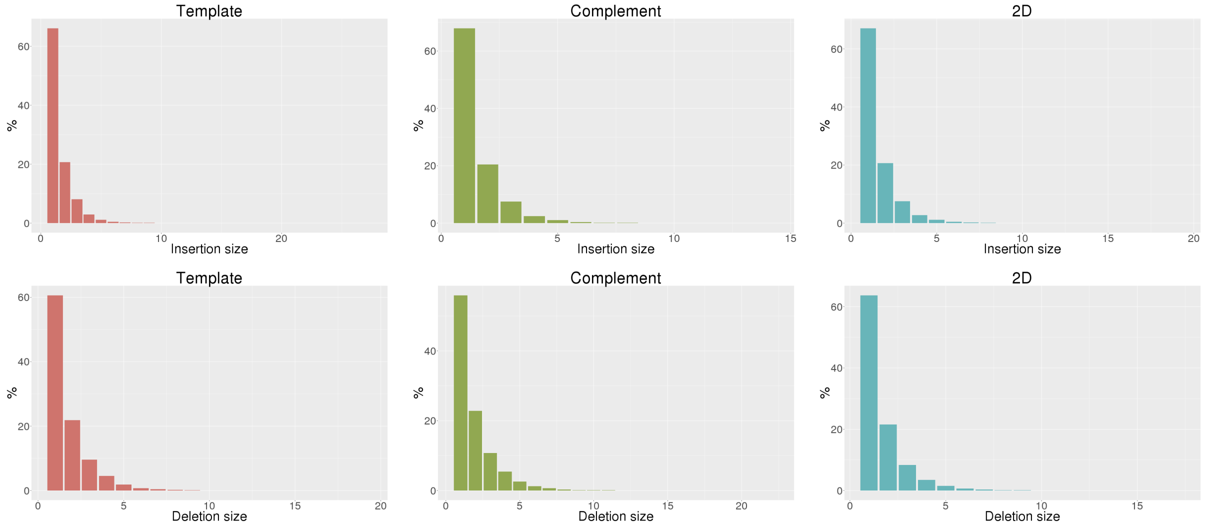

Control sequence read identity

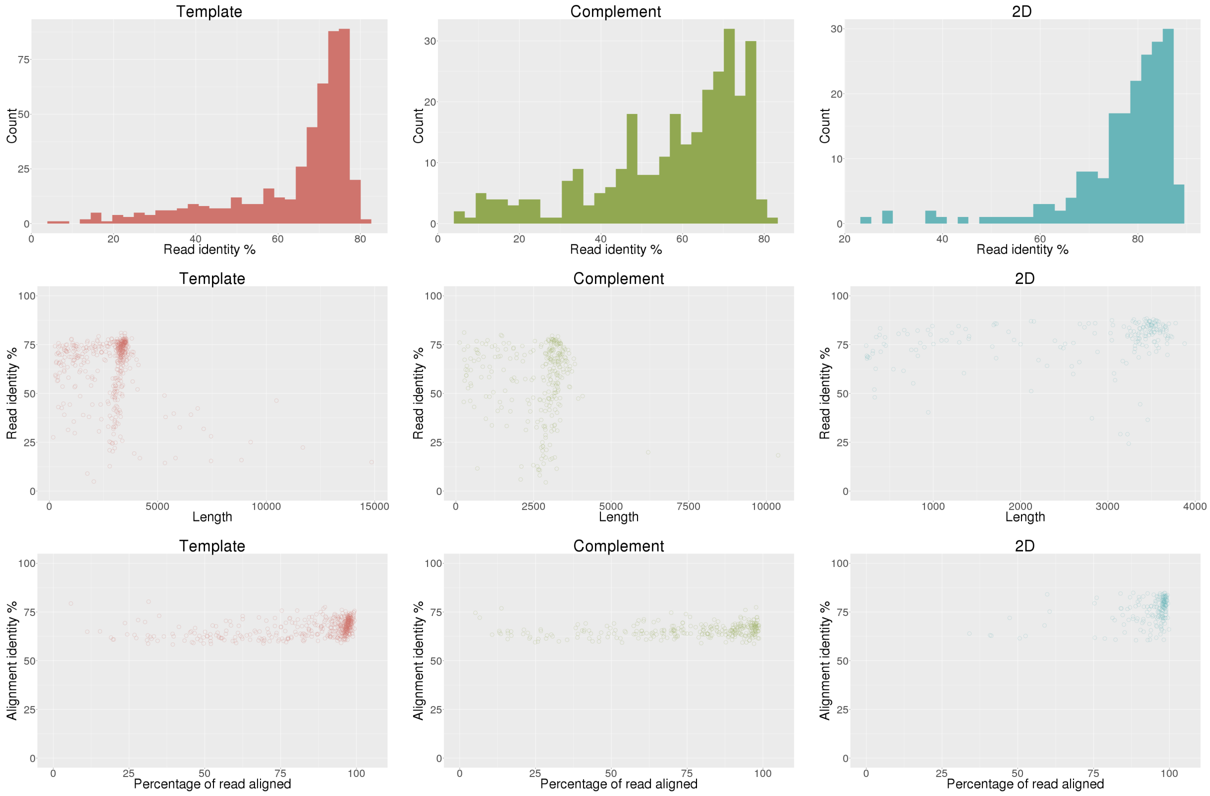

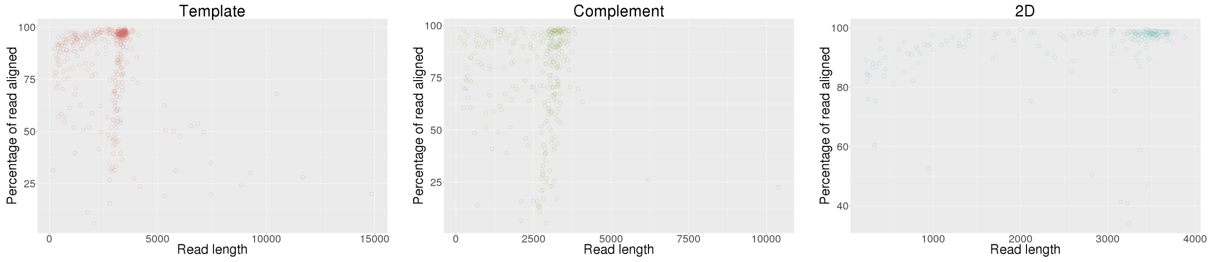

Control sequence perfect kmers

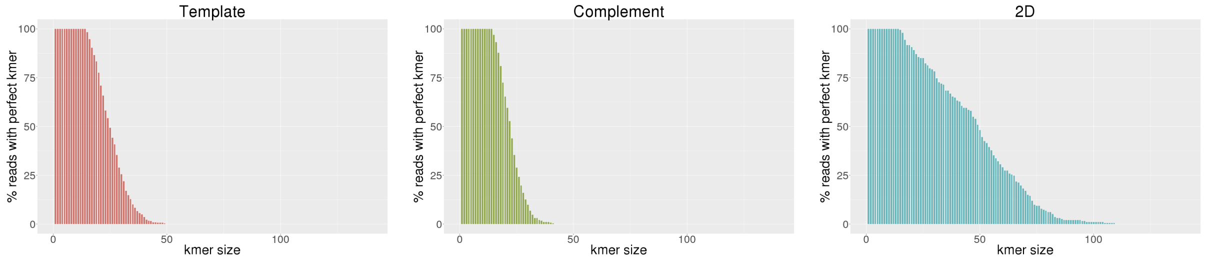

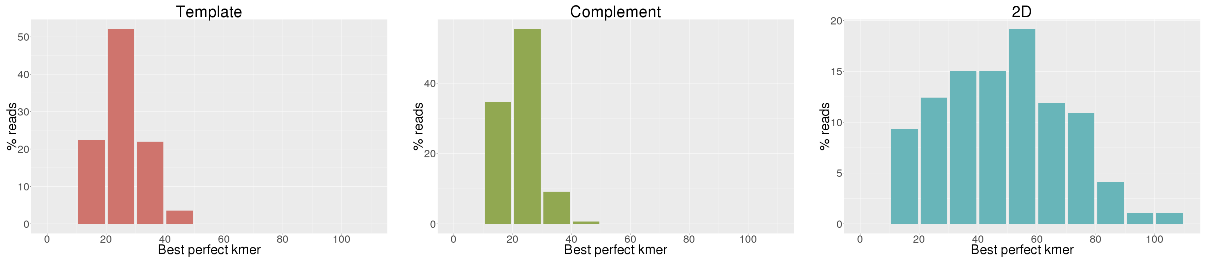

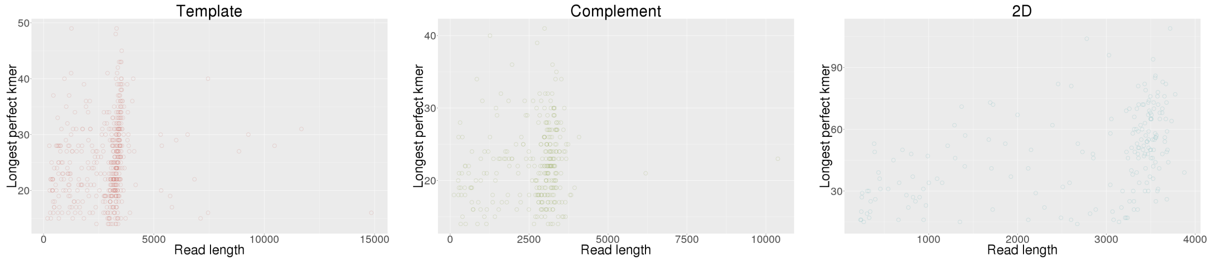

Control sequence coverage

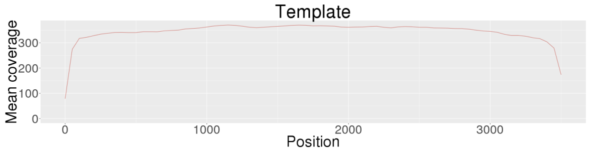

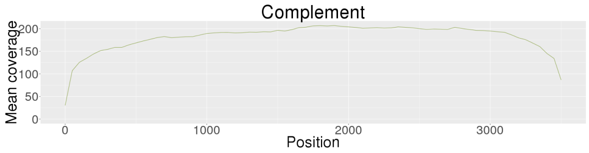

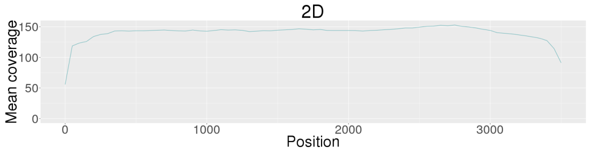

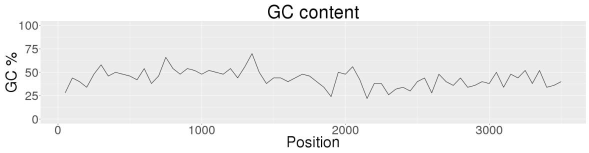

Control sequence 5-mer analysis

Under-represented 5-mers

	Template				Complement				2D			
Rank	kmer	Ref %	Read %	Diff %	kmer	Ref %	Read %	Diff %	kmer	Ref %	Read %	Diff %
1	TTTTT	0.759	0.112	-0.646	TTTTT	0.759	0.092	-0.667	TTTTT	0.759	0.052	-0.707
2	AAAAA	0.478	0.071	-0.407	AAAAA	0.478	0.034	-0.444	AAAAA	0.478	0.053	-0.424
3	TGATG	0.393	0.130	-0.264	AAAAC	0.337	0.101	-0.236	TGATG	0.393	0.151	-0.243
4	AAAAC	0.337	0.108	-0.229	GATGT	0.309	0.114	-0.195	AAAAC	0.337	0.155	-0.182
5	CTGAT	0.309	0.097	-0.212	AACAA	0.281	0.097	-0.184	GATGT	0.309	0.130	-0.180
6	GATGT	0.309	0.098	-0.211	GCAAT	0.309	0.127	-0.182	CTGAT	0.309	0.143	-0.166
7	GCTGA	0.281	0.105	-0.176	TTATC	0.309	0.128	-0.182	GCTGA	0.281	0.133	-0.148
8	AATAT	0.309	0.135	-0.174	TGATG	0.393	0.212	-0.181	TTATC	0.309	0.162	-0.147
9	GCAAT	0.309	0.135	-0.174	AGAAA	0.281	0.100	-0.181	GCAAT	0.309	0.165	-0.144
10	AACAA	0.281	0.109	-0.172	AATAT	0.309	0.141	-0.168	TGATT	0.309	0.169	-0.140

Over-represented 5-mers

	Template				Complement				2D			
Rank	kmer	Ref %	Read %	Diff %	kmer	Ref %	Read %	Diff %	kmer	Ref %	Read %	Diff %
1	CTTTG	0.028	0.156	0.128	GGAAT	0.112	0.247	0.135	TCAGC	0.028	0.134	0.106
2	ATCTA	0.000	0.124	0.124	CTTTG	0.028	0.158	0.130	TCTAC	0.000	0.098	0.098
3	CATCT	0.000	0.119	0.119	TGCTT	0.084	0.207	0.123	ATCTA	0.000	0.098	0.098
4	TACTT	0.000	0.119	0.119	GAGGA	0.000	0.117	0.117	CATCT	0.000	0.098	0.098
5	GCTCC	0.000	0.112	0.112	TACTT	0.000	0.117	0.117	GCATC	0.084	0.179	0.095
6	TCTAC	0.000	0.103	0.103	GGCTT	0.056	0.167	0.111	TACAT	0.028	0.122	0.094
7	TACAT	0.028	0.131	0.102	TATAC	0.000	0.110	0.110	AACCA	0.028	0.120	0.092
8	GAAAC	0.056	0.158	0.102	GCCGA	0.000	0.110	0.110	CTTTG	0.028	0.120	0.091
9	GAGGA	0.000	0.100	0.100	ACCCC	0.000	0.109	0.109	CCCGC	0.000	0.090	0.090
10	GATTC	0.028	0.126	0.098	TGCGA	0.028	0.136	0.107	ACCAA	0.028	0.118	0.090

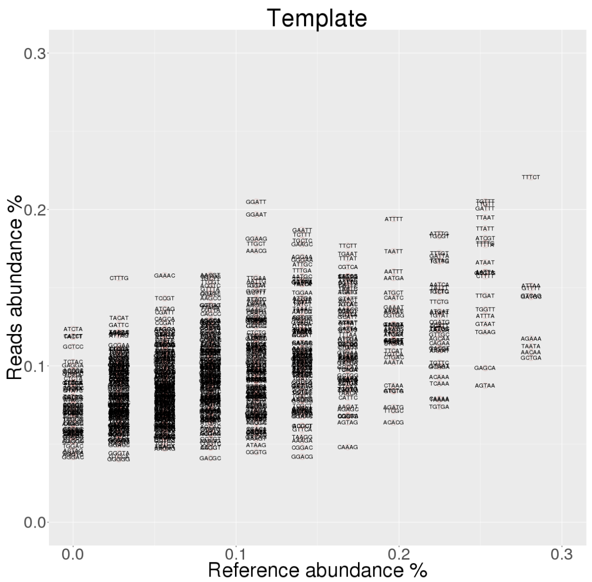

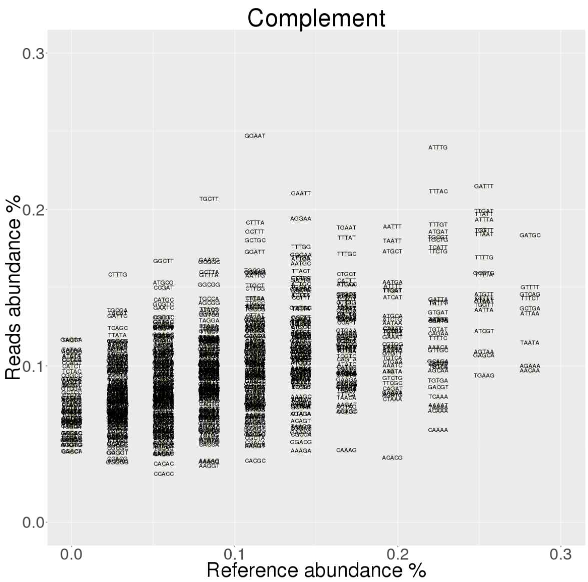

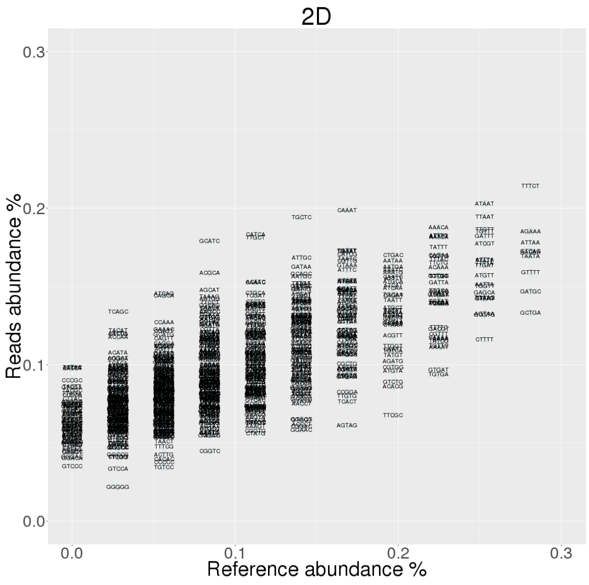

Control sequence GC content

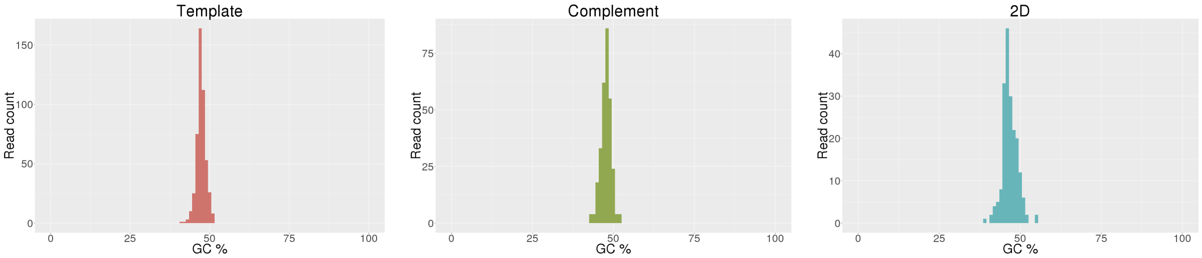

Escherichia coli error analysis

	Template	Complement	2D
Overall base identity (excluding indels)	60.53%	58.57%	78.88%
Aligned base identity (excluding indels)	79.30%	80.31%	87.76%
Identical bases per 100 aligned bases (including indels)	65.64%	65.04%	76.61%
Inserted bases per 100 aligned bases (including indels)	4.81%	4.20%	5.32%
Deleted bases per 100 aligned bases (including indels)	12.42%	14.81%	7.37%
Substitutions per 100 aligned bases (including indels)	17.13%	15.95%	10.69%
Mean insertion size	1.56	1.52	1.59
Mean deletion size	1.76	1.89	1.63

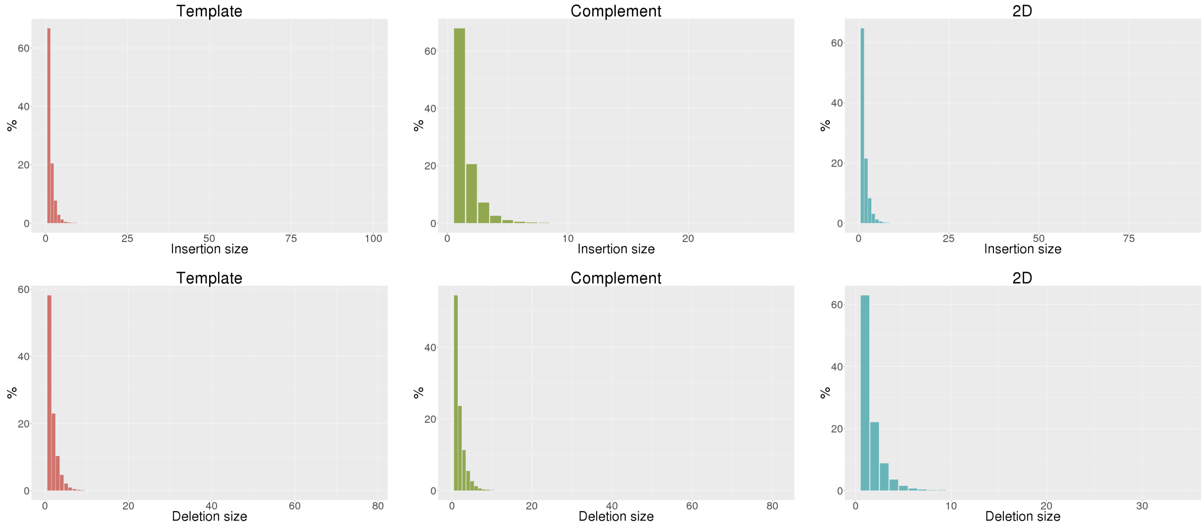

Escherichia coli read identity

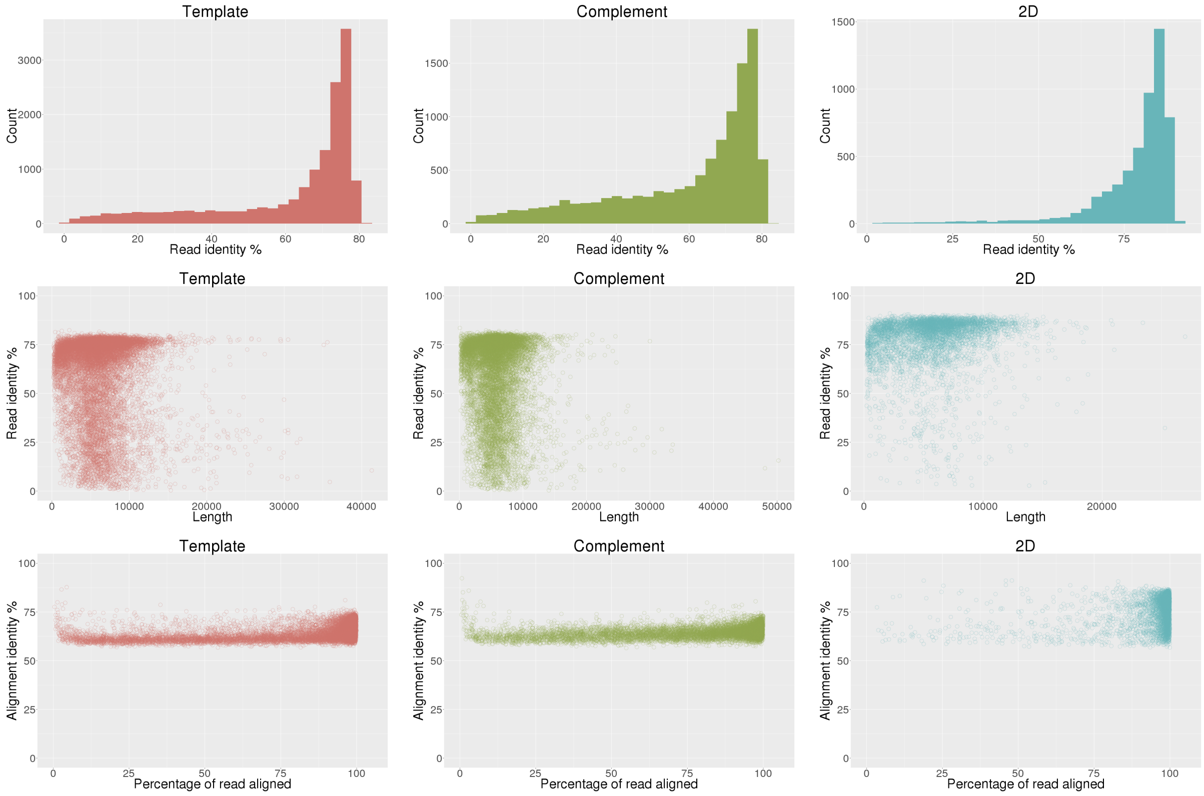

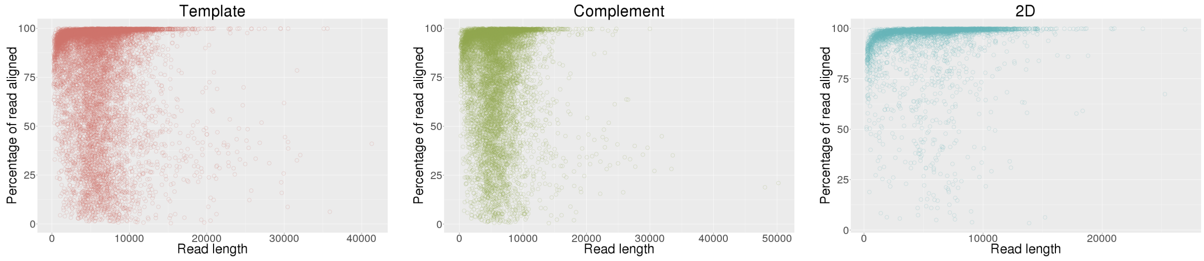

Escherichia coli perfect kmers

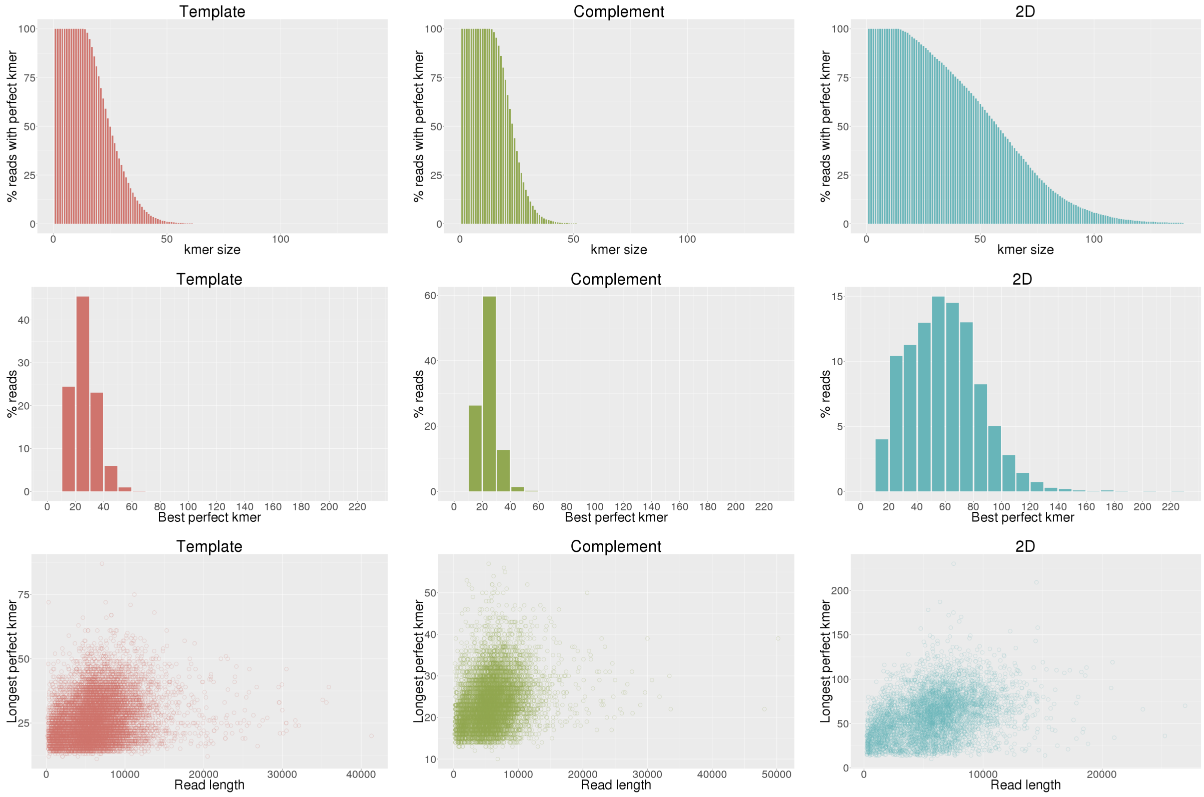

Escherichia coli coverage

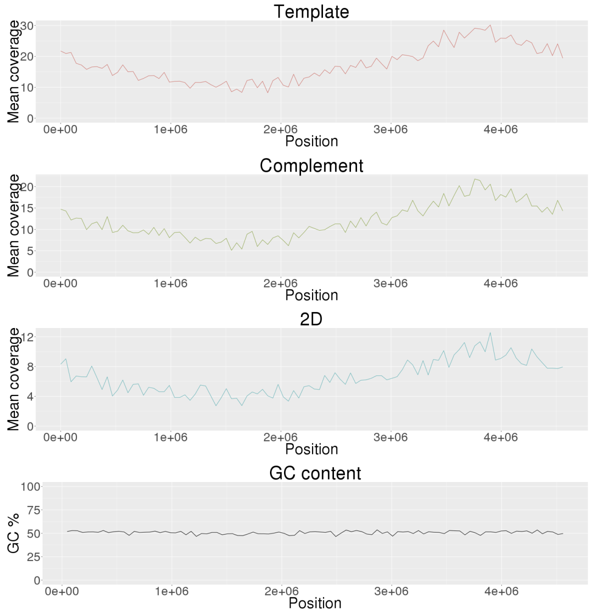

Escherichia coli 5-mer analysis

Under-represented 5-mers

	Template				Complement				2D			
Rank	kmer	Ref %	Read %	Diff %	kmer	Ref %	Read %	Diff %	kmer	Ref %	Read %	Diff %
1	AAAAA	0.247	0.050	-0.197	AAAAA	0.247	0.030	-0.217	TTTTT	0.251	0.040	-0.211
2	CGCTG	0.259	0.084	-0.175	CGCCA	0.288	0.076	-0.212	AAAAA	0.247	0.042	-0.205
3	TTTTT	0.251	0.077	-0.173	TTTTT	0.251	0.053	-0.197	CGCTG	0.259	0.159	-0.100
4	CGCCA	0.288	0.131	-0.157	CTGGC	0.278	0.133	-0.145	CGCCA	0.288	0.189	-0.099
5	GCTGG	0.279	0.127	-0.152	CCAGC	0.289	0.147	-0.142	GCTGG	0.279	0.182	-0.097
6	CTGGC	0.278	0.145	-0.133	CGCGC	0.201	0.061	-0.140	GCCAG	0.280	0.188	-0.092
7	GCCAG	0.280	0.156	-0.123	TGGCG	0.275	0.137	-0.139	CCAGC	0.289	0.201	-0.087
8	CCAGC	0.289	0.168	-0.120	CGCTG	0.259	0.124	-0.135	TGGCG	0.275	0.188	-0.087
9	CGCGC	0.201	0.086	-0.116	CAGCA	0.261	0.129	-0.133	AAAAT	0.195	0.108	-0.087
10	TGGCG	0.275	0.160	-0.115	GCGCA	0.202	0.069	-0.132	TGCGC	0.201	0.123	-0.078

Over-represented 5-mers

	Template				Complement				2D			
Rank	kmer	Ref %	Read %	Diff %	kmer	Ref %	Read %	Diff %	kmer	Ref %	Read %	Diff %
1	GGATT	0.098	0.192	0.094	TAGGA	0.012	0.101	0.089	GGGGT	0.039	0.101	0.062
2	CCTAG	0.003	0.096	0.093	ACCCC	0.040	0.128	0.087	CTAGA	0.003	0.063	0.060
3	TAGGA	0.012	0.101	0.089	GGAAT	0.089	0.167	0.078	TCTAG	0.003	0.063	0.059
4	ACCCC	0.040	0.126	0.086	TCCTA	0.013	0.085	0.072	CTCGT	0.042	0.099	0.057
5	ATCTA	0.033	0.116	0.083	TGCTT	0.099	0.171	0.071	GGGTC	0.040	0.097	0.056
6	TCCGT	0.066	0.148	0.082	CCTAG	0.003	0.073	0.071	CCTAG	0.003	0.058	0.055
7	TCTAA	0.025	0.100	0.075	GAACC	0.075	0.145	0.070	CCCAA	0.047	0.101	0.054
8	GAATT	0.089	0.164	0.075	ATTTA	0.112	0.181	0.070	CTAGG	0.003	0.056	0.053
9	CTCCC	0.040	0.114	0.074	CCTAT	0.028	0.096	0.068	CGGGG	0.054	0.107	0.053
10	TCCTA	0.013	0.087	0.074	TACCT	0.062	0.131	0.068	TCTAA	0.025	0.077	0.052

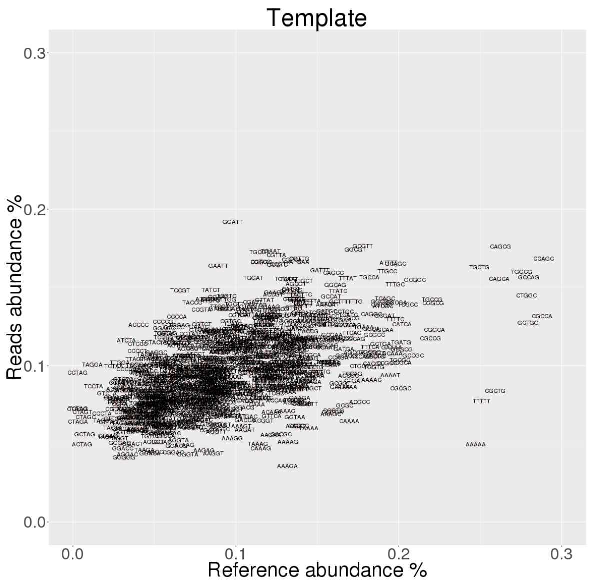

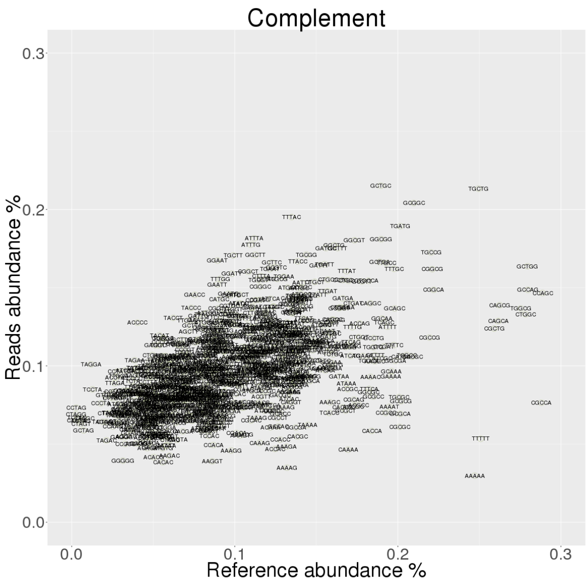

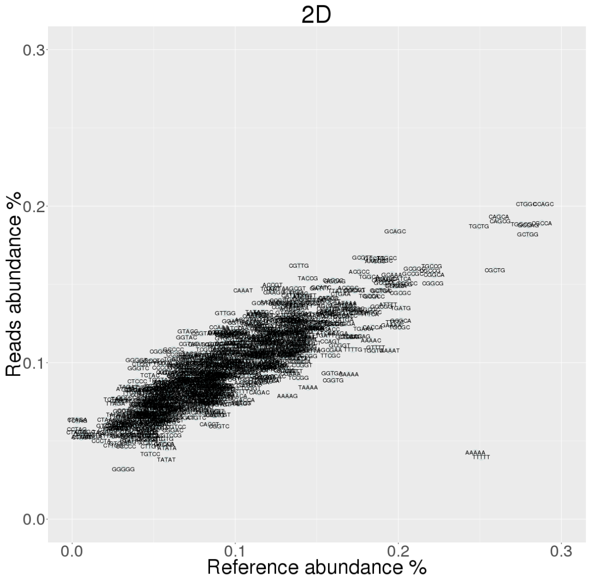

Escherichia coli GC content

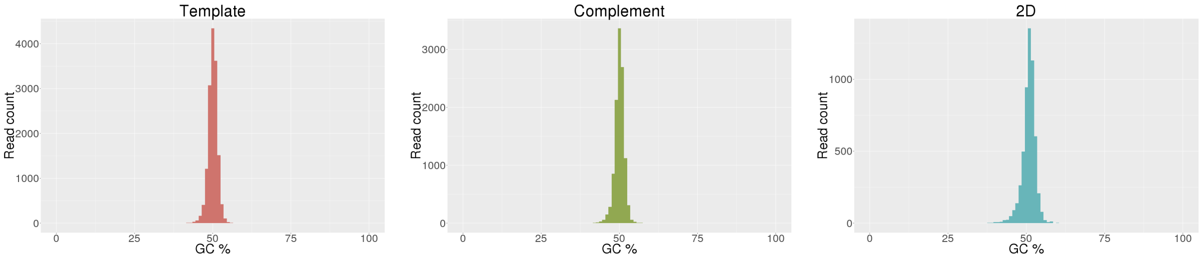

All reference 21mer analysis

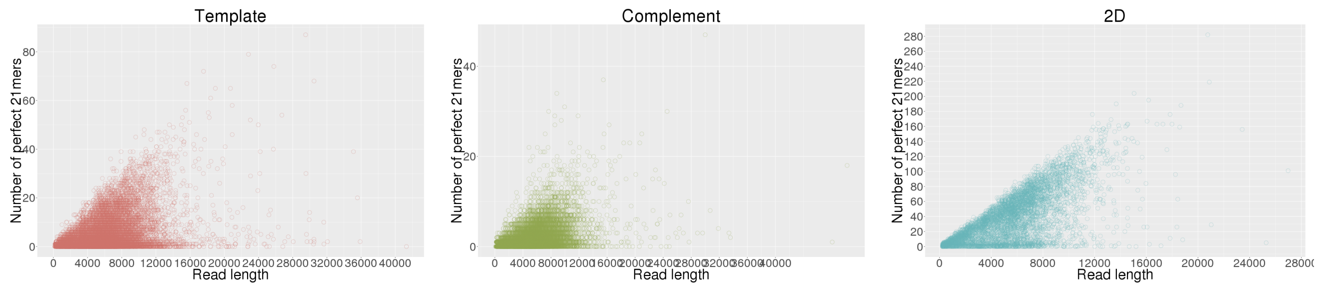

All reference substitutions

		Template substituted %				Complement substituted %				2D substituted %			
		a	c	g	t	a	c	g	t	a	c	g	t
Reference	A	0.00	8.60	8.76	5.11	0.00	8.87	8.66	5.42	0.00	8.96	8.54	4.68
	C	8.69	0.00	8.89	10.18	9.43	0.00	8.56	9.62	8.85	0.00	10.25	9.07
	G	9.61	8.90	0.00	8.45	9.22	8.69	0.00	8.83	9.09	10.29	0.00	8.41
	T	5.34	8.95	8.51	0.00	5.59	8.49	8.62	0.00	4.65	8.48	8.72	0.00

Kmer motifs before errors

3-mer error motif analysis

	Template			Complement			2D			
Rank	Insertion	Deletion	Substitution	Insertion	Deletion	Substitution	Insertion	Deletion	Substitution	
1	TTC (3.26%)	TTC (3.31%)	AAA (3.93%)	TGC (2.95%)	GGC (2.93%)	AAA (3.81%)	GCA (3.04%)	GGC (2.70%)	AAA (3.63%)	Most common
2	GCA (2.78%)	TGC (2.92%)	TTC (3.67%)	TTC (2.85%)	TGC (2.81%)	GCA (3.55%)	AAA (2.76%)	GCA (2.63%)	GCA (3.58%)	
3	AAA (2.77%)	AAA (2.82%)	GCA (3.30%)	GCA (2.78%)	AAA (2.69%)	GAA (3.28%)	TTC (2.75%)	AAA (2.55%)	GAA (2.96%)	
4	TGC (2.57%)	GCA (2.75%)	GAA (2.91%)	AAA (2.65%)	GCA (2.69%)	TTC (3.15%)	TCA (2.52%)	TGC (2.54%)	TTC (2.95%)	
5	ATC (2.55%)	GCC (2.52%)	TTT (2.69%)	GAA (2.51%)	TTC (2.61%)	TTT (2.65%)	GCG (2.38%)	GCG (2.49%)	TTT (2.93%)	
6	TCA (2.41%)	GGC (2.35%)	TGC (2.59%)	CAG (2.40%)	GAA (2.52%)	TGC (2.59%)	GAA (2.33%)	TCA (2.38%)	GTT (2.52%)	
7	GAA (2.30%)	TCA (2.32%)	AAT (2.45%)	ATC (2.31%)	CAG (2.30%)	TCA (2.29%)	TGC (2.32%)	TTC (2.35%)	GCC (2.35%)	
8	TTT (2.22%)	ATC (2.30%)	GCC (2.20%)	GGC (2.31%)	GCC (2.26%)	ATC (2.24%)	ATC (2.29%)	CAG (2.32%)	AAT (2.21%)	
9	GCC (2.21%)	AAT (2.23%)	ATC (2.18%)	TCA (2.29%)	TCA (2.21%)	GCC (2.14%)	TTT (2.22%)	GCC (2.27%)	GCG (2.21%)	
10	GGC (2.15%)	GAA (2.17%)	TCA (2.16%)	TTT (2.17%)	AAT (2.16%)	AAT (2.14%)	CAG (2.20%)	GAA (2.20%)	TGC (2.14%)	
	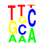	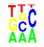	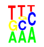	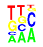	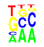	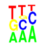	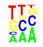	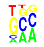	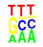	
-10	AGT (0.95%)	GTA (1.00%)	GGT (0.95%)	GTG (0.95%)	AGA (0.95%)	CTT (0.91%)	CTT (1.07%)	GTA (1.06%)	CCT (0.99%)	Least common
-9	GGA (0.92%)	AGT (0.90%)	GGG (0.89%)	GGA (0.95%)	AGT (0.95%)	CCC (0.90%)	CTC (1.03%)	CCC (1.02%)	TGA (0.97%)	
-8	CTC (0.91%)	CCT (0.89%)	AGG (0.87%)	AGA (0.93%)	CTT (0.94%)	CTC (0.87%)	GAG (0.94%)	CGA (0.92%)	ACT (0.93%)	
-7	CCC (0.87%)	CTT (0.86%)	AGA (0.85%)	CTC (0.87%)	CCC (0.93%)	AGG (0.85%)	GGA (0.87%)	ACT (0.88%)	GAG (0.86%)	
-6	AGA (0.82%)	CGA (0.85%)	TGT (0.81%)	CCC (0.80%)	CCT (0.87%)	CCT (0.82%)	AGA (0.86%)	CTT (0.85%)	CGA (0.83%)	
-5	AGG (0.73%)	GAG (0.84%)	CTT (0.79%)	AGG (0.78%)	GAG (0.85%)	GGG (0.78%)	CCC (0.84%)	AGA (0.84%)	CTT (0.80%)	
-4	GAG (0.72%)	GGA (0.72%)	AGT (0.78%)	GAG (0.70%)	CTC (0.84%)	ACT (0.77%)	AGG (0.83%)	CCT (0.80%)	GGA (0.67%)	
-3	GGG (0.69%)	AGA (0.71%)	GAG (0.62%)	GGG (0.58%)	GGG (0.78%)	GAG (0.62%)	GGG (0.77%)	GGA (0.79%)	AGA (0.67%)	
-2	CTA (0.49%)	TAG (0.53%)	TAG (0.40%)	CTA (0.54%)	TAG (0.53%)	CTA (0.42%)	CTA (0.61%)	CTA (0.68%)	TAG (0.50%)	
-1	TAG (0.43%)	CTA (0.49%)	CTA (0.36%)	TAG (0.42%)	CTA (0.52%)	TAG (0.39%)	TAG (0.52%)	TAG (0.66%)	CTA (0.41%)	
	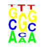	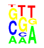	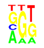	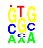	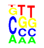	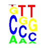	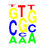	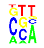	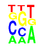	

Kmer space for 3-mers: 64 Random chance for any given 3-mer: 1.56%

4-mer error motif analysis

Rank	Template Insertion	Template Deletion	Template Substitution	Complement Insertion	Complement Deletion	Complement Substitution	2D Insertion	2D Deletion	2D Substitution	
1	TTTC (1.05%)	TTTC (1.09%)	TTTC (1.31%)	CTGC (0.89%)	TGGC (1.06%)	CAAA (1.03%)	ATCA (0.88%)	TGGC (1.02%)	GGCA (1.17%)	Most common
2	GAAA (0.90%)	TGCC (0.97%)	AAAA (1.24%)	ATCA (0.89%)	CGGC (1.06%)	AAAA (0.99%)	GGCA (0.87%)	CAGC (0.91%)	AAAA (1.06%)	
3	TTCA (0.83%)	TTCC (0.90%)	GAAA (1.09%)	CAGC (0.89%)	CAGC (1.02%)	GGCA (0.98%)	GCCA (0.83%)	TTCA (0.83%)	TTTC (1.03%)	
4	ATCA (0.82%)	TTGC (0.88%)	GGCA (0.99%)	TTGC (0.85%)	TTGC (0.88%)	TTTC (0.94%)	TTTC (0.82%)	CGGC (0.78%)	TTTT (0.96%)	
5	AAAA (0.82%)	TGGC (0.88%)	AAAT (0.91%)	CAAA (0.82%)	CTGC (0.87%)	TGAA (0.93%)	TGGC (0.78%)	CGCC (0.78%)	GAAA (0.93%)	
6	GGCA (0.79%)	TTCA (0.87%)	GGAA (0.89%)	TGGC (0.80%)	TTCC (0.83%)	GAAA (0.89%)	AACA (0.78%)	CCAG (0.76%)	GGAA (0.91%)	
7	TGCC (0.78%)	CAGC (0.86%)	TTTT (0.89%)	CGGC (0.80%)	CAAA (0.81%)	GGAA (0.89%)	TTCA (0.77%)	CTGC (0.76%)	CGTT (0.88%)	
8	TTGC (0.78%)	CTGC (0.85%)	GCAA (0.87%)	CCAG (0.78%)	TGCC (0.81%)	AGCA (0.88%)	GAAA (0.77%)	TGCC (0.75%)	TGCA (0.84%)	
9	CATC (0.77%)	AAAA (0.83%)	TGCC (0.86%)	ATGC (0.77%)	ATCA (0.80%)	ATCA (0.87%)	CAGC (0.76%)	GGCG (0.75%)	CGCC (0.83%)	
10	CTTC (0.77%)	GAAA (0.81%)	GTTC (0.86%)	TGCC (0.75%)	CCAG (0.76%)	AGAA (0.83%)	CGCC (0.75%)	GGCA (0.74%)	TGCC (0.81%)	
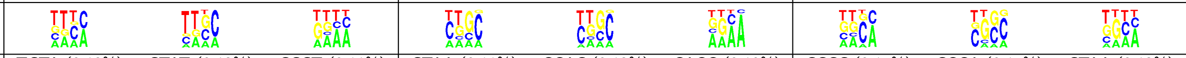										
-10	TCTA (0.13%)	CTAT (0.12%)	CCCT (0.11%)	CTAA (0.11%)	GGAC (0.12%)	GAGG (0.10%)	CCCC (0.15%)	CGGA (0.15%)	CTAA (0.12%)	Least common
-9	TTAG (0.12%)	CGGA (0.12%)	ACTA (0.10%)	CGAG (0.10%)	CCTC (0.11%)	GTGT (0.10%)	CCCT (0.15%)	CTAT (0.15%)	CGGA (0.11%)	
-8	CTAA (0.11%)	TAGT (0.12%)	CGAG (0.09%)	GAGG (0.10%)	CCCC (0.11%)	ACTA (0.10%)	CTAT (0.15%)	ACTT (0.15%)	CCCT (0.11%)	
-7	TAGT (0.11%)	GGAC (0.11%)	TAGA (0.09%)	GTGT (0.10%)	CTAA (0.11%)	GGAC (0.09%)	ACTA (0.14%)	ACCT (0.14%)	ACTT (0.11%)	
-6	GAGG (0.11%)	CCCT (0.11%)	TAGT (0.09%)	GGAC (0.09%)	TAGA (0.11%)	CTAT (0.09%)	GGAC (0.13%)	CTAA (0.12%)	TCTA (0.10%)	
-5	GGAC (0.10%)	CTAA (0.10%)	TCTA (0.08%)	TAGA (0.09%)	GTGT (0.10%)	CGAG (0.09%)	CTAA (0.11%)	CCCT (0.10%)	ACTA (0.10%)	
-4	TAGA (0.07%)	TAGG (0.08%)	TAGG (0.07%)	CCCT (0.08%)	TAGG (0.08%)	TAGG (0.07%)	TAGA (0.08%)	TAGG (0.10%)	TAGG (0.08%)	
-3	CCTA (0.06%)	TAGA (0.06%)	GGAC (0.07%)	CCTA (0.06%)	CCCT (0.07%)	CCCT (0.07%)	CCTA (0.08%)	TAGA (0.09%)	TAGA (0.06%)	
-2	TAGG (0.06%)	CCTA (0.05%)	CCTA (0.05%)	TAGG (0.06%)	CCTA (0.06%)	CCTA (0.05%)	TAGG (0.06%)	CCTA (0.09%)	CCTA (0.05%)	
-1	CTAG (0.01%)	CTAG (0.01%)	CTAG (0.01%)	CTAG (0.01%)	CTAG (0.01%)	CTAG (0.01%)	CTAG (0.01%)	CTAG (0.02%)	CTAG (0.02%)	
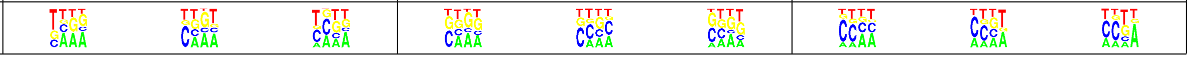										

Kmer space for 4-mers: 256 Random chance for any given 4-mer: 0.39%

5-mer error motif analysis

Rank	Template Insertion	Template Deletion	Template Substitution	Complement Insertion	Complement Deletion	Complement Substitution	2D Insertion	2D Deletion	2D Substitution	
1	CAGCA (0.34%)	CTGGC (0.36%)	CAGCA (0.41%)	CAGCA (0.42%)	CAGCA (0.43%)	CAGCA (0.54%)	CAGCA (0.37%)	CTGGC (0.42%)	TGGCA (0.44%)	Most common
2	ATTTC (0.32%)	CAGCA (0.35%)	GAAAA (0.40%)	GCTGC (0.34%)	CTGGC (0.40%)	CGGCA (0.40%)	CGCCA (0.36%)	CAGCA (0.32%)	CAGCA (0.44%)	
3	CATCA (0.30%)	TTGCC (0.33%)	CAAAA (0.36%)	CATCA (0.33%)	GCGGC (0.37%)	CATCA (0.36%)	CTGGC (0.34%)	TGGCG (0.31%)	CGGCA (0.39%)	
4	CTGGC (0.30%)	GCTGC (0.31%)	TGGCA (0.34%)	CGGCA (0.30%)	CCAGC (0.33%)	ATAAA (0.33%)	TGGCG (0.32%)	GCCAG (0.31%)	GAAAA (0.37%)	
5	TTATC (0.30%)	TTTGC (0.31%)	CGTTC (0.33%)	GCGGC (0.30%)	CATCA (0.33%)	GAAAA (0.33%)	CGGCA (0.31%)	CGCCA (0.30%)	GCGTT (0.35%)	
6	CGCCA (0.29%)	GCCAG (0.30%)	CGGCA (0.32%)	CTGGC (0.29%)	GCTGC (0.31%)	TGGCA (0.32%)	TGGCA (0.31%)	CCAGC (0.29%)	ATTTT (0.31%)	
7	TGGCG (0.29%)	CGCCA (0.30%)	CGTTT (0.32%)	AATCA (0.29%)	AATCA (0.31%)	GCAAA (0.31%)	CATCA (0.29%)	GCGGC (0.28%)	CAAAA (0.30%)	
8	TTTGC (0.28%)	TTTCC (0.30%)	TGTTC (0.31%)	GCAAA (0.29%)	TTTGC (0.29%)	AAGAA (0.31%)	GCCAG (0.29%)	TTTCA (0.28%)	TTGCC (0.30%)	
9	TGGCA (0.27%)	CCAGC (0.29%)	ATTTC (0.30%)	CCAGC (0.28%)	GCTGG (0.29%)	AATCA (0.31%)	CCAGC (0.28%)	TGGCA (0.27%)	CGCCA (0.30%)	
10	GCAGC (0.27%)	GAAAA (0.29%)	TTTGC (0.30%)	GATGC (0.28%)	TTGCC (0.28%)	ACGCA (0.31%)	GCAAA (0.27%)	TTGCC (0.26%)	GCCAG (0.28%)	
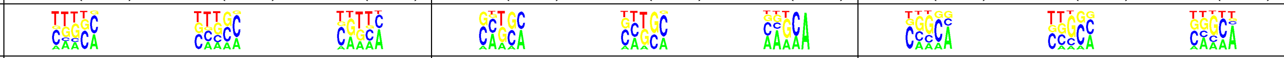										
-10	CCCTA (0.01%)	CCCTA (0.01%)	CTTAG (0.01%)	CCCTA (0.01%)	GGGGG (0.01%)	ACCTA (0.01%)	GGACC (0.01%)	TCCTA (0.01%)	CCCTA (0.01%)	Least common
-9	GGACC (0.00%)	GGACC (0.01%)	CCCTA (0.01%)	ACCTA (0.01%)	CCCCC (0.01%)	CCCTA (0.01%)	CCCTA (0.01%)	TAGGA (0.01%)	TAGGA (0.01%)	
-8	GCTAG (0.00%)	GCTAG (0.00%)	ACTAG (0.00%)	CTAGC (0.01%)	CTAGC (0.01%)	GCTAG (0.00%)	CTAGC (0.00%)	GCTAG (0.01%)	GCTAG (0.00%)	
-7	CTAGC (0.00%)	ACTAG (0.00%)	GCTAG (0.00%)	GCTAG (0.00%)	GCTAG (0.01%)	CTAGC (0.00%)	CTAGT (0.00%)	ACTAG (0.01%)	ACTAG (0.00%)	
-6	ACTAG (0.00%)	CTAGC (0.00%)	CTAGC (0.00%)	ACTAG (0.00%)	CTAGT (0.00%)	CTAGT (0.00%)	CCTAG (0.00%)	CTAGC (0.01%)	CTAGT (0.00%)	
-5	CTAGG (0.00%)	CTAGT (0.00%)	CTAGG (0.00%)	CTAGT (0.00%)	ACTAG (0.00%)	CCTAG (0.00%)	GCTAG (0.00%)	CTAGT (0.00%)	CTAGG (0.00%)	
-4	CTAGA (0.00%)	CTAGG (0.00%)	CTAGT (0.00%)	CTAGG (0.00%)	CTAGA (0.00%)	TCTAG (0.00%)	ACTAG (0.00%)	CTAGG (0.00%)	CTAGC (0.00%)	
-3	CCTAG (0.00%)	TCTAG (0.00%)	CTAGA (0.00%)	CCTAG (0.00%)	TCTAG (0.00%)	ACTAG (0.00%)	CTAGG (0.00%)	TCTAG (0.00%)	CCTAG (0.00%)	
-2	CTAGT (0.00%)	CCTAG (0.00%)	CCTAG (0.00%)	TCTAG (0.00%)	CTAGG (0.00%)	CTAGG (0.00%)	CTAGA (0.00%)	CCTAG (0.00%)	TCTAG (0.00%)	
-1	TCTAG (0.00%)	CTAGA (0.00%)	TCTAG (0.00%)	CTAGA (0.00%)	CCTAG (0.00%)	CTAGA (0.00%)	TCTAG (0.00%)	CTAGA (0.00%)	CTAGA (0.00%)	
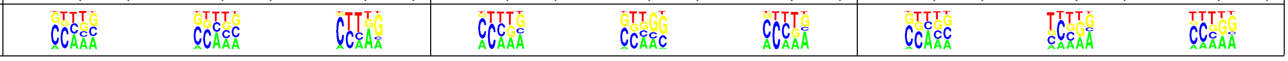										

Kmer space for 5-mers: 1024 Random chance for any given 5-mer: 0.10%
