# Supplementary material for: MinION Analysis and Reference Consortium: Phase 2 data release and analysis of R9.0 chemistry
Source: F1000Res. 2017 May 31;6:760. [Version 1] doi: 10.12688/f1000research.11354.1 (PMC5538040; doi:10.12688/f1000research.11354.1)
Supplement: Supplementary file 3 [file f1000research-6-12257-s0002.tgz › 5d2560fe-dd7a-44d1-b265-e3522e742149.zip!/SI File 3. NanoOK experiment reports/P2-Lab6_R1_1D_nanook_bwa_failonly.pdf]

NanoOK report for UBC_MARC_1D_R9_107_Called

Pass and fail counts

Type	Pass	Fail
Template	0	39259

Read lengths

Type	NumReads	TotalBases	Mean	Longest	Shortest	N50	N50Count	N90	N90Count
Template	39259	303312093	7725.93	478773	5	14797	5959	4095	20780

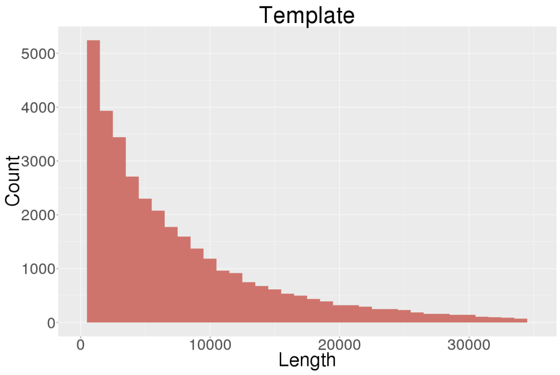

Template alignments

Number of reads	39259	
Number of reads with alignments	24578	(62.60%)
Number of reads without alignments	14681	(37.40%)

ID	Size	Number of Reads	% of Reads	Mean read length	Aligned bases	Mean coverage	Longest Perf Kmer
Control sequence	3560	0	0.00	0.00	0	0.00	0
Escherichia coli	4641652	24578	62.60	10089.79	202279583	43.58	124

Escherichia coli error analysis

	Template
Overall base identity (excluding indels)	55.79%
Aligned base identity (excluding indels)	80.99%
Identical bases per 100 aligned bases (including indels)	68.40%
Inserted bases per 100 aligned bases (including indels)	4.27%
Deleted bases per 100 aligned bases (including indels)	11.27%
Substitutions per 100 aligned bases (including indels)	16.06%
Mean insertion size	1.53
Mean deletion size	1.79

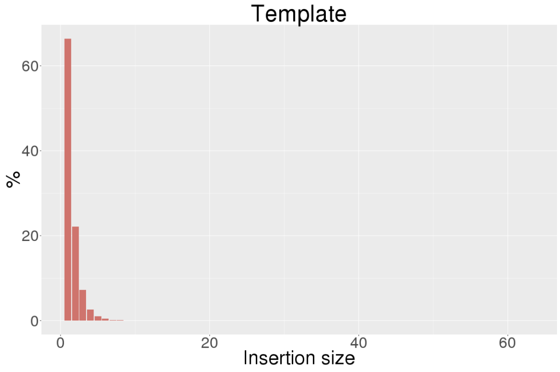

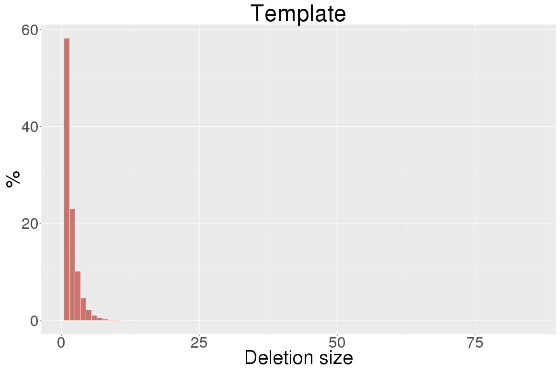

Escherichia coli read identity

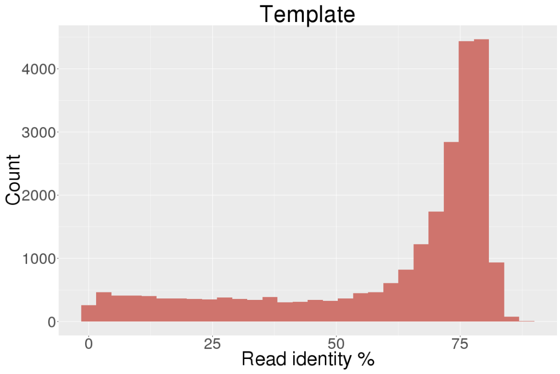

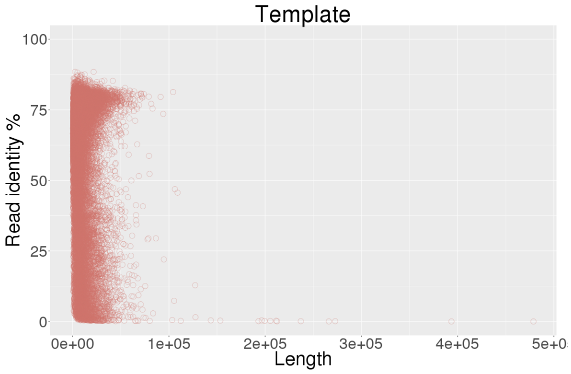

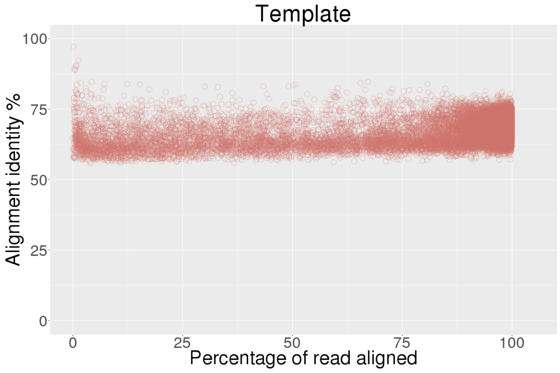

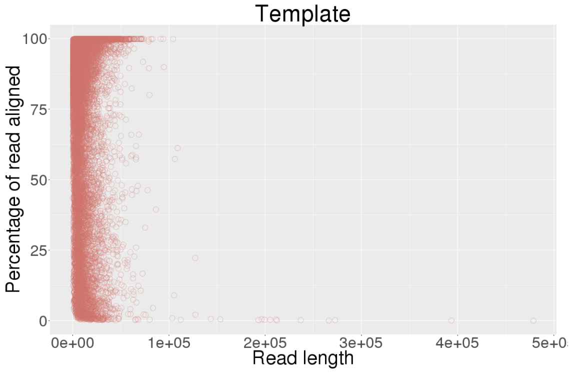

Escherichia coli perfect kmers

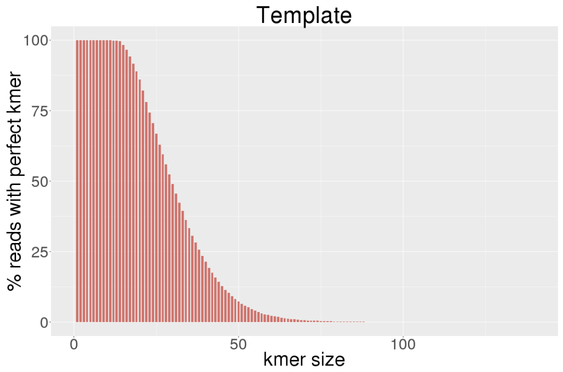

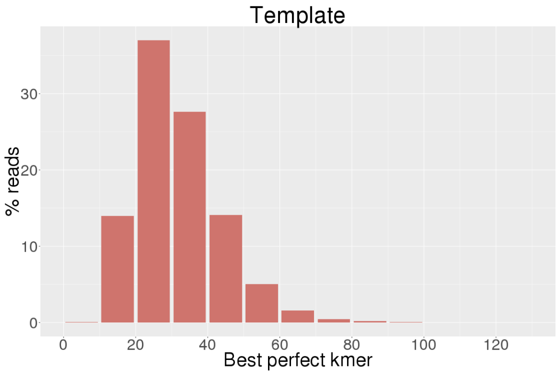

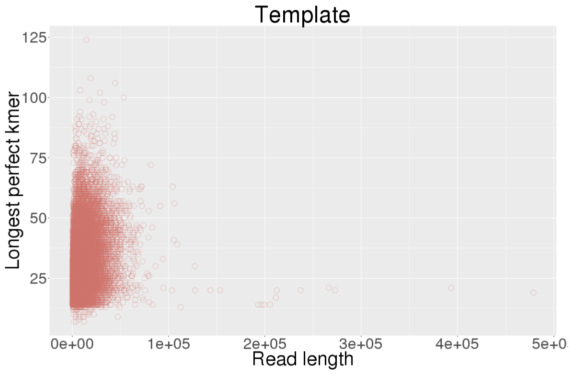

Escherichia coli coverage

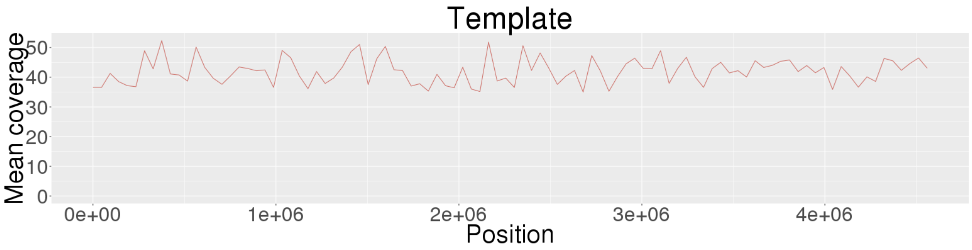

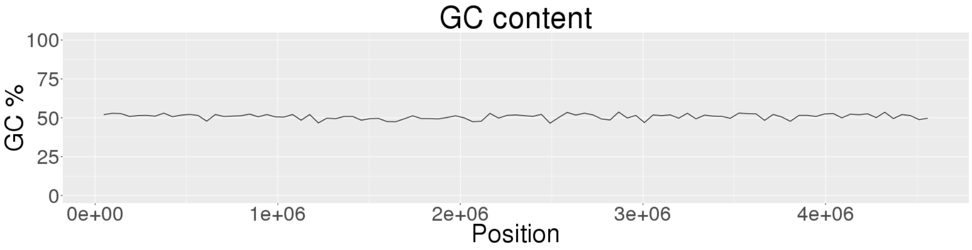

Escherichia coli 5-mer analysis

Under-represented 5-mers

Rank	kmer	Template Ref %	Read %	Diff %
1	TTTTT	0.251	0.022	-0.229
2	AAAAA	0.247	0.040	-0.207
3	GCCAG	0.280	0.140	-0.139
4	ATTTT	0.194	0.055	-0.139
5	GTTTT	0.186	0.056	-0.130
6	ATAAA	0.168	0.039	-0.130
7	TTTAT	0.169	0.039	-0.129
8	TTTTC	0.198	0.071	-0.127
9	TTATT	0.151	0.025	-0.126
10	GATAA	0.164	0.040	-0.124

Over-represented 5-mers

Rank	kmer	Template Ref %	Read %	Diff %
1	CGGCG	0.221	0.884	0.663
2	GCGGC	0.210	0.784	0.574
3	GGCGG	0.190	0.695	0.505
4	CGCCG	0.219	0.679	0.459
5	GCCGC	0.209	0.651	0.442
6	GGCCG	0.069	0.500	0.431
7	CGCGC	0.201	0.603	0.402
8	CCGGC	0.141	0.530	0.389
9	GCGCG	0.202	0.568	0.366
10	CGCGG	0.137	0.504	0.366

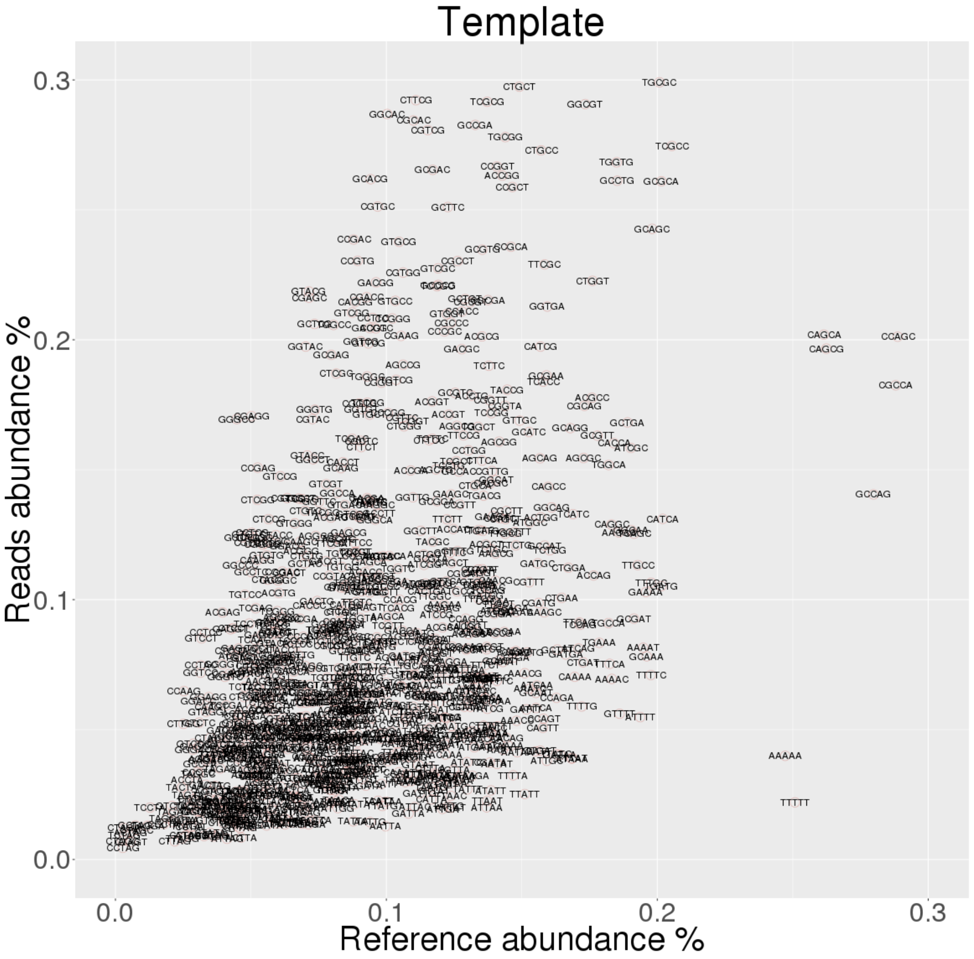

Escherichia coli GC content

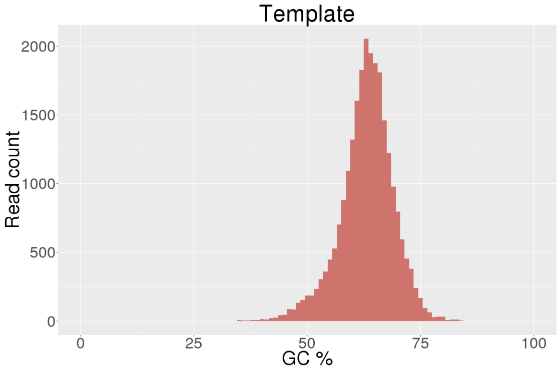

All reference 21mer analysis

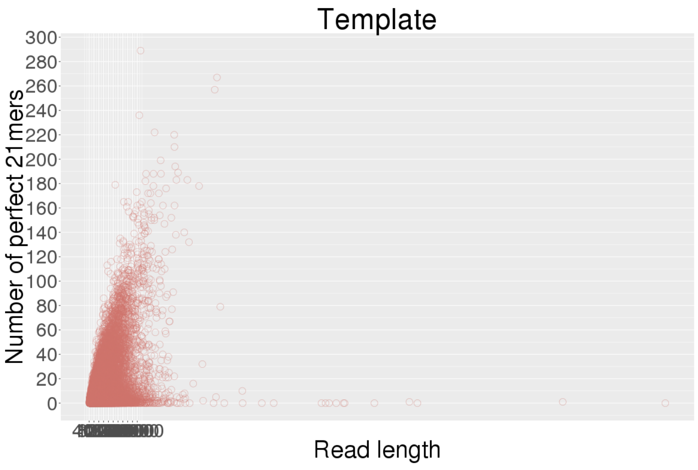

All reference substitutions

		Template substituted %			
		a	c	g	t
Reference	A	0.00	11.48	20.60	4.47
	C	2.41	0.00	4.78	6.26
	G	6.34	5.14	0.00	2.36
	T	4.48	20.50	11.19	0.00

Kmer motifs before errors

3-mer error motif analysis

	Template			
Rank	Insertion	Deletion	Substitution	
1	CGC (3.41%)	CGC (4.71%)	CGC (4.60%)	Most common
2	GCC (3.11%)	GCG (4.25%)	GCC (3.78%)	
3	TTC (2.96%)	GCC (3.76%)	GCG (3.41%)	
4	GCG (2.85%)	GGC (3.55%)	GGC (3.40%)	
5	ATC (2.60%)	TTC (3.24%)	TTC (2.95%)	
6	GGC (2.54%)	ACC (2.86%)	ACC (2.92%)	
7	ACC (2.46%)	ATC (2.77%)	ATC (2.86%)	
8	GCA (2.35%)	CCG (2.58%)	TGC (2.71%)	
9	CCA (2.33%)	CGG (2.44%)	CCG (2.45%)	
10	TTT (2.22%)	TGC (2.42%)	CGG (2.44%)	
	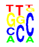	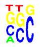	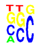	
-10	AGA (0.85%)	CAA (0.79%)	CAT (0.77%)	Least common
-9	AGG (0.80%)	TGT (0.77%)	AGA (0.74%)	
-8	GAG (0.80%)	CCT (0.70%)	ACT (0.67%)	
-7	AGT (0.75%)	AGT (0.58%)	ATA (0.59%)	
-6	TTA (0.67%)	ATA (0.55%)	TAA (0.53%)	
-5	TAT (0.60%)	TTA (0.50%)	AGT (0.52%)	
-4	GGG (0.57%)	TAT (0.45%)	TTA (0.50%)	
-3	TAA (0.52%)	TAA (0.38%)	TAT (0.44%)	
-2	CTA (0.42%)	TAG (0.29%)	TAG (0.30%)	
-1	TAG (0.27%)	CTA (0.24%)	CTA (0.22%)	
	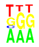	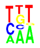	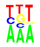	

Kmer space for 3-mers: 64 Random chance for any given 3-mer: 1.56%

4-mer error motif analysis

Rank	Template<br>Insertion	Deletion	Substitution	
1	CGCC (1.44%)	CGCC (1.79%)	CGCC (1.79%)	Most common
2	GCGC (1.12%)	GCGC (1.68%)	GCGC (1.52%)	
3	GCTG (1.04%)	GGCG (1.59%)	CGGC (1.41%)	
4	CGGC (1.01%)	CGGC (1.53%)	CCGC (1.26%)	
5	GGCG (0.95%)	CCGC (1.30%)	GGCG (1.20%)	
6	CAGC (0.95%)	CAGC (1.22%)	CAGC (1.14%)	
7	GCCA (0.94%)	CACC (1.12%)	CACC (1.12%)	
8	CTTC (0.93%)	CGCG (1.11%)	GCTG (1.11%)	
9	CACC (0.92%)	GCCG (1.06%)	CATC (1.06%)	
10	CCGC (0.91%)	CTTC (1.05%)	GCCG (0.96%)	
	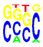	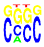	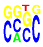	
-10	TAAG (0.08%)	ACTA (0.06%)	TATA (0.06%)	Least common
-9	CTTA (0.08%)	TCTA (0.06%)	TAAG (0.06%)	
-8	CCTA (0.07%)	CTAT (0.06%)	CTAT (0.05%)	
-7	CTAT (0.07%)	TATA (0.05%)	TAGA (0.05%)	
-6	TAGA (0.07%)	TTAG (0.05%)	CTTA (0.05%)	
-5	ATAG (0.06%)	TAGG (0.05%)	TAGT (0.05%)	
-4	TTAG (0.06%)	CTTA (0.05%)	TCTA (0.05%)	
-3	CTAA (0.05%)	CCTA (0.04%)	CTAA (0.05%)	
-2	TAGG (0.05%)	CTAA (0.04%)	CCTA (0.04%)	
-1	CTAG (0.01%)	CTAG (0.00%)	CTAG (0.00%)	
	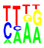	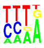	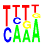	

Kmer space for 4-mers: 256 Random chance for any given 4-mer: 0.39%

5-mer error motif analysis

Rank	Template<br>Insertion	Deletion	Substitution	
1	CGCCA (0.50%)	CGGCG (0.59%)	CCGCC (0.54%)	Most common
2	CCGCC (0.42%)	GCGGC (0.58%)	GCGGC (0.53%)	
3	CCAGC (0.40%)	CCGCC (0.53%)	GCCGC (0.49%)	
4	GCGGC (0.39%)	GGCGC (0.53%)	GGCGC (0.47%)	
5	CGGCG (0.37%)	CCAGC (0.50%)	CGGCG (0.47%)	
6	CGCGC (0.36%)	GCCGC (0.48%)	CCAGC (0.47%)	
7	CAGCA (0.36%)	TGGCG (0.47%)	CGCGC (0.46%)	
8	CGCTG (0.35%)	CGCGC (0.47%)	TCGCC (0.44%)	
9	TCGCC (0.34%)	CGCCG (0.46%)	CGCCG (0.42%)	
10	GCCGC (0.34%)	TCGCC (0.43%)	GCGCC (0.42%)	
	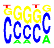	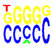	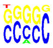	
-10	TAGGA (0.01%)	TCCTA (0.01%)	TCCTA (0.00%)	Least common
-9	GGGGG (0.01%)	CCTAA (0.01%)	TAGGA (0.00%)	
-8	CTAGC (0.00%)	CTAGC (0.00%)	CTAGC (0.00%)	
-7	GCTAG (0.00%)	ACTAG (0.00%)	TCTAG (0.00%)	
-6	CTAGT (0.00%)	CTAGT (0.00%)	ACTAG (0.00%)	
-5	TCTAG (0.00%)	GCTAG (0.00%)	CTAGT (0.00%)	
-4	ACTAG (0.00%)	TCTAG (0.00%)	GCTAG (0.00%)	
-3	CTAGA (0.00%)	CTAGA (0.00%)	CCTAG (0.00%)	
-2	CCTAG (0.00%)	CTAGG (0.00%)	CTAGA (0.00%)	
-1	CTAGG (0.00%)	CCTAG (0.00%)	CTAGG (0.00%)	
	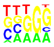	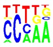	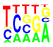	

Kmer space for 5-mers: 1024 Random chance for any given 5-mer: 0.10%
