# Supplementary material for: MinION Analysis and Reference Consortium: Phase 2 data release and analysis of R9.0 chemistry
Source: F1000Res. 2017 May 31;6:760. [Version 1] doi: 10.12688/f1000research.11354.1 (PMC5538040; doi:10.12688/f1000research.11354.1)
Supplement: Supplementary file 3 [file f1000research-6-12257-s0002.tgz › 5d2560fe-dd7a-44d1-b265-e3522e742149.zip!/SI File 3. NanoOK experiment reports/P2-Lab6-R1-1D_nanook_bwa_passfail.pdf]

NanoOK report for UBC_MARC_1D_R9_107_Called

Pass and fail counts

Type	Pass	Fail
Template	56904	39259

Read lengths

Type	NumReads	TotalBases	Mean	Longest	Shortest	N50	N50Count	N90	N90Count
Template	96163	829653092	8627.57	478773	5	15694	15662	4379	53723

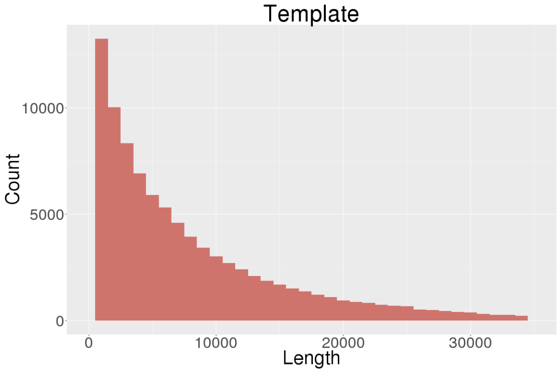

Template alignments

Number of reads	96163	
Number of reads with alignments	81408	(84.66%)
Number of reads without alignments	14755	(15.34%)

ID	Size	Number of Reads	% of Reads	Mean read length	Aligned bases	Mean coverage	Longest Perf Kmer
Control sequence	3560	0	0.00	0.00	0	0.00	0
Escherichia coli	4641652	81408	84.66	9492.46	742068606	159.87	235

Escherichia coli error analysis

	Template
Overall base identity (excluding indels)	74.80%
Aligned base identity (excluding indels)	88.22%
Identical bases per 100 aligned bases (including indels)	77.90%
Inserted bases per 100 aligned bases (including indels)	3.51%
Deleted bases per 100 aligned bases (including indels)	8.19%
Substitutions per 100 aligned bases (including indels)	10.40%
Mean insertion size	1.57
Mean deletion size	1.69

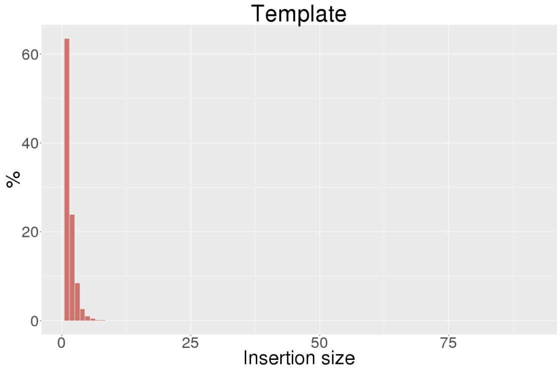

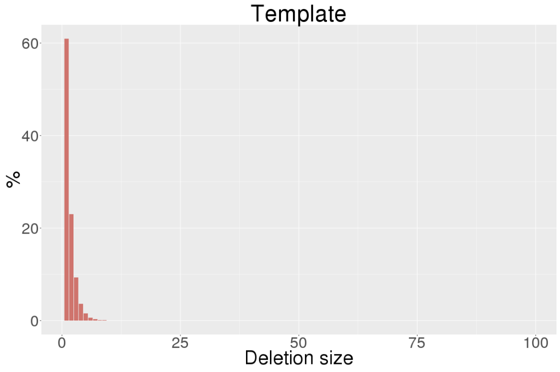

Escherichia coli read identity

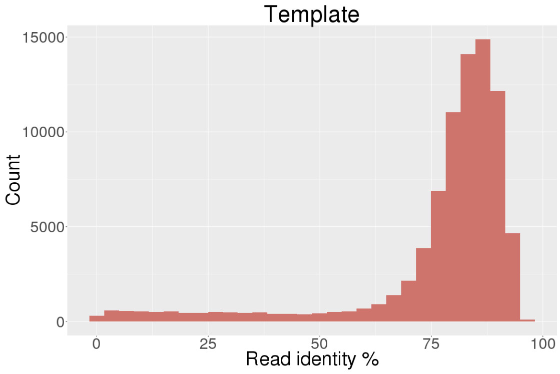

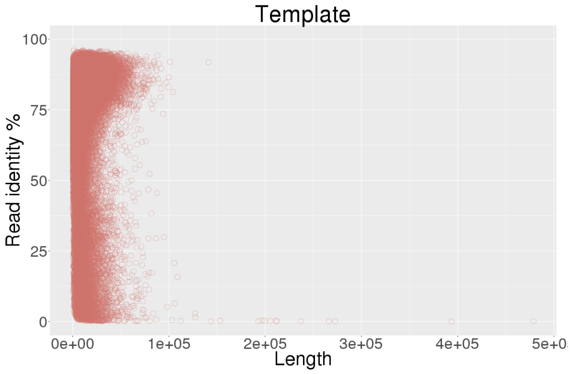

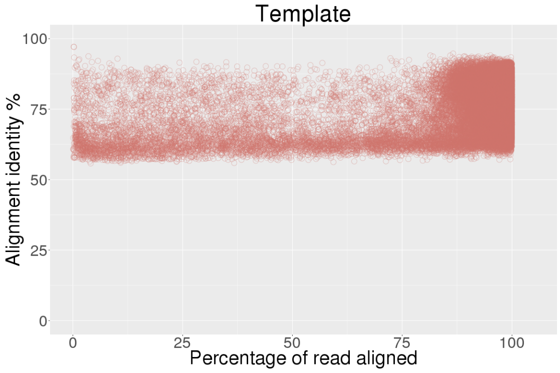

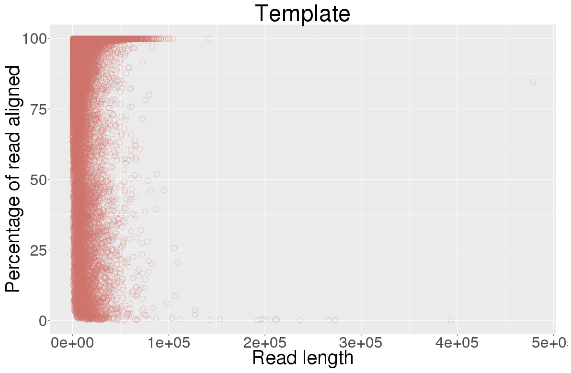

Escherichia coli perfect kmers

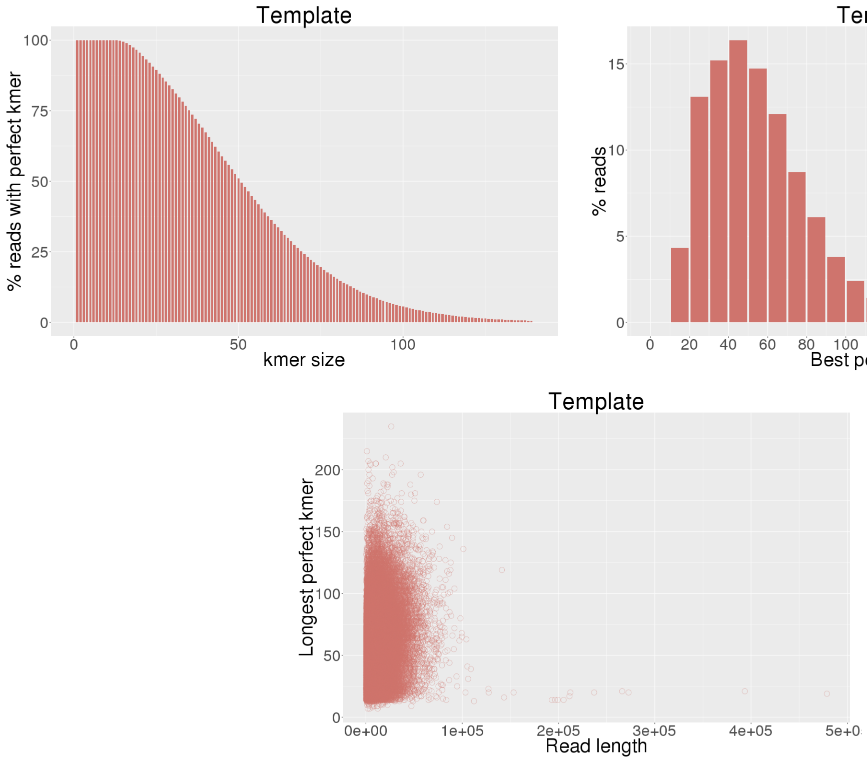

Escherichia coli coverage

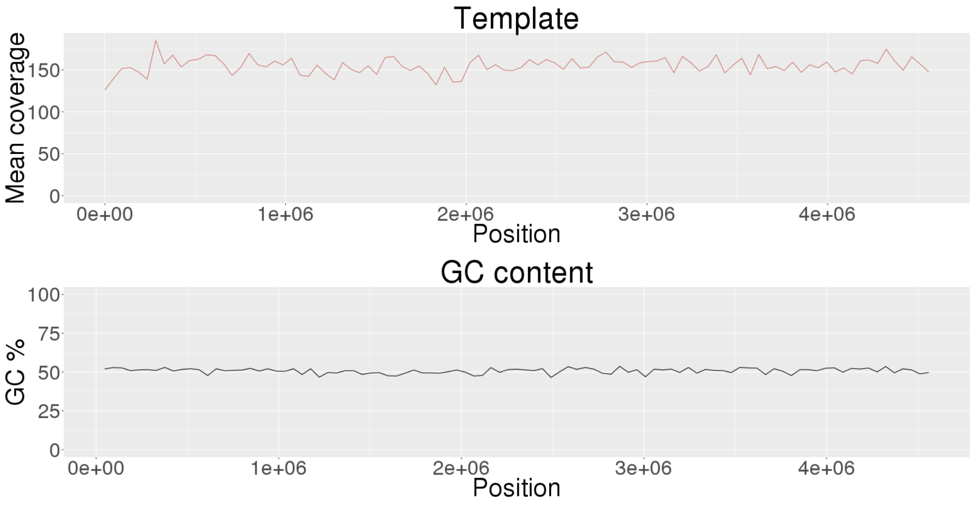

Escherichia coli 5-mer analysis

Under-represented 5-mers

Rank	kmer	Template Ref %	Template Read %	Template Diff %
1	TTTTT	0.251	0.037	-0.214
2	AAAAA	0.247	0.083	-0.164
3	GCCAG	0.280	0.173	-0.107
4	GTTTT	0.186	0.088	-0.098
5	ATTTT	0.194	0.109	-0.084
6	TTATT	0.151	0.071	-0.080
7	TTTTA	0.147	0.068	-0.079
8	TTTTG	0.172	0.095	-0.077
9	ATTGC	0.159	0.083	-0.076
10	TTAAT	0.137	0.063	-0.074

Over-represented 5-mers

Rank	kmer	Template Ref %	Template Read %	Template Diff %
1	CGGCG	0.221	0.542	0.321
2	GCGGC	0.210	0.482	0.272
3	GGCGG	0.190	0.427	0.237
4	CGCCG	0.219	0.425	0.206
5	CGCGC	0.201	0.403	0.201
6	GCCGC	0.209	0.404	0.195
7	GGCCG	0.069	0.260	0.191
8	GCGCG	0.202	0.382	0.180
9	CCGGC	0.141	0.315	0.174
10	CGCGG	0.137	0.308	0.171

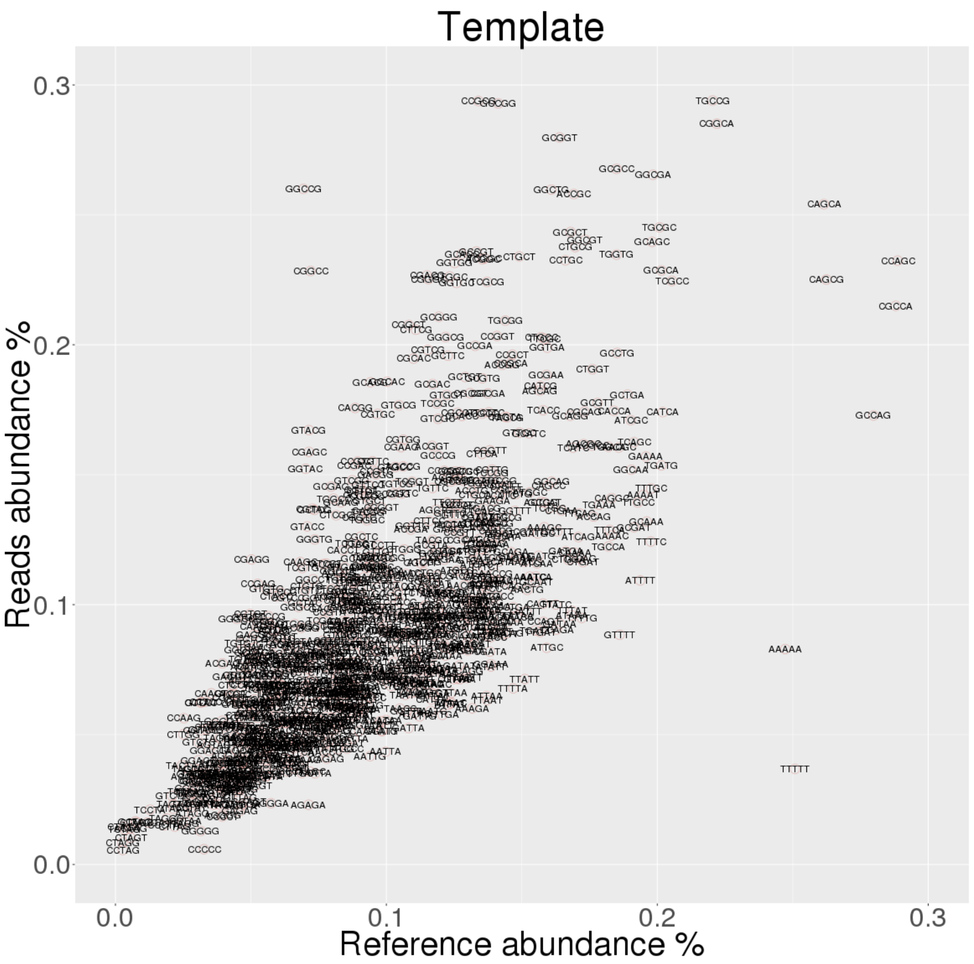

Escherichia coli GC content

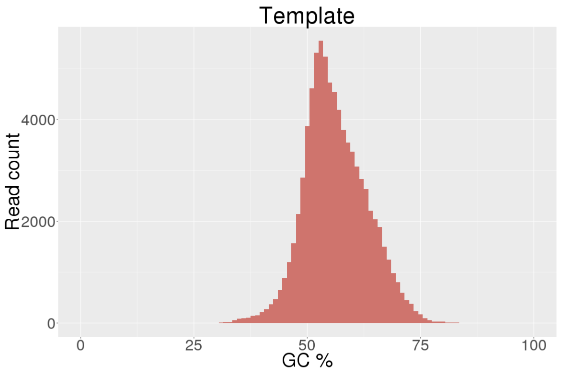

All reference 21mer analysis

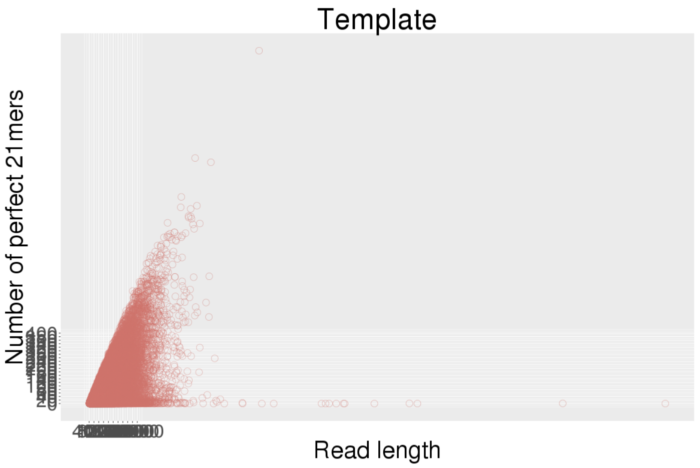

All reference substitutions

		Template substituted %			
		a	c	g	t
Reference	A	0.00	9.54	18.88	4.58
	C	3.03	0.00	4.25	9.47
	G	9.42	4.56	0.00	2.99
	T	4.58	19.19	9.49	0.00

Kmer motifs before errors

3-mer error motif analysis

		Template		
Rank	Insertion	Deletion	Substitution	
1	TTC (3.18%)	CGC (3.79%)	CGC (4.22%)	Most common
2	CGC (3.13%)	GCG (3.20%)	GCC (3.65%)	
3	ATC (2.77%)	GCC (3.17%)	GGC (3.11%)	
4	GCC (2.77%)	TTC (3.09%)	ATC (3.08%)	
5	TTT (2.55%)	GGC (2.99%)	ACC (3.06%)	
6	GCG (2.47%)	ATC (2.82%)	TGC (3.01%)	
7	ACC (2.36%)	ACC (2.62%)	TTC (2.99%)	
8	TGC (2.33%)	TTT (2.46%)	GCG (2.65%)	
9	GGC (2.29%)	TGC (2.30%)	AAC (2.62%)	
10	CTG (2.11%)	AGC (2.22%)	AGC (2.47%)	
	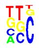	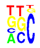	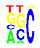	
-10	TTA (0.97%)	GTA (0.86%)	CCT (0.83%)	Least common
-9	GGA (0.88%)	TTA (0.85%)	ACT (0.79%)	
-8	AGT (0.86%)	TAT (0.84%)	TTA (0.78%)	
-7	GAG (0.85%)	AGG (0.82%)	TAT (0.74%)	
-6	AGA (0.84%)	AGT (0.75%)	GGA (0.70%)	
-5	TAA (0.79%)	GGG (0.70%)	GGG (0.70%)	
-4	AGG (0.75%)	TAA (0.69%)	AGA (0.69%)	
-3	GGG (0.52%)	CCT (0.68%)	AGT (0.57%)	
-2	CTA (0.48%)	TAG (0.36%)	TAG (0.37%)	
-1	TAG (0.38%)	CTA (0.33%)	CTA (0.27%)	
	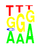	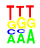	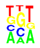	

Kmer space for 3-mers: 64 Random chance for any given 3-mer: 1.56%

4-mer error motif analysis

Rank	Insertion	Template Deletion	Substitution	
1	CGCC (1.21%)	CGCC (1.40%)	CGCC (1.62%)	Most common
2	TTTC (1.05%)	GCGC (1.32%)	GCGC (1.36%)	
3	GCGC (1.04%)	CGGC (1.19%)	CGGC (1.20%)	
4	CAGC (0.92%)	GGCG (1.12%)	CAGC (1.18%)	
5	GCTG (0.90%)	CAGC (1.10%)	CATC (1.05%)	
6	CATC (0.87%)	TTTC (0.99%)	CCGC (1.05%)	
7	CTTC (0.86%)	CATC (0.96%)	CACC (1.05%)	
8	CGGC (0.86%)	CCGC (0.95%)	ACGC (0.97%)	
9	GCCA (0.81%)	CACC (0.93%)	TGGC (0.93%)	
10	CACC (0.81%)	CTTC (0.86%)	GCTG (0.91%)	
	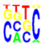	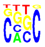	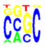	
-10	CTTA (0.11%)	TAGA (0.10%)	CTTA (0.09%)	Least common
-9	TCTA (0.11%)	CTTA (0.09%)	ACTA (0.08%)	
-8	AGGG (0.10%)	CCCT (0.09%)	GAGT (0.08%)	
-7	TTAG (0.09%)	ACTA (0.09%)	CTAA (0.08%)	
-6	TAGA (0.08%)	TCTA (0.08%)	TAGT (0.08%)	
-5	CTAA (0.07%)	TTAG (0.08%)	TAGG (0.07%)	
-4	CCTA (0.07%)	CTAA (0.07%)	TCTA (0.06%)	
-3	GGGG (0.07%)	TAGG (0.06%)	TAGA (0.06%)	
-2	TAGG (0.06%)	CCTA (0.05%)	CCTA (0.04%)	
-1	CTAG (0.01%)	CTAG (0.01%)	CTAG (0.01%)	
	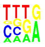	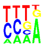	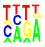	

Kmer space for 4-mers: 256 Random chance for any given 4-mer: 0.39%

5-mer error motif analysis

Rank	Insertion	Template Deletion	Substitution	
1	CGCCA (0.40%)	GCGGC (0.43%)	CCAGC (0.46%)	Most common
2	CCAGC (0.36%)	CCAGC (0.41%)	CCGCC (0.46%)	
3	CGCGC (0.33%)	GGCGC (0.40%)	GCGGC (0.44%)	
4	CCGCC (0.33%)	CCGCC (0.39%)	TCGCC (0.42%)	
5	ATTTC (0.32%)	CGGCG (0.38%)	CTGGC (0.40%)	
6	GCGGC (0.32%)	TGGCG (0.37%)	GCCGC (0.40%)	
7	TCGCC (0.31%)	TCGCC (0.37%)	CGCGC (0.39%)	
8	CAGCA (0.31%)	CGCGC (0.36%)	GCGCC (0.39%)	
9	GTTTC (0.29%)	GCCGC (0.34%)	GGCGC (0.38%)	
10	CGCTG (0.29%)	CTGGC (0.33%)	ACGCC (0.35%)	
	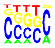	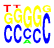	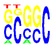	
-10	TAGGA (0.01%)	TCCTA (0.01%)	TCCTA (0.01%)	Least common
-9	CTAGC (0.01%)	TAGGA (0.01%)	CTAGC (0.01%)	
-8	GGGGG (0.00%)	CTAGC (0.00%)	TAGGA (0.01%)	
-7	ACTAG (0.00%)	CTAGT (0.00%)	ACTAG (0.00%)	
-6	GCTAG (0.00%)	ACTAG (0.00%)	CTAGT (0.00%)	
-5	CTAGT (0.00%)	GCTAG (0.00%)	GCTAG (0.00%)	
-4	TCTAG (0.00%)	TCTAG (0.00%)	TCTAG (0.00%)	
-3	CTAGA (0.00%)	CTAGA (0.00%)	CTAGG (0.00%)	
-2	CCTAG (0.00%)	CTAGG (0.00%)	CCTAG (0.00%)	
-1	CTAGG (0.00%)	CCTAG (0.00%)	CTAGA (0.00%)	
	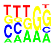	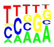	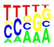	

Kmer space for 5-mers: 1024 Random chance for any given 5-mer: 0.10%
