# Supplementary material for: MinION Analysis and Reference Consortium: Phase 2 data release and analysis of R9.0 chemistry
Source: F1000Res. 2017 May 31;6:760. [Version 1] doi: 10.12688/f1000research.11354.1 (PMC5538040; doi:10.12688/f1000research.11354.1)
Supplement: Supplementary file 3 [file f1000research-6-12257-s0002.tgz › 5d2560fe-dd7a-44d1-b265-e3522e742149.zip!/SI File 3. NanoOK experiment reports/P2-Lab6-R1-1D_nanook_bwa_passonly.pdf]

NanoOK report for UBC_MARC_1D_R9_107_Called

Pass and fail counts

Type	Pass	Fail
Template	56904	0

Read lengths

Type	NumReads	TotalBases	Mean	Longest	Shortest	N50	N50Count	N90	N90Count
Template	56904	526340999	9249.63	141200	13	16214	9728	4560	32983

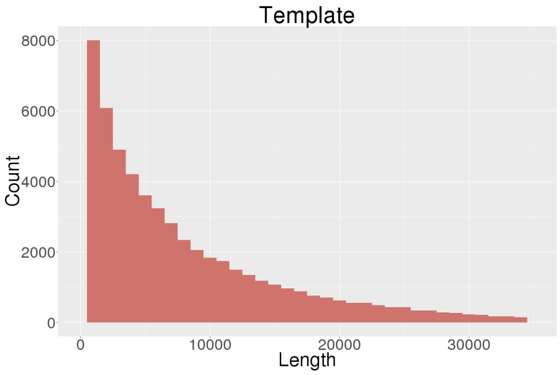

Template alignments

Number of reads	56904	
Number of reads with alignments	56830	(99.87%)
Number of reads without alignments	74	(0.13%)

ID	Size	Number of Reads	% of Reads	Mean read length	Aligned bases	Mean coverage	Longest Perf Kmer
Control sequence	3560	0	0.00	0.00	0	0.00	0
Escherichia coli	4641652	56830	99.87	9234.13	539931271	116.32	235

Escherichia coli error analysis

	Template
Overall base identity (excluding indels)	83.81%
Aligned base identity (excluding indels)	90.77%
Identical bases per 100 aligned bases (including indels)	81.46%
Inserted bases per 100 aligned bases (including indels)	3.22%
Deleted bases per 100 aligned bases (including indels)	7.04%
Substitutions per 100 aligned bases (including indels)	8.28%
Mean insertion size	1.59
Mean deletion size	1.63

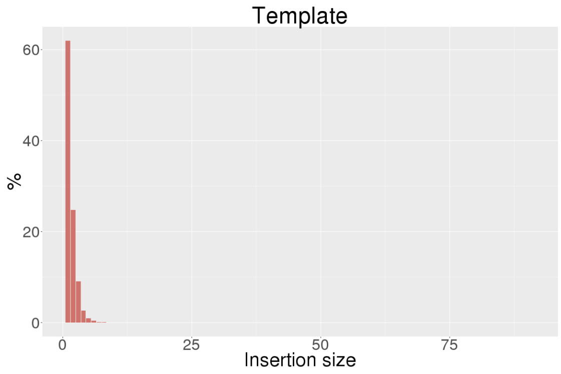

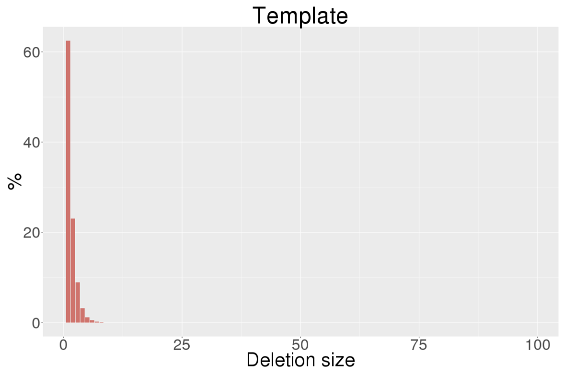

Escherichia coli read identity

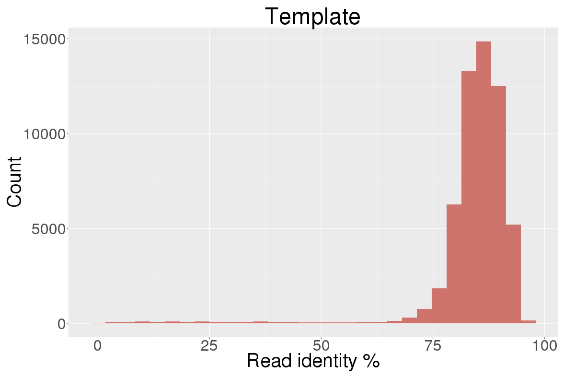

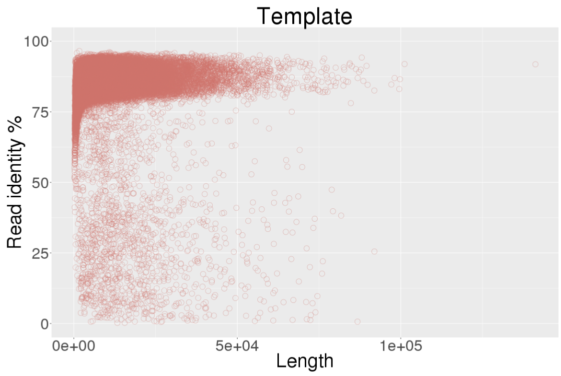

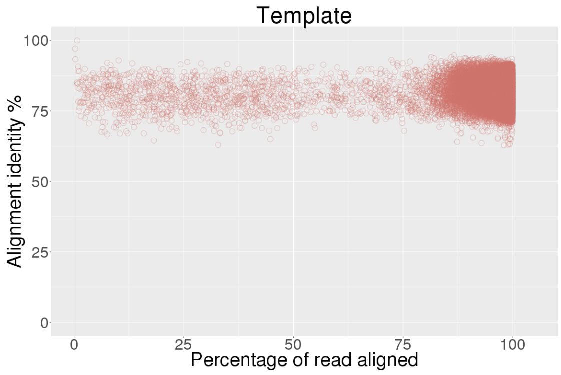

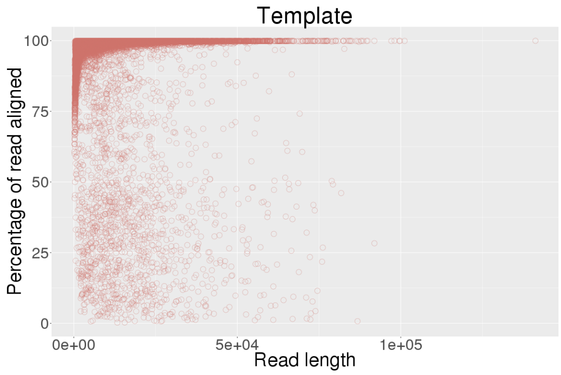

Escherichia coli perfect kmers

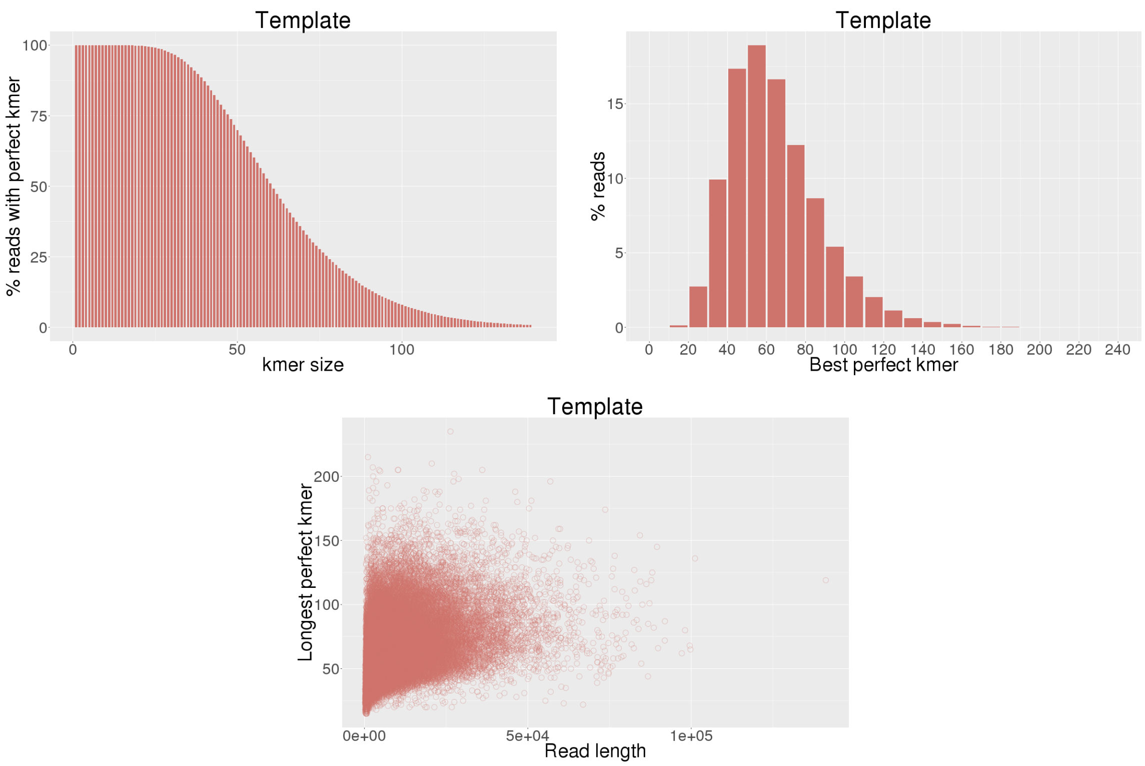

Escherichia coli coverage

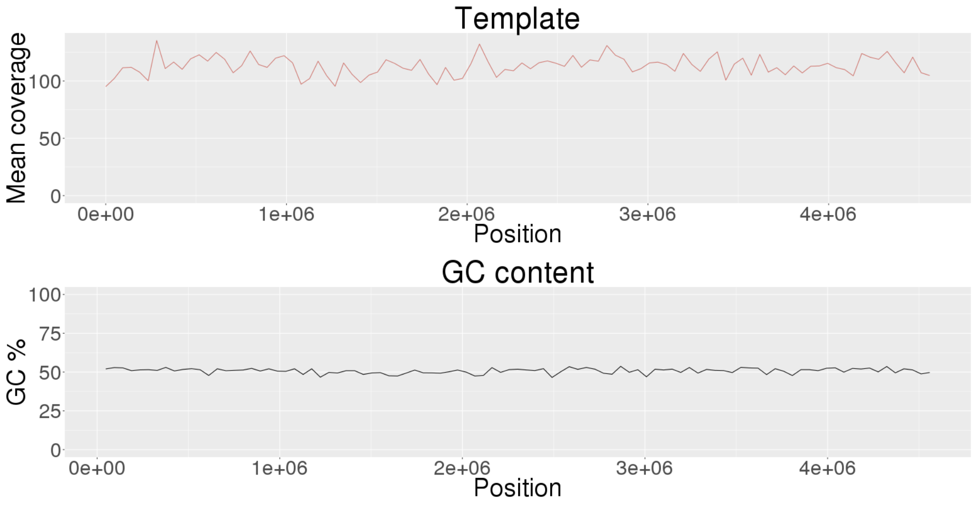

Escherichia coli 5-mer analysis

Under-represented 5-mers

Rank	kmer	Template<br>Ref %	Read %	Diff %
1	TTTTT	0.251	0.044	-0.207
2	AAAAA	0.247	0.103	-0.144
3	GCCAG	0.280	0.188	-0.092
4	GTTTT	0.186	0.104	-0.082
5	CCTGG	0.130	0.060	-0.070
6	CCAGG	0.129	0.066	-0.063
7	TTTTA	0.147	0.084	-0.062
8	TTTTG	0.172	0.112	-0.060
9	CCAGA	0.163	0.104	-0.059
10	ATTTT	0.194	0.135	-0.059

Over-represented 5-mers

Rank	kmer	Template<br>Ref %	Read %	Diff %
1	CGGCG	0.221	0.381	0.160
2	GCGGC	0.210	0.339	0.130
3	GGCGG	0.190	0.300	0.111
4	CGCGC	0.201	0.308	0.107
5	CGGTG	0.160	0.263	0.103
6	GCGCG	0.202	0.294	0.092
7	CGCCG	0.219	0.305	0.086
8	CGCGG	0.137	0.216	0.079
9	GCCGC	0.209	0.287	0.078
10	GGCCG	0.069	0.146	0.077

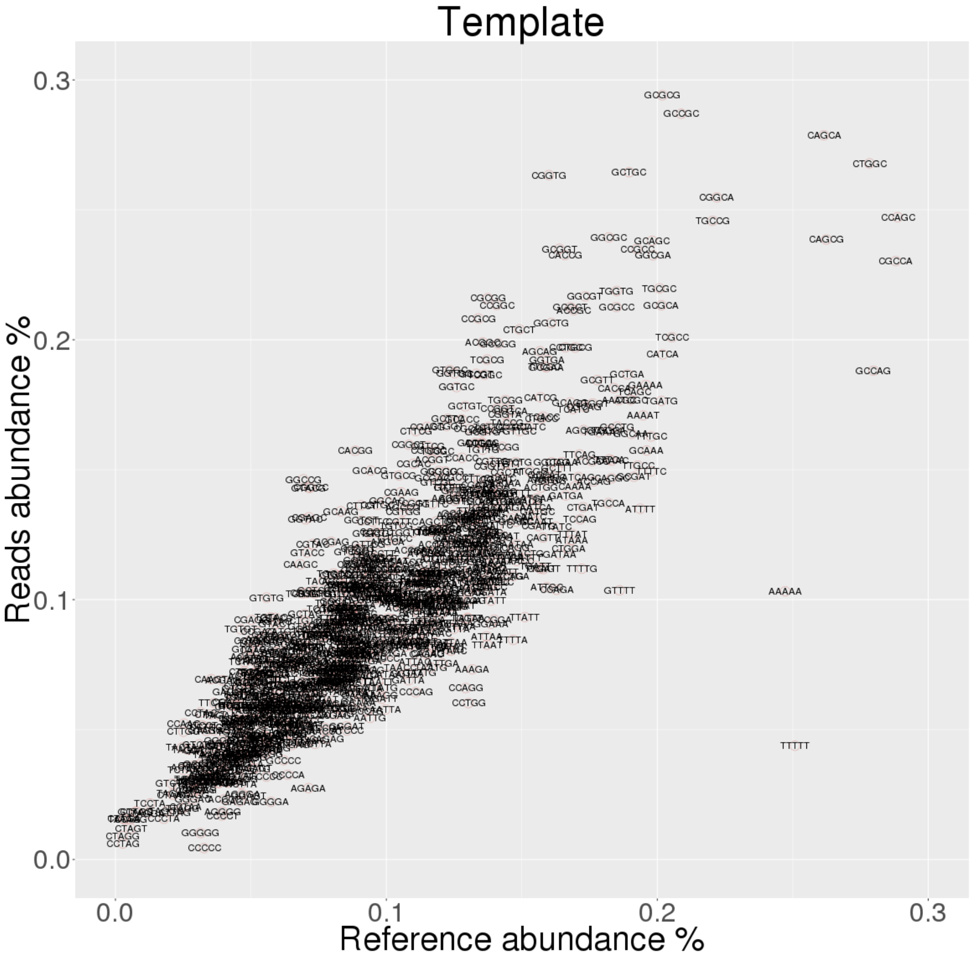

Escherichia coli GC content

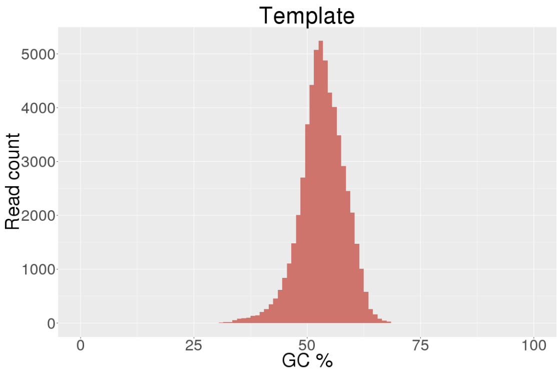

All reference 21mer analysis

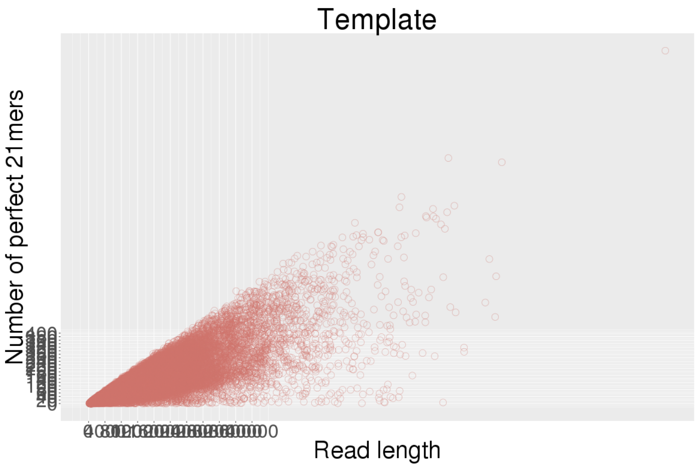

All reference substitutions

		Template substituted %			
		a	c	g	t
Reference	A	0.00	8.14	17.64	4.66
	C	3.48	0.00	3.87	11.82
	G	11.66	4.14	0.00	3.45
	T	4.66	18.24	8.24	0.00

Kmer motifs before errors

3-mer error motif analysis

		Template		
Rank	Insertion	Deletion	Substitution	
1	TTC (3.27%)	CGC (3.44%)	CGC (4.04%)	Most common
2	CGC (3.02%)	TTC (3.03%)	GCC (3.59%)	
3	ATC (2.84%)	GCC (2.95%)	ATC (3.18%)	
4	TTT (2.67%)	ATC (2.84%)	TGC (3.14%)	
5	GCC (2.64%)	GCG (2.81%)	ACC (3.13%)	
6	TGC (2.37%)	GGC (2.77%)	TTC (3.01%)	
7	GCG (2.33%)	TTT (2.72%)	GGC (2.99%)	
8	ACC (2.32%)	ACC (2.53%)	AAC (2.78%)	
9	GGC (2.19%)	TGC (2.25%)	AGC (2.55%)	
10	CTG (2.07%)	AGC (2.17%)	TTT (2.48%)	
	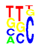	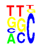	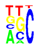	
-10	TGG (1.07%)	TTA (0.98%)	GTA (0.87%)	Least common
-9	AGT (0.91%)	GTA (0.88%)	ACT (0.84%)	
-8	TAA (0.89%)	GGA (0.86%)	CCT (0.83%)	
-7	GAG (0.87%)	AGT (0.82%)	GAG (0.82%)	
-6	AGA (0.84%)	TAA (0.81%)	AGA (0.66%)	
-5	GGA (0.81%)	AGG (0.77%)	GGG (0.65%)	
-4	AGG (0.74%)	CCT (0.67%)	GGA (0.60%)	
-3	CTA (0.50%)	GGG (0.63%)	AGT (0.59%)	
-2	GGG (0.50%)	TAG (0.39%)	TAG (0.40%)	
-1	TAG (0.43%)	CTA (0.37%)	CTA (0.30%)	
	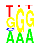	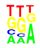	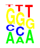	

Kmer space for 3-mers: 64   Random chance for any given 3-mer: 1.56%

4-mer error motif analysis

Rank	Insertion	Template Deletion	Substitution	
1	CGCC (1.13%)	CGCC (1.28%)	CGCC (1.56%)	Most common
2	TTTC (1.12%)	GCGC (1.20%)	GCGC (1.30%)	
3	GCGC (1.01%)	CGGC (1.07%)	CAGC (1.20%)	
4	CAGC (0.92%)	CAGC (1.06%)	CGGC (1.12%)	
5	CATC (0.87%)	TTTC (1.00%)	CATC (1.04%)	
6	GCTG (0.85%)	GGCG (0.97%)	CACC (1.02%)	
7	CTTC (0.84%)	CATC (0.93%)	ACGC (0.97%)	
8	CGGC (0.81%)	CACC (0.87%)	CCGC (0.96%)	
9	CACC (0.78%)	CCGC (0.83%)	TGGC (0.94%)	
10	GCCA (0.77%)	TGGC (0.83%)	TGCC (0.91%)	
	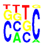	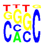	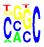	
-10	GGGA (0.11%)	CTTA (0.11%)	GGGG (0.09%)	Least common
-9	TCTA (0.11%)	ACTA (0.10%)	CTAA (0.09%)	
-8	TTAG (0.10%)	GGGG (0.09%)	TAGT (0.08%)	
-7	AGGG (0.09%)	TTAG (0.09%)	ACTA (0.08%)	
-6	CTAA (0.08%)	TCTA (0.09%)	GAGT (0.08%)	
-5	TAGA (0.08%)	CCCT (0.09%)	TAGG (0.08%)	
-4	CCTA (0.07%)	CTAA (0.08%)	TCTA (0.06%)	
-3	GGGG (0.07%)	TAGG (0.06%)	TAGA (0.06%)	
-2	TAGG (0.06%)	CCTA (0.05%)	CCTA (0.04%)	
-1	CTAG (0.01%)	CTAG (0.01%)	CTAG (0.01%)	
	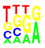	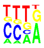	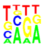	

Kmer space for 4-mers: 256 Random chance for any given 4-mer: 0.39%

5-mer error motif analysis

Rank	Insertion	Template Deletion	Substitution	
1	CGCCA (0.37%)	GCGGC (0.39%)	CCAGC (0.46%)	Most common
2	CCAGC (0.35%)	CCAGC (0.38%)	CCGCC (0.43%)	
3	ATTTC (0.34%)	GGCGC (0.36%)	TCGCC (0.41%)	
4	CGCGC (0.32%)	TCGCC (0.35%)	GCGGC (0.41%)	
5	GTTTC (0.31%)	CCGCC (0.34%)	CTGGC (0.40%)	
6	CCGCC (0.30%)	TGGCG (0.34%)	GCGCC (0.38%)	
7	TCGCC (0.30%)	CGCGC (0.32%)	CGCGC (0.37%)	
8	GCGGC (0.29%)	CTGGC (0.32%)	GCCGC (0.37%)	
9	CAGCA (0.29%)	CGGCG (0.31%)	ACGCC (0.35%)	
10	TGCTG (0.28%)	TGCGC (0.30%)	GGCGC (0.35%)	
	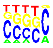	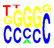	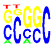	
-10	CTAGC (0.01%)	CCTGG (0.01%)	CTAGC (0.01%)	Least common
-9	CCTGG (0.01%)	CCAGG (0.01%)	GGGGG (0.01%)	
-8	ACTAG (0.00%)	CTAGC (0.00%)	TAGGA (0.01%)	
-7	GCTAG (0.00%)	CTAGT (0.00%)	ACTAG (0.00%)	
-6	GGGGG (0.00%)	ACTAG (0.00%)	CTAGT (0.00%)	
-5	CTAGT (0.00%)	GCTAG (0.00%)	GCTAG (0.00%)	
-4	TCTAG (0.00%)	TCTAG (0.00%)	TCTAG (0.00%)	
-3	CTAGA (0.00%)	CTAGA (0.00%)	CTAGG (0.00%)	
-2	CCTAG (0.00%)	CTAGG (0.00%)	CCTAG (0.00%)	
-1	CTAGG (0.00%)	CCTAG (0.00%)	CTAGA (0.00%)	
	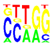	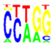	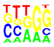	

Kmer space for 5-mers: 1024 Random chance for any given 5-mer: 0.10%
