# Supplementary material for: MinION Analysis and Reference Consortium: Phase 2 data release and analysis of R9.0 chemistry
Source: F1000Res. 2017 May 31;6:760. [Version 1] doi: 10.12688/f1000research.11354.1 (PMC5538040; doi:10.12688/f1000research.11354.1)
Supplement: Supplementary file 3 [file f1000research-6-12257-s0002.tgz › 5d2560fe-dd7a-44d1-b265-e3522e742149.zip!/SI File 3. NanoOK experiment reports/P2-Lab6-R1-2D_nanook_bwa_failonly.pdf]

NanoOK report for UBC_MARC_2D_R9_107_Called

Pass and fail counts

Type	Pass	Fail
Template	0	84957
Complement	0	47422
2D	0	21885

Read lengths

Type	NumReads	TotalBases	Mean	Longest	Shortest	N50	N50Count	N90	N90Count
Template	84957	460038194	5414.95	119036	5	8605	16794	3209	54642
Complement	47422	151429691	3193.24	403748	5	5280	8075	2172	26505
2D	21885	94109976	4300.20	34218	98	4410	5818	2856	16826

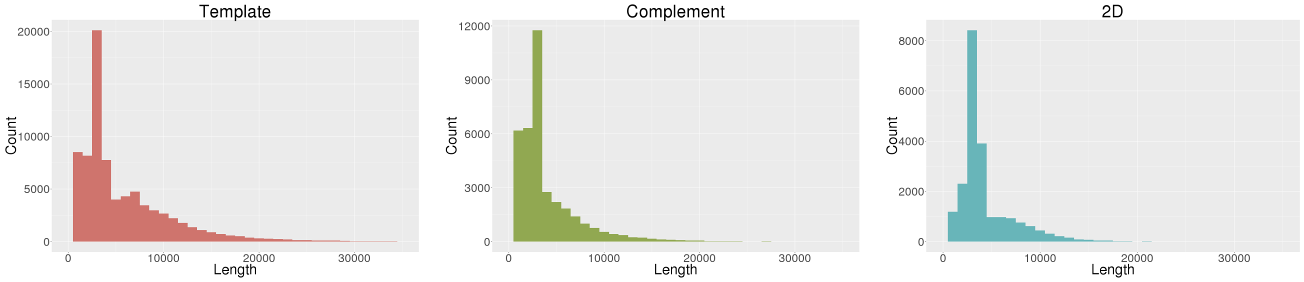

Template alignments

Number of reads	84957	
Number of reads with alignments	61718	(72.65%)
Number of reads without alignments	23239	(27.35%)

ID	Size	Number of Reads	% of Reads	Mean read length	Aligned bases	Mean coverage	Longest Perf Kmer
Control sequence	3560	23527	27.69	3528.09	75858727	21308.63	217
Escherichia coli	4641652	38191	44.95	8367.61	262952330	56.65	275

Complement alignments

Number of reads	47422	
Number of reads with alignments	29159	(61.49%)
Number of reads without alignments	18263	(38.51%)

ID	Size	Number of Reads	% of Reads	Mean read length	Aligned bases	Mean coverage	Longest Perf Kmer
Control sequence	3560	12670	26.72	2702.54	33833561	9503.81	169
Escherichia coli	4641652	16489	34.77	5696.12	61219855	13.19	161

2D alignments

Number of reads	21885	
Number of reads with alignments	18320	(83.71%)
Number of reads without alignments	3565	(16.29%)

ID	Size	Number of Reads	% of Reads	Mean read length	Aligned bases	Mean coverage	Longest Perf Kmer
Control sequence	3560	11050	50.49	3229.66	36789483	10334.12	696
Escherichia coli	4641652	7270	33.22	6514.23	43787384	9.43	263

Control sequence error analysis

	Template	Complement	2D
Overall base identity (excluding indels)	74.49%	75.66%	89.62%
Aligned base identity (excluding indels)	90.91%	90.62%	94.75%
Identical bases per 100 aligned bases (including indels)	81.50%	76.57%	86.93%
Inserted bases per 100 aligned bases (including indels)	2.73%	2.10%	2.69%
Deleted bases per 100 aligned bases (including indels)	7.61%	13.40%	5.56%
Substitutions per 100 aligned bases (including indels)	8.15%	7.93%	4.81%
Mean insertion size	1.52	1.41	1.68
Mean deletion size	1.70	2.28	1.84

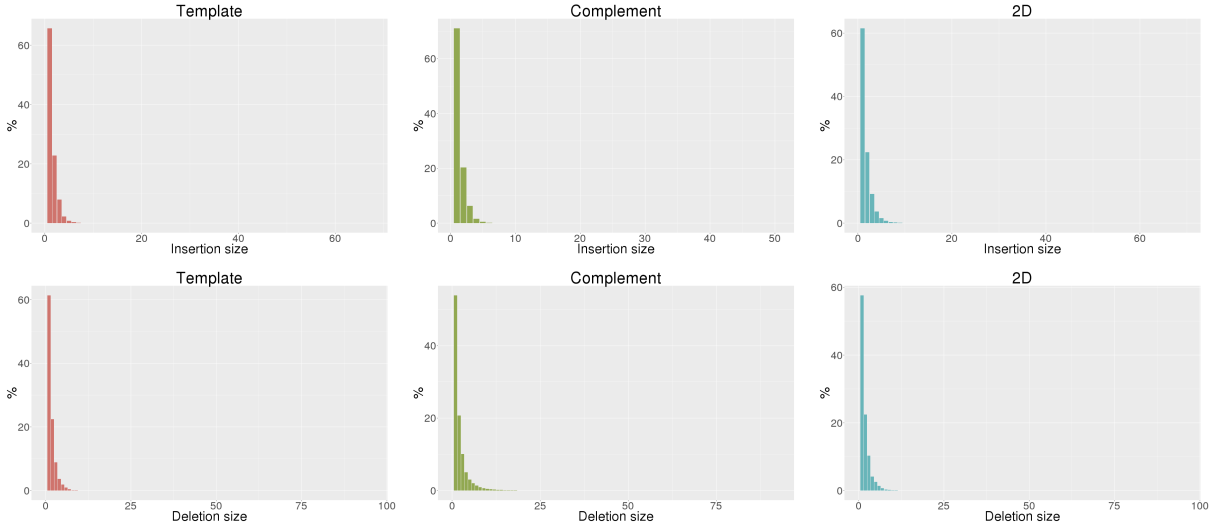

Control sequence read identity

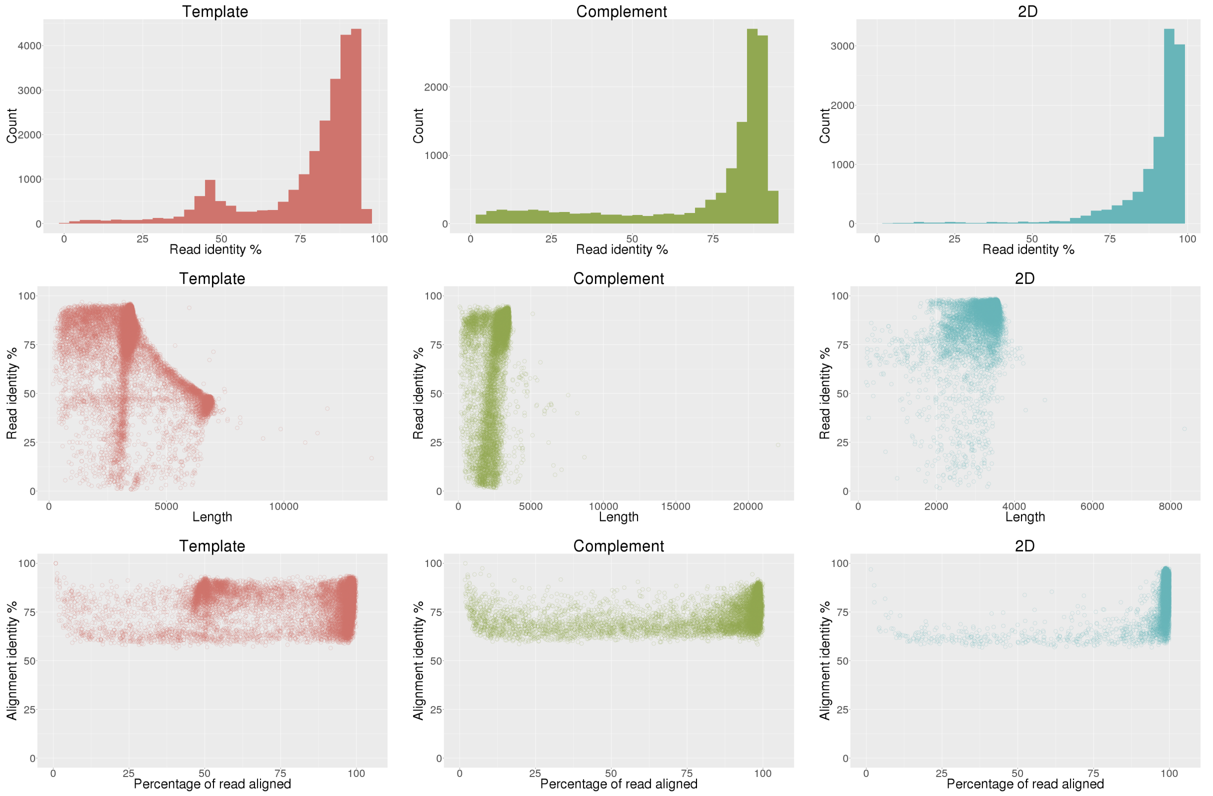

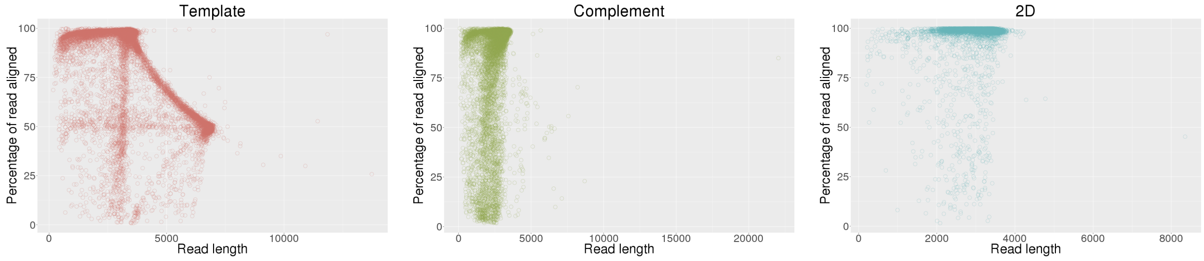

Control sequence perfect kmers

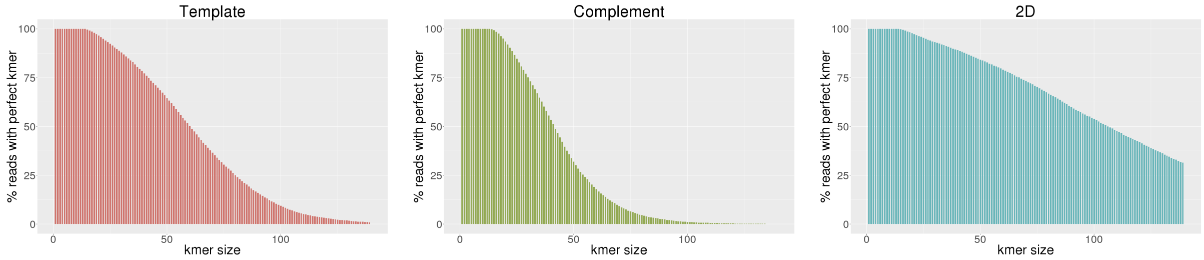

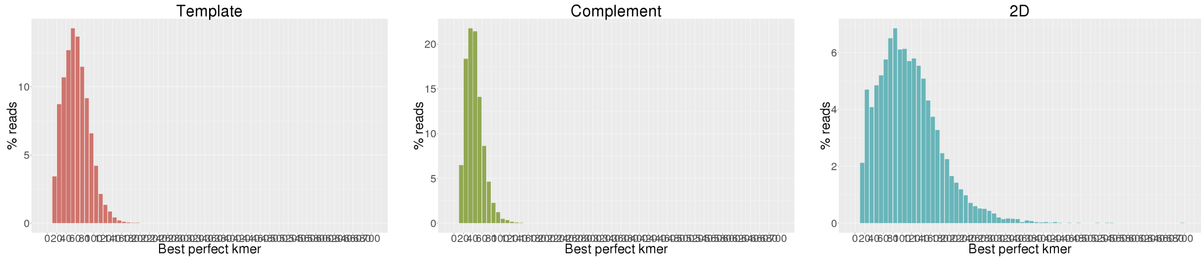

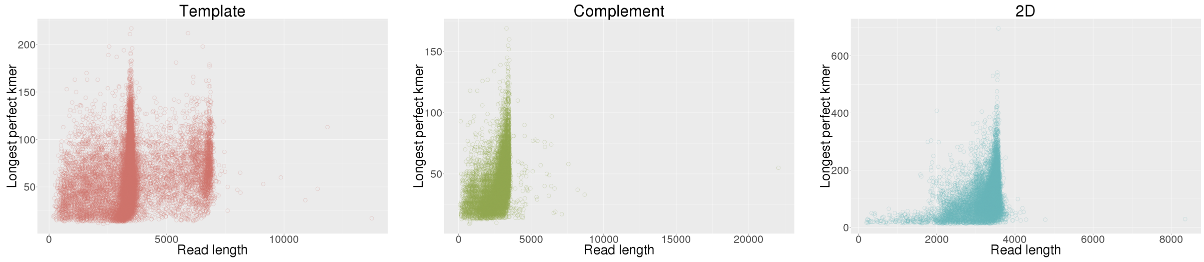

Control sequence coverage

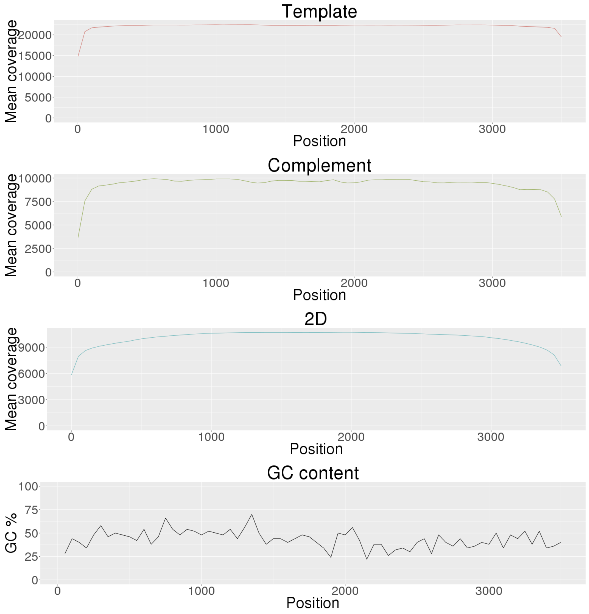

Control sequence 5-mer analysis

Under-represented 5-mers

	Template				Complement				2D			
Rank	kmer	Ref %	Read %	Diff %	kmer	Ref %	Read %	Diff %	kmer	Ref %	Read %	Diff %
1	TTTTT	0.759	0.099	-0.660	TTTTT	0.759	0.135	-0.624	TTTTT	0.759	0.205	-0.554
2	AAAAA	0.478	0.171	-0.306	AAAAA	0.478	0.176	-0.302	AAAAA	0.478	0.222	-0.255
3	TGATG	0.393	0.207	-0.186	GAGCA	0.253	0.116	-0.137	TGATG	0.393	0.235	-0.158
4	GCAAT	0.309	0.156	-0.154	TGATG	0.393	0.258	-0.136	CTGAT	0.309	0.171	-0.138
5	CTGAT	0.309	0.157	-0.152	AGTAG	0.169	0.034	-0.135	GATGT	0.309	0.177	-0.132
6	GATGC	0.281	0.137	-0.144	CTGAT	0.309	0.178	-0.131	GCAAT	0.309	0.182	-0.127
7	GATGT	0.309	0.169	-0.140	GATGT	0.309	0.181	-0.128	GCTGA	0.281	0.156	-0.125
8	GTTTT	0.281	0.144	-0.137	CTTTT	0.253	0.127	-0.126	TGTGA	0.225	0.101	-0.124
9	TTATC	0.309	0.172	-0.137	AGTAA	0.253	0.128	-0.125	TGGTT	0.253	0.141	-0.112
10	GTCAG	0.281	0.148	-0.133	GATGC	0.281	0.164	-0.117	GATGC	0.281	0.170	-0.111

Over-represented 5-mers

	Template				Complement				2D			
Rank	kmer	Ref %	Read %	Diff %	kmer	Ref %	Read %	Diff %	kmer	Ref %	Read %	Diff %
1	CATCA	0.112	0.268	0.156	CATCA	0.112	0.280	0.168	CATCA	0.112	0.272	0.160
2	CGGCG	0.028	0.166	0.138	CAGCA	0.056	0.218	0.162	TCAGC	0.028	0.168	0.140
3	CATCT	0.000	0.124	0.124	ATCAG	0.056	0.199	0.142	ATCAG	0.056	0.194	0.138
4	CAGCA	0.056	0.180	0.124	AACCA	0.028	0.162	0.134	AACCA	0.028	0.157	0.129
5	ATCAG	0.056	0.176	0.120	GCGAA	0.028	0.160	0.132	CATCT	0.000	0.117	0.117
6	TCAGC	0.028	0.140	0.112	TCAGC	0.028	0.155	0.127	GCATC	0.084	0.200	0.116
7	GCATC	0.084	0.195	0.110	ATAAA	0.112	0.235	0.122	CAGCA	0.056	0.170	0.114
8	CCGGC	0.028	0.136	0.107	AATAA	0.197	0.314	0.117	GACGA	0.000	0.096	0.096
9	TCATC	0.056	0.163	0.107	CGGCG	0.028	0.139	0.111	GTCGA	0.000	0.093	0.093
10	GACGA	0.000	0.106	0.106	GGCGA	0.028	0.137	0.109	CTTCA	0.112	0.206	0.093

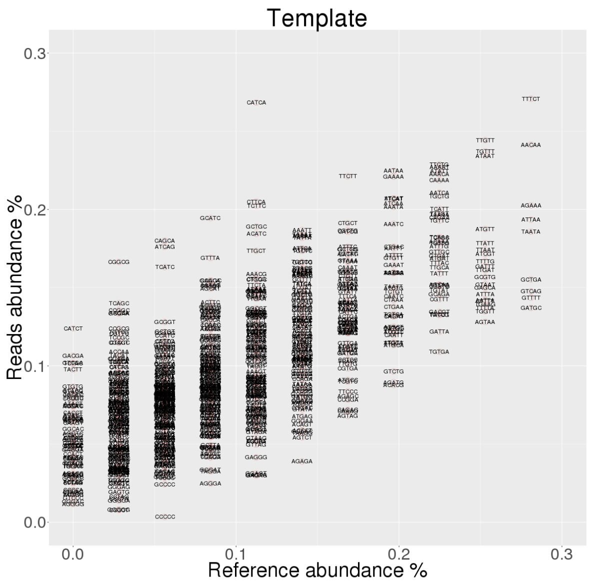

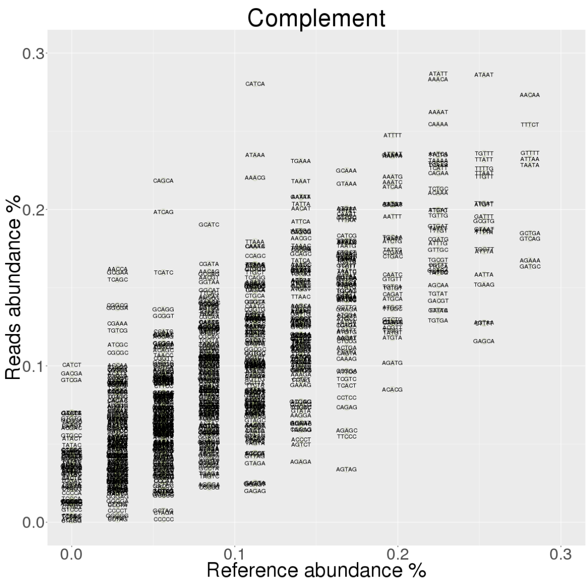

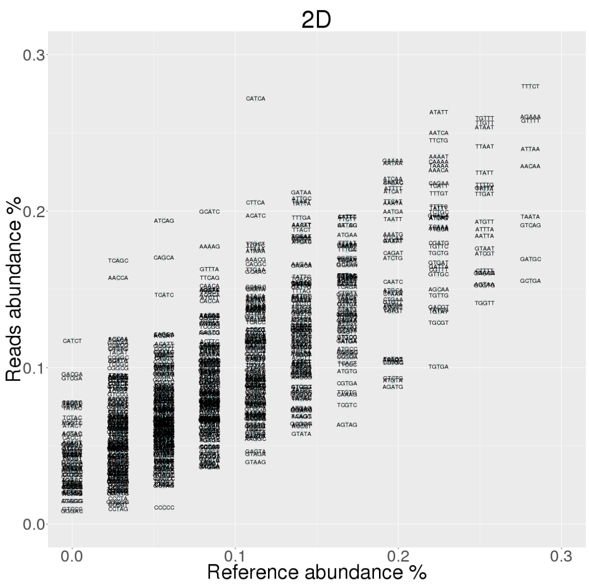

Control sequence GC content

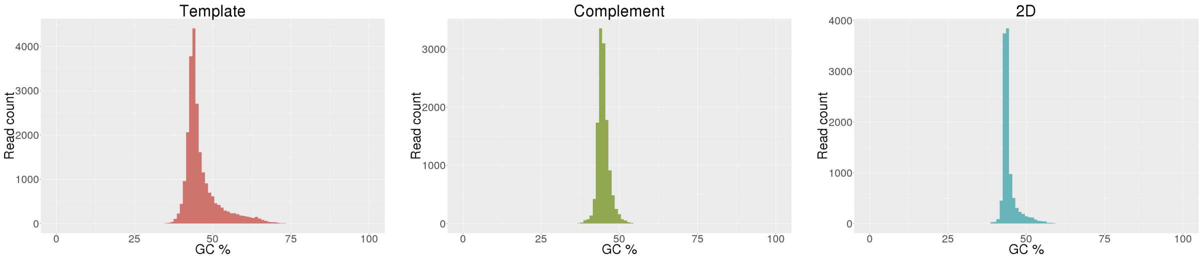

Escherichia coli error analysis

	Template	Complement	2D
Overall base identity (excluding indels)	64.71%	46.87%	62.74%
Aligned base identity (excluding indels)	89.00%	87.67%	81.74%
Identical bases per 100 aligned bases (including indels)	78.64%	71.90%	67.86%
Inserted bases per 100 aligned bases (including indels)	2.79%	2.53%	8.12%
Deleted bases per 100 aligned bases (including indels)	8.86%	15.46%	8.86%
Substitutions per 100 aligned bases (including indels)	9.72%	10.11%	15.16%
Mean insertion size	1.52	1.46	1.94
Mean deletion size	1.75	2.33	1.82

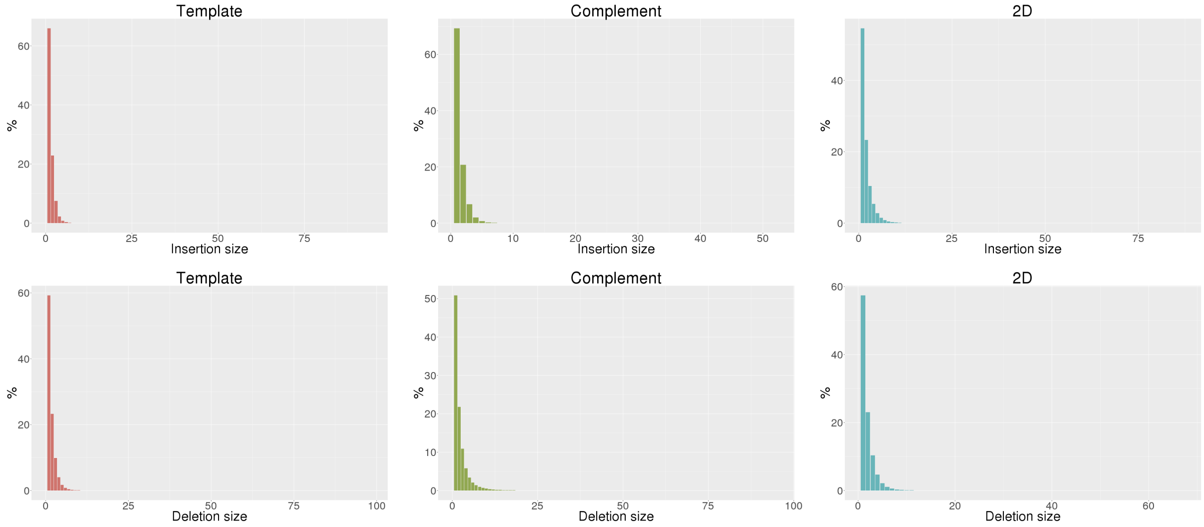

Escherichia coli read identity

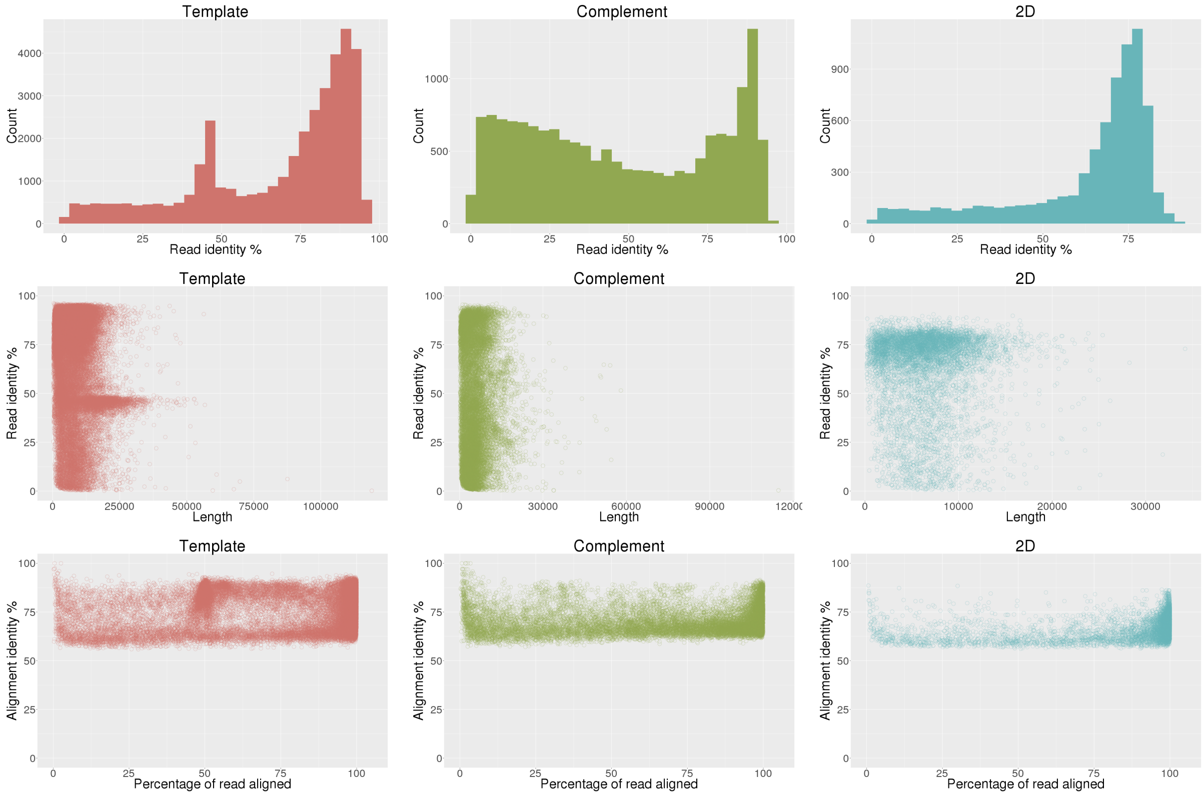

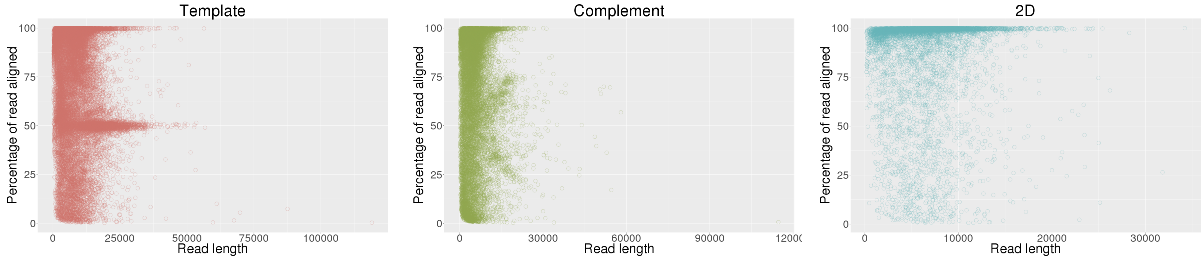

Escherichia coli perfect kmers

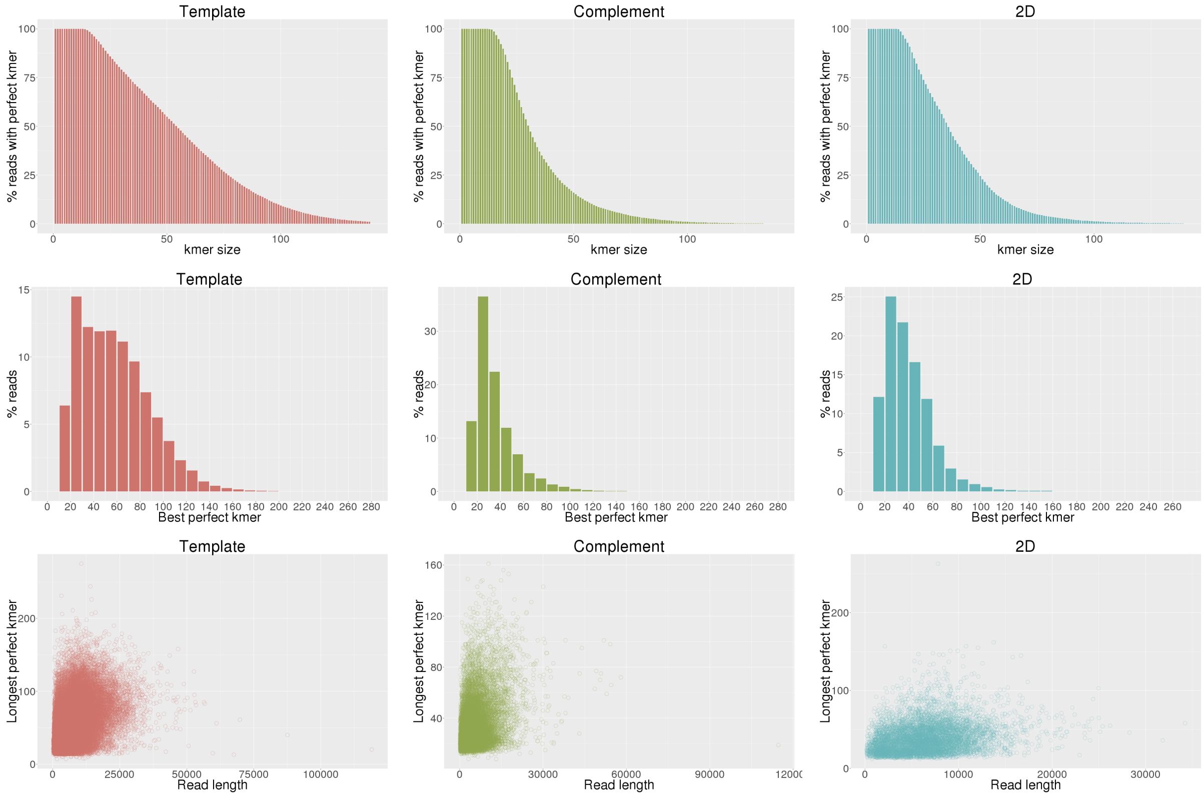

Escherichia coli coverage

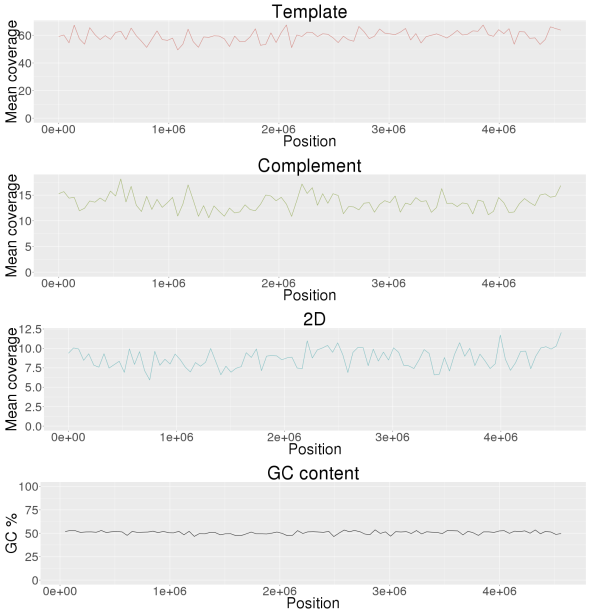

Escherichia coli 5-mer analysis

Under-represented 5-mers

	Template				Complement				2D			
Rank	kmer	Ref %	Read %	Diff %	kmer	Ref %	Read %	Diff %	kmer	Ref %	Read %	Diff %
1	TTTTT	0.251	0.035	-0.216	TTTTT	0.251	0.091	-0.160	TTTTT	0.251	0.102	-0.148
2	AAAAA	0.247	0.091	-0.156	AAAAA	0.247	0.129	-0.118	AAAAA	0.247	0.112	-0.135
3	GCCAG	0.280	0.173	-0.107	CCCAG	0.111	0.039	-0.071	GCCAG	0.280	0.208	-0.071
4	GTTTT	0.186	0.082	-0.104	TCCAG	0.171	0.102	-0.070	ATTGC	0.159	0.097	-0.062
5	TTTTC	0.198	0.120	-0.078	CCAGG	0.129	0.067	-0.063	TTGCC	0.193	0.136	-0.057
6	ATTTT	0.194	0.116	-0.078	AAAGA	0.132	0.077	-0.055	TGCCA	0.182	0.125	-0.057
7	TTTTG	0.172	0.096	-0.076	CCCGG	0.101	0.046	-0.055	TATTG	0.123	0.067	-0.056
8	TTTTA	0.147	0.073	-0.074	CCAGA	0.163	0.111	-0.052	AATAT	0.141	0.086	-0.055
9	CGCCA	0.288	0.217	-0.071	ATCCC	0.086	0.038	-0.048	AATAA	0.148	0.095	-0.053
10	CCAGA	0.163	0.093	-0.070	CCTGG	0.130	0.083	-0.047	ATATT	0.139	0.087	-0.052

Over-represented 5-mers

	Template				Complement				2D			
Rank	kmer	Ref %	Read %	Diff %	kmer	Ref %	Read %	Diff %	kmer	Ref %	Read %	Diff %
1	CGGCG	0.221	0.474	0.253	CGGCG	0.221	0.338	0.117	CGGCG	0.221	0.388	0.167
2	GCGGC	0.210	0.418	0.208	GCGGC	0.210	0.316	0.106	GCGGC	0.210	0.337	0.128
3	GGCGG	0.190	0.376	0.186	CGCTG	0.259	0.357	0.097	GGCGG	0.190	0.316	0.126
4	CGCCG	0.219	0.379	0.160	TGCCG	0.220	0.313	0.093	CCGGC	0.141	0.264	0.124
5	GGCCG	0.069	0.228	0.158	CGCCG	0.219	0.311	0.091	CGGTG	0.160	0.268	0.108
6	CCGGC	0.141	0.282	0.141	CATCA	0.202	0.287	0.085	CGCCG	0.219	0.327	0.108
7	CGGTG	0.160	0.300	0.140	GCCGC	0.209	0.288	0.079	CACCG	0.166	0.271	0.105
8	GCCGC	0.209	0.344	0.135	AAATG	0.128	0.204	0.076	GGCTG	0.161	0.262	0.101
9	CGCGG	0.137	0.269	0.132	GCAAA	0.196	0.272	0.075	GGCCG	0.069	0.168	0.098
10	CGGCC	0.072	0.199	0.127	TGGCG	0.275	0.349	0.074	GCTGG	0.279	0.377	0.098

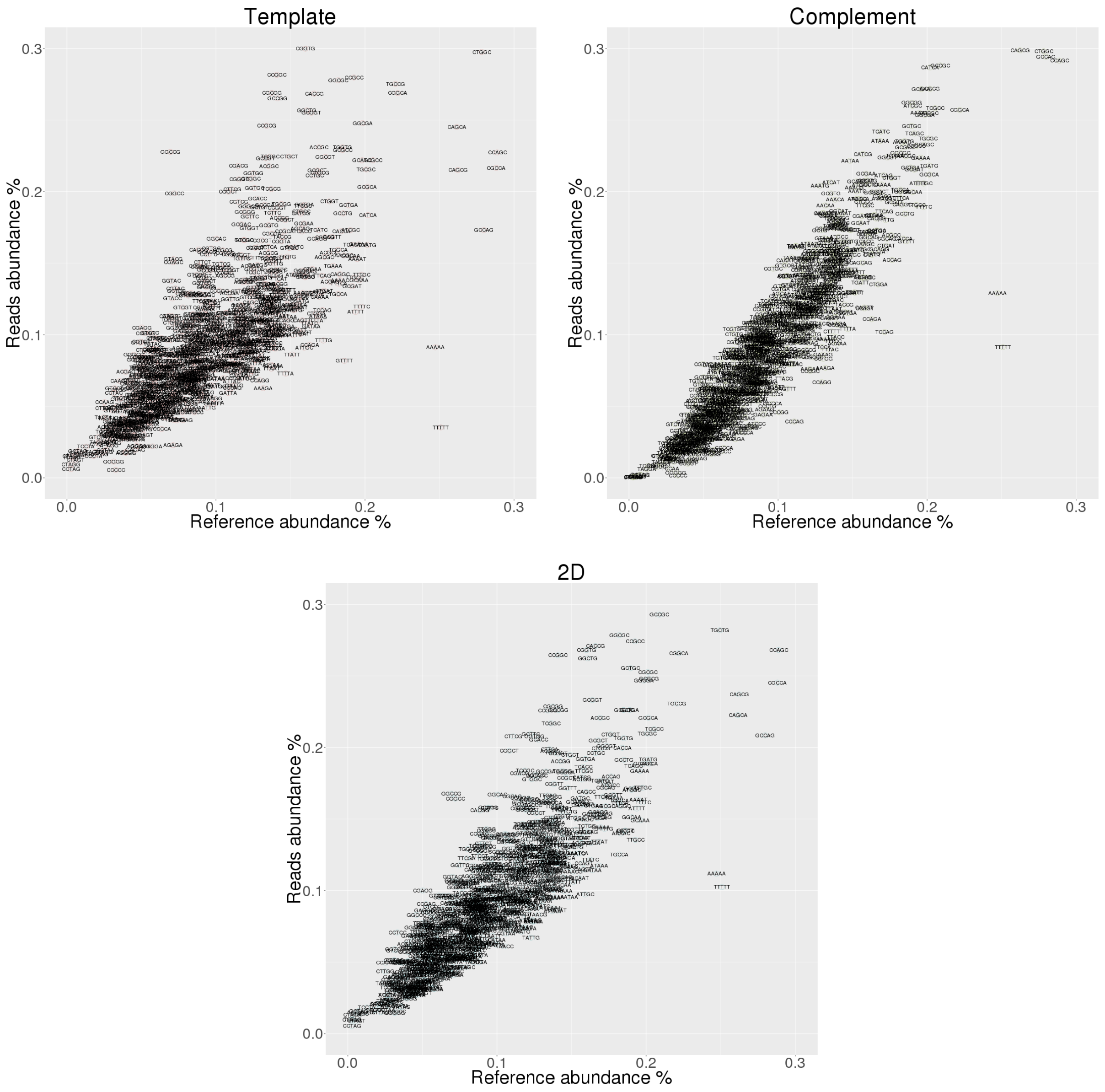

Escherichia coli GC content

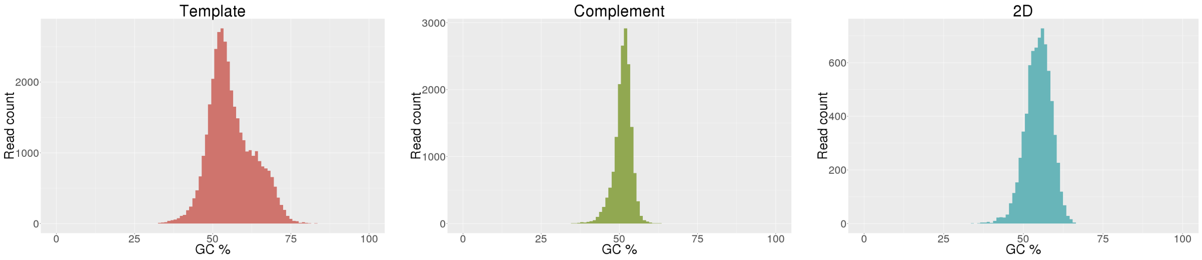

All reference 21mer analysis

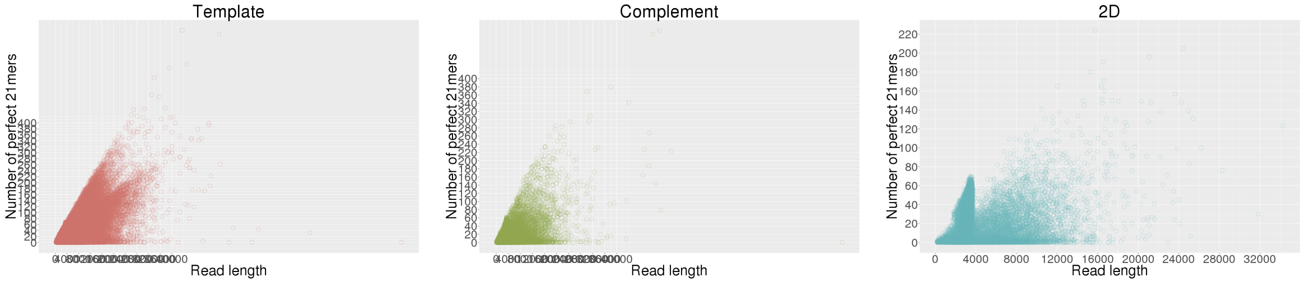

All reference substitutions

		Template substituted %				Complement substituted %				2D substituted %			
		a	c	g	t	a	c	g	t	a	c	g	t
Reference	A	0.00	8.74	16.95	4.88	0.00	5.88	14.40	5.76	0.00	8.61	13.51	6.58
	C	3.45	0.00	4.09	11.15	5.59	0.00	4.10	12.88	5.50	0.00	5.49	9.87
	G	10.81	4.42	0.00	3.48	14.23	4.38	0.00	5.61	9.79	5.70	0.00	5.45
	T	4.98	18.09	8.95	0.00	6.04	15.13	5.98	0.00	6.78	14.20	8.52	0.00

Kmer motifs before errors

3-mer error motif analysis

	Template			Complement			2D			
Rank	Insertion	Deletion	Substitution	Insertion	Deletion	Substitution	Insertion	Deletion	Substitution	
1	TTC (3.36%)	CGC (3.22%)	CGC (3.69%)	TTT (3.49%)	TTT (3.23%)	TTT (3.52%)	TTT (3.19%)	TTT (3.31%)	TTT (3.72%)	Most common
2	TTT (2.81%)	TTC (3.06%)	GCC (3.19%)	ATC (3.10%)	ATC (3.04%)	ATC (3.34%)	TTC (3.14%)	ATT (3.08%)	CGC (3.22%)	
3	ATC (2.79%)	ATC (2.75%)	ATC (3.02%)	TTC (2.91%)	CGC (2.78%)	CGC (3.09%)	ATC (2.85%)	ATC (3.08%)	ATC (3.05%)	
4	CGC (2.75%)	TTT (2.75%)	TGC (2.97%)	CGC (2.82%)	ATT (2.78%)	AAA (2.83%)	CGC (2.63%)	TTC (2.98%)	TTC (2.85%)	
5	GCC (2.44%)	GCC (2.64%)	TTC (2.86%)	ATT (2.57%)	TTC (2.77%)	TGC (2.79%)	TGC (2.42%)	CGC (2.77%)	GCC (2.67%)	
6	TGC (2.35%)	GCG (2.59%)	ACC (2.81%)	GCG (2.48%)	AAA (2.53%)	AAC (2.74%)	ATT (2.30%)	TGC (2.53%)	TGC (2.66%)	
7	ACC (2.20%)	GGC (2.55%)	GGC (2.77%)	TGC (2.44%)	TGC (2.53%)	GCC (2.63%)	GCG (2.22%)	ACC (2.44%)	AAA (2.58%)	
8	GCG (2.18%)	ACC (2.50%)	TTT (2.70%)	AAC (2.27%)	GAT (2.28%)	TTC (2.53%)	GCC (2.18%)	GCG (2.15%)	ACC (2.42%)	
9	AAC (2.14%)	TGC (2.34%)	AAC (2.68%)	AAT (2.26%)	ATG (2.26%)	GCG (2.32%)	CTG (2.16%)	GCC (2.08%)	GCG (2.41%)	
10	GGC (2.08%)	ATT (2.30%)	AGC (2.43%)	GAT (2.25%)	AAT (2.23%)	ATG (2.20%)	TCA (2.13%)	GAT (2.05%)	GGC (2.37%)	
	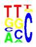	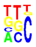	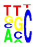	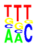	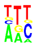	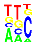	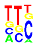	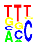	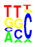	
-10	CCC (1.07%)	TAT (0.98%)	GTA (0.97%)	GAG (1.01%)	ACT (0.92%)	AGA (0.90%)	TAC (0.99%)	GAG (0.98%)	ACA (0.91%)	Least common
-9	GAG (0.97%)	AGA (0.97%)	CAT (0.95%)	AGA (0.88%)	CCC (0.89%)	TCT (0.90%)	AGT (0.98%)	ACA (0.95%)	TAT (0.90%)	
-8	AGT (0.94%)	GTA (0.88%)	CCT (0.88%)	CCC (0.83%)	CCT (0.84%)	GTA (0.89%)	GTA (0.92%)	TAT (0.92%)	GTA (0.88%)	
-7	TAA (0.91%)	CCT (0.87%)	ACT (0.87%)	AGT (0.80%)	GAG (0.76%)	CCT (0.72%)	GAG (0.91%)	AGA (0.83%)	ACT (0.84%)	
-6	AGA (0.83%)	AGT (0.85%)	AGT (0.68%)	AGG (0.80%)	AGG (0.75%)	ACT (0.66%)	GGA (0.87%)	GTA (0.79%)	AGA (0.73%)	
-5	GGA (0.81%)	TAA (0.81%)	AGA (0.68%)	GTA (0.75%)	AGT (0.75%)	GGA (0.61%)	AGA (0.86%)	AGT (0.78%)	AGT (0.70%)	
-4	AGG (0.79%)	AGG (0.78%)	GGG (0.65%)	GGA (0.74%)	GTA (0.70%)	AGT (0.61%)	AGG (0.85%)	AGG (0.77%)	GGG (0.68%)	
-3	GGG (0.52%)	GGG (0.59%)	GGA (0.62%)	GGG (0.47%)	GGG (0.47%)	GGG (0.53%)	GGG (0.57%)	GGG (0.53%)	GGA (0.65%)	
-2	TAG (0.49%)	TAG (0.43%)	TAG (0.46%)	TAG (0.42%)	TAG (0.35%)	TAG (0.43%)	TAG (0.43%)	TAG (0.40%)	TAG (0.45%)	
-1	CTA (0.49%)	CTA (0.36%)	CTA (0.32%)	CTA (0.28%)	CTA (0.26%)	CTA (0.26%)	CTA (0.36%)	CTA (0.36%)	CTA (0.30%)	
	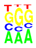	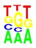	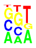	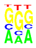	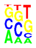	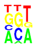	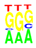	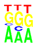	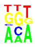	

Kmer space for 3-mers: 64 Random chance for any given 3-mer: 1.56%

4-mer error motif analysis

Rank	Template			Complement			2D			
	Insertion	Deletion	Substitution	Insertion	Deletion	Substitution	Insertion	Deletion	Substitution	
1	TTTC (1.10%)	CGCC (1.12%)	CGCC (1.36%)	TTTT (1.31%)	TTTC (1.10%)	CGCC (1.13%)	TTTT (1.13%)	TTTT (1.31%)	TTTT (1.27%)	Most common
2	CGCC (1.02%)	GCGC (1.07%)	GCGC (1.17%)	ATTT (0.99%)	TTTT (1.07%)	TTTT (1.11%)	TTTC (1.02%)	TATT (1.24%)	CGCC (1.11%)	
3	GCGC (0.89%)	TTTC (1.07%)	CAGC (1.04%)	TTTC (0.95%)	ATGC (0.96%)	GATG (0.99%)	ATTT (0.91%)	ATGC (1.13%)	GCGC (1.04%)	
4	CTTC (0.89%)	CGGC (0.98%)	CGGC (1.00%)	GATG (0.89%)	CAGC (0.95%)	CATC (0.96%)	CGCC (0.88%)	TTTC (1.10%)	ATTT (1.00%)	
5	TTTT (0.88%)	CAGC (0.91%)	CATC (0.94%)	GCGC (0.89%)	GATG (0.94%)	AAAC (0.96%)	CTTC (0.86%)	GATG (0.93%)	AAAA (0.99%)	
6	ATTT (0.82%)	GGCG (0.85%)	ACGC (0.90%)	CGCC (0.87%)	TGAT (0.91%)	GCGC (0.94%)	GATG (0.84%)	TGAT (0.93%)	GATG (0.95%)	
7	CAGC (0.82%)	CATC (0.85%)	CTGC (0.90%)	AATC (0.86%)	GCGC (0.91%)	AAAA (0.94%)	GCGC (0.84%)	TGCT (0.88%)	TTTC (0.92%)	
8	GTTC (0.81%)	TTTT (0.84%)	CACC (0.88%)	TGAT (0.84%)	TATT (0.89%)	ATCA (0.93%)	AATC (0.83%)	AATT (0.87%)	GTTT (0.91%)	
9	GCTG (0.78%)	ATGC (0.78%)	CCGC (0.87%)	CAGC (0.84%)	CGCC (0.89%)	ATTT (0.89%)	CAGC (0.82%)	AACC (0.86%)	CATC (0.88%)	
10	GATG (0.78%)	TATT (0.77%)	TGGC (0.86%)	CATC (0.81%)	ATTT (0.87%)	TGCC (0.86%)	GCTG (0.79%)	GCGC (0.85%)	TTCC (0.85%)	
	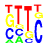	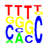	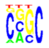	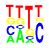	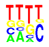	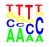	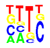	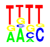	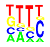	
-10	TCTA (0.12%)	ACTA (0.10%)	GCTA (0.11%)	CTTA (0.09%)	CTTG (0.09%)	CAAG (0.08%)	TTAG (0.11%)	TTAG (0.09%)	TAGT (0.10%)	Least common
-9	GGGA (0.11%)	TAGA (0.10%)	GGGA (0.09%)	CTAA (0.09%)	CAAG (0.09%)	TAGT (0.07%)	CTTA (0.10%)	CAAG (0.09%)	CTAA (0.10%)	
-8	CTTA (0.11%)	TTAG (0.10%)	GGGG (0.09%)	TCTA (0.08%)	CTTA (0.08%)	CCCT (0.07%)	GGGG (0.10%)	TCTA (0.08%)	GCTA (0.10%)	
-7	AGGG (0.10%)	TCTA (0.09%)	ACTA (0.08%)	GGGA (0.07%)	ACTA (0.07%)	GGGA (0.07%)	CTAA (0.09%)	CTAA (0.08%)	GGGA (0.09%)	
-6	CCTA (0.09%)	CTTA (0.08%)	GAGT (0.08%)	TAGA (0.07%)	TAGA (0.07%)	TAGA (0.07%)	ACTA (0.09%)	GGGG (0.07%)	CCTA (0.08%)	
-5	CTAA (0.09%)	GGGG (0.08%)	CCTA (0.08%)	ACTA (0.06%)	TCTA (0.05%)	ACTA (0.05%)	TCTA (0.08%)	TAGA (0.07%)	ACTA (0.08%)	
-4	TAGA (0.09%)	CTAA (0.08%)	TCTA (0.07%)	GGGG (0.06%)	GGGG (0.05%)	CCTA (0.05%)	TAGG (0.08%)	CTTA (0.06%)	TCTA (0.07%)	
-3	GGGG (0.08%)	TAGG (0.06%)	TAGG (0.07%)	TAGG (0.05%)	TAGG (0.05%)	TCTA (0.05%)	CCTA (0.08%)	CCTA (0.06%)	TAGA (0.05%)	
-2	TAGG (0.07%)	CCTA (0.05%)	TAGA (0.06%)	CCTA (0.04%)	CCTA (0.04%)	TAGG (0.05%)	TAGA (0.07%)	TAGG (0.04%)	TAGG (0.05%)	
-1	CTAG (0.02%)	CTAG (0.01%)	CTAG (0.01%)	CTAG (0.00%)	CTAG (0.00%)	CTAG (0.00%)	CTAG (0.02%)	CTAG (0.01%)	CTAG (0.01%)	
	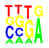	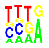	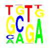	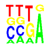	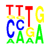	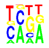	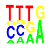	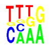	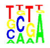	

Kmer space for 4-mers: 256 Random chance for any given 4-mer: 0.39%

5-mer error motif analysis

Rank	Template			Complement			2D			
	Insertion	Deletion	Substitution	Insertion	Deletion	Substitution	Insertion	Deletion	Substitution	
1	ATTTC (0.35%)	TTATT (0.41%)	CCAGC (0.42%)	TTTTT (0.44%)	TTATT (0.50%)	TGATG (0.47%)	TTTTT (0.41%)	TTATT (0.93%)	TGATG (0.41%)	Most common
2	CGCCA (0.34%)	GCGGC (0.41%)	CCGCC (0.38%)	TGATG (0.41%)	GATGC (0.43%)	TTATC (0.44%)	TGATG (0.35%)	GATGC (0.63%)	TTATC (0.38%)	
3	GTTTC (0.33%)	TTTTC (0.37%)	CTGGC (0.37%)	GTTTT (0.37%)	ATGAT (0.42%)	TCGCC (0.37%)	CGCCA (0.35%)	TCGGA (0.60%)	TATTT (0.37%)	
4	TGATG (0.31%)	GGCGC (0.35%)	GCGGC (0.37%)	TTATC (0.36%)	TGATG (0.41%)	TTCGC (0.34%)	AAATC (0.34%)	CTGCT (0.60%)	GATGC (0.35%)	
5	CCAGC (0.31%)	GATGC (0.35%)	TCGCC (0.36%)	ATTTT (0.36%)	TTTTC (0.39%)	CTGGC (0.34%)	CCAGC (0.33%)	ATGAT (0.56%)	CCAGC (0.34%)	
6	TGTTC (0.30%)	CCAGC (0.33%)	GCGCC (0.34%)	CCAGC (0.33%)	ATTTT (0.38%)	GATGC (0.34%)	GATGC (0.33%)	AGCCT (0.55%)	GATTT (0.33%)	
7	TTTTC (0.28%)	GCGAC (0.31%)	GATGC (0.33%)	CTGGC (0.33%)	TTATC (0.37%)	AATCA (0.34%)	ATTTC (0.33%)	TAGCA (0.55%)	TTTTT (0.33%)	
8	GCGGC (0.27%)	ATTTT (0.30%)	GCCGC (0.33%)	TGGCG (0.31%)	CCAGC (0.36%)	CCAGC (0.33%)	TTATC (0.31%)	TTTTT (0.50%)	TCGCC (0.33%)	
9	CCGCC (0.27%)	TGATG (0.30%)	TGATG (0.32%)	ACGTT (0.31%)	GCGGC (0.35%)	TCATC (0.33%)	GCTTC (0.31%)	TGGAA (0.48%)	TTTTC (0.32%)	
10	TCTTC (0.27%)	CCGCC (0.30%)	GGCGC (0.32%)	GATTT (0.30%)	TATCG (0.34%)	AAAAC (0.32%)	GTTTT (0.30%)	CGTGT (0.46%)	CTGGC (0.32%)	
	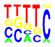	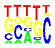	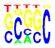	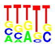	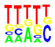	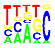	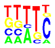	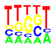	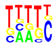	
-10	TAGGG (0.01%)	GGGGG (0.01%)	CTCTA (0.01%)	CCCTA (0.00%)	CTTAG (0.00%)	CTAGA (0.00%)	CTAGA (0.01%)	ATAGG (0.01%)	TAGGT (0.01%)	Least common
-9	CCTGG (0.01%)	TAGGA (0.01%)	CCCTA (0.01%)	TCCTA (0.00%)	GCTAG (0.00%)	TAGGA (0.00%)	CCCTA (0.01%)	TCCTA (0.00%)	CTCTA (0.01%)	
-8	CCTAG (0.01%)	GCTAG (0.00%)	GGGGG (0.01%)	GCTAG (0.00%)	GGGGG (0.00%)	GCTAG (0.00%)	CCTAG (0.01%)	GCTAG (0.00%)	CTTAG (0.01%)	
-7	CTAGA (0.01%)	CTAGA (0.00%)	CTAGC (0.01%)	CCCCC (0.00%)	CTAGA (0.00%)	CCAAG (0.00%)	TAGGG (0.01%)	CTTAG (0.00%)	GTCTA (0.01%)	
-6	CTAGT (0.01%)	CTAGT (0.00%)	CTAGA (0.00%)	CTAGA (0.00%)	CTAGC (0.00%)	CCCTA (0.00%)	CTTAG (0.00%)	TAGGG (0.00%)	GCTAG (0.00%)	
-5	GGGGG (0.00%)	CTAGC (0.00%)	GCTAG (0.00%)	GGGGG (0.00%)	CCTAG (0.00%)	CTAGC (0.00%)	CTAGT (0.00%)	ACTAG (0.00%)	CCTAG (0.00%)	
-4	CTAGC (0.00%)	ACTAG (0.00%)	ACTAG (0.00%)	CCTAG (0.00%)	CTAGT (0.00%)	CCTAG (0.00%)	CTAGC (0.00%)	CTAGC (0.00%)	CTAGC (0.00%)	
-3	ACTAG (0.00%)	CCTAG (0.00%)	CCTAG (0.00%)	ACTAG (0.00%)	ACTAG (0.00%)	ACTAG (0.00%)	ACTAG (0.00%)	TCTAG (0.00%)	TCTAG (0.00%)	
-2	TCTAG (0.00%)	TCTAG (0.00%)	TCTAG (0.00%)	CTAGT (0.00%)	TCTAG (0.00%)	TCTAG (0.00%)	CTAGG (0.00%)	CTAGT (0.00%)	ACTAG (0.00%)	
-1	CTAGG (0.00%)	CTAGG (0.00%)	CTAGG (0.00%)	CTAGC (0.00%)	CTAGG (0.00%)	CTAGG (0.00%)	TCTAG (0.00%)	CTAGG (0.00%)	CTAGG (0.00%)	
	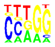	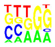	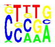	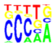	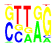	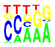	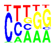	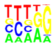	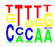	

Kmer space for 5-mers: 1024 Random chance for any given 5-mer: 0.10%
