# Supplementary material for: MinION Analysis and Reference Consortium: Phase 2 data release and analysis of R9.0 chemistry
Source: F1000Res. 2017 May 31;6:760. [Version 1] doi: 10.12688/f1000research.11354.1 (PMC5538040; doi:10.12688/f1000research.11354.1)
Supplement: Supplementary file 3 [file f1000research-6-12257-s0002.tgz › 5d2560fe-dd7a-44d1-b265-e3522e742149.zip!/SI File 3. NanoOK experiment reports/P2-Lab6-R1-2D_nanook_bwa_passfail.pdf]

NanoOK report for UBC_MARC_2D_R9_107_Called

Pass and fail counts

Type	Pass	Fail
Template	41740	84957
Complement	41740	47422
2D	41740	21885

Read lengths

Type	NumReads	TotalBases	Mean	Longest	Shortest	N50	N50Count	N90	N90Count
Template	126697	790514925	6239.41	119036	5	9419	29458	3390	83117
Complement	89162	437643944	4908.41	403748	5	7991	19495	2895	54151
2D	63625	414923263	6521.39	50913	98	9066	17212	3401	45116

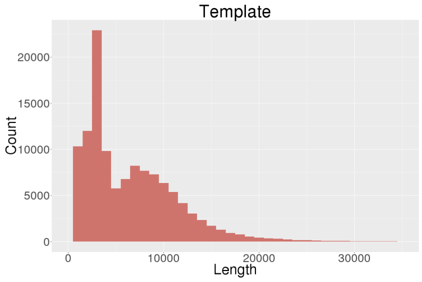

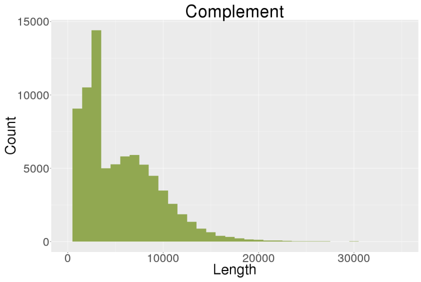

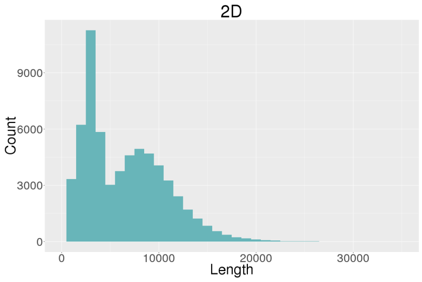

Template alignments

Number of reads	126697	
Number of reads with alignments	103391	(81.60%)
Number of reads without alignments	23306	(18.40%)

ID	Size	Number of Reads	% of Reads	Mean read length	Aligned bases	Mean coverage	Longest Perf Kmer
Control sequence	3560	25189	19.88	3428.71	79178777	22241.23	217
Escherichia coli	4641652	78202	61.72	8263.10	601834518	129.66	275

Complement alignments

Number of reads	89162	
Number of reads with alignments	70714	(79.31%)
Number of reads without alignments	18448	(20.69%)

ID	Size	Number of Reads	% of Reads	Mean read length	Aligned bases	Mean coverage	Longest Perf Kmer
Control sequence	3560	14308	16.05	2580.77	36620452	10286.64	181
Escherichia coli	4641652	56406	63.26	6672.40	357521621	77.02	228

2D alignments

Number of reads	63625	
Number of reads with alignments	60011	(94.32%)
Number of reads without alignments	3614	(5.68%)

ID	Size	Number of Reads	% of Reads	Mean read length	Aligned bases	Mean coverage	Longest Perf Kmer
Control sequence	3560	12719	19.99	3040.22	39852059	11194.40	696
Escherichia coli	4641652	47292	74.33	7712.66	373788154	80.53	713

Control sequence error analysis

	Template	Complement	2D
Overall base identity (excluding indels)	74.78%	75.93%	89.75%
Aligned base identity (excluding indels)	90.95%	90.64%	94.85%
Identical bases per 100 aligned bases (including indels)	81.57%	76.56%	87.09%
Inserted bases per 100 aligned bases (including indels)	2.73%	2.09%	2.66%
Deleted bases per 100 aligned bases (including indels)	7.58%	13.44%	5.53%
Substitutions per 100 aligned bases (including indels)	8.12%	7.90%	4.73%
Mean insertion size	1.52	1.41	1.68
Mean deletion size	1.70	2.28	1.84

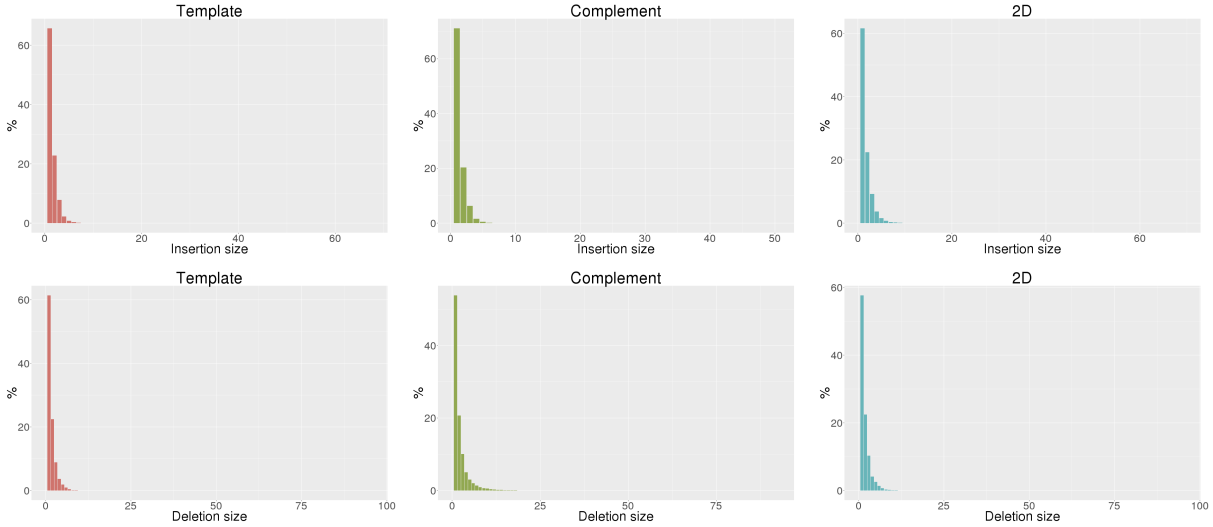

Control sequence read identity

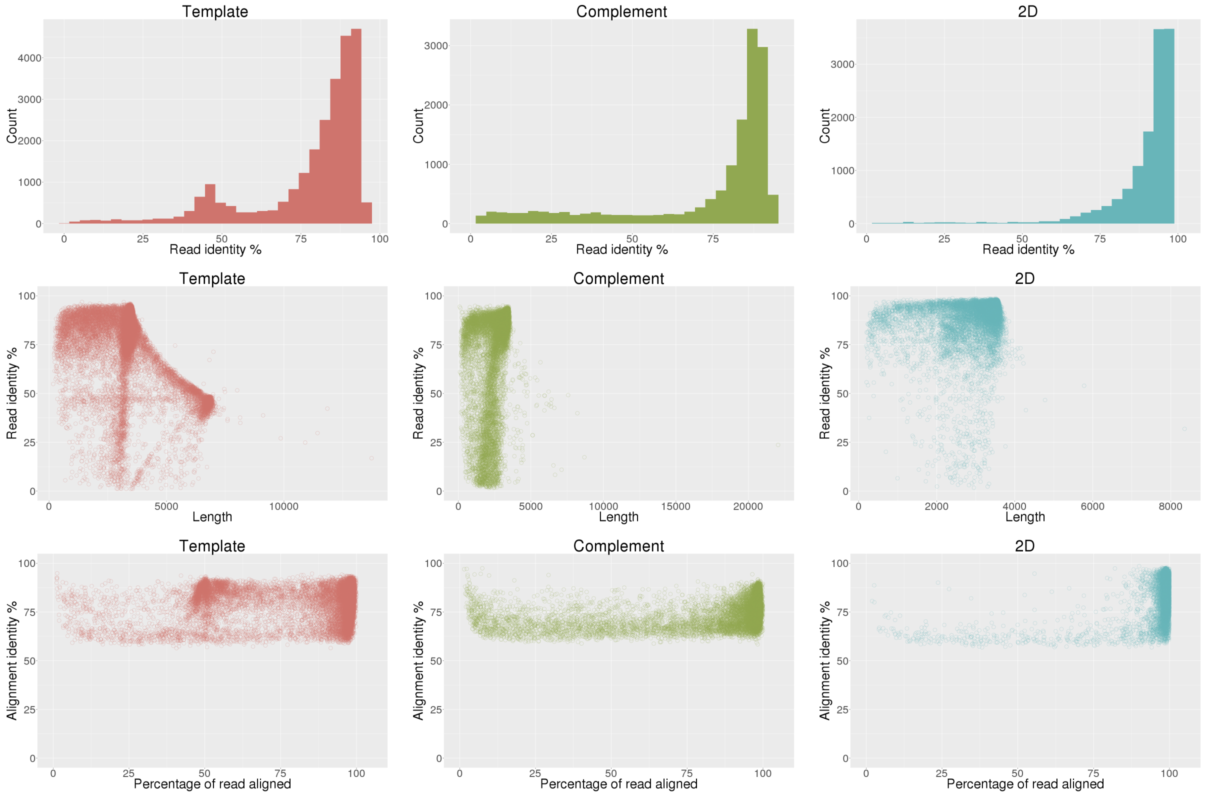

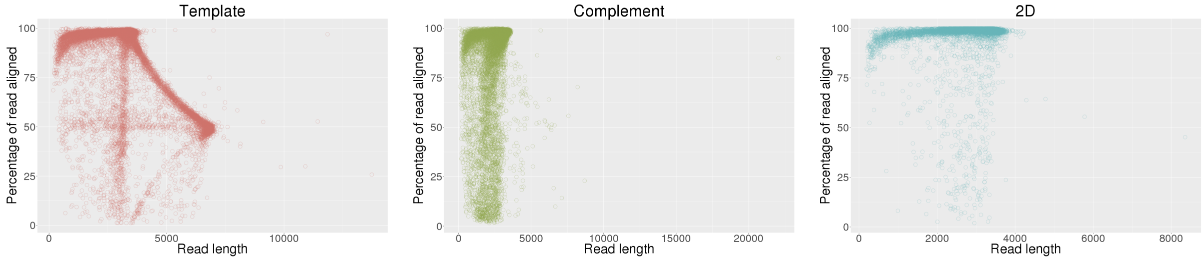

Control sequence perfect kmers

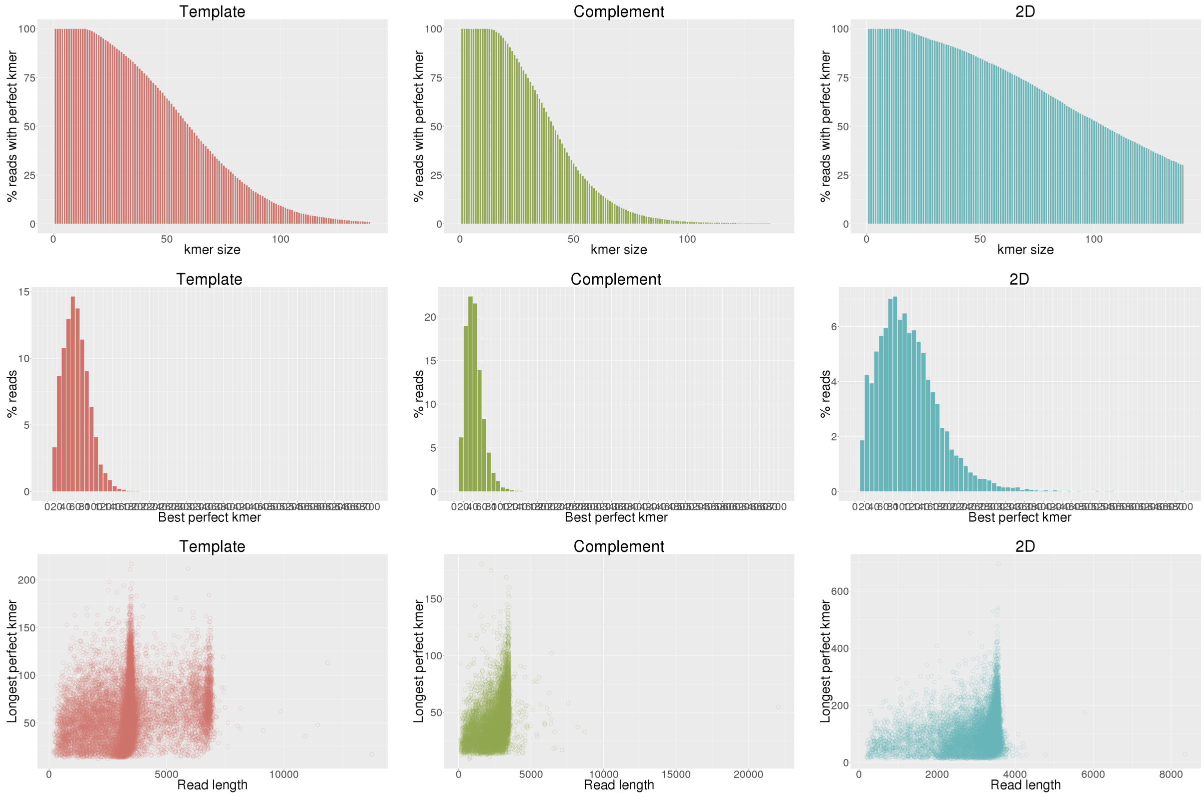

Control sequence coverage

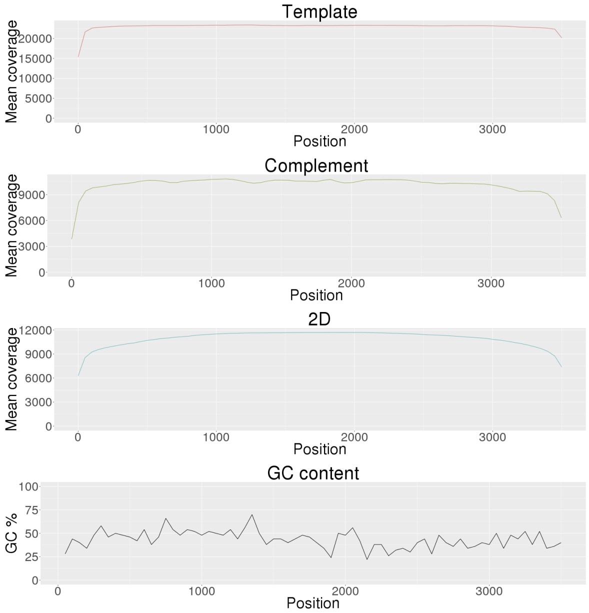

Control sequence 5-mer analysis

Under-represented 5-mers

	Template				Complement				2D			
Rank	kmer	Ref %	Read %	Diff %	kmer	Ref %	Read %	Diff %	kmer	Ref %	Read %	Diff %
1	TTTTT	0.759	0.099	-0.660	TTTTT	0.759	0.134	-0.625	TTTTT	0.759	0.205	-0.553
2	AAAAA	0.478	0.171	-0.306	AAAAA	0.478	0.176	-0.302	AAAAA	0.478	0.222	-0.256
3	TGATG	0.393	0.207	-0.186	GAGCA	0.253	0.116	-0.137	TGATG	0.393	0.235	-0.158
4	GCAAT	0.309	0.156	-0.153	TGATG	0.393	0.258	-0.136	CTGAT	0.309	0.170	-0.140
5	CTGAT	0.309	0.158	-0.151	AGTAG	0.169	0.034	-0.135	GATGT	0.309	0.177	-0.132
6	GATGC	0.281	0.137	-0.144	CTGAT	0.309	0.177	-0.132	GCAAT	0.309	0.182	-0.127
7	GATGT	0.309	0.169	-0.140	GATGT	0.309	0.182	-0.128	GCTGA	0.281	0.155	-0.126
8	GTTTT	0.281	0.144	-0.137	CTTTT	0.253	0.127	-0.126	TGTGA	0.225	0.101	-0.124
9	TTATC	0.309	0.172	-0.137	AGTAA	0.253	0.128	-0.125	TGGTT	0.253	0.141	-0.112
10	GTCAG	0.281	0.148	-0.133	GATGC	0.281	0.164	-0.117	GATGC	0.281	0.170	-0.111

Over-represented 5-mers

	Template				Complement				2D			
Rank	kmer	Ref %	Read %	Diff %	kmer	Ref %	Read %	Diff %	kmer	Ref %	Read %	Diff %
1	CATCA	0.112	0.269	0.157	CATCA	0.112	0.279	0.167	CATCA	0.112	0.273	0.161
2	CGGCG	0.028	0.165	0.137	CAGCA	0.056	0.219	0.163	TCAGC	0.028	0.169	0.141
3	CAGCA	0.056	0.180	0.124	ATCAG	0.056	0.197	0.141	ATCAG	0.056	0.194	0.138
4	CATCT	0.000	0.124	0.124	AACCA	0.028	0.162	0.134	AACCA	0.028	0.158	0.129
5	ATCAG	0.056	0.177	0.120	GCGAA	0.028	0.160	0.132	CATCT	0.000	0.117	0.117
6	TCAGC	0.028	0.141	0.113	TCAGC	0.028	0.155	0.127	GCATC	0.084	0.200	0.116
7	GCATC	0.084	0.195	0.110	ATAAA	0.112	0.235	0.123	CAGCA	0.056	0.171	0.115
8	TCATC	0.056	0.163	0.107	AATAA	0.197	0.314	0.117	GACGA	0.000	0.096	0.096
9	CCGGC	0.028	0.134	0.106	CGGCG	0.028	0.138	0.110	AAAAG	0.084	0.178	0.093
10	GACGA	0.000	0.106	0.106	GGCGA	0.028	0.137	0.108	CTTCA	0.112	0.205	0.092

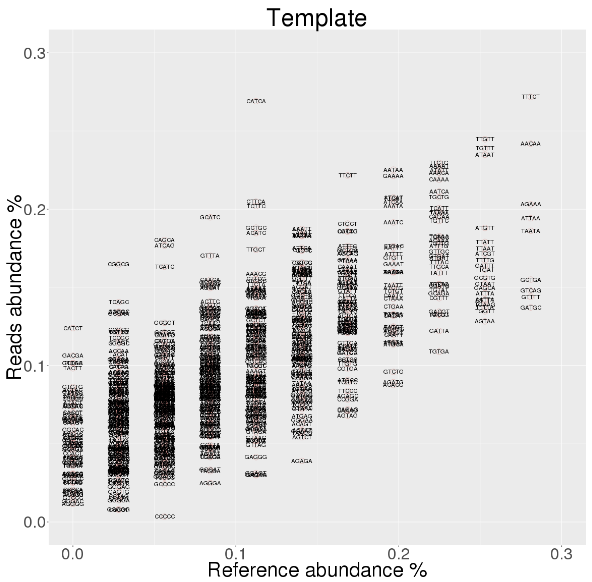

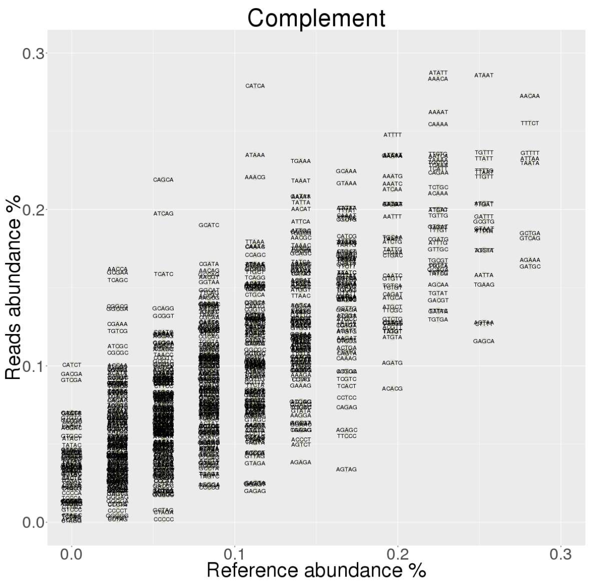

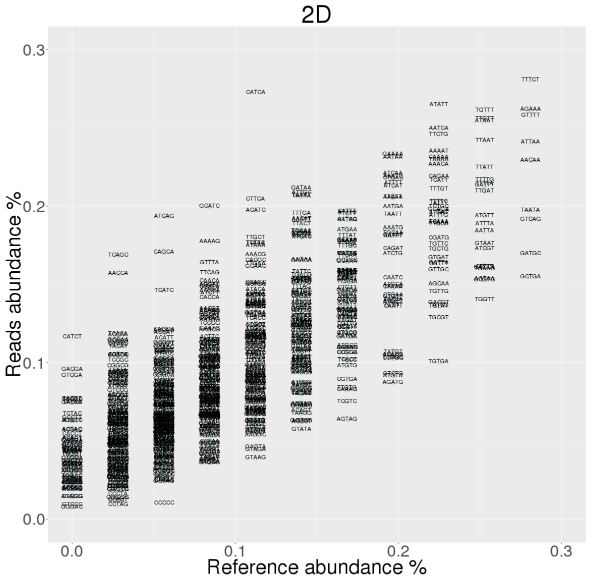

Control sequence GC content

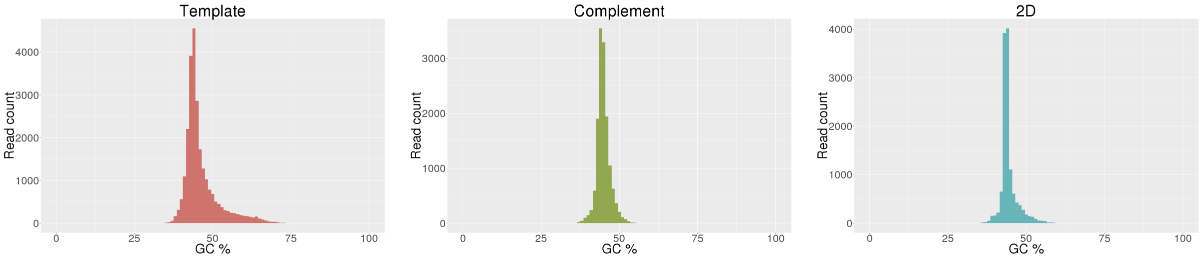

Escherichia coli error analysis

	Template	Complement	2D
Overall base identity (excluding indels)	75.34%	71.88%	88.64%
Aligned base identity (excluding indels)	90.42%	90.58%	94.40%
Identical bases per 100 aligned bases (including indels)	80.90%	75.67%	86.50%
Inserted bases per 100 aligned bases (including indels)	2.58%	2.03%	2.81%
Deleted bases per 100 aligned bases (including indels)	7.95%	14.43%	5.56%
Substitutions per 100 aligned bases (including indels)	8.58%	7.87%	5.13%
Mean insertion size	1.51	1.42	1.72
Mean deletion size	1.69	2.39	1.80

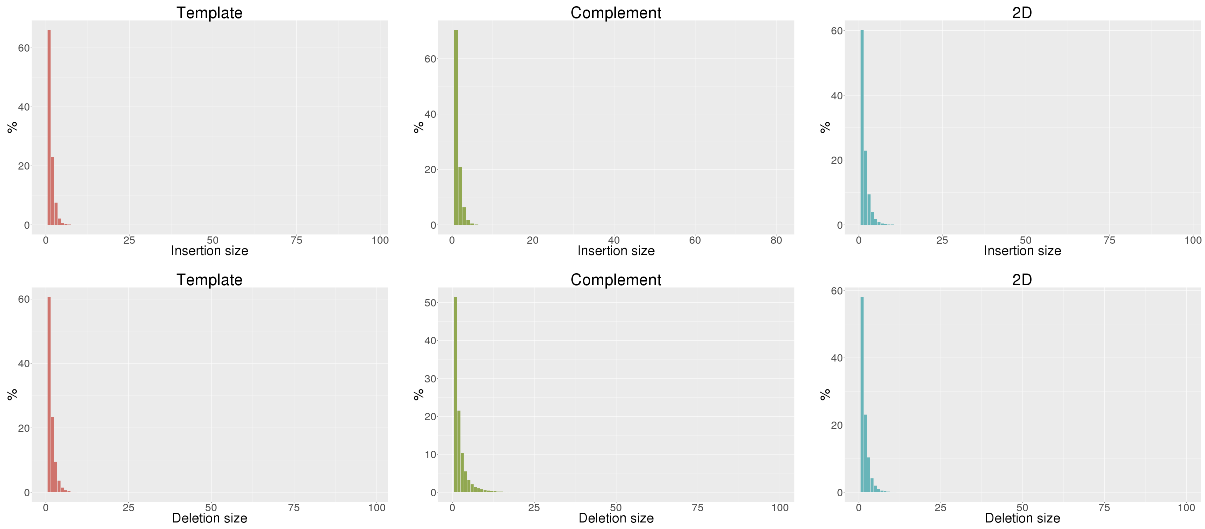

Escherichia coli read identity

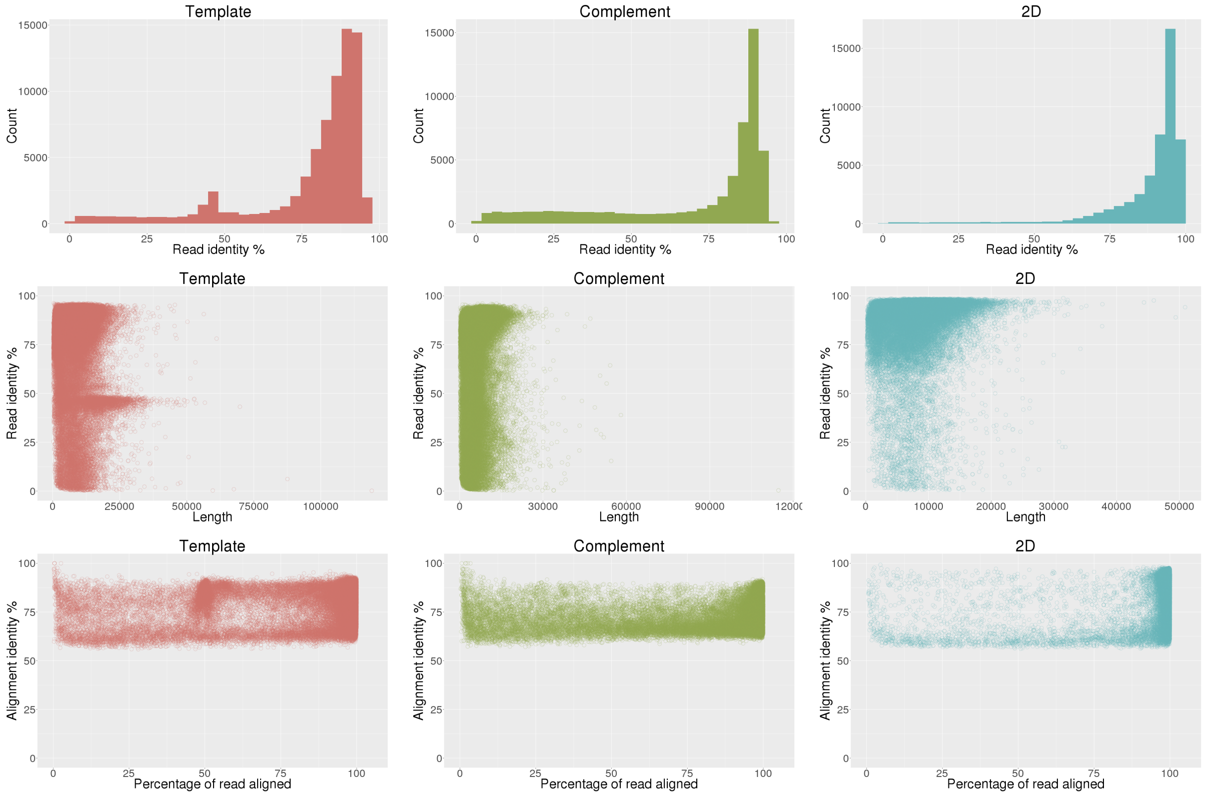

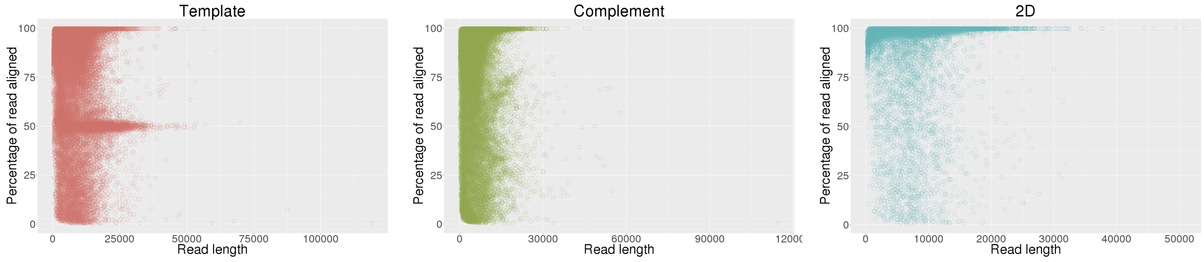

Escherichia coli perfect kmers

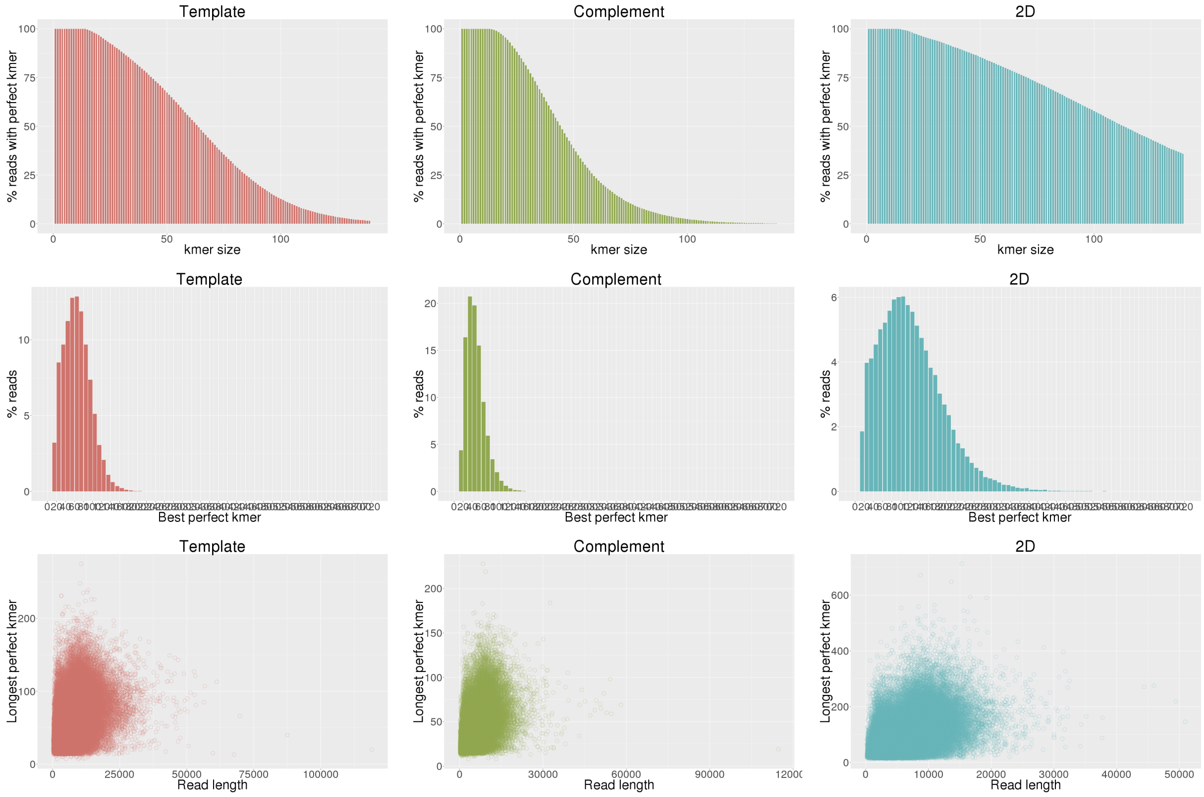

Escherichia coli coverage

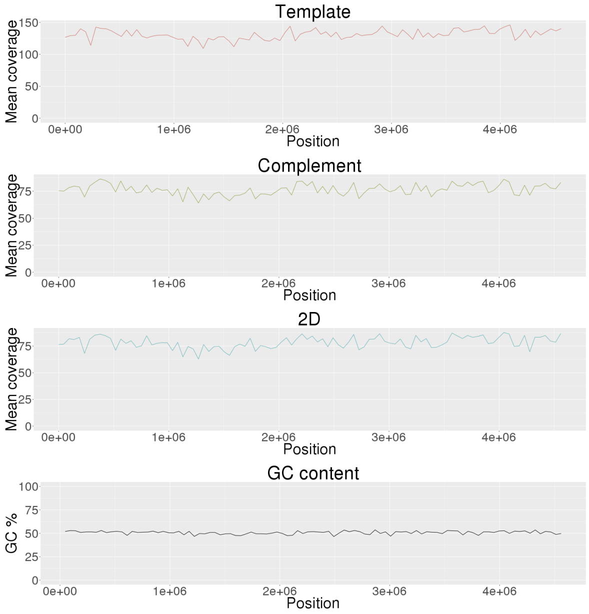

Escherichia coli 5-mer analysis

Under-represented 5-mers

	Template				Complement				2D			
Rank	kmer	Ref %	Read %	Diff %	kmer	Ref %	Read %	Diff %	kmer	Ref %	Read %	Diff %
1	TTTTT	0.251	0.038	-0.213	TTTTT	0.251	0.072	-0.179	TTTTT	0.251	0.130	-0.121
2	AAAAA	0.247	0.099	-0.149	AAAAA	0.247	0.112	-0.135	AAAAA	0.247	0.146	-0.101
3	GTTTT	0.186	0.088	-0.098	CCAGG	0.129	0.065	-0.064	CCTGG	0.130	0.051	-0.080
4	GCCAG	0.280	0.182	-0.097	CCTGG	0.130	0.068	-0.062	CCAGG	0.129	0.055	-0.074
5	TTTTG	0.172	0.104	-0.068	CCCAG	0.111	0.049	-0.062	GCCAG	0.280	0.227	-0.053
6	TTTTC	0.198	0.130	-0.067	TCCAG	0.171	0.114	-0.058	CAGGC	0.183	0.150	-0.033
7	ATTTT	0.194	0.127	-0.066	AAAGA	0.132	0.081	-0.051	GCCTG	0.185	0.155	-0.030
8	TTTTA	0.147	0.081	-0.066	GAAAG	0.130	0.083	-0.047	GTTTT	0.186	0.156	-0.030
9	CCAGG	0.129	0.065	-0.064	AGAAA	0.140	0.094	-0.046	CCCAG	0.111	0.084	-0.027
10	AAAGA	0.132	0.069	-0.063	ATCCC	0.086	0.041	-0.045	TGCCA	0.182	0.156	-0.026

Over-represented 5-mers

	Template				Complement				2D			
Rank	kmer	Ref %	Read %	Diff %	kmer	Ref %	Read %	Diff %	kmer	Ref %	Read %	Diff %
1	CGGCG	0.221	0.404	0.183	CGGCG	0.221	0.298	0.077	CGGCG	0.221	0.274	0.053
2	GCGGC	0.210	0.356	0.147	GCGGC	0.210	0.277	0.067	CGGTG	0.160	0.210	0.050
3	GGCGG	0.190	0.321	0.131	TGCCG	0.220	0.276	0.055	CACGG	0.089	0.130	0.042
4	CGCCG	0.219	0.333	0.113	GGCGG	0.190	0.242	0.052	CGCCG	0.219	0.258	0.038
5	CGGTG	0.160	0.272	0.112	CGCCG	0.219	0.271	0.052	CCGGC	0.141	0.178	0.037
6	GGCCG	0.069	0.181	0.111	AACAA	0.132	0.181	0.049	GGCGG	0.190	0.226	0.036
7	CCGGC	0.141	0.238	0.098	AATAA	0.148	0.197	0.048	CACCG	0.166	0.202	0.036
8	GCCGC	0.209	0.304	0.095	GCAAA	0.196	0.244	0.048	GCTGG	0.279	0.314	0.035
9	CGCGG	0.137	0.231	0.094	CATCA	0.202	0.248	0.046	GCGGC	0.210	0.243	0.033
10	CGGCC	0.072	0.163	0.091	AAACA	0.138	0.184	0.046	GGCTG	0.161	0.192	0.031

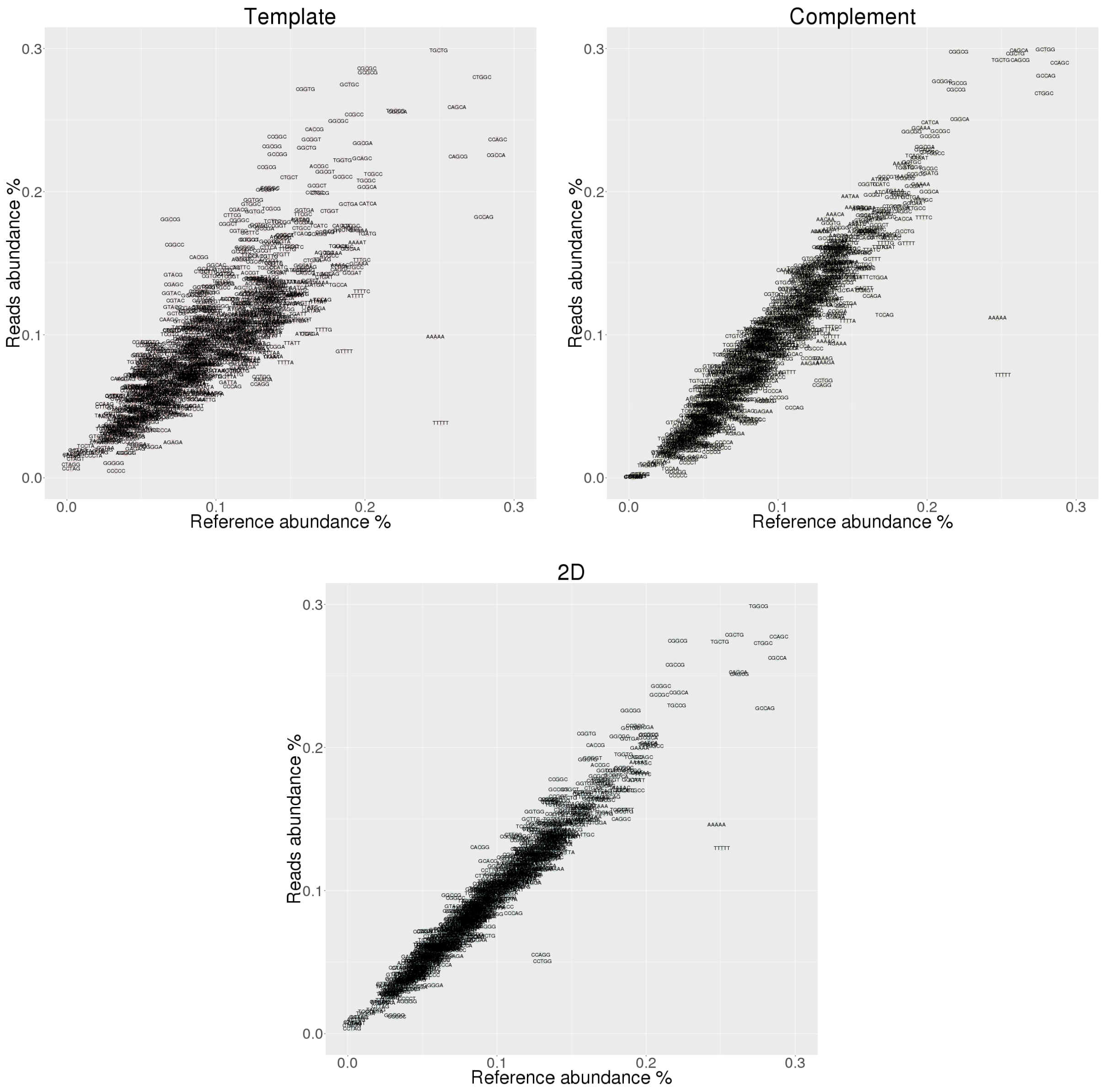

Escherichia coli GC content

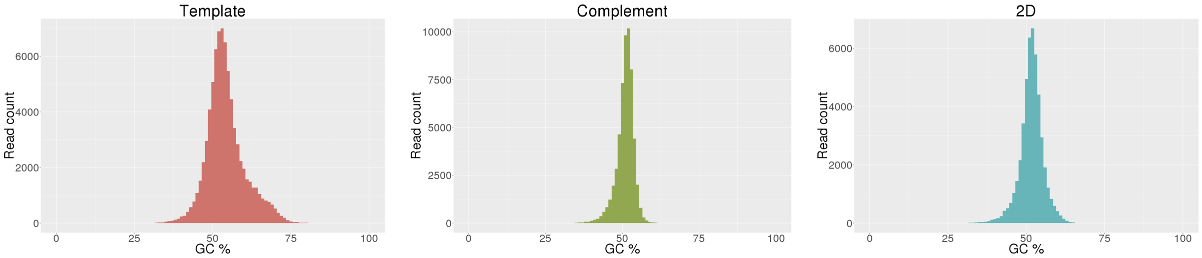

All reference 21mer analysis

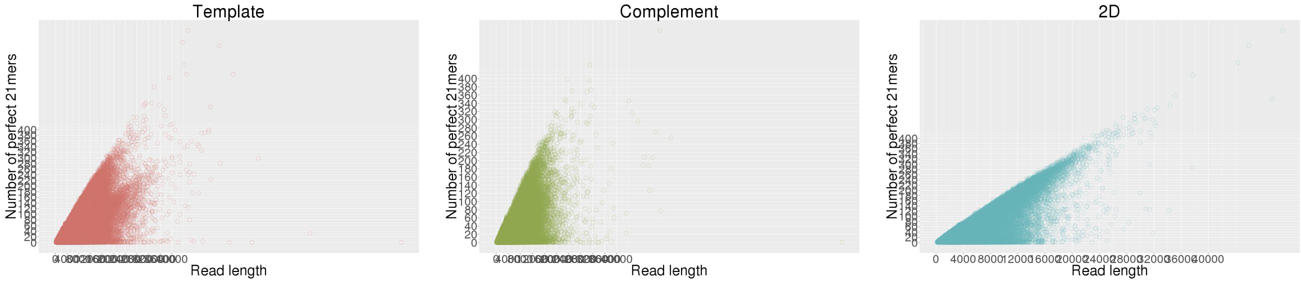

All reference substitutions

		Template substituted %				Complement substituted %				2D substituted %			
		a	c	g	t	a	c	g	t	a	c	g	t
Reference	A	0.00	8.19	16.78	4.80	0.00	5.26	14.66	5.45	0.00	7.43	14.32	5.87
	C	3.63	0.00	3.99	12.04	5.57	0.00	3.96	14.67	5.43	0.00	4.71	12.13
	G	11.70	4.28	0.00	3.62	14.68	4.13	0.00	5.47	11.76	4.88	0.00	5.27
	T	4.87	17.68	8.43	0.00	5.55	15.16	5.44	0.00	6.00	14.84	7.36	0.00

Kmer motifs before errors

3-mer error motif analysis

	Template			Complement			2D			
Rank	Insertion	Deletion	Substitution	Insertion	Deletion	Substitution	Insertion	Deletion	Substitution	
1	TTC (3.37%)	CGC (3.23%)	CGC (3.79%)	CGC (3.16%)	CGC (3.02%)	GCC (3.21%)	TTC (3.07%)	TTT (3.06%)	GCC (3.99%)	Most common
2	CGC (2.85%)	TTC (3.04%)	GCC (3.39%)	ATC (3.02%)	TTT (2.94%)	CGC (3.20%)	CGC (2.97%)	CGC (2.89%)	CGC (3.46%)	
3	ATC (2.85%)	ATC (2.80%)	ATC (3.12%)	TTT (2.89%)	ATC (2.85%)	ATC (3.19%)	TTT (2.82%)	ATC (2.85%)	ACC (3.28%)	
4	TTT (2.75%)	TTT (2.78%)	TGC (3.05%)	TTC (2.76%)	TTC (2.56%)	TTT (2.88%)	ATC (2.72%)	GCC (2.71%)	TTT (3.25%)	
5	GCC (2.54%)	GCC (2.76%)	ACC (3.01%)	GCG (2.55%)	GGC (2.49%)	TGC (2.81%)	GCC (2.64%)	TTC (2.71%)	ATC (3.14%)	
6	TGC (2.37%)	GGC (2.61%)	TTC (2.93%)	TGC (2.48%)	GCC (2.47%)	AAC (2.79%)	TGC (2.61%)	ACC (2.55%)	TGC (3.02%)	
7	ACC (2.30%)	GCG (2.61%)	GGC (2.84%)	GGC (2.45%)	GCG (2.46%)	TTC (2.60%)	GGC (2.38%)	GGC (2.48%)	TTC (2.92%)	
8	GCG (2.21%)	ACC (2.52%)	AAC (2.78%)	GCC (2.37%)	ATT (2.37%)	ACC (2.59%)	ACC (2.27%)	ATT (2.41%)	TCC (2.68%)	
9	GGC (2.15%)	TGC (2.27%)	TTT (2.59%)	AAC (2.30%)	TGC (2.35%)	AAA (2.47%)	AGC (2.19%)	TGC (2.26%)	GGC (2.57%)	
10	AAC (2.15%)	ATT (2.16%)	AGC (2.52%)	ATT (2.27%)	AAA (2.32%)	GGC (2.41%)	GCG (2.13%)	GCG (2.23%)	AAC (2.52%)	
	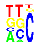	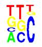	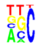	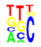	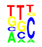	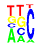	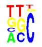	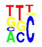	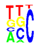	
-10	CCC (1.10%)	AGA (0.97%)	GTA (0.92%)	GAG (0.97%)	GGA (0.95%)	TGT (0.86%)	TAA (1.02%)	GGA (0.96%)	CGA (0.87%)	Least common
-9	AGT (0.93%)	GGA (0.92%)	GAG (0.91%)	AGA (0.93%)	CCC (0.90%)	TCT (0.85%)	GTA (0.98%)	AGA (0.95%)	TAT (0.85%)	
-8	GAG (0.92%)	GTA (0.87%)	ACT (0.88%)	CCC (0.92%)	GAG (0.79%)	GAG (0.82%)	AGT (0.96%)	GAG (0.92%)	GTA (0.84%)	
-7	TAA (0.92%)	AGT (0.85%)	CCT (0.86%)	GTA (0.82%)	GTA (0.78%)	ACT (0.67%)	GAG (0.95%)	AGT (0.87%)	GAG (0.84%)	
-6	AGA (0.82%)	TAA (0.84%)	AGA (0.65%)	AGG (0.79%)	AGG (0.77%)	GGA (0.64%)	AGA (0.80%)	CCT (0.86%)	GGG (0.71%)	
-5	GGA (0.80%)	CCT (0.79%)	AGT (0.64%)	GGA (0.77%)	AGT (0.73%)	GGG (0.62%)	AGG (0.78%)	GTA (0.85%)	AGA (0.67%)	
-4	AGG (0.77%)	AGG (0.77%)	GGG (0.64%)	AGT (0.77%)	CCT (0.69%)	CCT (0.61%)	GGA (0.76%)	AGG (0.74%)	AGT (0.59%)	
-3	GGG (0.51%)	GGG (0.60%)	GGA (0.59%)	GGG (0.51%)	GGG (0.54%)	AGT (0.52%)	GGG (0.57%)	GGG (0.60%)	GGA (0.56%)	
-2	CTA (0.48%)	TAG (0.42%)	TAG (0.44%)	TAG (0.37%)	TAG (0.33%)	TAG (0.40%)	TAG (0.44%)	CTA (0.40%)	TAG (0.41%)	
-1	TAG (0.47%)	CTA (0.37%)	CTA (0.31%)	CTA (0.28%)	CTA (0.27%)	CTA (0.27%)	CTA (0.40%)	TAG (0.40%)	CTA (0.31%)	
	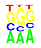	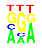	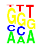	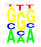	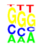	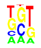	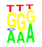	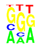	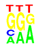	

Kmer space for 3-mers: 64 Random chance for any given 3-mer: 1.56%

4-mer error motif analysis

Rank	Template			Complement			2D			
	Insertion	Deletion	Substitution	Insertion	Deletion	Substitution	Insertion	Deletion	Substitution	
1	TTTC (1.14%)	CGCC (1.17%)	CGCC (1.45%)	CAGC (1.05%)	CAGC (1.08%)	CGCC (1.36%)	CGCC (1.08%)	CGCC (1.11%)	CGCC (1.65%)	Most common
2	CGCC (1.07%)	GCGC (1.10%)	GCGC (1.20%)	CGCC (1.02%)	CGCC (1.06%)	CAGC (1.06%)	TTTC (1.01%)	GCGC (0.97%)	TGCC (1.11%)	
3	GCGC (0.94%)	TTTC (1.04%)	CAGC (1.13%)	GCGC (1.01%)	GCGC (1.02%)	GCGC (0.99%)	CAGC (0.96%)	CAGC (0.95%)	GCGC (1.09%)	
4	CTTC (0.88%)	CGGC (1.00%)	CGGC (1.03%)	TTTC (0.90%)	TTTC (0.92%)	TGCC (0.98%)	GCGC (0.95%)	TTTT (0.91%)	TTTT (1.06%)	
5	CAGC (0.87%)	CAGC (0.97%)	CATC (0.98%)	TTTT (0.90%)	CGGC (0.91%)	CATC (0.95%)	TTTT (0.84%)	TTTC (0.89%)	CAGC (1.02%)	
6	TTTT (0.82%)	CATC (0.88%)	CACC (0.95%)	CGGC (0.88%)	TTTT (0.86%)	AAAC (0.93%)	TGGC (0.82%)	CGGC (0.89%)	CACC (1.01%)	
7	CATC (0.81%)	GGCG (0.88%)	ACGC (0.92%)	CATC (0.87%)	TGGC (0.86%)	TGGC (0.87%)	CTTC (0.82%)	CATC (0.83%)	AACC (1.00%)	
8	GCTG (0.80%)	TTTT (0.80%)	CTGC (0.90%)	TGGC (0.86%)	CATC (0.86%)	ATCA (0.87%)	CGGC (0.80%)	ATTT (0.82%)	TTCC (0.98%)	
9	GTTC (0.78%)	CACC (0.79%)	CCGC (0.90%)	ACGC (0.81%)	ATTT (0.81%)	TTTT (0.86%)	CTGC (0.77%)	GTTT (0.80%)	ATCC (0.95%)	
10	CGGC (0.76%)	GTTT (0.78%)	TGGC (0.90%)	GGCG (0.80%)	GGCG (0.80%)	AACC (0.83%)	CATC (0.76%)	CACC (0.79%)	TTTC (0.91%)	
	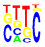	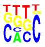	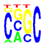	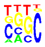	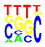	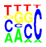	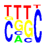	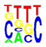	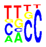	
-10	CTTA (0.12%)	ACTA (0.10%)	TAGT (0.10%)	TAGT (0.10%)	TAGT (0.09%)	GAGT (0.08%)	AGGG (0.11%)	CCCT (0.11%)	CTAA (0.10%)	Least common
-9	TCTA (0.11%)	TTAG (0.10%)	GGGA (0.09%)	GGGA (0.09%)	CCCT (0.09%)	GGGA (0.08%)	ACTA (0.10%)	TTAG (0.10%)	GAGT (0.09%)	
-8	GGGA (0.11%)	CTTA (0.10%)	GGGG (0.09%)	CTAA (0.09%)	CTAA (0.09%)	GGGG (0.07%)	GGGA (0.10%)	ACTA (0.10%)	GGGA (0.09%)	
-7	AGGG (0.10%)	CCCT (0.10%)	ACTA (0.08%)	TAGA (0.08%)	TAGA (0.08%)	ACTA (0.07%)	GGGG (0.10%)	TAGA (0.10%)	ACTA (0.08%)	
-6	CTAA (0.09%)	TCTA (0.09%)	GAGT (0.08%)	TCTA (0.07%)	ACTA (0.07%)	TAGA (0.07%)	CTAA (0.09%)	CTAA (0.10%)	TAGT (0.08%)	
-5	TAGA (0.09%)	GGGG (0.09%)	TAGG (0.07%)	ACTA (0.07%)	GGGG (0.06%)	TAGG (0.06%)	TCTA (0.09%)	TCTA (0.09%)	TCTA (0.06%)	
-4	CCTA (0.08%)	CTAA (0.08%)	TCTA (0.07%)	GGGG (0.07%)	TCTA (0.06%)	TAGT (0.06%)	TAGA (0.08%)	GGGG (0.09%)	TAGG (0.06%)	
-3	GGGG (0.08%)	TAGG (0.06%)	TAGA (0.06%)	TAGG (0.05%)	TAGG (0.05%)	TCTA (0.05%)	TAGG (0.07%)	CCTA (0.06%)	CCTA (0.05%)	
-2	TAGG (0.07%)	CCTA (0.05%)	CCTA (0.06%)	CCTA (0.04%)	CCTA (0.03%)	CCTA (0.04%)	CCTA (0.06%)	TAGG (0.05%)	TAGA (0.05%)	
-1	CTAG (0.02%)	CTAG (0.01%)	CTAG (0.01%)	CTAG (0.00%)	CTAG (0.00%)	CTAG (0.00%)	CTAG (0.01%)	CTAG (0.01%)	CTAG (0.01%)	
	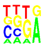	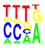	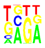	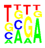	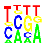	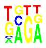	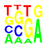	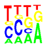	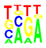	

Kmer space for 4-mers: 256   Random chance for any given 4-mer: 0.39%

5-mer error motif analysis

Rank	Template			Complement			2D			
	Insertion	Deletion	Substitution	Insertion	Deletion	Substitution	Insertion	Deletion	Substitution	
1	ATTTC (0.36%)	GCGGC (0.39%)	CCAGC (0.45%)	CCAGC (0.40%)	CCAGC (0.41%)	CCAGC (0.41%)	CCAGC (0.35%)	CCAGC (0.35%)	CCGCC (0.46%)	Most common
2	CGCCA (0.34%)	CCAGC (0.35%)	CCGCC (0.40%)	CTGGC (0.35%)	CTGGC (0.36%)	TCGCC (0.40%)	CGCCA (0.34%)	GCGGC (0.33%)	TCGCC (0.46%)	
3	GTTTC (0.34%)	GGCGC (0.35%)	TCGCC (0.39%)	TGGCG (0.32%)	GCGGC (0.35%)	CTGGC (0.37%)	CTGGC (0.33%)	CGCCA (0.32%)	CAGCC (0.41%)	
4	CCAGC (0.33%)	TTTTC (0.33%)	CTGGC (0.38%)	GCGGC (0.31%)	CGCCA (0.33%)	GCGCC (0.34%)	TCGCC (0.30%)	TCGCC (0.31%)	GCGCC (0.40%)	
5	CCGCC (0.28%)	TCGCC (0.31%)	GCGGC (0.38%)	CGCCA (0.31%)	TGGCG (0.32%)	CCGCC (0.34%)	ATTTC (0.30%)	TTATT (0.31%)	CCAGC (0.39%)	
6	GCGGC (0.28%)	CCGCC (0.31%)	GCGCC (0.36%)	CAGCG (0.31%)	TGATG (0.30%)	CGCCA (0.33%)	GCGGC (0.29%)	GGCGC (0.31%)	ACGCC (0.38%)	
7	TTTTC (0.28%)	TGGCG (0.30%)	GCCGC (0.34%)	TGATG (0.31%)	ATTTT (0.30%)	GCGGC (0.32%)	CCGCC (0.28%)	CTGGC (0.31%)	TTGCC (0.35%)	
8	TCGCC (0.28%)	CTGGC (0.30%)	ACGCC (0.33%)	ATTTT (0.30%)	TCGCC (0.30%)	TTATC (0.32%)	GTTTC (0.28%)	ATTTT (0.29%)	CTGGC (0.34%)	
9	CTGGC (0.27%)	TTATT (0.29%)	GATGC (0.32%)	AACGC (0.29%)	TTTTC (0.29%)	TGATG (0.32%)	ACGCC (0.26%)	CCGCC (0.29%)	TCACC (0.33%)	
10	TGATG (0.27%)	CGCCA (0.28%)	GGCGC (0.32%)	TTATC (0.29%)	GGCGC (0.28%)	TCATC (0.31%)	TGCGC (0.26%)	GATGC (0.28%)	AAACC (0.33%)	
	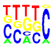	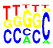	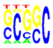	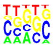	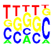	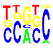	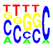	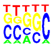	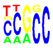	
-10	CCTGG (0.01%)	TAGGA (0.01%)	CTAGT (0.01%)	CCCCC (0.00%)	TCCTA (0.00%)	TAGGA (0.00%)	TAGGG (0.01%)	TCCTA (0.01%)	CCCTA (0.01%)	Least common
-9	GCTAG (0.01%)	GGGGG (0.01%)	TAGGA (0.01%)	GGGGG (0.00%)	GGGGG (0.00%)	GGGGG (0.00%)	TCCTA (0.01%)	TAGGA (0.01%)	CTAGC (0.01%)	
-8	CTAGC (0.00%)	GCTAG (0.00%)	CTAGC (0.01%)	GCTAG (0.00%)	CTAGC (0.00%)	GCTAG (0.00%)	CTAGC (0.01%)	GCTAG (0.00%)	TAGGA (0.00%)	
-7	GGGGG (0.00%)	CTAGT (0.00%)	GGGGG (0.01%)	CTAGC (0.00%)	GCTAG (0.00%)	CTAGC (0.00%)	GCTAG (0.01%)	CTAGC (0.00%)	CTAGT (0.00%)	
-6	CTAGT (0.00%)	CTAGC (0.00%)	GCTAG (0.00%)	CTAGA (0.00%)	CTAGA (0.00%)	CTAGA (0.00%)	CTAGT (0.00%)	CTAGT (0.00%)	GCTAG (0.00%)	
-5	CCTAG (0.00%)	ACTAG (0.00%)	ACTAG (0.00%)	CTAGT (0.00%)	CTAGT (0.00%)	ACTAG (0.00%)	CTAGA (0.00%)	CTAGA (0.00%)	ACTAG (0.00%)	
-4	CTAGA (0.00%)	CTAGA (0.00%)	CTAGA (0.00%)	CCTAG (0.00%)	ACTAG (0.00%)	TCTAG (0.00%)	ACTAG (0.00%)	ACTAG (0.00%)	CTAGA (0.00%)	
-3	ACTAG (0.00%)	TCTAG (0.00%)	TCTAG (0.00%)	ACTAG (0.00%)	CTAGG (0.00%)	CTAGG (0.00%)	TCTAG (0.00%)	CCTAG (0.00%)	TCTAG (0.00%)	
-2	TCTAG (0.00%)	CCTAG (0.00%)	CCTAG (0.00%)	TCTAG (0.00%)	TCTAG (0.00%)	CTAGT (0.00%)	CCTAG (0.00%)	TCTAG (0.00%)	CTAGG (0.00%)	
-1	CTAGG (0.00%)	CTAGG (0.00%)	CTAGG (0.00%)	CTAGG (0.00%)	CCTAG (0.00%)	CCTAG (0.00%)	CTAGG (0.00%)	CTAGG (0.00%)	CCTAG (0.00%)	
	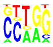	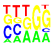	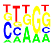	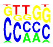	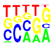	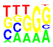	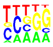	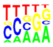	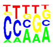	

Kmer space for 5-mers: 1024   Random chance for any given 5-mer: 0.10%
