# Supplementary material for: MinION Analysis and Reference Consortium: Phase 2 data release and analysis of R9.0 chemistry
Source: F1000Res. 2017 May 31;6:760. [Version 1] doi: 10.12688/f1000research.11354.1 (PMC5538040; doi:10.12688/f1000research.11354.1)
Supplement: Supplementary file 3 [file f1000research-6-12257-s0002.tgz › 5d2560fe-dd7a-44d1-b265-e3522e742149.zip!/SI File 3. NanoOK experiment reports/P2-Lab6-R1-2D_nanook_bwa_passonly.pdf]

NanoOK report for UBC_MARC_2D_R9_107_Called

Pass and fail counts

Type	Pass	Fail
Template	41740	0
Complement	41740	0
2D	41740	0

Read lengths

Type	NumReads	TotalBases	Mean	Longest	Shortest	N50	N50Count	N90	N90Count
Template	41740	330476731	7917.51	50265	168	10005	12837	5312	29553
Complement	41740	286214253	6857.07	47023	111	8794	12392	4506	29286
2D	41740	320813287	7685.99	50913	116	9785	12645	5117	29378

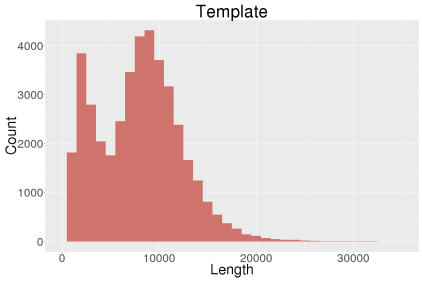

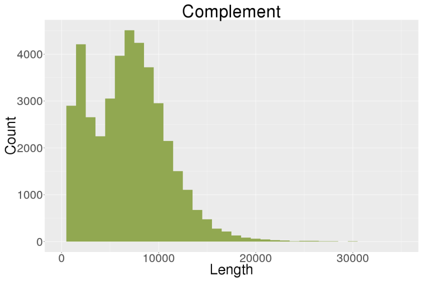

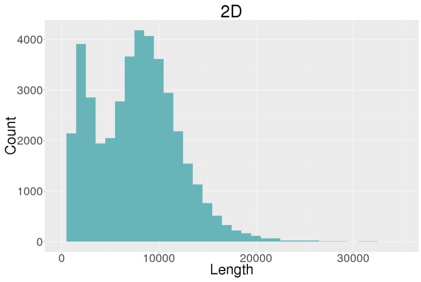

Template alignments

Number of reads	41740	
Number of reads with alignments	41673	(99.84%)
Number of reads without alignments	67	(0.16%)

ID	Size	Number of Reads	% of Reads	Mean read length	Aligned bases	Mean coverage	Longest Perf Kmer
Control sequence	3560	1662	3.98	2021.93	3301868	927.49	170
Escherichia coli	4641652	40011	95.86	8163.35	338220678	72.87	248

Complement alignments

Number of reads	41740	
Number of reads with alignments	41555	(99.56%)
Number of reads without alignments	185	(0.44%)

ID	Size	Number of Reads	% of Reads	Mean read length	Aligned bases	Mean coverage	Longest Perf Kmer
Control sequence	3560	1638	3.92	1638.89	2762637	776.02	181
Escherichia coli	4641652	39917	95.63	7075.68	296943940	63.97	228

2D alignments

Number of reads	41740	
Number of reads with alignments	41691	(99.88%)
Number of reads without alignments	49	(0.12%)

ID	Size	Number of Reads	% of Reads	Mean read length	Aligned bases	Mean coverage	Longest Perf Kmer
Control sequence	3560	1669	4.00	1785.95	3077683	864.52	454
Escherichia coli	4641652	40022	95.88	7930.35	330000615	71.10	713

Control sequence error analysis

	Template	Complement	2D
Overall base identity (excluding indels)	81.58%	78.69%	91.75%
Aligned base identity (excluding indels)	91.85%	90.96%	95.98%
Identical bases per 100 aligned bases (including indels)	83.02%	76.46%	88.86%
Inserted bases per 100 aligned bases (including indels)	2.75%	1.97%	2.27%
Deleted bases per 100 aligned bases (including indels)	6.87%	13.96%	5.14%
Substitutions per 100 aligned bases (including indels)	7.36%	7.60%	3.73%
Mean insertion size	1.51	1.40	1.63
Mean deletion size	1.66	2.34	1.83

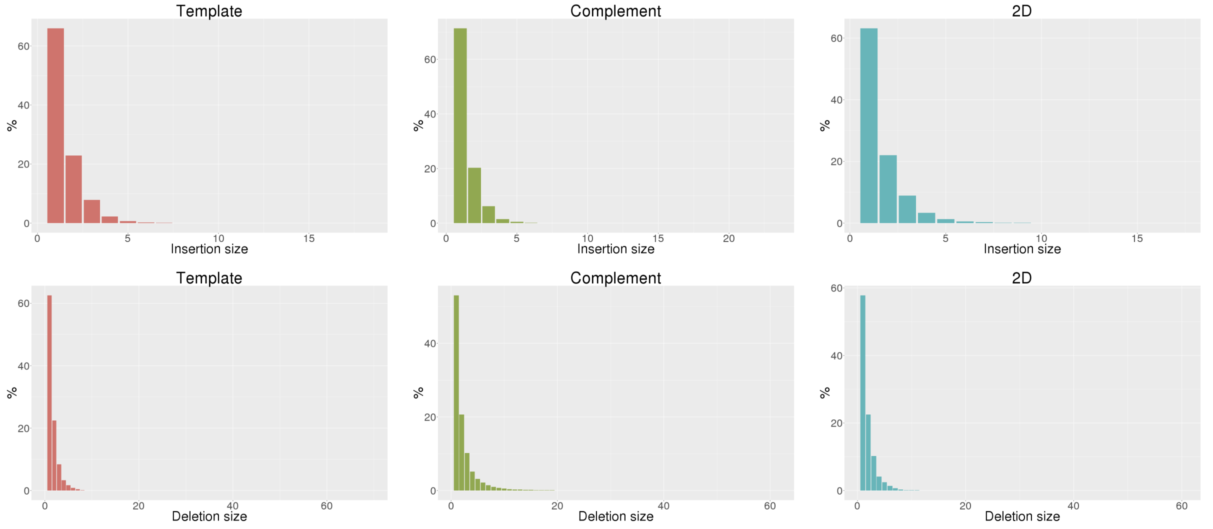

Control sequence read identity

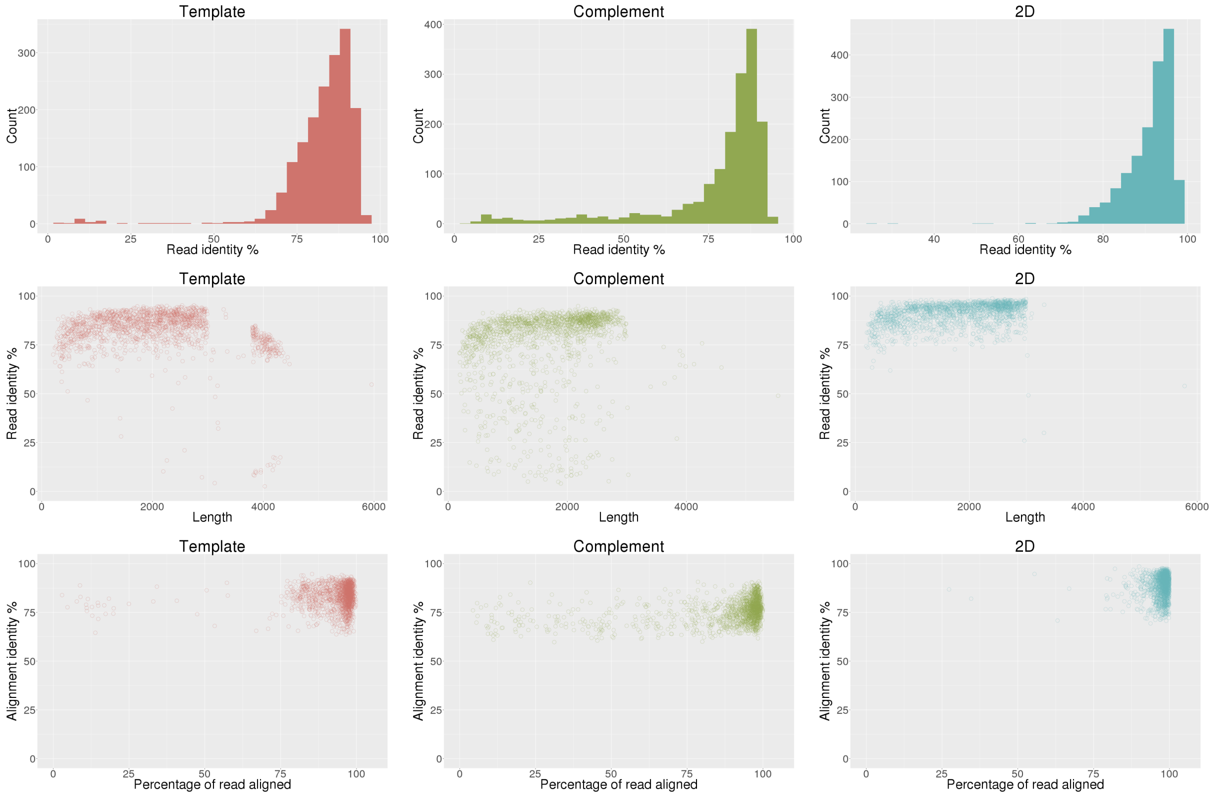

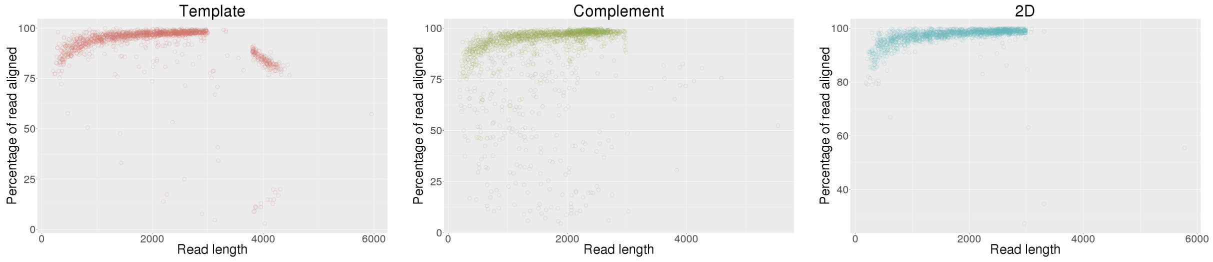

Control sequence perfect kmers

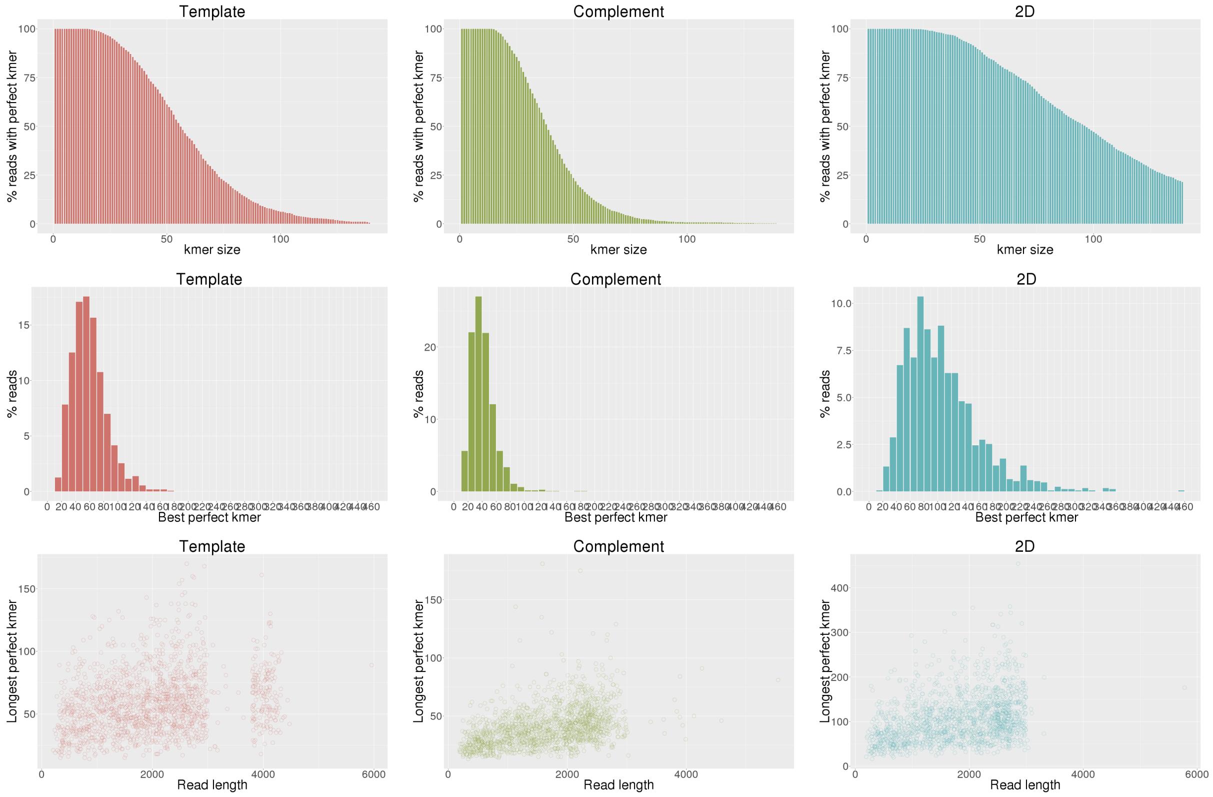

Control sequence coverage

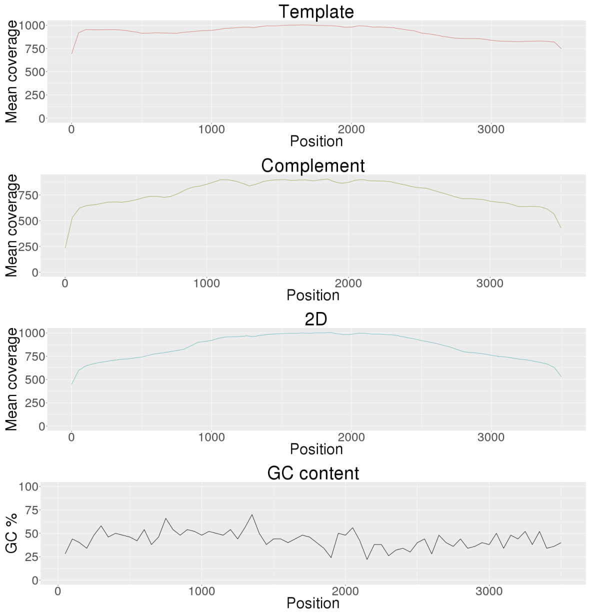

Control sequence 5-mer analysis

Under-represented 5-mers

	Template				Complement				2D			
Rank	kmer	Ref %	Read %	Diff %	kmer	Ref %	Read %	Diff %	kmer	Ref %	Read %	Diff %
1	TTTTT	0.759	0.103	-0.656	TTTTT	0.759	0.118	-0.641	TTTTT	0.759	0.210	-0.549
2	AAAAA	0.478	0.178	-0.300	AAAAA	0.478	0.170	-0.307	AAAAA	0.478	0.216	-0.261
3	TGATG	0.393	0.206	-0.187	CTGAT	0.309	0.170	-0.140	TGATG	0.393	0.233	-0.161
4	GCAAT	0.309	0.158	-0.151	TGATG	0.393	0.258	-0.135	CTGAT	0.309	0.156	-0.153
5	CTGAT	0.309	0.165	-0.144	GAGCA	0.253	0.118	-0.135	GATGT	0.309	0.172	-0.137
6	GATGT	0.309	0.168	-0.142	AGTAG	0.169	0.034	-0.134	GCTGA	0.281	0.151	-0.130
7	GATGC	0.281	0.141	-0.140	CTTTT	0.253	0.122	-0.131	TGTGA	0.225	0.104	-0.120
8	GCTGA	0.281	0.148	-0.133	GATGT	0.309	0.184	-0.125	TGGTT	0.253	0.135	-0.118
9	TTATC	0.309	0.181	-0.128	AGTAA	0.253	0.132	-0.121	GCAAT	0.309	0.191	-0.118
10	GTTTT	0.281	0.153	-0.127	GATGC	0.281	0.165	-0.116	AGATG	0.197	0.088	-0.109

Over-represented 5-mers

	Template				Complement				2D			
Rank	kmer	Ref %	Read %	Diff %	kmer	Ref %	Read %	Diff %	kmer	Ref %	Read %	Diff %
1	CATCA	0.112	0.287	0.174	CAGCA	0.056	0.226	0.170	CATCA	0.112	0.285	0.173
2	ATCAG	0.056	0.192	0.136	CATCA	0.112	0.262	0.150	TCAGC	0.028	0.177	0.149
3	CAGCA	0.056	0.188	0.131	AACCA	0.028	0.161	0.133	ATCAG	0.056	0.193	0.136
4	TCAGC	0.028	0.154	0.126	GCGAA	0.028	0.159	0.131	AACCA	0.028	0.159	0.131
5	CATCT	0.000	0.124	0.124	ATCAG	0.056	0.183	0.126	CAGCA	0.056	0.179	0.122
6	CGTTC	0.028	0.147	0.119	TCAGC	0.028	0.154	0.126	GCATC	0.084	0.205	0.121
7	GTTTA	0.084	0.201	0.117	ATAAA	0.112	0.237	0.125	CATCT	0.000	0.114	0.114
8	GCATC	0.084	0.201	0.116	AATAA	0.197	0.314	0.117	TCACA	0.028	0.130	0.102
9	TACTT	0.000	0.115	0.115	AAACG	0.112	0.228	0.115	GACGA	0.000	0.098	0.098
10	AACCA	0.028	0.142	0.114	CGGCG	0.028	0.132	0.104	AAAAG	0.084	0.182	0.098

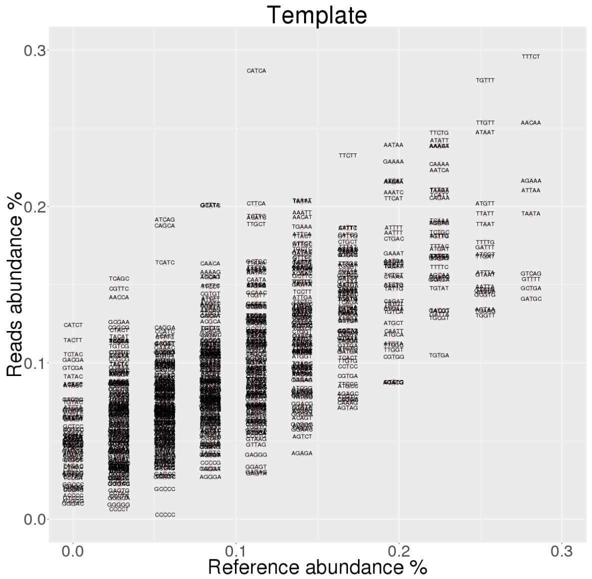

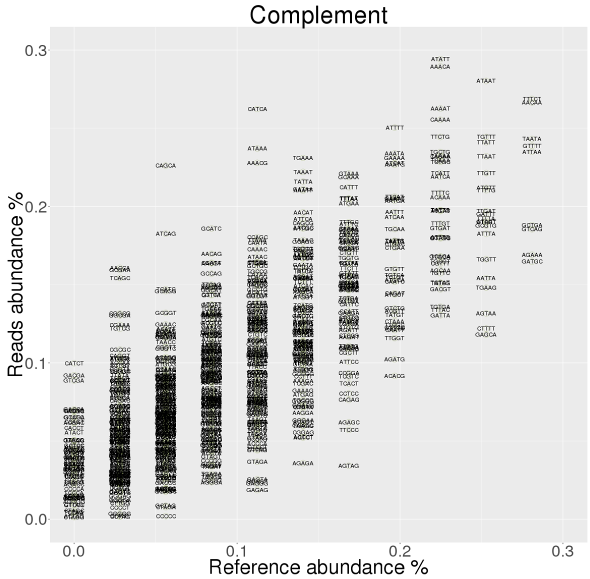

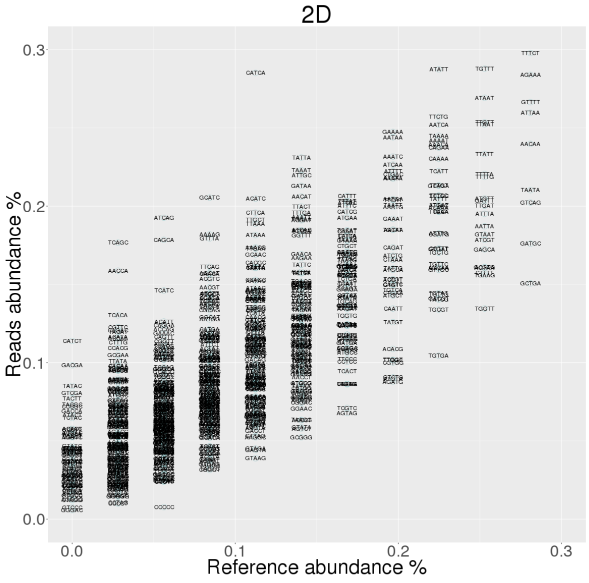

Control sequence GC content

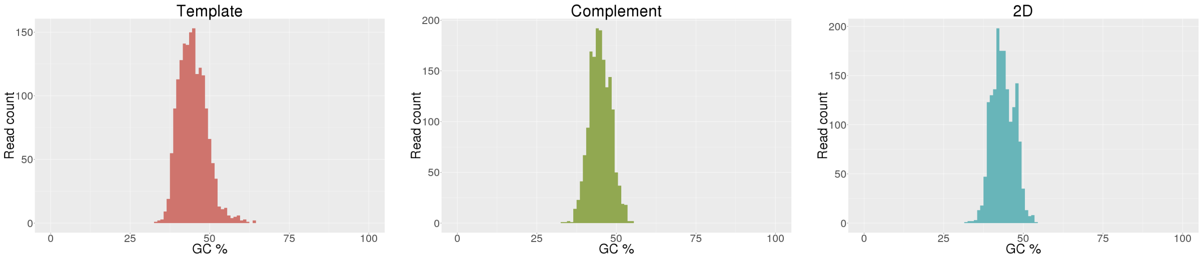

Escherichia coli error analysis

	Template	Complement	2D
Overall base identity (excluding indels)	85.58%	80.38%	92.51%
Aligned base identity (excluding indels)	91.49%	91.17%	95.91%
Identical bases per 100 aligned bases (including indels)	82.65%	76.45%	88.97%
Inserted bases per 100 aligned bases (including indels)	2.43%	1.93%	2.11%
Deleted bases per 100 aligned bases (including indels)	7.24%	14.22%	5.13%
Substitutions per 100 aligned bases (including indels)	7.69%	7.40%	3.80%
Mean insertion size	1.50	1.41	1.63
Mean deletion size	1.64	2.40	1.79

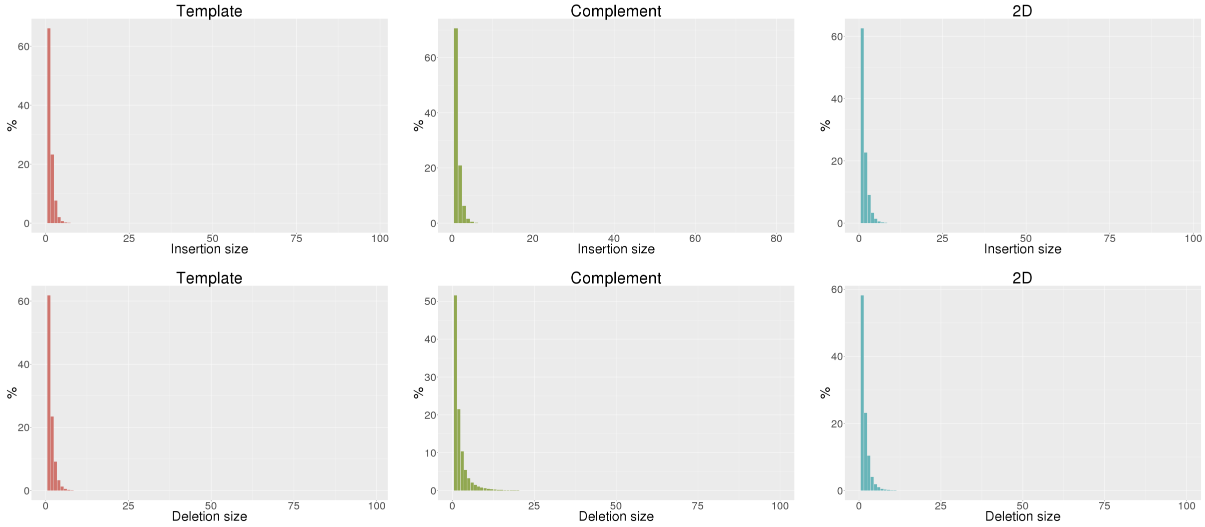

Escherichia coli read identity

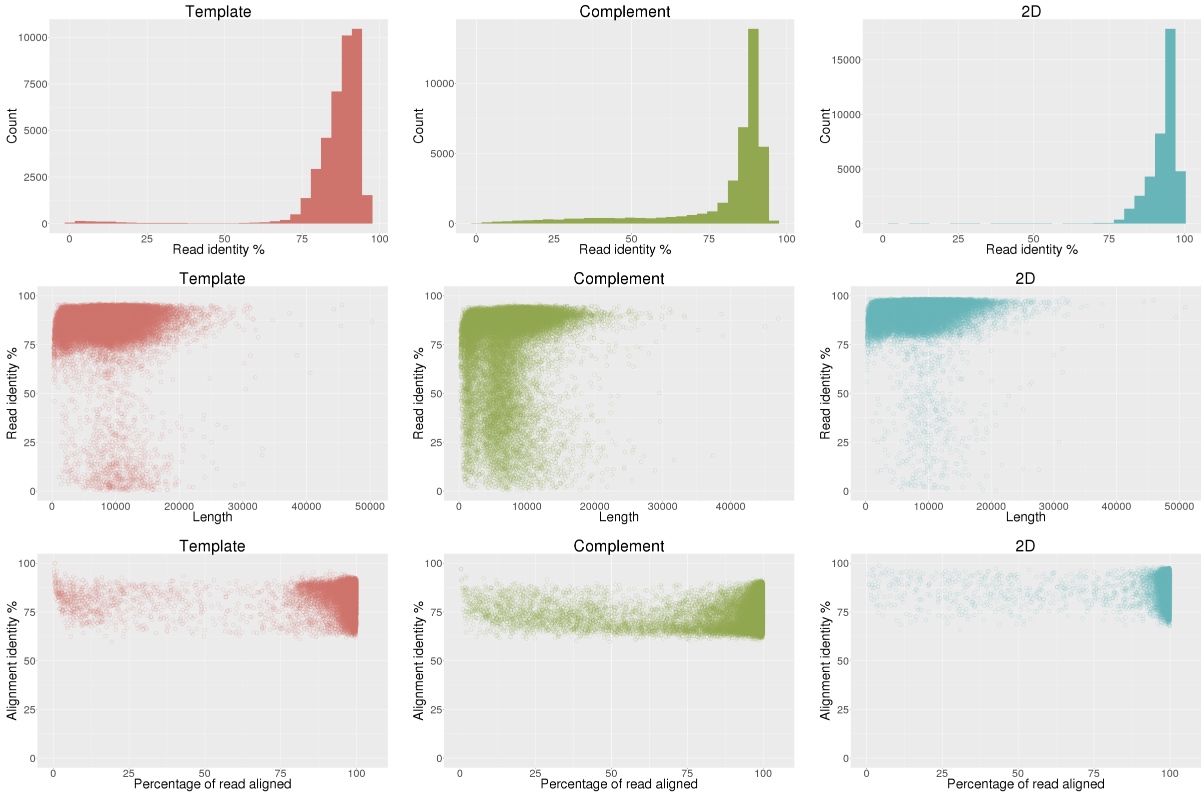

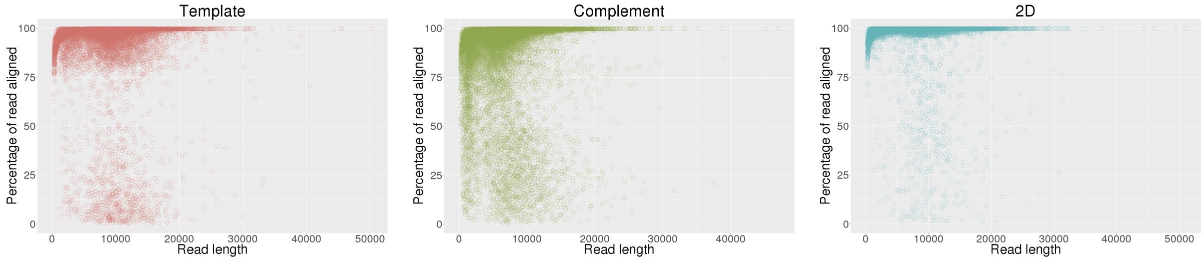

Escherichia coli perfect kmers

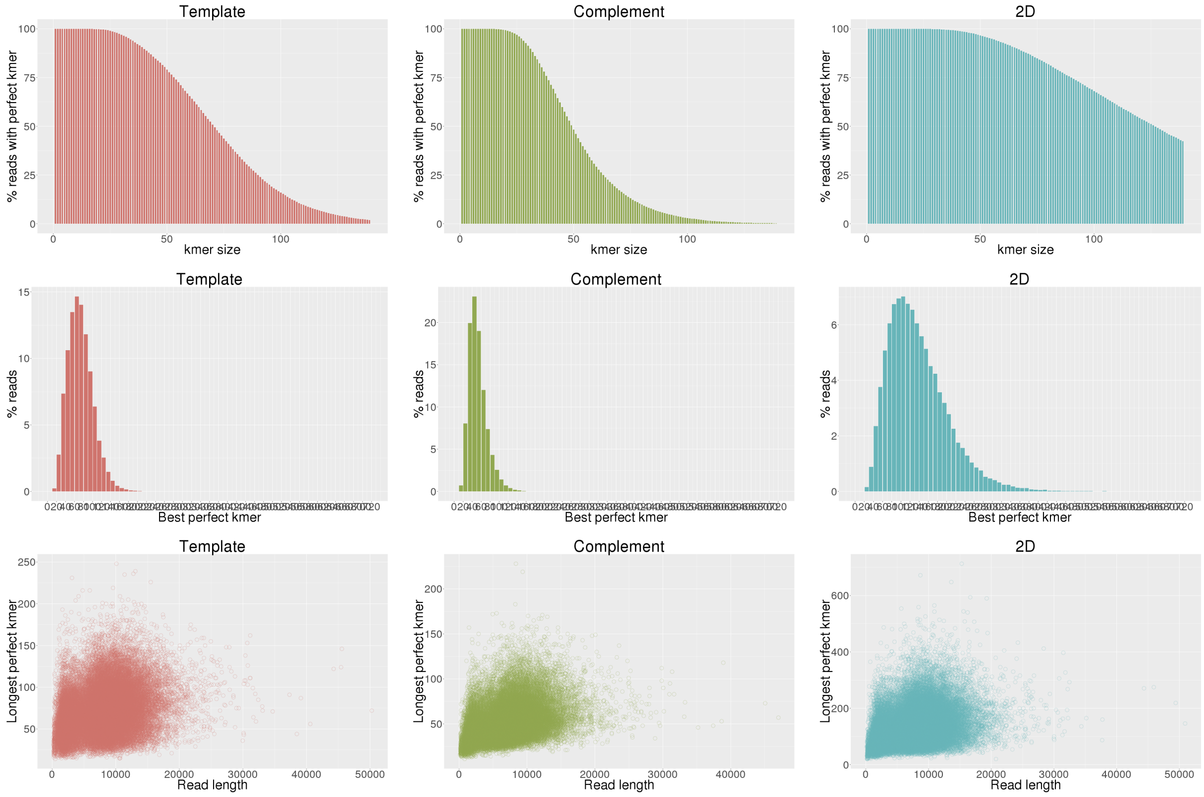

Escherichia coli coverage

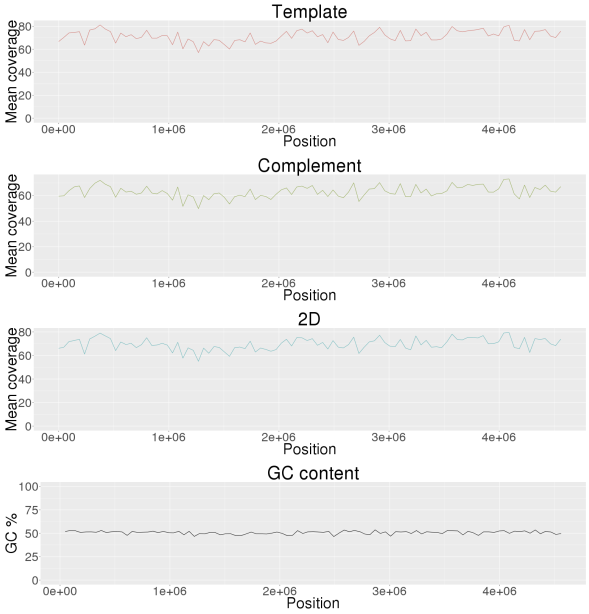

Escherichia coli 5-mer analysis

Under-represented 5-mers

	Template				Complement				2D			
Rank	kmer	Ref %	Read %	Diff %	kmer	Ref %	Read %	Diff %	kmer	Ref %	Read %	Diff %
1	TTTTT	0.251	0.041	-0.210	TTTTT	0.251	0.065	-0.185	TTTTT	0.251	0.134	-0.117
2	AAAAA	0.247	0.106	-0.141	AAAAA	0.247	0.106	-0.141	AAAAA	0.247	0.151	-0.096
3	GTTTT	0.186	0.095	-0.091	CCTGG	0.130	0.063	-0.067	CCTGG	0.130	0.039	-0.091
4	GCCAG	0.280	0.191	-0.088	CCAGG	0.129	0.064	-0.065	CCAGG	0.129	0.046	-0.083
5	CCTGG	0.130	0.057	-0.073	CCCAG	0.111	0.052	-0.059	GCCAG	0.280	0.230	-0.050
6	CCAGG	0.129	0.063	-0.067	TCCAG	0.171	0.118	-0.053	GCCTG	0.185	0.150	-0.035
7	TTTTG	0.172	0.111	-0.061	AAAGA	0.132	0.082	-0.050	CAGGC	0.183	0.149	-0.034
8	TTTTA	0.147	0.088	-0.059	GAAAG	0.130	0.083	-0.048	CCCAG	0.111	0.082	-0.028
9	AAAGA	0.132	0.074	-0.057	AGAAA	0.140	0.094	-0.046	GTTTT	0.186	0.159	-0.028
10	TTTTC	0.198	0.141	-0.057	ATCCC	0.086	0.042	-0.044	CCCTG	0.093	0.066	-0.026

Over-represented 5-mers

	Template				Complement				2D			
Rank	kmer	Ref %	Read %	Diff %	kmer	Ref %	Read %	Diff %	kmer	Ref %	Read %	Diff %
1	CGGCG	0.221	0.335	0.114	CGGCG	0.221	0.284	0.063	CGGTG	0.160	0.201	0.041
2	GCGGC	0.210	0.296	0.086	GCGGC	0.210	0.264	0.054	CACGG	0.089	0.127	0.038
3	CGGTG	0.160	0.244	0.084	AACAA	0.132	0.177	0.046	CGGCG	0.221	0.257	0.036
4	GGCGG	0.190	0.267	0.077	GGCGG	0.190	0.235	0.045	CGCCG	0.219	0.247	0.028
5	CGCCG	0.219	0.287	0.068	CGGTG	0.160	0.204	0.044	GCTGG	0.279	0.305	0.026
6	GGCCG	0.069	0.134	0.065	TGCCG	0.220	0.263	0.043	CACCG	0.166	0.191	0.025
7	CACGG	0.089	0.149	0.060	AAACA	0.138	0.181	0.042	CCGGC	0.141	0.165	0.024
8	GTACG	0.071	0.132	0.060	AATAA	0.148	0.188	0.040	TGCTG	0.250	0.273	0.023
9	CGCGC	0.201	0.260	0.059	GCAAA	0.196	0.235	0.039	ACGGC	0.136	0.159	0.023
10	CGCGG	0.137	0.195	0.057	CGCCG	0.219	0.258	0.039	GCGGT	0.164	0.187	0.023

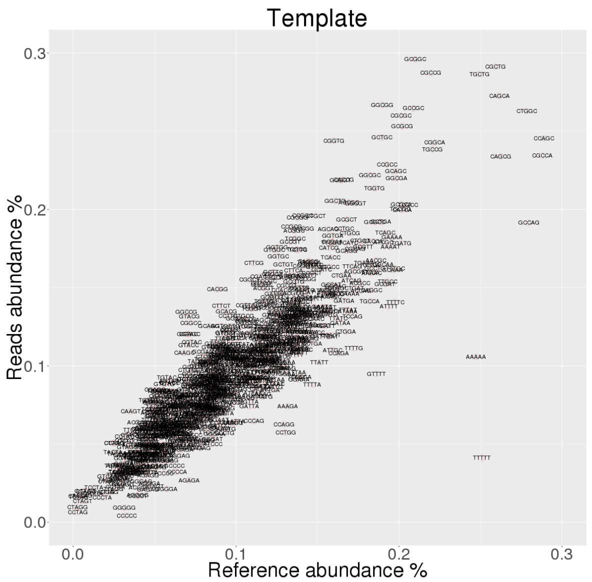

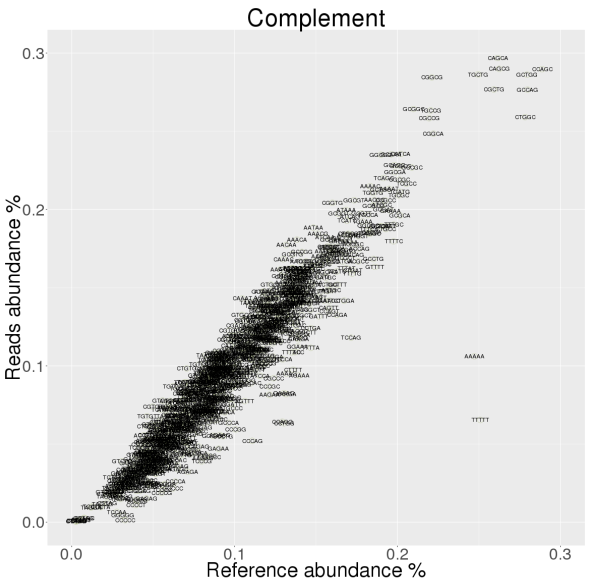

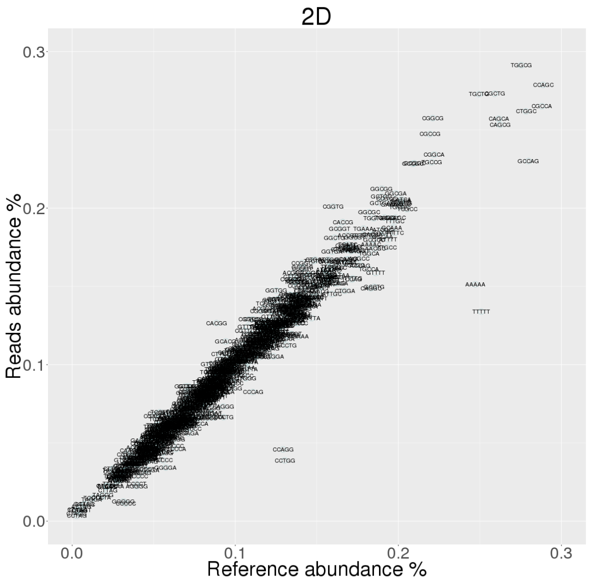

Escherichia coli GC content

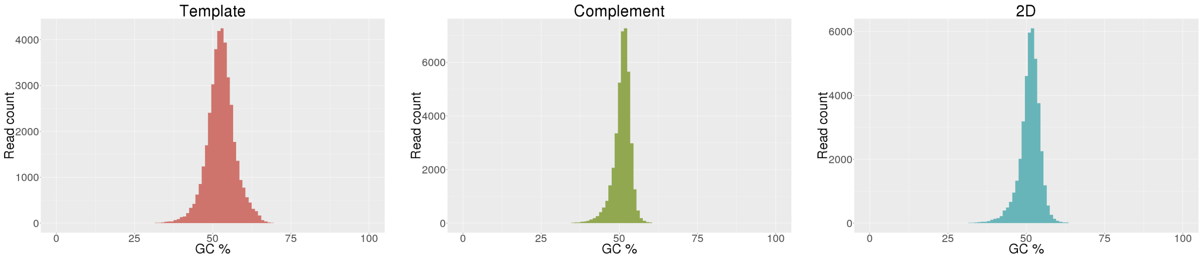

All reference 21mer analysis

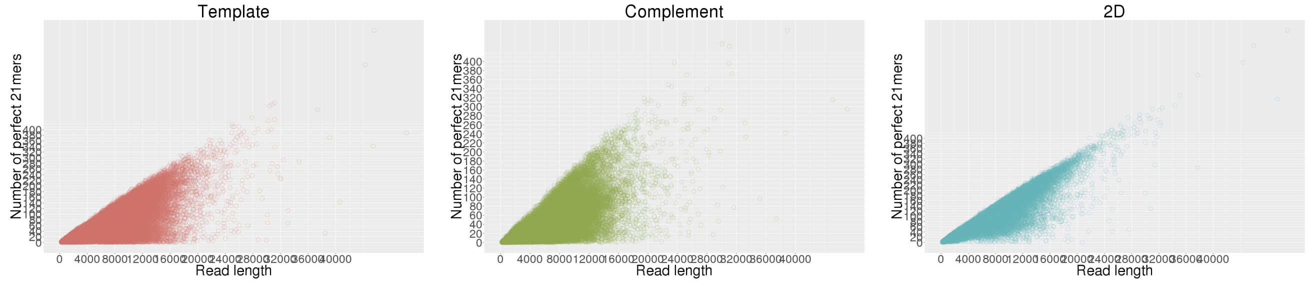

All reference substitutions

		Template substituted %				Complement substituted %				2D substituted %			
		a	c	g	t	a	c	g	t	a	c	g	t
Reference	A	0.00	7.53	16.58	4.70	0.00	5.01	14.80	5.33	0.00	6.65	14.86	5.41
	C	3.85	0.00	3.85	13.11	5.56	0.00	3.91	15.35	5.38	0.00	4.20	13.63
	G	12.77	4.11	0.00	3.78	14.89	4.02	0.00	5.41	13.08	4.34	0.00	5.14
	T	4.72	17.18	7.81	0.00	5.35	15.15	5.23	0.00	5.47	15.26	6.59	0.00

Kmer motifs before errors

3-mer error motif analysis

	Template			Complement			2D			
Rank	Insertion	Deletion	Substitution	Insertion	Deletion	Substitution	Insertion	Deletion	Substitution	
1	TTC (3.38%)	CGC (3.25%)	CGC (3.90%)	CGC (3.28%)	CGC (3.09%)	GCC (3.41%)	CGC (3.09%)	TTT (3.00%)	GCC (4.47%)	Most common
2	CGC (2.97%)	TTC (3.02%)	GCC (3.61%)	ATC (3.00%)	TTT (2.85%)	CGC (3.23%)	TTC (3.05%)	CGC (2.92%)	ACC (3.60%)	
3	ATC (2.91%)	GCC (2.87%)	ATC (3.22%)	TTC (2.71%)	ATC (2.80%)	ATC (3.15%)	GCC (2.81%)	GCC (2.87%)	CGC (3.55%)	
4	TTT (2.68%)	ATC (2.85%)	ACC (3.22%)	TTT (2.69%)	GCC (2.58%)	TGC (2.82%)	TTT (2.69%)	ATC (2.80%)	ATC (3.18%)	
5	GCC (2.64%)	TTT (2.81%)	TGC (3.13%)	GCG (2.57%)	GGC (2.58%)	AAC (2.81%)	ATC (2.68%)	TTC (2.65%)	TGC (3.16%)	
6	ACC (2.41%)	GGC (2.68%)	TTC (2.99%)	GGC (2.56%)	GCG (2.54%)	ACC (2.74%)	TGC (2.67%)	GGC (2.59%)	TTT (3.08%)	
7	TGC (2.38%)	GCG (2.63%)	GGC (2.91%)	TGC (2.50%)	TTC (2.49%)	TTT (2.67%)	GGC (2.49%)	ACC (2.57%)	TTC (2.94%)	
8	GCG (2.24%)	ACC (2.54%)	AAC (2.89%)	GCC (2.47%)	GAT (2.31%)	TTC (2.64%)	ACC (2.40%)	ATT (2.25%)	TCC (2.91%)	
9	GGC (2.22%)	TGC (2.20%)	AGC (2.61%)	AAC (2.32%)	TGC (2.30%)	GGC (2.50%)	AGC (2.25%)	GCG (2.25%)	GGC (2.65%)	
10	AAC (2.16%)	AGC (2.13%)	TTT (2.48%)	GAT (2.22%)	AAA (2.26%)	GCG (2.40%)	GCG (2.10%)	TGC (2.20%)	AAC (2.60%)	
	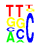	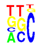	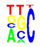	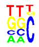	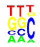	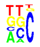	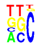	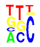	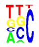	
-10	TGG (1.10%)	AGA (0.98%)	TGA (0.88%)	AGA (0.95%)	GGA (0.92%)	TGT (0.84%)	TAA (1.01%)	AGA (0.97%)	TAT (0.83%)	Least common
-9	TAA (0.92%)	TAA (0.87%)	GTA (0.86%)	CCC (0.95%)	CCC (0.90%)	TCT (0.83%)	GTA (1.00%)	GAG (0.90%)	GTA (0.83%)	
-8	AGT (0.91%)	GTA (0.87%)	CCT (0.85%)	CCT (0.95%)	GTA (0.81%)	GAG (0.79%)	GAG (0.96%)	AGT (0.90%)	CGA (0.82%)	
-7	GAG (0.88%)	AGT (0.85%)	GAG (0.82%)	GTA (0.84%)	GAG (0.80%)	ACT (0.67%)	AGT (0.95%)	GTA (0.87%)	GAG (0.78%)	
-6	AGA (0.81%)	GGA (0.83%)	GGG (0.63%)	GGA (0.79%)	AGG (0.77%)	GGG (0.65%)	AGA (0.78%)	GGA (0.86%)	GGG (0.73%)	
-5	GGA (0.78%)	AGG (0.76%)	AGA (0.63%)	AGG (0.78%)	AGT (0.72%)	GGA (0.64%)	AGG (0.75%)	CCT (0.75%)	AGA (0.64%)	
-4	AGG (0.74%)	CCT (0.70%)	AGT (0.60%)	AGT (0.76%)	CCT (0.64%)	CCT (0.58%)	GGA (0.72%)	AGG (0.73%)	AGT (0.55%)	
-3	GGG (0.50%)	GGG (0.61%)	GGA (0.56%)	GGG (0.52%)	GGG (0.56%)	AGT (0.49%)	GGG (0.58%)	GGG (0.61%)	GGA (0.52%)	
-2	CTA (0.46%)	TAG (0.41%)	TAG (0.43%)	TAG (0.36%)	TAG (0.33%)	TAG (0.39%)	TAG (0.44%)	CTA (0.41%)	TAG (0.39%)	
-1	TAG (0.45%)	CTA (0.37%)	CTA (0.30%)	CTA (0.28%)	CTA (0.27%)	CTA (0.27%)	CTA (0.41%)	TAG (0.40%)	CTA (0.32%)	
	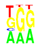	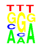	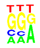	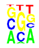	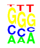	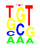	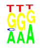	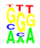	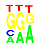	

Kmer space for 3-mers: 64 Random chance for any given 3-mer: 1.56%

4-mer error motif analysis

Rank	Template			Complement			2D			
	Insertion	Deletion	Substitution	Insertion	Deletion	Substitution	Insertion	Deletion	Substitution	
1	TTTC (1.18%)	CGCC (1.21%)	CGCC (1.54%)	CAGC (1.12%)	CAGC (1.12%)	CGCC (1.44%)	CGCC (1.14%)	CGCC (1.17%)	CGCC (1.82%)	Most common
2	CGCC (1.12%)	GCGC (1.13%)	GCGC (1.23%)	CGCC (1.07%)	CGCC (1.11%)	CAGC (1.13%)	CAGC (1.01%)	CAGC (1.00%)	TGCC (1.23%)	
3	GCGC (0.99%)	CAGC (1.04%)	CAGC (1.22%)	GCGC (1.05%)	GCGC (1.05%)	TGCC (1.02%)	TTTC (1.00%)	GCGC (1.00%)	CACC (1.10%)	
4	CAGC (0.93%)	CGGC (1.01%)	CGGC (1.06%)	CGGC (0.94%)	CGGC (0.94%)	GCGC (1.00%)	GCGC (0.98%)	CGGC (0.93%)	GCGC (1.10%)	
5	CTTC (0.87%)	TTTC (1.01%)	CATC (1.02%)	CATC (0.89%)	TGGC (0.89%)	CATC (0.95%)	TGGC (0.86%)	TTTC (0.85%)	CAGC (1.08%)	
6	CATC (0.86%)	CATC (0.91%)	CACC (1.02%)	TGGC (0.89%)	CATC (0.87%)	AAAC (0.92%)	CGGC (0.84%)	CACC (0.85%)	AACC (1.07%)	
7	GCTG (0.81%)	GGCG (0.90%)	ACGC (0.95%)	TTTC (0.88%)	TTTC (0.86%)	TGGC (0.89%)	CTTC (0.81%)	ATTT (0.84%)	ATCC (1.05%)	
8	CACC (0.80%)	CACC (0.85%)	TGGC (0.94%)	ACGC (0.83%)	GGCG (0.84%)	CGGC (0.88%)	CTGC (0.79%)	CATC (0.84%)	TTCC (1.02%)	
9	CGGC (0.80%)	TGGC (0.81%)	TGCC (0.93%)	GGCG (0.82%)	TTTT (0.80%)	AACC (0.86%)	TGCC (0.79%)	TTTT (0.83%)	TTTT (0.99%)	
10	TTTT (0.75%)	GTTT (0.81%)	CCGC (0.92%)	GCTG (0.78%)	ATTT (0.79%)	ATCA (0.84%)	CATC (0.77%)	GTTT (0.81%)	AGCC (0.94%)	
	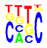	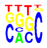	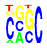	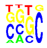	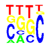	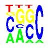	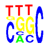	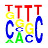	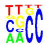	
-10	TTAG (0.12%)	CTTA (0.11%)	TAGT (0.09%)	GGGA (0.10%)	TTAG (0.09%)	GGGA (0.08%)	AGGG (0.11%)	TTAG (0.11%)	CTAA (0.09%)	Least common
-9	TCTA (0.10%)	TTAG (0.10%)	ACTA (0.09%)	TAGT (0.09%)	CTAA (0.09%)	GAGT (0.07%)	ACTA (0.11%)	TAGA (0.11%)	GAGT (0.09%)	
-8	GGGA (0.10%)	ACTA (0.10%)	GGGA (0.09%)	CTAA (0.09%)	CCCT (0.09%)	ACTA (0.07%)	GGGA (0.10%)	CCCT (0.11%)	ACTA (0.08%)	
-7	AGGG (0.10%)	TCTA (0.09%)	GGGG (0.08%)	TAGA (0.09%)	TAGT (0.08%)	TAGG (0.07%)	GGGG (0.10%)	ACTA (0.10%)	GGGA (0.08%)	
-6	CTAA (0.09%)	CCCT (0.09%)	GAGT (0.08%)	ACTA (0.07%)	ACTA (0.07%)	TAGA (0.07%)	CTAA (0.09%)	CTAA (0.10%)	TAGG (0.07%)	
-5	TAGA (0.08%)	GGGG (0.09%)	TAGG (0.08%)	TCTA (0.07%)	GGGG (0.07%)	GGGG (0.07%)	TCTA (0.09%)	TCTA (0.09%)	TAGT (0.07%)	
-4	GGGG (0.07%)	CTAA (0.09%)	TCTA (0.06%)	GGGG (0.07%)	TCTA (0.06%)	TAGT (0.05%)	TAGA (0.08%)	GGGG (0.09%)	TCTA (0.06%)	
-3	CCTA (0.07%)	TAGG (0.06%)	TAGA (0.06%)	TAGG (0.05%)	TAGG (0.05%)	TCTA (0.05%)	TAGG (0.06%)	TAGG (0.06%)	TAGA (0.05%)	
-2	TAGG (0.07%)	CCTA (0.05%)	CCTA (0.04%)	CCTA (0.04%)	CCTA (0.03%)	CCTA (0.03%)	CCTA (0.06%)	CCTA (0.06%)	CCTA (0.05%)	
-1	CTAG (0.01%)	CTAG (0.01%)	CTAG (0.01%)	CTAG (0.00%)	CTAG (0.00%)	CTAG (0.00%)	CTAG (0.01%)	CTAG (0.01%)	CTAG (0.01%)	
	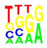	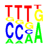	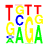	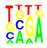	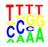	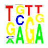	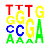	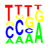	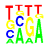	

Kmer space for 4-mers: 256 Random chance for any given 4-mer: 0.39%

5-mer error motif analysis

Rank	Template			Complement			2D			
	Insertion	Deletion	Substitution	Insertion	Deletion	Substitution	Insertion	Deletion	Substitution	
1	ATTTC (0.36%)	GCGGC (0.37%)	CCAGC (0.47%)	CCAGC (0.43%)	CCAGC (0.43%)	CCAGC (0.44%)	CCAGC (0.36%)	CCAGC (0.36%)	CCGCC (0.51%)	Most common
2	CCAGC (0.35%)	CCAGC (0.37%)	CCGCC (0.42%)	CTGGC (0.36%)	CTGGC (0.38%)	TCGCC (0.42%)	CTGGC (0.34%)	CGCCA (0.33%)	TCGCC (0.50%)	
3	CGCCA (0.34%)	GGCGC (0.34%)	TCGCC (0.41%)	GCGGC (0.33%)	GCGGC (0.35%)	CTGGC (0.38%)	CGCCA (0.33%)	GCGGC (0.33%)	CAGCC (0.46%)	
4	GTTTC (0.34%)	TCGCC (0.33%)	CTGGC (0.40%)	TGGCG (0.32%)	CGCCA (0.34%)	CCGCC (0.36%)	TCGCC (0.32%)	TCGCC (0.33%)	GCGCC (0.44%)	
5	TCGCC (0.30%)	CCGCC (0.32%)	GCGGC (0.38%)	CGCCA (0.32%)	TGGCG (0.33%)	GCGCC (0.36%)	GCGGC (0.30%)	CTGGC (0.32%)	ACGCC (0.42%)	
6	CGCGC (0.30%)	TGGCG (0.32%)	GCGCC (0.38%)	CAGCG (0.31%)	TCGCC (0.31%)	CGCCA (0.35%)	CCGCC (0.29%)	CCGCC (0.31%)	CCAGC (0.41%)	
7	CCGCC (0.29%)	CTGGC (0.32%)	ACGCC (0.36%)	CGCGC (0.31%)	CGCGC (0.29%)	ACGCC (0.33%)	ATTTC (0.29%)	GGCGC (0.31%)	TTGCC (0.39%)	
8	GCGGC (0.29%)	CGCGC (0.29%)	CAGCC (0.35%)	AACGC (0.30%)	CCGCC (0.29%)	GCGGC (0.33%)	ACGCC (0.28%)	ACGCC (0.28%)	CTGCC (0.35%)	
9	CTGGC (0.28%)	TTTTC (0.29%)	GCCGC (0.35%)	TCGCC (0.29%)	GGCGC (0.29%)	TTGCC (0.32%)	GTTTC (0.27%)	TCACC (0.27%)	CTGGC (0.35%)	
10	TTTTC (0.28%)	ACGCC (0.29%)	CGCGC (0.35%)	ATTTT (0.29%)	GCTGG (0.28%)	CAGCC (0.32%)	TGCGC (0.27%)	TGGCG (0.27%)	TCACC (0.35%)	
	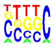	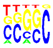	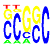	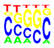	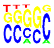	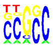	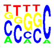	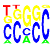	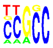	
-10	CCTGG (0.01%)	CCAGG (0.01%)	CTAGC (0.01%)	CCCCC (0.00%)	TCCTA (0.00%)	CCCCC (0.00%)	TCCTA (0.01%)	TCCTA (0.01%)	CTAGC (0.01%)	Least common
-9	CTAGC (0.01%)	GGGGG (0.01%)	GGGGG (0.00%)	GGGGG (0.00%)	GGGGG (0.00%)	GGGGG (0.00%)	TAGGA (0.01%)	TAGGA (0.01%)	TCCTA (0.01%)	
-8	ACTAG (0.00%)	CTAGT (0.00%)	TAGGA (0.00%)	CTAGC (0.00%)	CTAGC (0.00%)	CTAGC (0.00%)	CTAGC (0.01%)	CTAGC (0.00%)	TAGGA (0.00%)	
-7	GGGGG (0.00%)	CTAGC (0.00%)	ACTAG (0.00%)	GCTAG (0.00%)	GCTAG (0.00%)	GCTAG (0.00%)	GCTAG (0.00%)	CTAGT (0.00%)	GCTAG (0.00%)	
-6	GCTAG (0.00%)	GCTAG (0.00%)	CTAGT (0.00%)	CTAGT (0.00%)	CTAGT (0.00%)	ACTAG (0.00%)	CTAGT (0.00%)	GCTAG (0.00%)	ACTAG (0.00%)	
-5	CTAGT (0.00%)	ACTAG (0.00%)	GCTAG (0.00%)	CTAGA (0.00%)	CTAGA (0.00%)	TCTAG (0.00%)	TCTAG (0.00%)	ACTAG (0.00%)	CTAGT (0.00%)	
-4	TCTAG (0.00%)	TCTAG (0.00%)	TCTAG (0.00%)	ACTAG (0.00%)	ACTAG (0.00%)	CTAGT (0.00%)	ACTAG (0.00%)	TCTAG (0.00%)	TCTAG (0.00%)	
-3	CTAGA (0.00%)	CTAGA (0.00%)	CTAGG (0.00%)	TCTAG (0.00%)	CTAGG (0.00%)	CTAGG (0.00%)	CTAGA (0.00%)	CTAGA (0.00%)	CTAGG (0.00%)	
-2	CCTAG (0.00%)	CTAGG (0.00%)	CCTAG (0.00%)	CCTAG (0.00%)	TCTAG (0.00%)	CTAGA (0.00%)	CCTAG (0.00%)	CTAGG (0.00%)	CTAGA (0.00%)	
-1	CTAGG (0.00%)	CCTAG (0.00%)	CTAGA (0.00%)	CTAGG (0.00%)	CCTAG (0.00%)	CCTAG (0.00%)	CTAGG (0.00%)	CCTAG (0.00%)	CCTAG (0.00%)	
	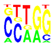	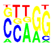	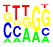	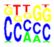	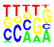	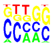	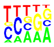	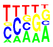	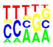	

Kmer space for 5-mers: 1024 Random chance for any given 5-mer: 0.10%
