# Supplementary material for: MinION Analysis and Reference Consortium: Phase 2 data release and analysis of R9.0 chemistry
Source: F1000Res. 2017 May 31;6:760. [Version 1] doi: 10.12688/f1000research.11354.1 (PMC5538040; doi:10.12688/f1000research.11354.1)
Supplement: Supplementary file 3 [file f1000research-6-12257-s0002.tgz › 5d2560fe-dd7a-44d1-b265-e3522e742149.zip!/SI File 3. NanoOK experiment reports/P2-Lab7-R1-1D_nanook_bwa_failonly.pdf]

NanoOK report for Nott_R9_run2_1D

Pass and fail counts

Type	Pass	Fail
Template	0	22074

Read lengths

Type	NumReads	TotalBases	Mean	Longest	Shortest	N50	N50Count	N90	N90Count
Template	22074	134294206	6083.82	353096	5	11991	3112	3157	11416

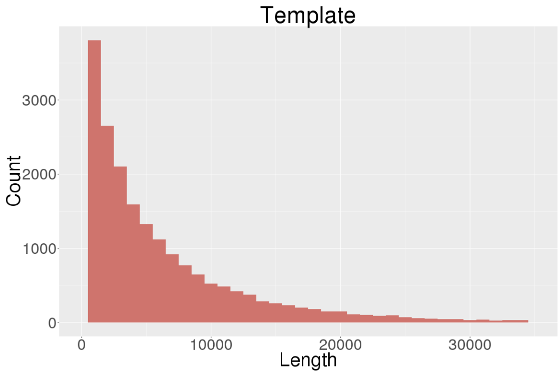

Template alignments

Number of reads	22074	
Number of reads with alignments	12687	(57.47%)
Number of reads without alignments	9387	(42.53%)

ID	Size	Number of Reads	% of Reads	Mean read length	Aligned bases	Mean coverage	Longest Perf Kmer
Control sequence	3560	0	0.00	0.00	0	0.00	0
Escherichia coli	4641652	12687	57.47	8189.66	84716413	18.25	113

Escherichia coli error analysis

	Template
Overall base identity (excluding indels)	55.33%
Aligned base identity (excluding indels)	80.43%
Identical bases per 100 aligned bases (including indels)	67.86%
Inserted bases per 100 aligned bases (including indels)	4.35%
Deleted bases per 100 aligned bases (including indels)	11.28%
Substitutions per 100 aligned bases (including indels)	16.51%
Mean insertion size	1.52
Mean deletion size	1.78

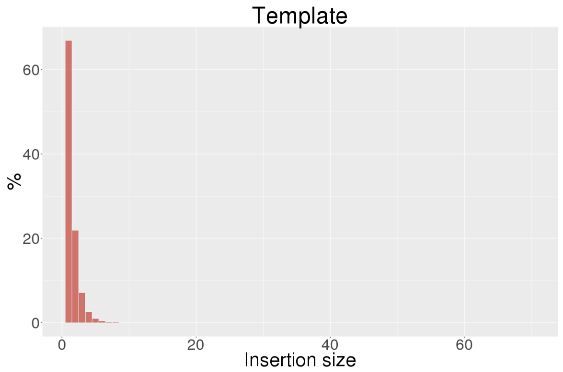

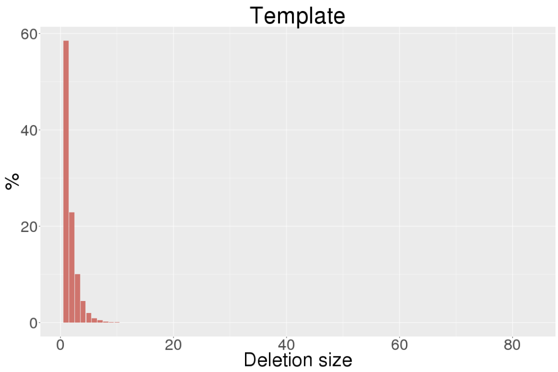

Escherichia coli read identity

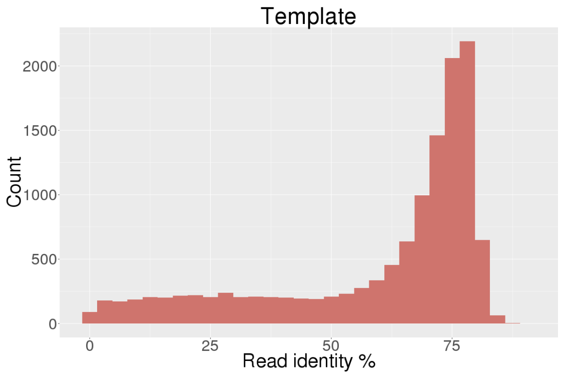

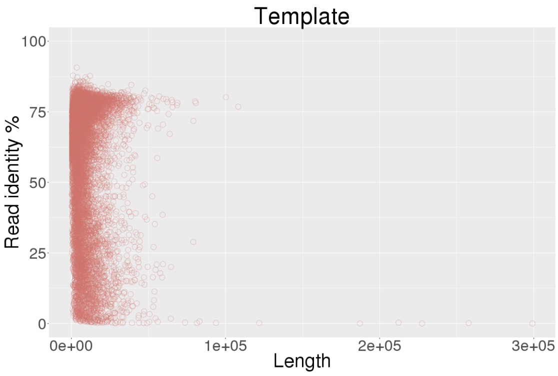

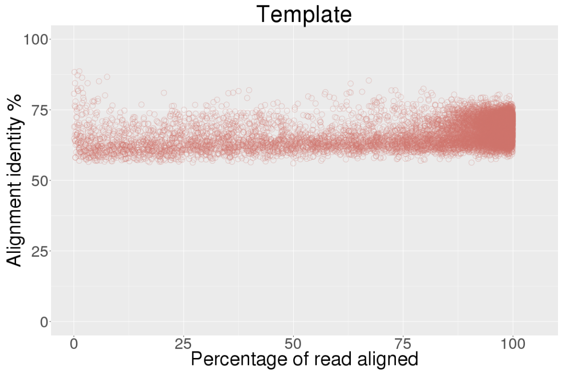

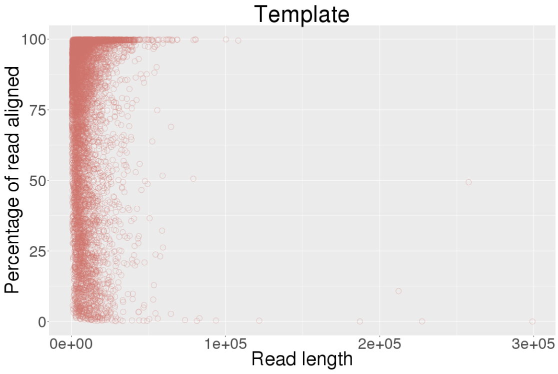

Escherichia coli perfect kmers

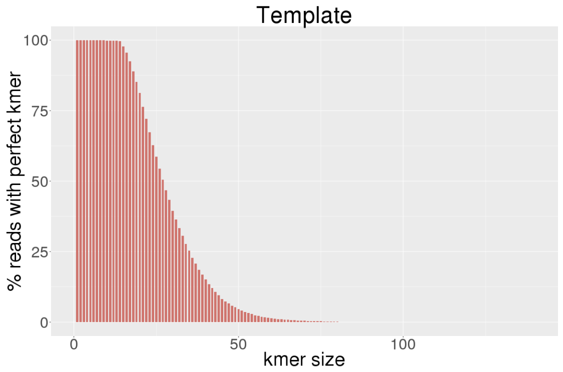

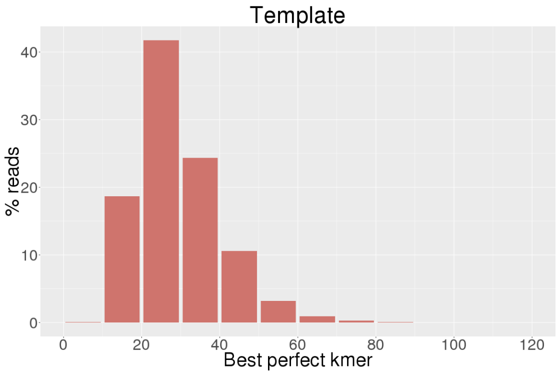

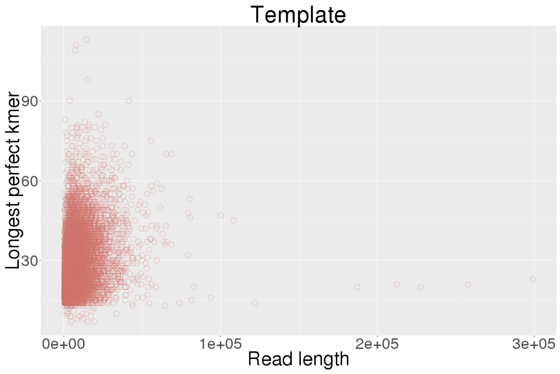

Escherichia coli coverage

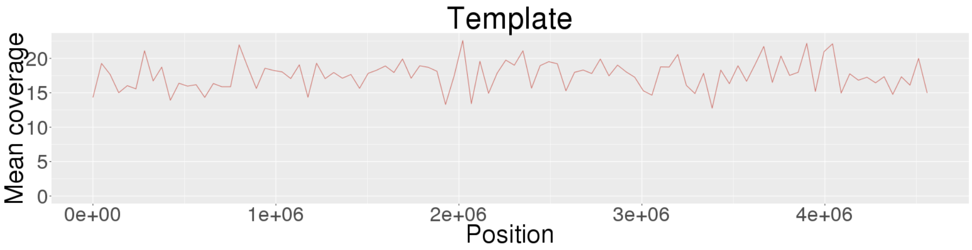

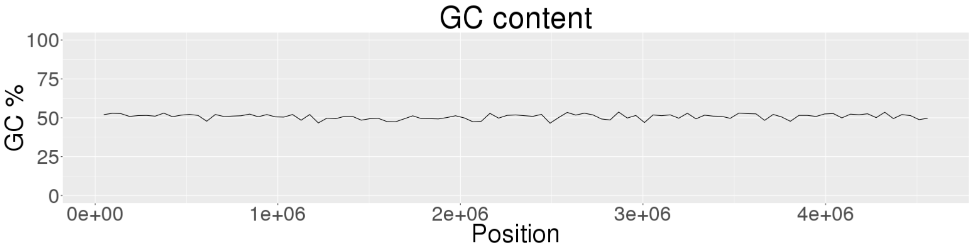

Escherichia coli 5-mer analysis

Under-represented 5-mers

		Template		
Rank	kmer	Ref %	Read %	Diff %
1	TTTTT	0.251	0.022	-0.229
2	AAAAA	0.247	0.041	-0.206
3	GCCAG	0.280	0.142	-0.138
4	ATTTT	0.194	0.056	-0.138
5	GTTTT	0.186	0.055	-0.131
6	TTTAT	0.169	0.043	-0.126
7	TTTTC	0.198	0.072	-0.126
8	TTATT	0.151	0.027	-0.124
9	GATAA	0.164	0.043	-0.122
10	GCAAA	0.196	0.075	-0.121

Over-represented 5-mers

		Template		
Rank	kmer	Ref %	Read %	Diff %
1	CGGCG	0.221	0.841	0.620
2	GCGGC	0.210	0.752	0.542
3	GGCGG	0.190	0.664	0.474
4	CGCCG	0.219	0.671	0.452
5	GCCGC	0.209	0.651	0.442
6	GGCCG	0.069	0.492	0.422
7	CGCGC	0.201	0.587	0.386
8	CCGGC	0.141	0.512	0.371
9	GCGCG	0.202	0.548	0.346
10	CGCGG	0.137	0.482	0.345

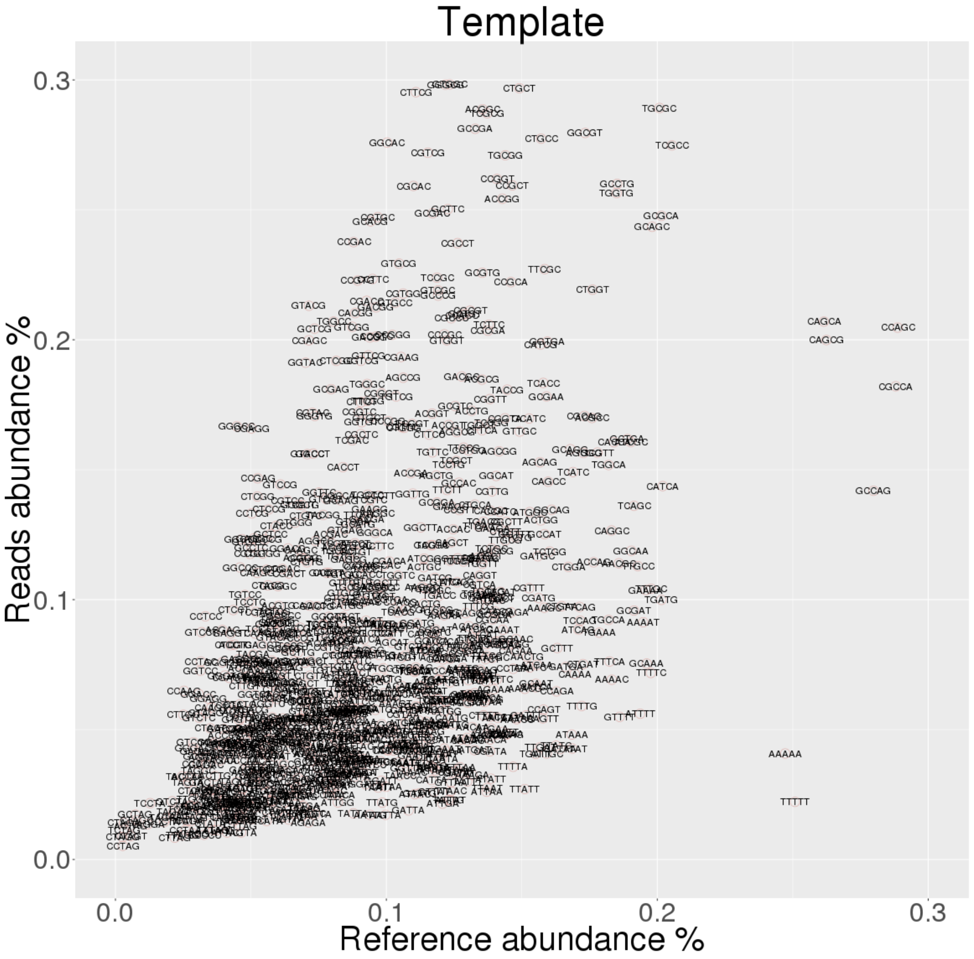

Escherichia coli GC content

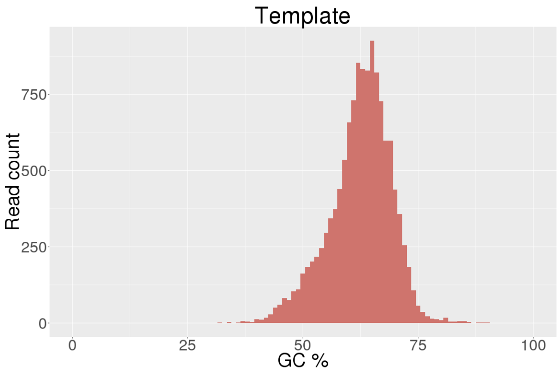

All reference 21mer analysis

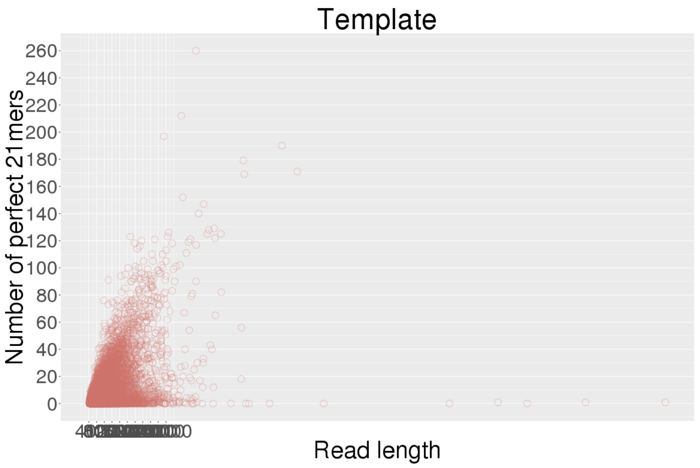

All reference substitutions

		Template substituted %			
		a	c	g	t
Reference	A	0.00	11.12	20.20	4.61
	C	2.67	0.00	4.89	6.75
	G	6.85	5.22	0.00	2.61
	T	4.58	19.76	10.75	0.00

Kmer motifs before errors

3-mer error motif analysis

	Template			
Rank	Insertion	Deletion	Substitution	
1	CGC (3.46%)	CGC (4.56%)	CGC (4.48%)	Most common
2	GCC (3.11%)	GCG (4.22%)	GCC (3.69%)	
3	TTC (2.98%)	GCC (3.70%)	GCG (3.42%)	
4	GCG (2.85%)	GGC (3.48%)	GGC (3.39%)	
5	ATC (2.60%)	TTC (3.23%)	TTC (2.90%)	
6	GGC (2.57%)	ATC (2.92%)	ATC (2.89%)	
7	ACC (2.46%)	ACC (2.88%)	ACC (2.85%)	
8	CCA (2.29%)	CCG (2.53%)	TGC (2.65%)	
9	GCA (2.26%)	CGG (2.38%)	CCG (2.43%)	
10	TTT (2.24%)	TGC (2.35%)	CTG (2.39%)	
	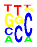	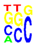	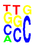	
-10	AGA (0.85%)	TGT (0.77%)	CAT (0.76%)	Least common
-9	AGG (0.81%)	GTA (0.75%)	AGA (0.74%)	
-8	GAG (0.77%)	CCT (0.73%)	ACT (0.68%)	
-7	AGT (0.74%)	AGT (0.58%)	ATA (0.63%)	
-6	TTA (0.69%)	ATA (0.58%)	TAA (0.56%)	
-5	TAT (0.63%)	TTA (0.54%)	AGT (0.54%)	
-4	GGG (0.58%)	TAT (0.49%)	TTA (0.53%)	
-3	TAA (0.53%)	TAA (0.42%)	TAT (0.48%)	
-2	CTA (0.43%)	TAG (0.29%)	TAG (0.31%)	
-1	TAG (0.27%)	CTA (0.26%)	CTA (0.22%)	
	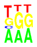	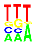	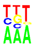	

Kmer space for 3-mers: 64   Random chance for any given 3-mer: 1.56%

4-mer error motif analysis

Rank	Template Insertion	Template Deletion	Template Substitution	
1	CGCC (1.45%)	CGCC (1.76%)	CGCC (1.74%)	Most common
2	GCGC (1.11%)	GCGC (1.60%)	GCGC (1.49%)	
3	GCTG (1.07%)	GGCG (1.59%)	CGGC (1.38%)	
4	CGGC (1.01%)	CGGC (1.46%)	CCGC (1.25%)	
5	GGCG (0.97%)	CCGC (1.28%)	GGCG (1.23%)	
6	CAGC (0.96%)	CAGC (1.18%)	CAGC (1.15%)	
7	CTTC (0.96%)	CACC (1.13%)	GCTG (1.13%)	
8	GCCA (0.94%)	CGCG (1.08%)	CACC (1.08%)	
9	CCGC (0.93%)	CTTC (1.07%)	CATC (1.04%)	
10	CACC (0.91%)	CATC (1.06%)	GCCG (0.96%)	
	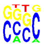	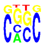	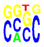	
-10	TAAG (0.08%)	ACTA (0.06%)	ACTA (0.06%)	Least common
-9	CTTA (0.08%)	TATA (0.06%)	TAGT (0.06%)	
-8	CTAT (0.07%)	TAGA (0.06%)	CTAT (0.06%)	
-7	CCTA (0.07%)	CTAT (0.06%)	TAAG (0.06%)	
-6	TAGA (0.07%)	TTAG (0.05%)	CTTA (0.05%)	
-5	ATAG (0.07%)	CTTA (0.05%)	CTAA (0.05%)	
-4	TTAG (0.06%)	TAGG (0.05%)	TCTA (0.05%)	
-3	CTAA (0.05%)	CCTA (0.04%)	TAGA (0.05%)	
-2	TAGG (0.05%)	CTAA (0.04%)	CCTA (0.04%)	
-1	CTAG (0.01%)	CTAG (0.01%)	CTAG (0.01%)	
	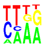	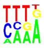	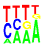	

Kmer space for 4-mers: 256   Random chance for any given 4-mer: 0.39%

5-mer error motif analysis

Rank	Template Insertion	Template Deletion	Template Substitution	
1	CGCCA (0.50%)	CGGCG (0.57%)	CCGCC (0.54%)	Most common
2	CCGCC (0.43%)	GCGGC (0.56%)	GCGGC (0.54%)	
3	CCAGC (0.41%)	CCGCC (0.54%)	CCAGC (0.49%)	
4	GCGGC (0.38%)	GGCGC (0.52%)	GCCGC (0.48%)	
5	CGGCG (0.37%)	CCAGC (0.49%)	GGCGC (0.47%)	
6	CGCGC (0.36%)	TGGCG (0.47%)	CGGCG (0.47%)	
7	CGCTG (0.36%)	GCCGC (0.47%)	CGCCG (0.43%)	
8	GCCGC (0.35%)	CGCGC (0.45%)	TCGCC (0.43%)	
9	TCGCC (0.34%)	CGCCG (0.45%)	CGCGC (0.42%)	
10	GCGCC (0.34%)	TCGCC (0.44%)	CTGGC (0.41%)	
	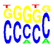	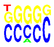	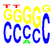	
-10	CTTAG (0.01%)	CCTAA (0.01%)	TCCTA (0.00%)	Least common
-9	CCTAA (0.01%)	TAGGA (0.00%)	TAGGA (0.00%)	
-8	TAGGA (0.01%)	GCTAG (0.00%)	CTAGC (0.00%)	
-7	CTAGC (0.00%)	CTAGC (0.00%)	GCTAG (0.00%)	
-6	ACTAG (0.00%)	TCTAG (0.00%)	TCTAG (0.00%)	
-5	GCTAG (0.00%)	CTAGT (0.00%)	CTAGT (0.00%)	
-4	CTAGT (0.00%)	ACTAG (0.00%)	ACTAG (0.00%)	
-3	CTAGA (0.00%)	CTAGA (0.00%)	CCTAG (0.00%)	
-2	TCTAG (0.00%)	CCTAG (0.00%)	CTAGG (0.00%)	
-1	CCTAG (0.00%)	CTAGG (0.00%)	CTAGA (0.00%)	
	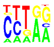	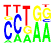	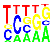	

Kmer space for 5-mers: 1024   Random chance for any given 5-mer: 0.10%
