# Supplementary material for: MinION Analysis and Reference Consortium: Phase 2 data release and analysis of R9.0 chemistry
Source: F1000Res. 2017 May 31;6:760. [Version 1] doi: 10.12688/f1000research.11354.1 (PMC5538040; doi:10.12688/f1000research.11354.1)
Supplement: Supplementary file 3 [file f1000research-6-12257-s0002.tgz › 5d2560fe-dd7a-44d1-b265-e3522e742149.zip!/SI File 3. NanoOK experiment reports/P2-Lab7-R1-1D_nanook_bwa_passfail.pdf]

NanoOK report for Nott_R9_run2_1D

Pass and fail counts

Type	Pass	Fail
Template	35306	22074

Read lengths

Type	NumReads	TotalBases	Mean	Longest	Shortest	N50	N50Count	N90	N90Count
Template	57380	410564114	7155.18	353096	5	13072	9081	3578	31900

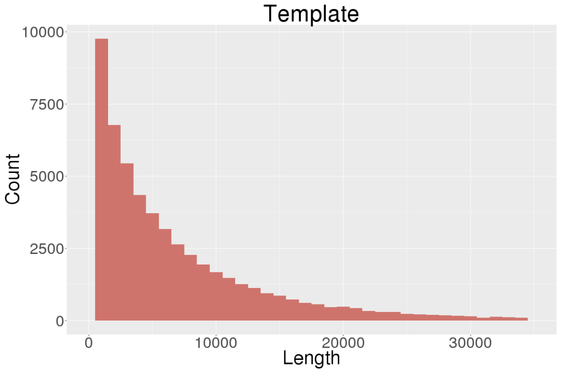

Template alignments

Number of reads	57380	
Number of reads with alignments	47940	(83.55%)
Number of reads without alignments	9440	(16.45%)

ID	Size	Number of Reads	% of Reads	Mean read length	Aligned bases	Mean coverage	Longest Perf Kmer
Control sequence	3560	0	0.00	0.00	0	0.00	0
Escherichia coli	4641652	47940	83.55	7910.68	367038326	79.07	273

Escherichia coli error analysis

	Template
Overall base identity (excluding indels)	76.46%
Aligned base identity (excluding indels)	88.98%
Identical bases per 100 aligned bases (including indels)	79.00%
Inserted bases per 100 aligned bases (including indels)	3.37%
Deleted bases per 100 aligned bases (including indels)	7.85%
Substitutions per 100 aligned bases (including indels)	9.78%
Mean insertion size	1.57
Mean deletion size	1.67

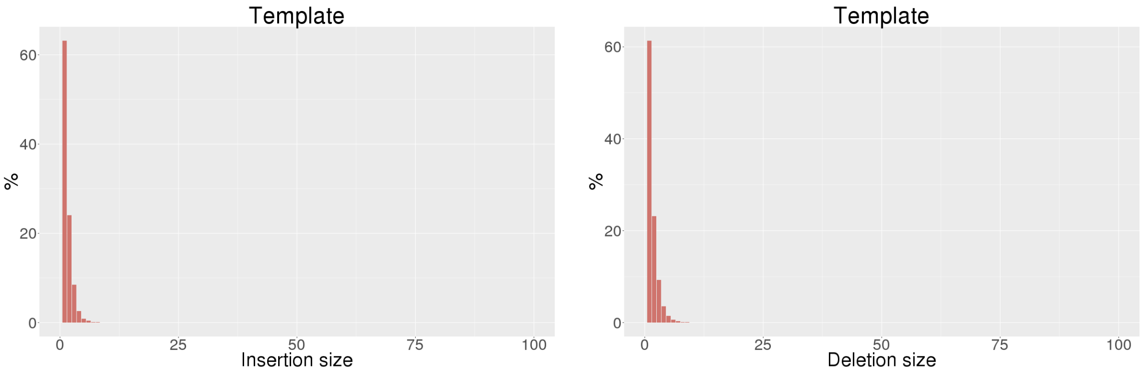

Escherichia coli read identity

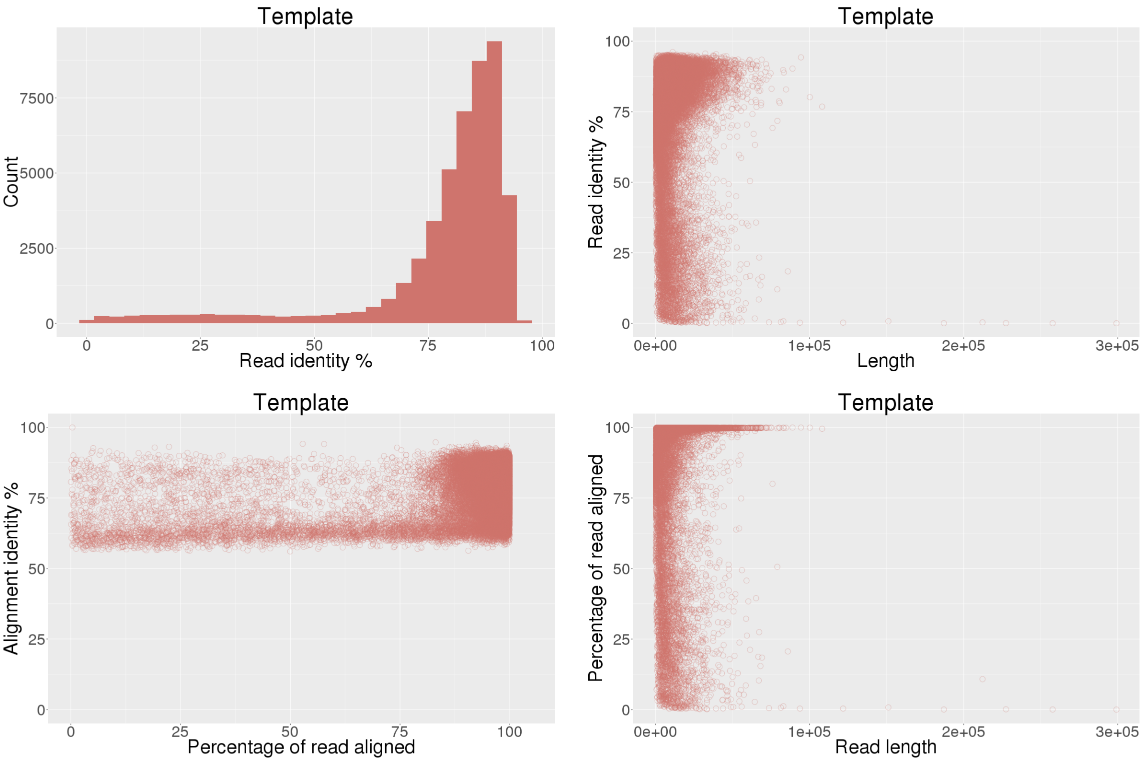

Escherichia coli perfect kmers

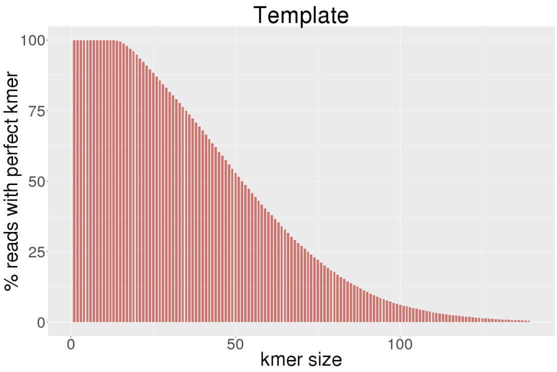

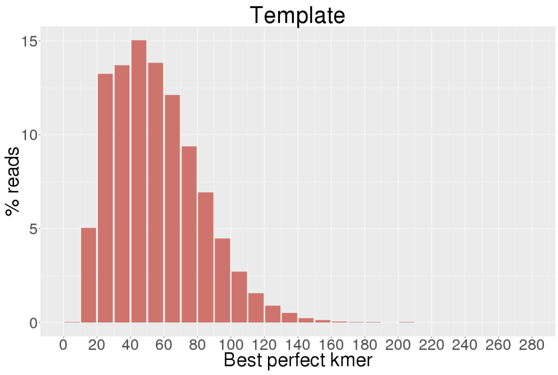

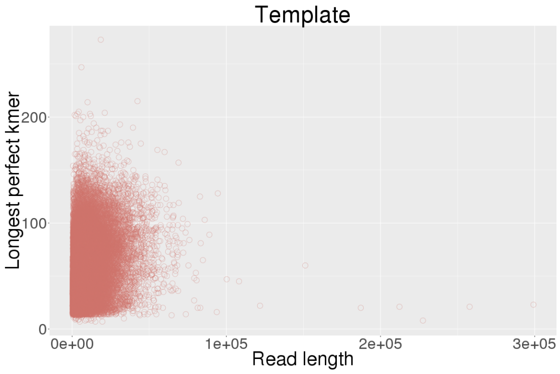

Escherichia coli coverage

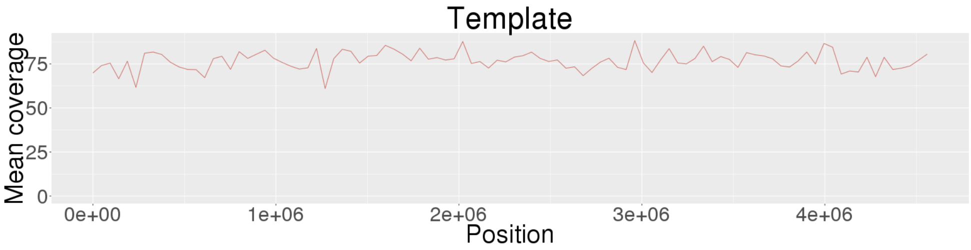

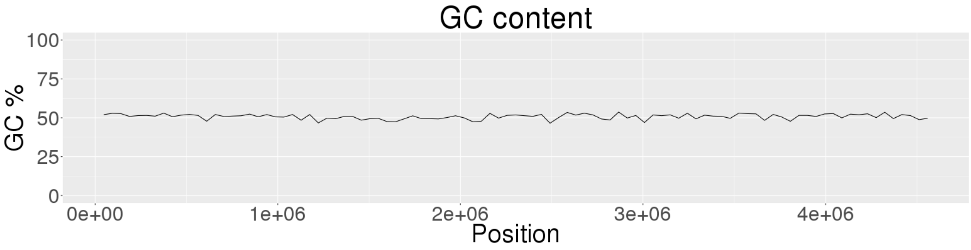

Escherichia coli 5-mer analysis

Under-represented 5-mers

		Template		
Rank	kmer	Ref %	Read %	Diff %
1	TTTTT	0.251	0.039	-0.211
2	AAAAA	0.247	0.092	-0.155
3	GCCAG	0.280	0.176	-0.104
4	GTTTT	0.186	0.093	-0.093
5	CGCCA	0.288	0.214	-0.074
6	ATTTT	0.194	0.120	-0.074
7	TTTTG	0.172	0.100	-0.072
8	TTTTA	0.147	0.080	-0.067
9	CCAGA	0.163	0.097	-0.065
10	AAAGA	0.132	0.068	-0.064

Over-represented 5-mers

		Template		
Rank	kmer	Ref %	Read %	Diff %
1	CGGCG	0.221	0.451	0.230
2	GCGGC	0.210	0.405	0.195
3	GGCGG	0.190	0.364	0.174
4	CGCCG	0.219	0.374	0.154
5	CGCGC	0.201	0.354	0.153
6	GGCCG	0.069	0.218	0.149
7	GCCGC	0.209	0.358	0.149
8	GCGCG	0.202	0.332	0.130
9	CCGGC	0.141	0.266	0.125
10	CGGTG	0.160	0.284	0.124

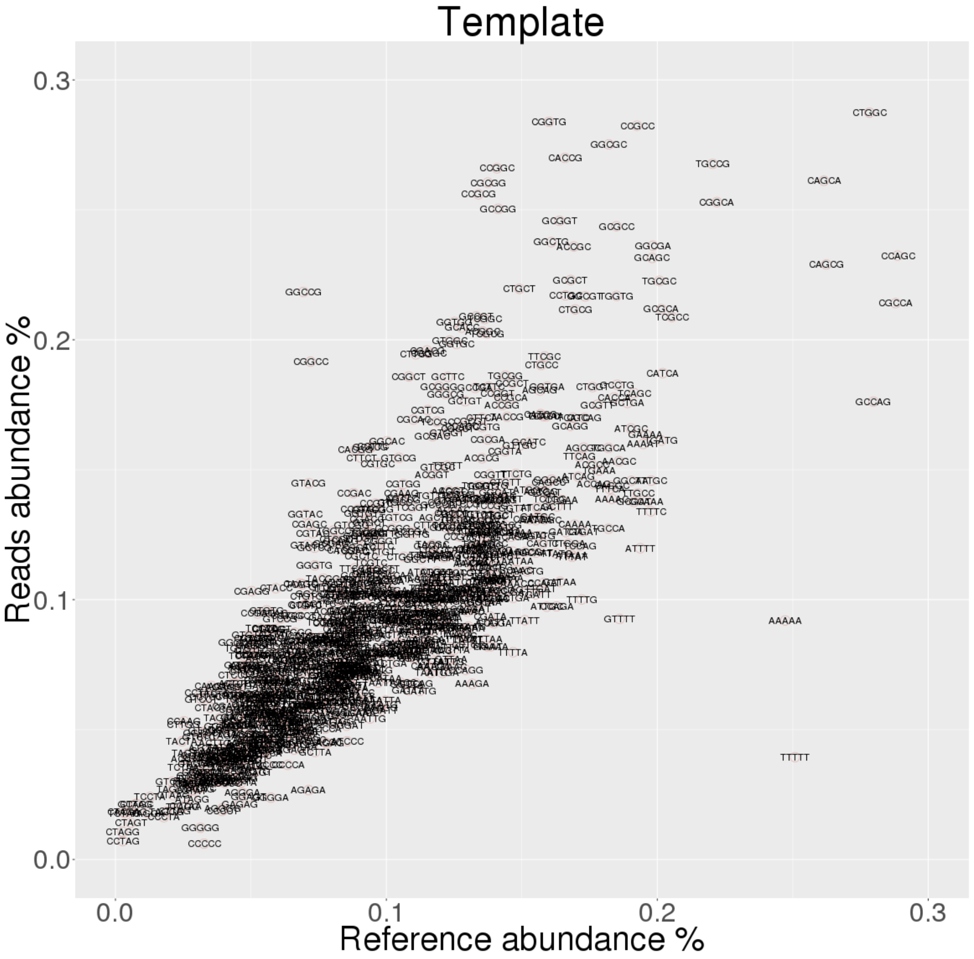

Escherichia coli GC content

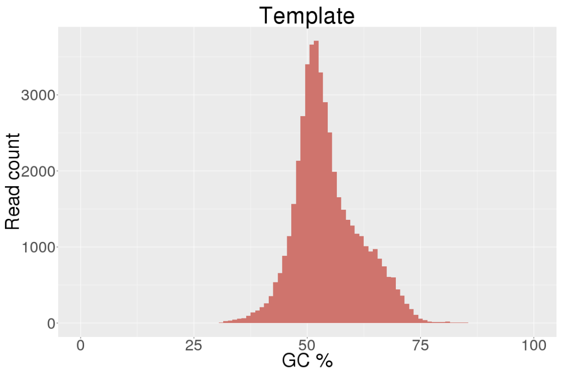

All reference 21mer analysis

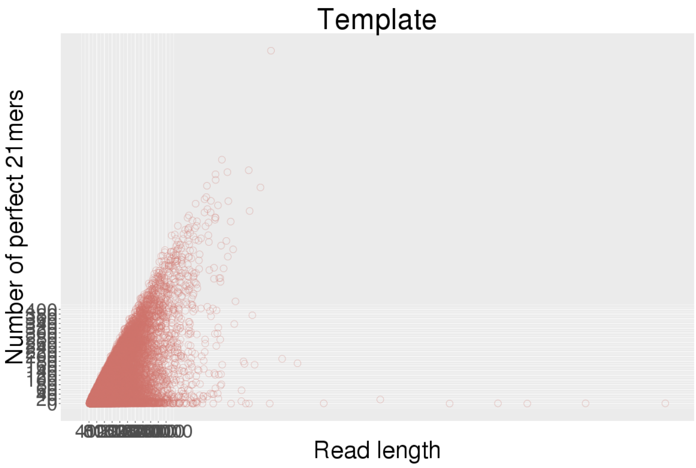

All reference substitutions

		Template substituted %			
		a	c	g	t
Reference	A	0.00	8.61	17.20	4.81
	C	3.70	0.00	4.25	11.44
	G	11.37	4.52	0.00	3.61
	T	4.81	17.20	8.48	0.00

Kmer motifs before errors

3-mer error motif analysis

		Template		
Rank	Insertion	Deletion	Substitution	
1	TTC (3.32%)	CGC (3.38%)	CGC (3.98%)	Most common
2	CGC (3.14%)	TTC (3.07%)	GCC (3.52%)	
3	ATC (2.82%)	GCC (2.96%)	ACC (3.14%)	
4	GCC (2.73%)	ATC (2.94%)	ATC (3.12%)	
5	TTT (2.64%)	GCG (2.90%)	TGC (2.98%)	
6	ACC (2.40%)	GGC (2.72%)	TTC (2.96%)	
7	TGC (2.38%)	TTT (2.72%)	GGC (2.95%)	
8	GCG (2.36%)	ACC (2.58%)	AAC (2.76%)	
9	GGC (2.28%)	TGC (2.18%)	AGC (2.54%)	
10	AGC (2.11%)	AGC (2.09%)	TTT (2.50%)	
	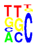	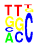	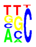	
-10	TTA (1.06%)	AGA (0.92%)	ACT (0.88%)	Least common
-9	AGT (0.89%)	GGA (0.84%)	TAT (0.84%)	
-8	TAA (0.85%)	GTA (0.84%)	GTA (0.84%)	
-7	AGA (0.83%)	AGG (0.82%)	GAG (0.83%)	
-6	GGA (0.82%)	TAA (0.81%)	GGG (0.67%)	
-5	GAG (0.80%)	AGT (0.78%)	AGA (0.65%)	
-4	AGG (0.75%)	CCT (0.72%)	GGA (0.61%)	
-3	GGG (0.51%)	GGG (0.65%)	AGT (0.58%)	
-2	CTA (0.47%)	TAG (0.40%)	TAG (0.40%)	
-1	TAG (0.41%)	CTA (0.37%)	CTA (0.29%)	
	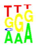	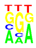	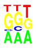	

Kmer space for 3-mers: 64   Random chance for any given 3-mer: 1.56%

4-mer error motif analysis

Rank	Insertion	Template<br>Deletion	Substitution	
1	CGCC (1.17%)	CGCC (1.27%)	CGCC (1.52%)	Most common
2	TTTC (1.15%)	GCGC (1.16%)	GCGC (1.27%)	
3	GCGC (1.03%)	CAGC (1.03%)	CAGC (1.20%)	
4	CAGC (0.95%)	CGGC (1.02%)	CGGC (1.07%)	
5	CTTC (0.90%)	TTTC (1.00%)	CACC (1.03%)	
6	GCTG (0.88%)	GGCG (1.00%)	CATC (1.00%)	
7	CATC (0.85%)	CATC (0.95%)	CCGC (0.97%)	
8	CGGC (0.83%)	CACC (0.89%)	ACGC (0.92%)	
9	CACC (0.80%)	CCGC (0.83%)	TGGC (0.91%)	
10	GCCA (0.77%)	CTTC (0.83%)	CTGC (0.88%)	
	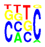	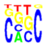	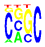	
-10	TCTA (0.11%)	TAGA (0.10%)	GGGA (0.09%)	Least common
-9	GGGA (0.11%)	GGGG (0.10%)	CTAA (0.09%)	
-8	TTAG (0.11%)	TTAG (0.10%)	ACTA (0.08%)	
-7	AGGG (0.10%)	ACTA (0.10%)	TAGT (0.08%)	
-6	TAGA (0.08%)	TCTA (0.09%)	GAGT (0.08%)	
-5	CTAA (0.08%)	CCCT (0.09%)	TAGG (0.07%)	
-4	GGGG (0.07%)	CTAA (0.08%)	TCTA (0.07%)	
-3	CCTA (0.07%)	TAGG (0.06%)	TAGA (0.06%)	
-2	TAGG (0.06%)	CCTA (0.05%)	CCTA (0.04%)	
-1	CTAG (0.01%)	CTAG (0.01%)	CTAG (0.01%)	
	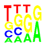	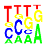	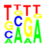	

Kmer space for 4-mers: 256 Random chance for any given 4-mer: 0.39%

5-mer error motif analysis

Rank	Insertion	Template<br>Deletion	Substitution	
1	CGCCA (0.37%)	CCAGC (0.38%)	CCAGC (0.47%)	Most common
2	CCAGC (0.37%)	GCGGC (0.37%)	CCGCC (0.43%)	
3	ATTTC (0.34%)	GGCGC (0.35%)	TCGCC (0.40%)	
4	GTTTC (0.33%)	CCGCC (0.35%)	GCGGC (0.40%)	
5	CGCGC (0.32%)	TCGCC (0.34%)	CTGGC (0.38%)	
6	CCGCC (0.31%)	TGGCG (0.33%)	GCGCC (0.36%)	
7	GCGGC (0.30%)	CGGCG (0.32%)	CGCGC (0.36%)	
8	TCGCC (0.30%)	CTGGC (0.31%)	GCCGC (0.36%)	
9	TCTTC (0.30%)	CGCGC (0.31%)	GGCGC (0.34%)	
10	CAGCA (0.29%)	CGCCA (0.30%)	CAGCC (0.34%)	
	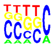	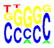	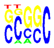	
-10	TAGGA (0.01%)	GGGGG (0.01%)	GGGGG (0.01%)	Least common
-9	CTAGC (0.01%)	TAGGA (0.01%)	CTAGC (0.01%)	
-8	GGGGG (0.01%)	CTAGC (0.00%)	TAGGA (0.00%)	
-7	ACTAG (0.00%)	CTAGT (0.00%)	ACTAG (0.00%)	
-6	GCTAG (0.00%)	GCTAG (0.00%)	GCTAG (0.00%)	
-5	CTAGT (0.00%)	ACTAG (0.00%)	CTAGT (0.00%)	
-4	TCTAG (0.00%)	TCTAG (0.00%)	TCTAG (0.00%)	
-3	CTAGA (0.00%)	CTAGA (0.00%)	CTAGG (0.00%)	
-2	CTAGG (0.00%)	CTAGG (0.00%)	CCTAG (0.00%)	
-1	CCTAG (0.00%)	CCTAG (0.00%)	CTAGA (0.00%)	
	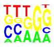	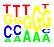	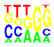	

Kmer space for 5-mers: 1024 Random chance for any given 5-mer: 0.10%
