# Supplementary material for: MinION Analysis and Reference Consortium: Phase 2 data release and analysis of R9.0 chemistry
Source: F1000Res. 2017 May 31;6:760. [Version 1] doi: 10.12688/f1000research.11354.1 (PMC5538040; doi:10.12688/f1000research.11354.1)
Supplement: Supplementary file 3 [file f1000research-6-12257-s0002.tgz › 5d2560fe-dd7a-44d1-b265-e3522e742149.zip!/SI File 3. NanoOK experiment reports/P2-Lab7-R1-1D_nanook_bwa_passonly.pdf]

NanoOK report for Nott_R9_run2_1D

Pass and fail counts

Type	Pass	Fail
Template	35306	0

Read lengths

Type	NumReads	TotalBases	Mean	Longest	Shortest	N50	N50Count	N90	N90Count
Template	35306	276269908	7825.01	151217	153	13568	5991	3833	20561

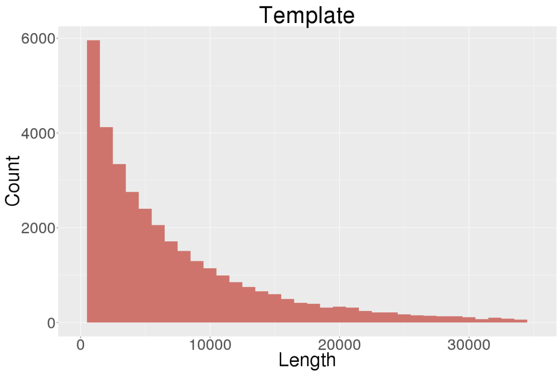

Template alignments

Number of reads	35306	
Number of reads with alignments	35253	(99.85%)
Number of reads without alignments	53	(0.15%)

ID	Size	Number of Reads	% of Reads	Mean read length	Aligned bases	Mean coverage	Longest Perf Kmer
Control sequence	3560	0	0.00	0.00	0	0.00	0
Escherichia coli	4641652	35253	99.85	7810.28	282858586	60.94	273

Escherichia coli error analysis

	Template
Overall base identity (excluding indels)	84.58%
Aligned base identity (excluding indels)	91.38%
Identical bases per 100 aligned bases (including indels)	82.33%
Inserted bases per 100 aligned bases (including indels)	3.08%
Deleted bases per 100 aligned bases (including indels)	6.82%
Substitutions per 100 aligned bases (including indels)	7.77%
Mean insertion size	1.59
Mean deletion size	1.62

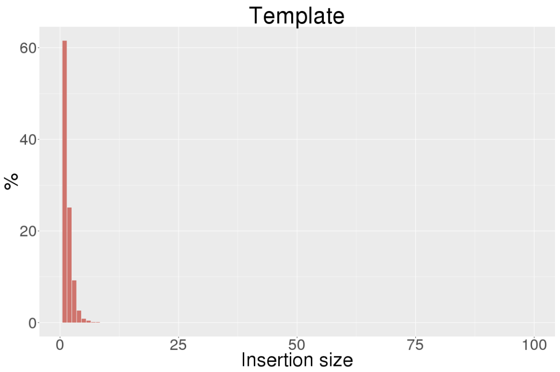

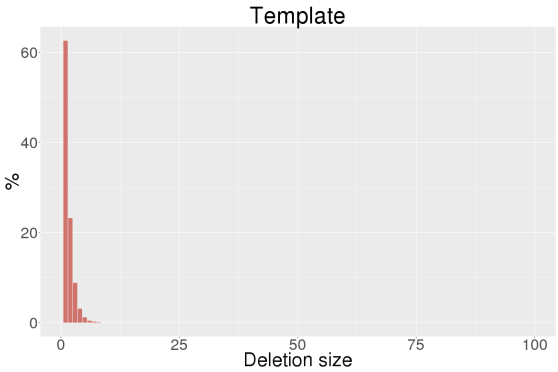

Escherichia coli read identity

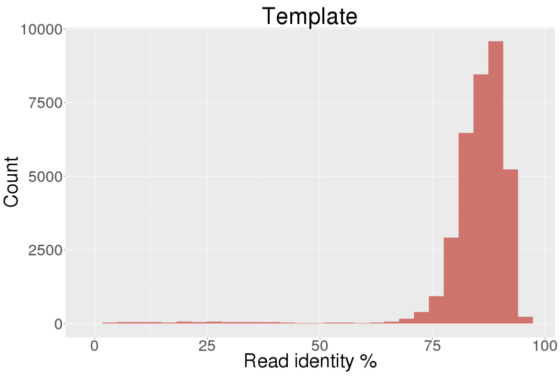

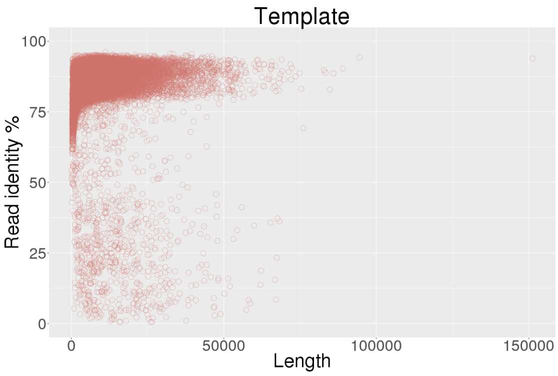

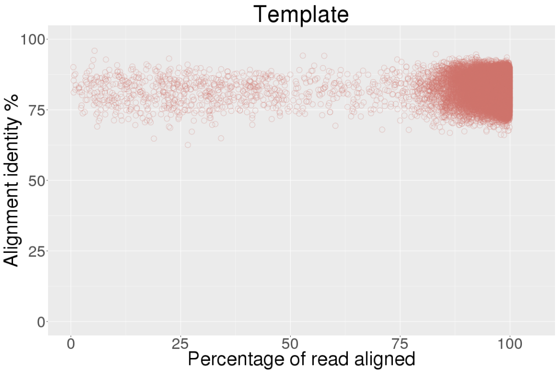

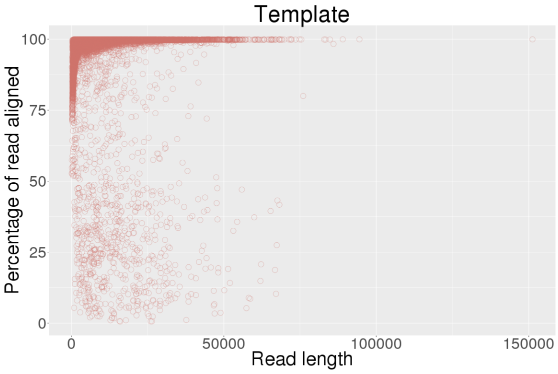

Escherichia coli perfect kmers

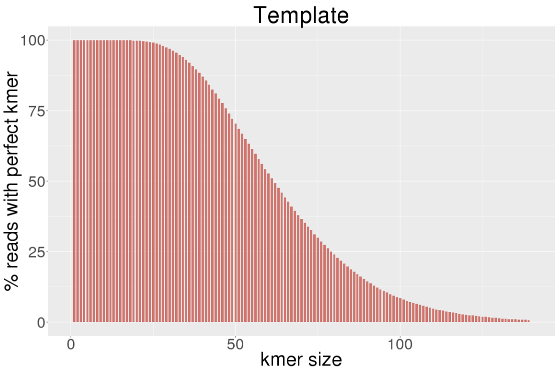

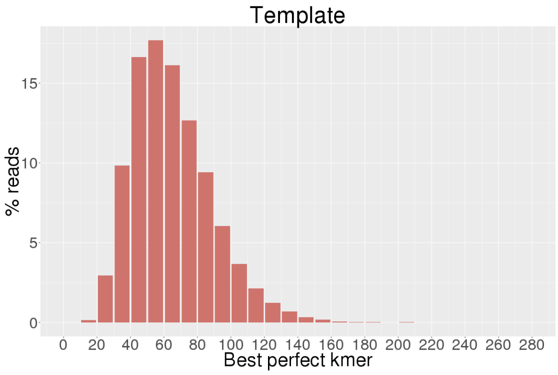

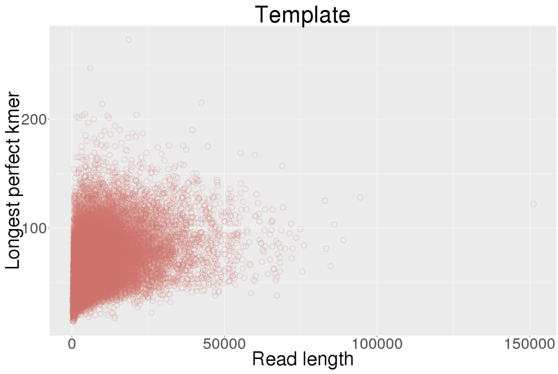

Escherichia coli coverage

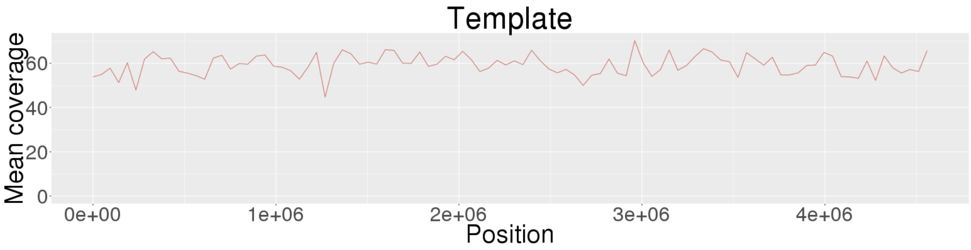

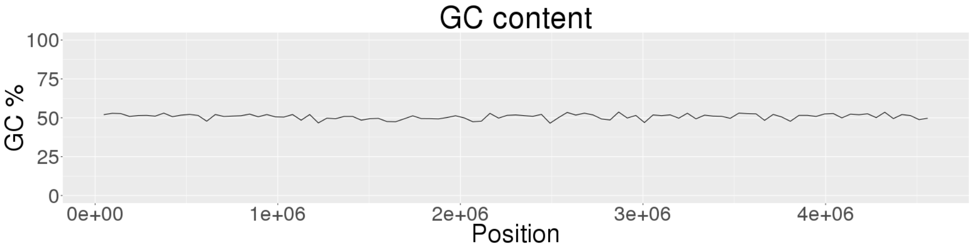

Escherichia coli 5-mer analysis

Under-represented 5-mers

	Template			
Rank	kmer	Ref %	Read %	Diff %
1	TTTTT	0.251	0.046	-0.205
2	AAAAA	0.247	0.111	-0.136
3	GCCAG	0.280	0.189	-0.091
4	GTTTT	0.186	0.107	-0.079
5	CCTGG	0.130	0.054	-0.076
6	CCAGG	0.129	0.065	-0.064
7	CGCCA	0.288	0.226	-0.062
8	TTTTG	0.172	0.116	-0.056
9	CCAGA	0.163	0.110	-0.053
10	AAAGA	0.132	0.081	-0.051

Over-represented 5-mers

	Template			
Rank	kmer	Ref %	Read %	Diff %
1	CGGCG	0.221	0.304	0.083
2	CGCGC	0.201	0.266	0.065
3	GCGGC	0.210	0.274	0.064
4	CGGTG	0.160	0.223	0.063
5	GGCGG	0.190	0.251	0.061
6	CTTCT	0.091	0.147	0.056
7	CACGG	0.089	0.138	0.049
8	GCGCG	0.202	0.250	0.048
9	GTACG	0.071	0.119	0.048
10	GGCCG	0.069	0.115	0.046

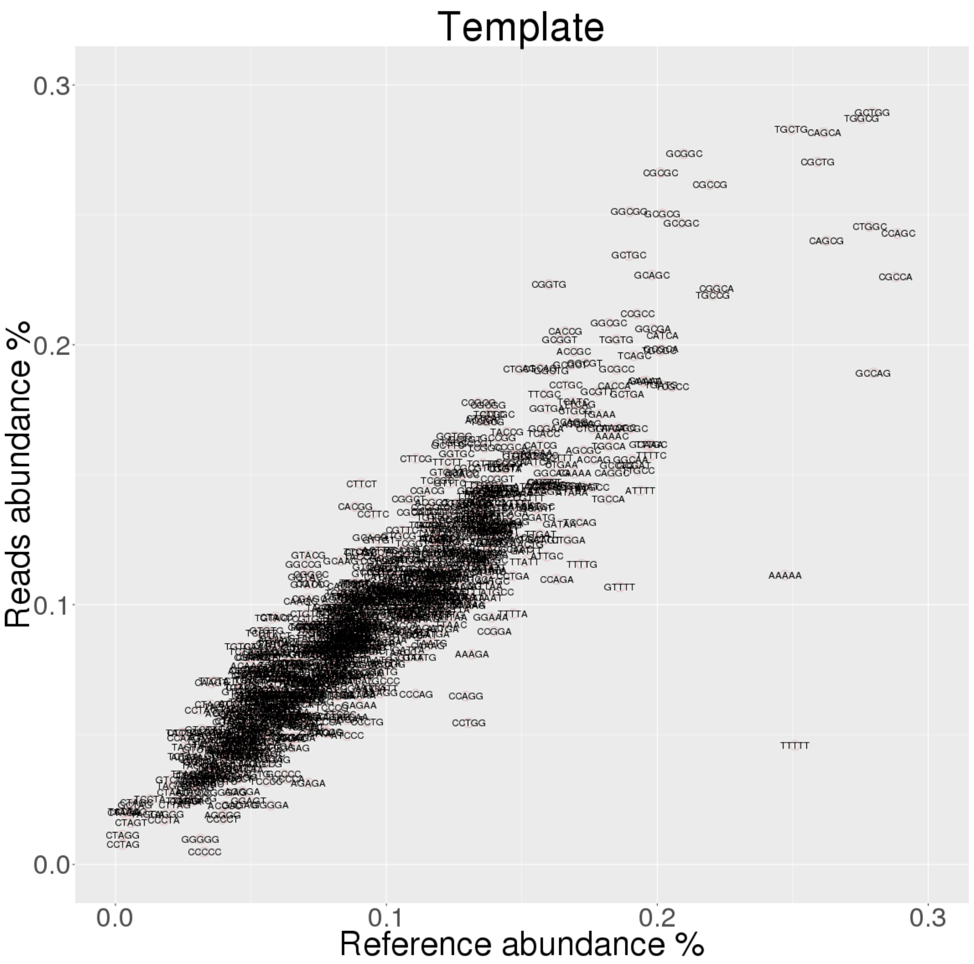

Escherichia coli GC content

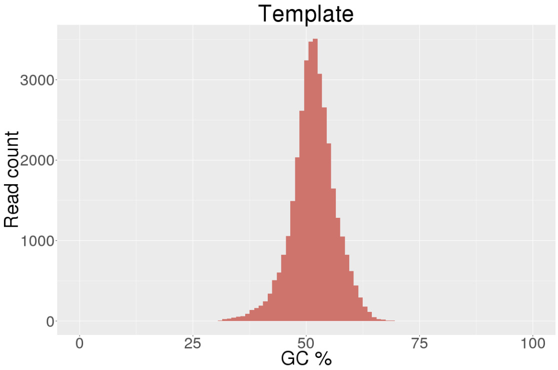

All reference 21mer analysis

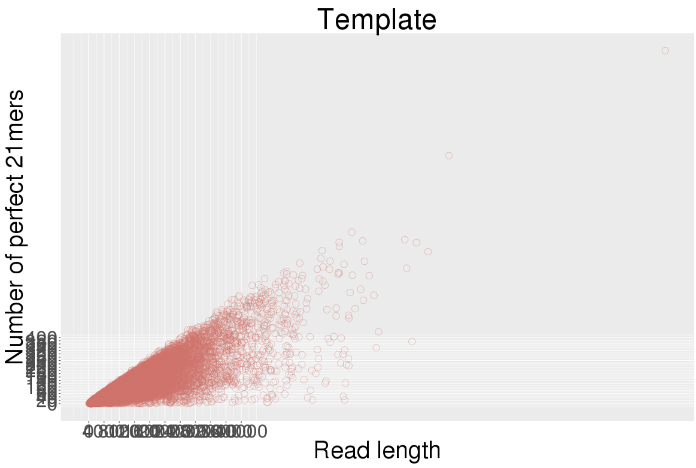

All reference substitutions

		Template substituted %			
		a	c	g	t
Reference	A	0.00	7.01	15.29	4.93
	C	4.36	0.00	3.84	14.43
	G	14.24	4.08	0.00	4.25
	T	4.95	15.58	7.04	0.00

Kmer motifs before errors

3-mer error motif analysis

	Template			
Rank	Insertion	Deletion	Substitution	
1	TTC (3.43%)	CGC (3.02%)	CGC (3.79%)	Most common
2	CGC (3.04%)	TTC (3.02%)	GCC (3.46%)	
3	ATC (2.89%)	TTT (2.97%)	ACC (3.25%)	
4	TTT (2.77%)	ATC (2.94%)	ATC (3.20%)	
5	GCC (2.62%)	GCC (2.74%)	TGC (3.11%)	
6	TGC (2.43%)	GCG (2.51%)	TTC (2.98%)	
7	ACC (2.37%)	GGC (2.50%)	AAC (2.97%)	
8	GCG (2.21%)	ACC (2.49%)	GGC (2.79%)	
9	GGC (2.19%)	ATT (2.16%)	TTT (2.66%)	
10	AAC (2.17%)	TGC (2.13%)	AGC (2.63%)	
	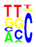	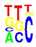	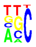	
-10	TGG (1.10%)	AGA (0.96%)	CGA (0.88%)	Least common
-9	TAA (0.95%)	TAA (0.93%)	TGA (0.86%)	
-8	AGT (0.94%)	GTA (0.86%)	GTA (0.84%)	
-7	AGA (0.83%)	AGT (0.84%)	GAG (0.81%)	
-6	GAG (0.81%)	GGA (0.81%)	AGA (0.62%)	
-5	GGA (0.75%)	AGG (0.77%)	GGG (0.61%)	
-4	AGG (0.74%)	CCT (0.72%)	AGT (0.59%)	
-3	CTA (0.49%)	GGG (0.59%)	GGA (0.51%)	
-2	GGG (0.48%)	TAG (0.43%)	TAG (0.44%)	
-1	TAG (0.45%)	CTA (0.40%)	CTA (0.32%)	
	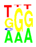	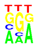	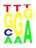	

Kmer space for 3-mers: 64 Random chance for any given 3-mer: 1.56%

4-mer error motif analysis

Rank	Insertion	Template Deletion	Substitution	
1	TTTC (1.22%)	CGCC (1.15%)	CGCC (1.45%)	Most common
2	CGCC (1.10%)	GCGC (1.04%)	CAGC (1.22%)	
3	GCGC (1.01%)	TTTC (1.01%)	GCGC (1.21%)	
4	CAGC (0.95%)	CAGC (0.99%)	CACC (1.01%)	
5	CTTC (0.88%)	CATC (0.92%)	CATC (0.99%)	
6	CATC (0.84%)	CGGC (0.91%)	CGGC (0.97%)	
7	GCTG (0.83%)	GGCG (0.84%)	ACGC (0.93%)	
8	TTTT (0.78%)	GTTT (0.83%)	ATCC (0.93%)	
9	CGGC (0.78%)	ATTT (0.83%)	AACC (0.92%)	
10	CACC (0.77%)	CACC (0.83%)	TTTC (0.91%)	
	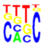	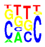	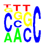	
-10	TTAG (0.12%)	TAGA (0.11%)	TAGT (0.09%)	Least common
-9	TCTA (0.11%)	TTAG (0.11%)	ACTA (0.09%)	
-8	AGGG (0.10%)	ACTA (0.10%)	GGGG (0.08%)	
-7	GGGA (0.10%)	TCTA (0.10%)	TAGG (0.08%)	
-6	CTAA (0.09%)	CTAA (0.09%)	GAGT (0.08%)	
-5	TAGA (0.09%)	CCCT (0.09%)	GGGA (0.08%)	
-4	CCTA (0.07%)	GGGG (0.09%)	TCTA (0.07%)	
-3	GGGG (0.07%)	TAGG (0.06%)	TAGA (0.06%)	
-2	TAGG (0.06%)	CCTA (0.06%)	CCTA (0.04%)	
-1	CTAG (0.01%)	CTAG (0.01%)	CTAG (0.01%)	
	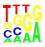	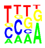	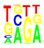	

Kmer space for 4-mers: 256 Random chance for any given 4-mer: 0.39%

5-mer error motif analysis

Rank	Insertion	Template Deletion	Substitution	
1	ATTTC (0.36%)	CCAGC (0.35%)	CCAGC (0.47%)	Most common
2	CCAGC (0.36%)	GCGGC (0.33%)	TCGCC (0.40%)	
3	GTTTC (0.34%)	TCGCC (0.32%)	CCGCC (0.39%)	
4	CGCCA (0.34%)	GGCGC (0.31%)	CTGGC (0.38%)	
5	CGCGC (0.31%)	CCGCC (0.30%)	GCGGC (0.36%)	
6	TTTTC (0.30%)	TGGCG (0.30%)	GCGCC (0.35%)	
7	TCGCC (0.29%)	CTGGC (0.30%)	ACGCC (0.34%)	
8	TCTTC (0.29%)	CGCCA (0.29%)	CGCGC (0.34%)	
9	CCGCC (0.29%)	TTTTC (0.29%)	CAGCC (0.34%)	
10	GCGGC (0.28%)	TCATC (0.28%)	TCACC (0.34%)	
	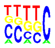	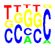	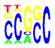	
-10	TAGGA (0.01%)	CCAGG (0.01%)	TCCTA (0.01%)	Least common
-9	CTAGC (0.01%)	GGGGG (0.01%)	GGGGG (0.01%)	
-8	GGGGG (0.00%)	CTAGC (0.00%)	TAGGA (0.00%)	
-7	ACTAG (0.00%)	CTAGT (0.00%)	ACTAG (0.00%)	
-6	GCTAG (0.00%)	ACTAG (0.00%)	CTAGT (0.00%)	
-5	CTAGT (0.00%)	GCTAG (0.00%)	GCTAG (0.00%)	
-4	TCTAG (0.00%)	TCTAG (0.00%)	TCTAG (0.00%)	
-3	CTAGA (0.00%)	CTAGA (0.00%)	CTAGG (0.00%)	
-2	CTAGG (0.00%)	CTAGG (0.00%)	CCTAG (0.00%)	
-1	CCTAG (0.00%)	CCTAG (0.00%)	CTAGA (0.00%)	
	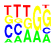	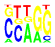	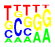	

Kmer space for 5-mers: 1024 Random chance for any given 5-mer: 0.10%
