# Supplementary material for: MinION Analysis and Reference Consortium: Phase 2 data release and analysis of R9.0 chemistry
Source: F1000Res. 2017 May 31;6:760. [Version 1] doi: 10.12688/f1000research.11354.1 (PMC5538040; doi:10.12688/f1000research.11354.1)
Supplement: Supplementary file 3 [file f1000research-6-12257-s0002.tgz › 5d2560fe-dd7a-44d1-b265-e3522e742149.zip!/SI File 3. NanoOK experiment reports/P2-Lab7-R1-2D_nanook_bwa_failonly.pdf]

NanoOK report for Nott_R92_2D

Pass and fail counts

Type	Pass	Fail
Template	0	135818
Complement	0	65961
2D	0	30811

Read lengths

Type	NumReads	TotalBases	Mean	Longest	Shortest	N50	N50Count	N90	N90Count
Template	135818	722522775	5319.79	110841	5	7183	33607	3268	94242
Complement	65961	199770579	3028.62	253028	5	4391	13527	2059	39218
2D	30811	131811218	4278.06	40111	87	4559	9240	2974	24179

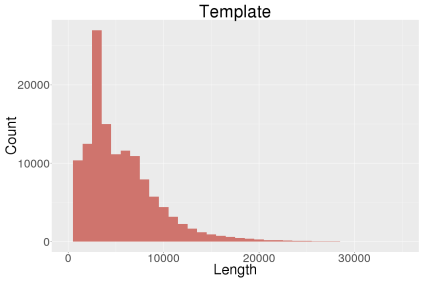

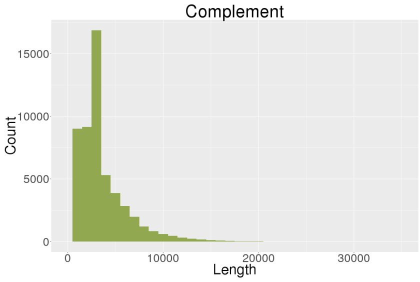

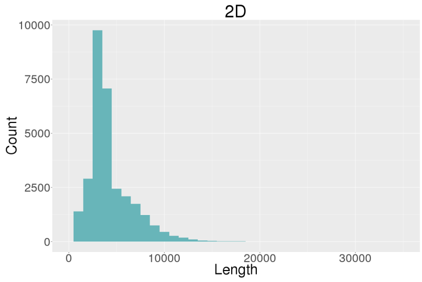

Template alignments

Number of reads	135818	
Number of reads with alignments	104791	(77.16%)
Number of reads without alignments	31027	(22.84%)

ID	Size	Number of Reads	% of Reads	Mean read length	Aligned bases	Mean coverage	Longest Perf Kmer
Control sequence	3560	25311	18.64	3538.14	83231011	23379.50	279
Escherichia coli	4641652	79480	58.52	6911.36	469014922	101.04	274

Complement alignments

Number of reads	65961	
Number of reads with alignments	41558	(63.00%)
Number of reads without alignments	24403	(37.00%)

ID	Size	Number of Reads	% of Reads	Mean read length	Aligned bases	Mean coverage	Longest Perf Kmer
Control sequence	3560	13855	21.00	2835.37	40158156	11280.38	191
Escherichia coli	4641652	27703	42.00	4619.32	92439503	19.92	184

2D alignments

Number of reads	30811	
Number of reads with alignments	25402	(82.44%)
Number of reads without alignments	5409	(17.56%)

ID	Size	Number of Reads	% of Reads	Mean read length	Aligned bases	Mean coverage	Longest Perf Kmer
Control sequence	3560	12430	40.34	3317.22	42140753	11837.29	611
Escherichia coli	4641652	12972	42.10	5657.06	68191644	14.69	215

Control sequence error analysis

	Template	Complement	2D
Overall base identity (excluding indels)	76.64%	79.56%	90.17%
Aligned base identity (excluding indels)	91.42%	90.98%	95.31%
Identical bases per 100 aligned bases (including indels)	82.46%	77.83%	88.23%
Inserted bases per 100 aligned bases (including indels)	2.64%	2.17%	2.68%
Deleted bases per 100 aligned bases (including indels)	7.17%	12.29%	4.74%
Substitutions per 100 aligned bases (including indels)	7.74%	7.72%	4.34%
Mean insertion size	1.51	1.41	1.68
Mean deletion size	1.69	2.22	1.80

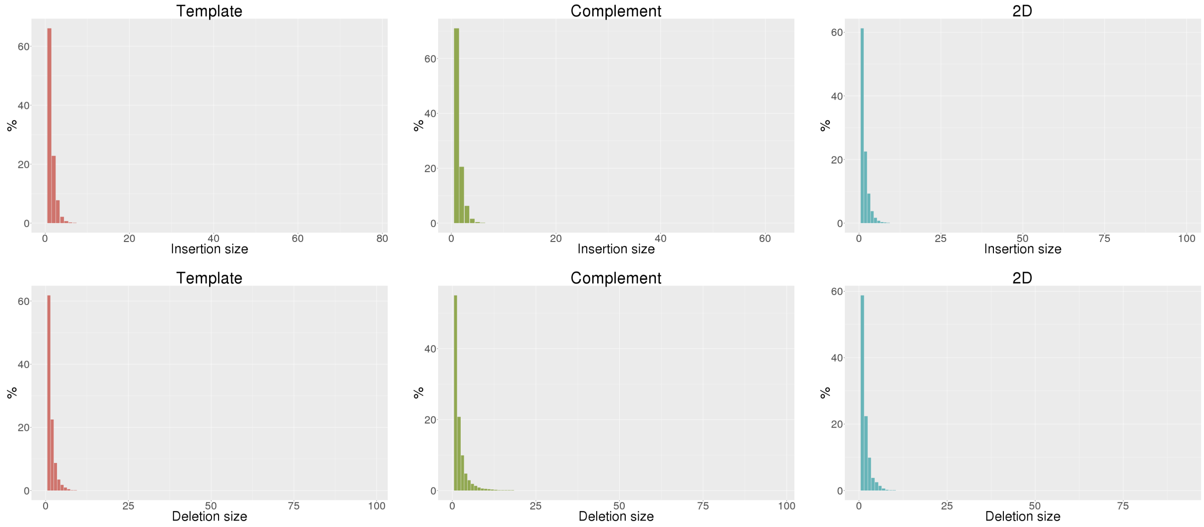

Control sequence read identity

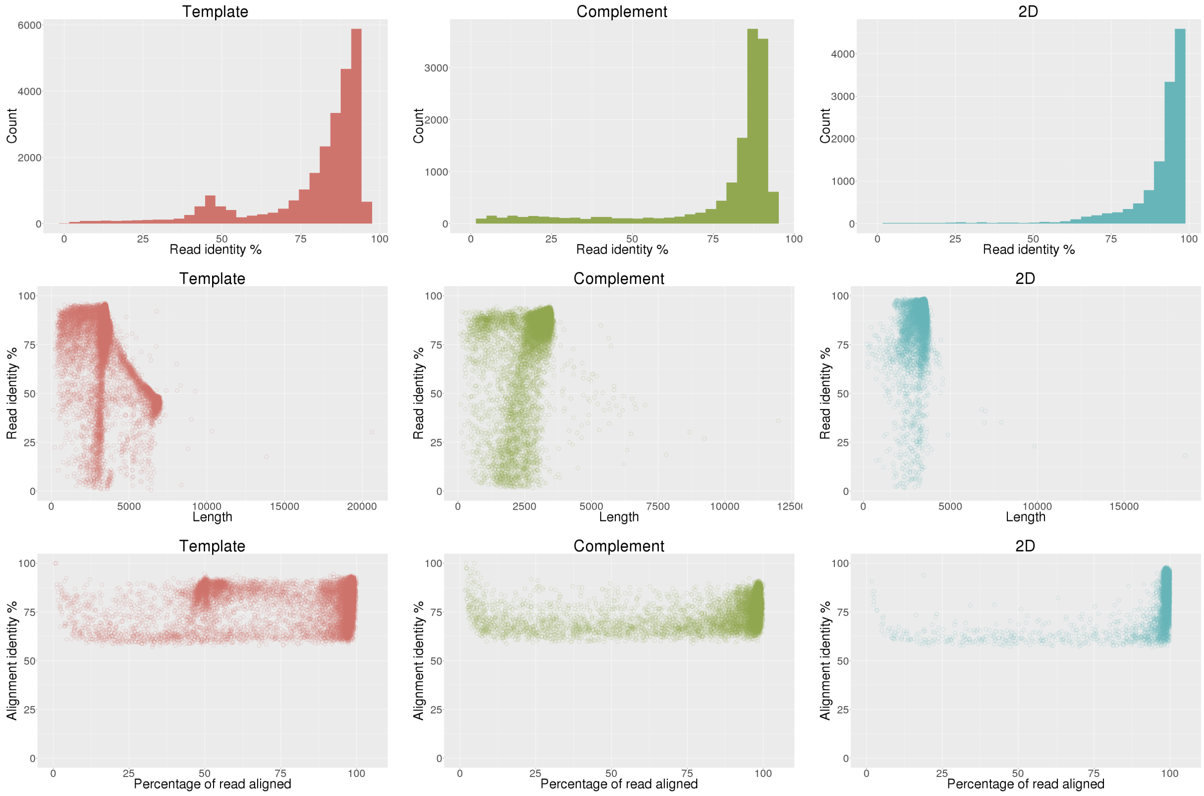

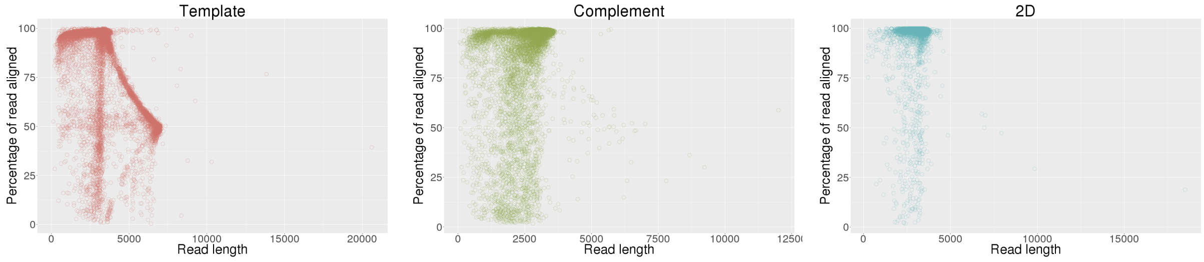

Control sequence perfect kmers

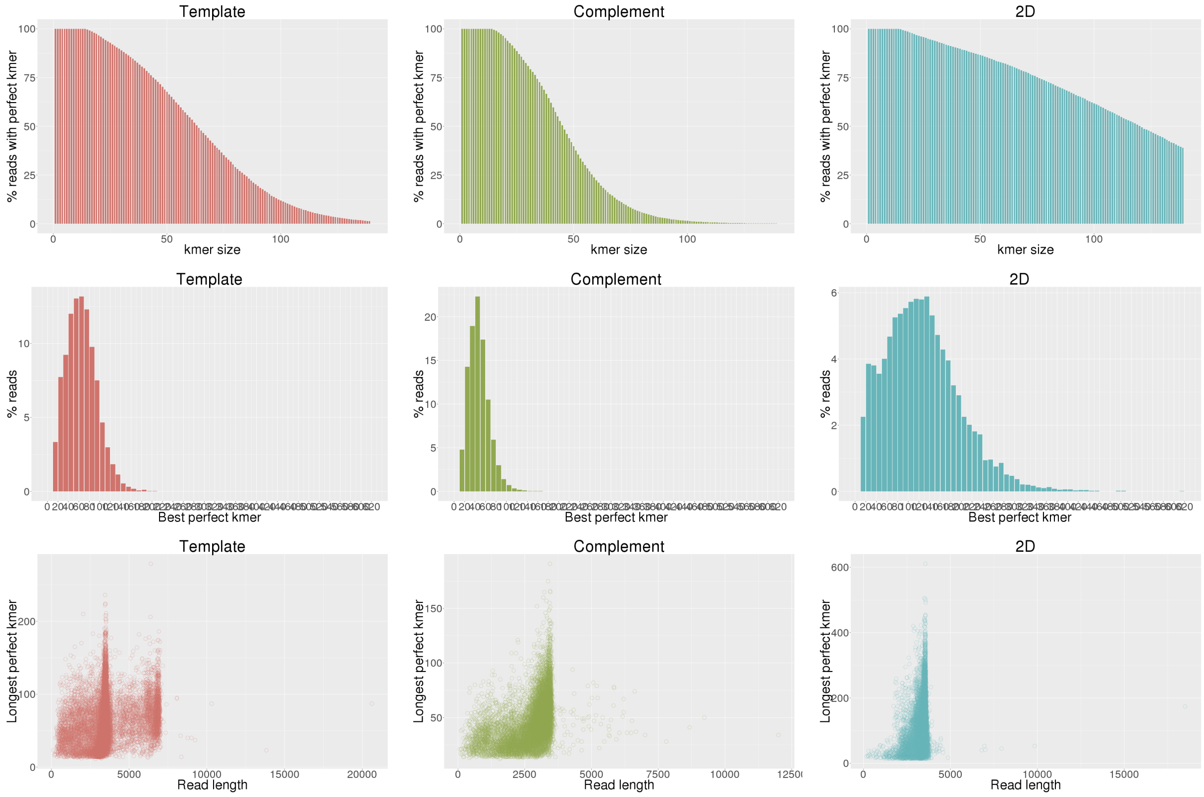

Control sequence coverage

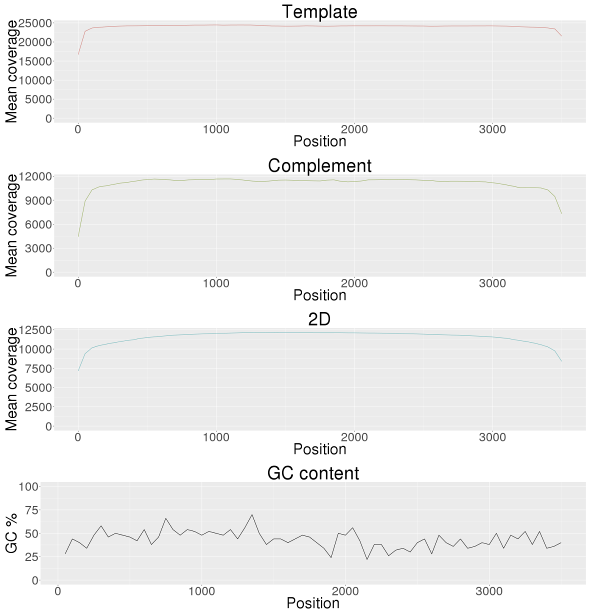

Control sequence 5-mer analysis

Under-represented 5-mers

	Template				Complement				2D			
Rank	kmer	Ref %	Read %	Diff %	kmer	Ref %	Read %	Diff %	kmer	Ref %	Read %	Diff %
1	TTTTT	0.759	0.099	-0.660	TTTTT	0.759	0.135	-0.624	TTTTT	0.759	0.212	-0.546
2	AAAAA	0.478	0.169	-0.309	AAAAA	0.478	0.175	-0.302	AAAAA	0.478	0.225	-0.253
3	TGATG	0.393	0.205	-0.188	TGATG	0.393	0.258	-0.136	TGATG	0.393	0.235	-0.158
4	CTGAT	0.309	0.155	-0.154	GAGCA	0.253	0.118	-0.135	CTGAT	0.309	0.171	-0.138
5	GCAAT	0.309	0.156	-0.154	AGTAG	0.169	0.034	-0.134	GATGT	0.309	0.180	-0.129
6	GATGC	0.281	0.140	-0.141	CTGAT	0.309	0.178	-0.131	GCAAT	0.309	0.183	-0.126
7	GATGT	0.309	0.169	-0.140	CTTTT	0.253	0.126	-0.127	GCTGA	0.281	0.157	-0.124
8	TTATC	0.309	0.170	-0.139	AGTAA	0.253	0.127	-0.126	TGTGA	0.225	0.102	-0.123
9	GTTTT	0.281	0.147	-0.134	GATGT	0.309	0.183	-0.126	TGGTT	0.253	0.141	-0.112
10	GTCAG	0.281	0.152	-0.129	GATGC	0.281	0.167	-0.114	GATGC	0.281	0.173	-0.108

Over-represented 5-mers

	Template				Complement				2D			
Rank	kmer	Ref %	Read %	Diff %	kmer	Ref %	Read %	Diff %	kmer	Ref %	Read %	Diff %
1	CATCA	0.112	0.271	0.158	CATCA	0.112	0.281	0.169	CATCA	0.112	0.271	0.158
2	CGGCG	0.028	0.175	0.147	CAGCA	0.056	0.216	0.160	TCAGC	0.028	0.170	0.141
3	CAGCA	0.056	0.190	0.133	ATCAG	0.056	0.202	0.146	ATCAG	0.056	0.195	0.139
4	ATCAG	0.056	0.182	0.126	AACCA	0.028	0.166	0.137	AACCA	0.028	0.155	0.127
5	CATCT	0.000	0.121	0.121	GCGAA	0.028	0.160	0.132	CAGCA	0.056	0.172	0.115
6	TCAGC	0.028	0.145	0.117	TCAGC	0.028	0.154	0.126	GCATC	0.084	0.200	0.115
7	GCATC	0.084	0.200	0.116	ATAAA	0.112	0.232	0.120	CATCT	0.000	0.115	0.115
8	CCGGC	0.028	0.141	0.113	AATAA	0.197	0.314	0.117	GACGA	0.000	0.097	0.097
9	GACGA	0.000	0.110	0.110	AAACG	0.112	0.223	0.111	AAAAG	0.084	0.178	0.093
10	GCGAA	0.028	0.136	0.108	GCATC	0.084	0.192	0.107	GTCGA	0.000	0.093	0.093

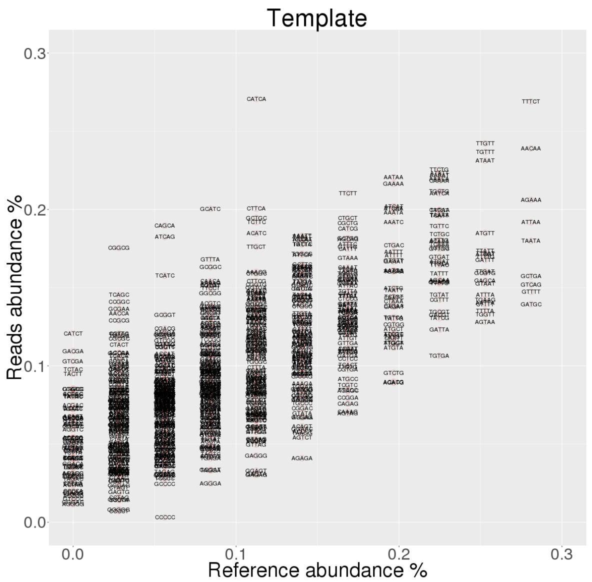

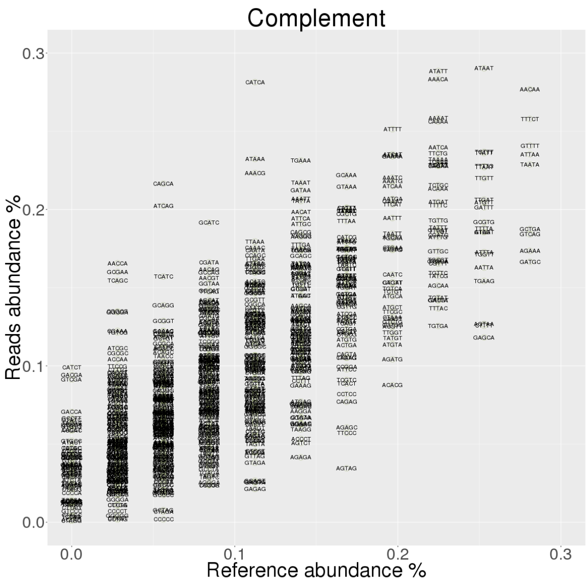

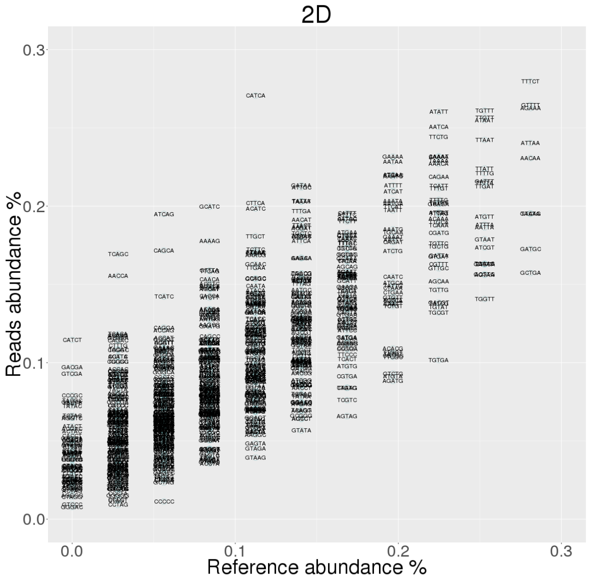

Control sequence GC content

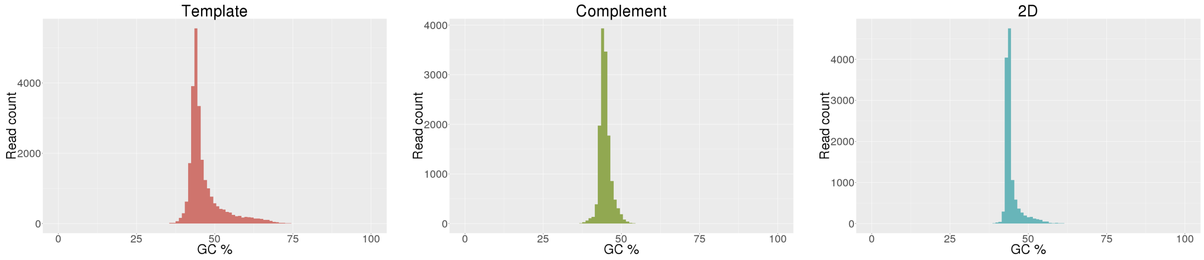

Escherichia coli error analysis

	Template	Complement	2D
Overall base identity (excluding indels)	67.95%	52.31%	62.81%
Aligned base identity (excluding indels)	89.47%	87.52%	81.42%
Identical bases per 100 aligned bases (including indels)	79.58%	72.41%	67.59%
Inserted bases per 100 aligned bases (including indels)	2.75%	2.68%	8.51%
Deleted bases per 100 aligned bases (including indels)	8.30%	14.58%	8.48%
Substitutions per 100 aligned bases (including indels)	9.37%	10.33%	15.42%
Mean insertion size	1.51	1.45	1.94
Mean deletion size	1.72	2.28	1.80

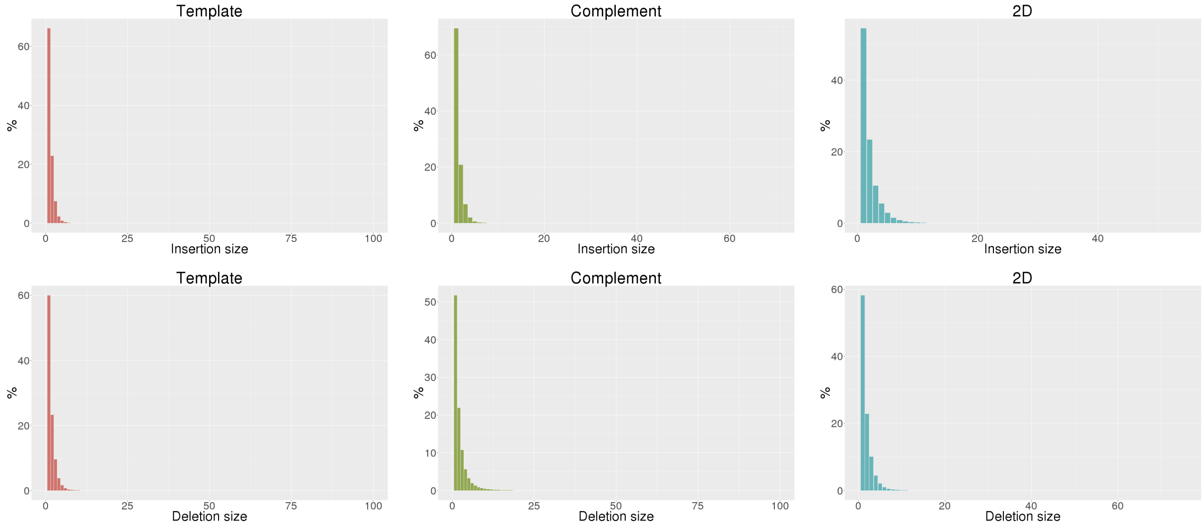

Escherichia coli read identity

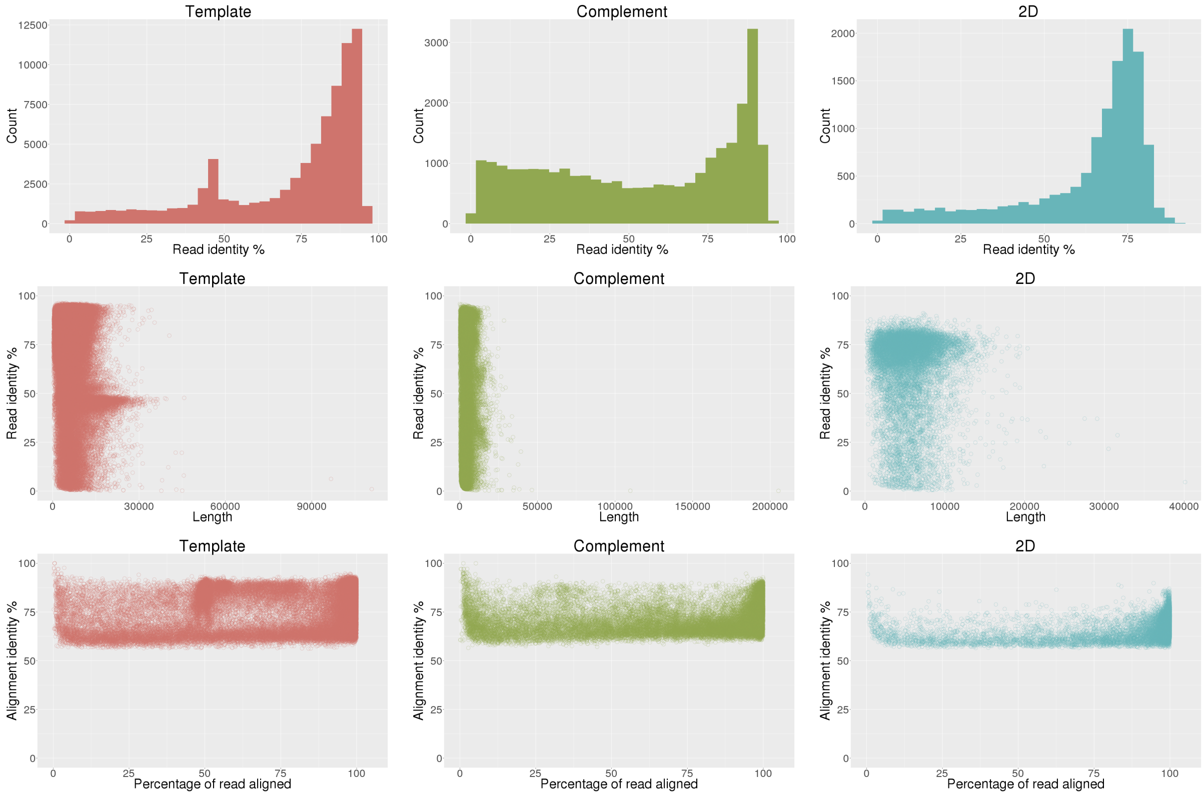

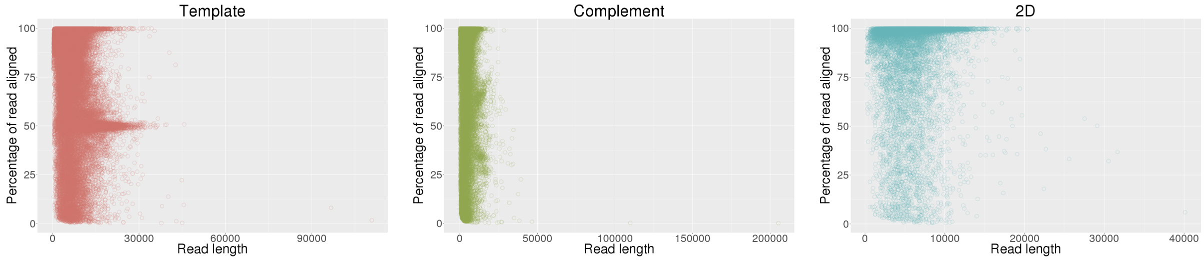

Escherichia coli perfect kmers

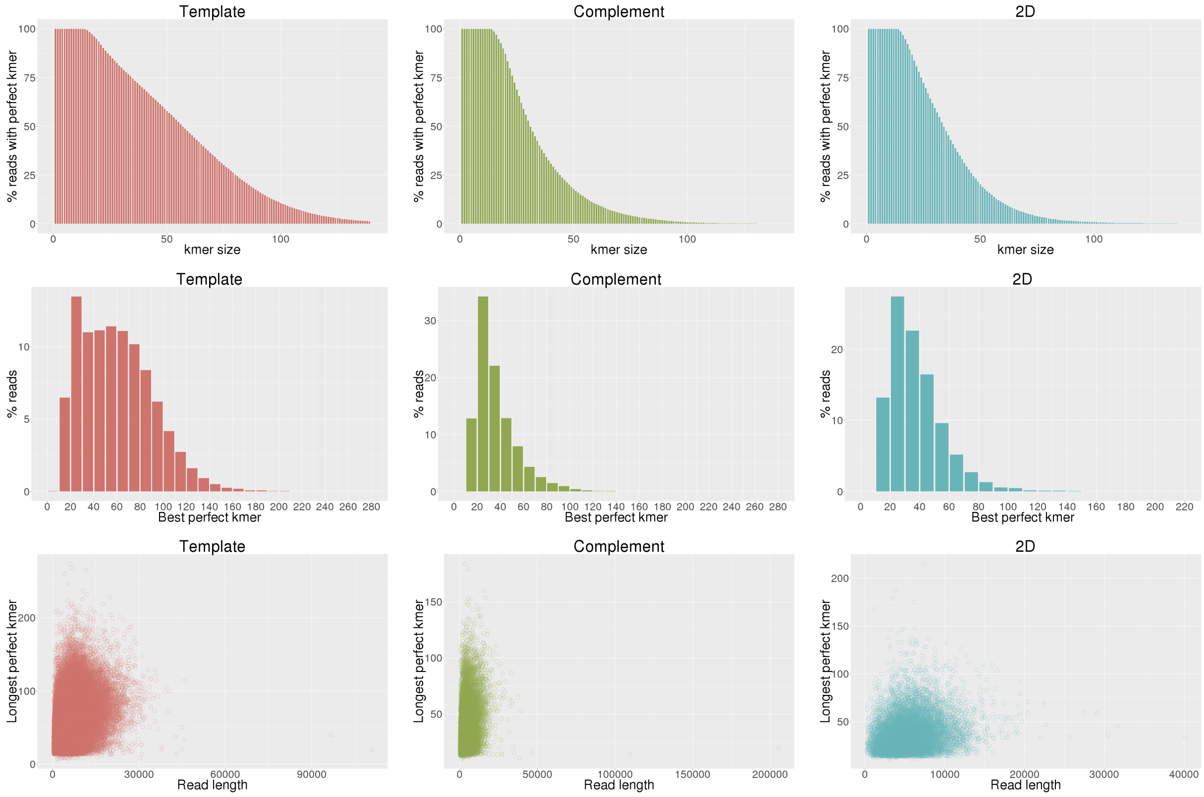

Escherichia coli coverage

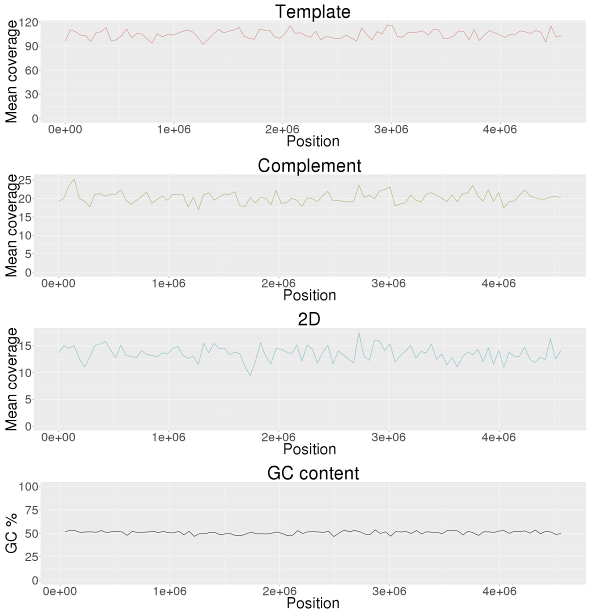

Escherichia coli 5-mer analysis

Under-represented 5-mers

	Template				Complement				2D			
Rank	kmer	Ref %	Read %	Diff %	kmer	Ref %	Read %	Diff %	kmer	Ref %	Read %	Diff %
1	TTTTT	0.251	0.035	-0.215	TTTTT	0.251	0.097	-0.153	TTTTT	0.251	0.103	-0.148
2	AAAAA	0.247	0.088	-0.159	AAAAA	0.247	0.128	-0.119	AAAAA	0.247	0.114	-0.133
3	GTTTT	0.186	0.083	-0.103	CCCAG	0.111	0.039	-0.072	GCCAG	0.280	0.208	-0.072
4	GCCAG	0.280	0.178	-0.102	TCCAG	0.171	0.104	-0.067	ATTGC	0.159	0.093	-0.066
5	TTTTC	0.198	0.120	-0.077	CCAGG	0.129	0.069	-0.060	TGCCA	0.182	0.121	-0.061
6	ATTTT	0.194	0.117	-0.077	CCCGG	0.101	0.046	-0.055	TTGCC	0.193	0.132	-0.060
7	TTTTG	0.172	0.097	-0.075	AAAGA	0.132	0.081	-0.050	TATTG	0.123	0.064	-0.058
8	TTTTA	0.147	0.073	-0.074	GCCCA	0.097	0.048	-0.048	AATAT	0.141	0.083	-0.058
9	CGCCA	0.288	0.218	-0.070	CCTGG	0.130	0.082	-0.048	AATAA	0.148	0.092	-0.056
10	AAAGA	0.132	0.064	-0.068	ATCCC	0.086	0.038	-0.048	ATATT	0.139	0.083	-0.056

Over-represented 5-mers

	Template				Complement				2D			
Rank	kmer	Ref %	Read %	Diff %	kmer	Ref %	Read %	Diff %	kmer	Ref %	Read %	Diff %
1	CGGCG	0.221	0.489	0.268	CGCTG	0.259	0.378	0.118	CGGCG	0.221	0.401	0.180
2	GCGGC	0.210	0.431	0.221	CGGCG	0.221	0.332	0.110	GCGGC	0.210	0.348	0.138
3	GGCGG	0.190	0.387	0.198	TGCCG	0.220	0.316	0.095	GGCGG	0.190	0.326	0.137
4	GGCCG	0.069	0.233	0.164	GCGGC	0.210	0.302	0.092	CCGGC	0.141	0.272	0.131
5	CGCCG	0.219	0.379	0.160	CGCCG	0.219	0.308	0.089	CGGTG	0.160	0.274	0.114
6	CCGGC	0.141	0.287	0.146	GCTGG	0.279	0.357	0.077	GGCTG	0.161	0.275	0.114
7	CGGTG	0.160	0.306	0.146	GCAAA	0.196	0.273	0.077	GCTGG	0.279	0.391	0.111
8	CGCGG	0.137	0.277	0.139	GCCGC	0.209	0.282	0.074	CACCG	0.166	0.275	0.109
9	GCCGC	0.209	0.346	0.137	CATCA	0.202	0.273	0.071	CGCCG	0.219	0.326	0.107
10	CGGCC	0.072	0.203	0.131	TGCTG	0.250	0.321	0.071	GGCGC	0.182	0.285	0.103

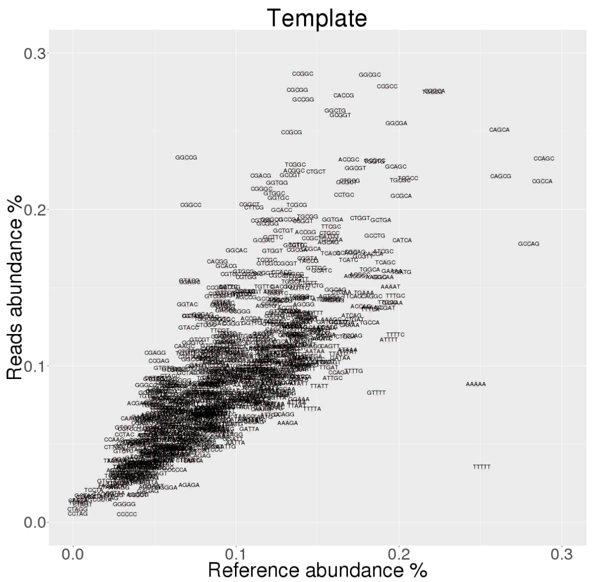

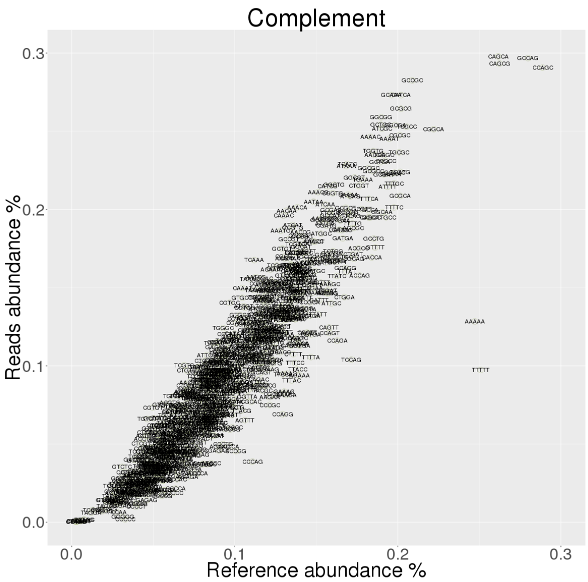

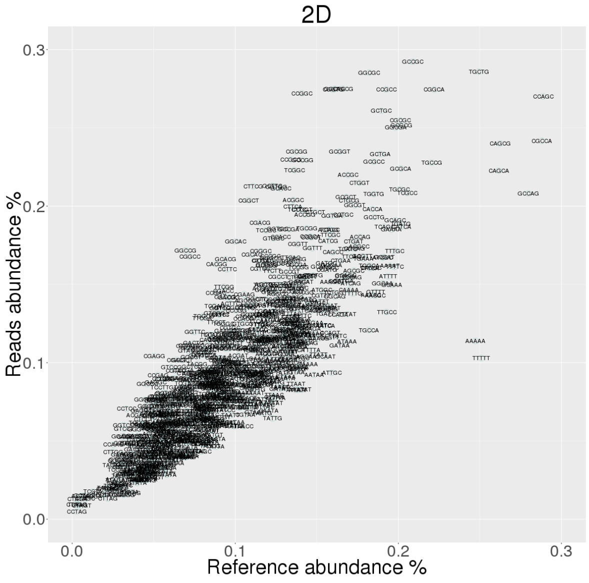

Escherichia coli GC content

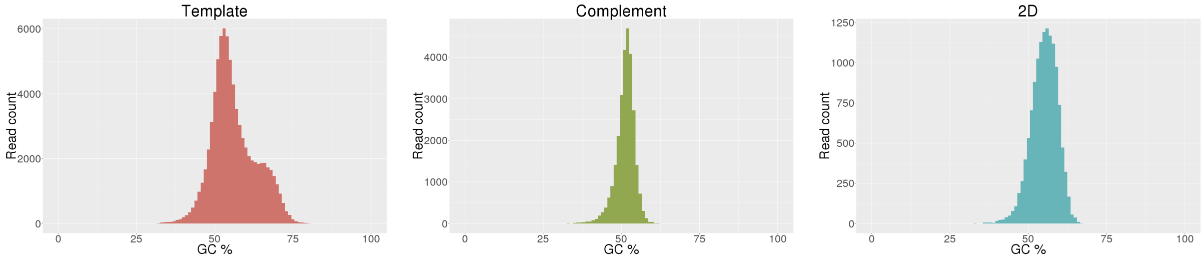

All reference 21mer analysis

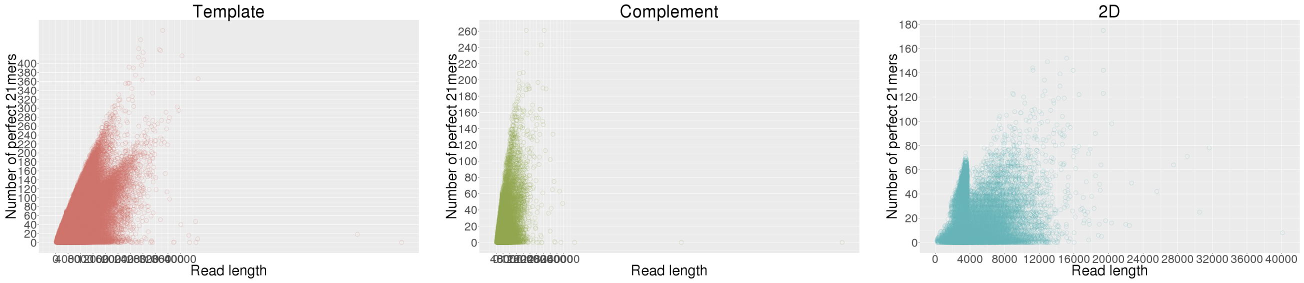

All reference substitutions

		Template substituted %				Complement substituted %				2D substituted %			
		a	c	g	t	a	c	g	t	a	c	g	t
Reference	A	0.00	8.85	17.91	4.66	0.00	5.81	14.42	5.70	0.00	8.79	14.08	6.51
	C	3.22	0.00	4.11	10.65	5.58	0.00	4.14	13.20	5.31	0.00	5.62	9.39
	G	10.32	4.43	0.00	3.23	14.05	4.40	0.00	5.62	9.34	5.79	0.00	5.31
	T	4.70	18.91	9.01	0.00	5.90	15.33	5.85	0.00	6.66	14.55	8.67	0.00

Kmer motifs before errors

3-mer error motif analysis

	Template			Complement			2D			
Rank	Insertion	Deletion	Substitution	Insertion	Deletion	Substitution	Insertion	Deletion	Substitution	
1	TTC (3.31%)	CGC (3.40%)	CGC (3.92%)	TTT (3.38%)	TTT (3.26%)	TTT (3.47%)	TTT (3.13%)	TTT (3.21%)	TTT (3.55%)	Most common
2	CGC (2.86%)	TTC (3.06%)	GCC (3.44%)	ATC (3.11%)	ATC (3.06%)	ATC (3.34%)	TTC (3.08%)	ATC (3.12%)	CGC (3.34%)	
3	ATC (2.77%)	GCC (2.81%)	ATC (3.06%)	TTC (2.95%)	CGC (2.86%)	CGC (3.15%)	ATC (2.84%)	TTC (3.03%)	ATC (3.08%)	
4	TTT (2.73%)	ATC (2.76%)	TGC (3.06%)	CGC (2.92%)	TTC (2.78%)	AAC (2.81%)	CGC (2.76%)	CGC (2.97%)	TTC (2.89%)	
5	GCC (2.56%)	GCG (2.76%)	ACC (2.94%)	ATT (2.53%)	ATT (2.72%)	TGC (2.80%)	TGC (2.38%)	ATT (2.84%)	GCC (2.80%)	
6	TGC (2.34%)	GGC (2.70%)	GGC (2.93%)	GCG (2.50%)	AAA (2.53%)	AAA (2.80%)	GCG (2.28%)	ACC (2.51%)	TGC (2.65%)	
7	GCG (2.25%)	TTT (2.68%)	TTC (2.89%)	TGC (2.44%)	TGC (2.49%)	GCC (2.71%)	GCC (2.26%)	TGC (2.47%)	AAA (2.58%)	
8	ACC (2.24%)	ACC (2.56%)	AAC (2.71%)	AAC (2.31%)	GAT (2.29%)	TTC (2.62%)	ATT (2.23%)	GCG (2.33%)	GGC (2.50%)	
9	GGC (2.17%)	TGC (2.35%)	TTT (2.53%)	GAT (2.27%)	GCG (2.27%)	GCG (2.29%)	GGC (2.17%)	GCC (2.26%)	GCG (2.49%)	
10	AAC (2.07%)	ATT (2.11%)	AGC (2.52%)	AAT (2.20%)	GGC (2.26%)	ACC (2.21%)	TCA (2.17%)	GGC (2.17%)	ACC (2.47%)	
	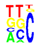	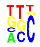	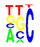	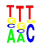	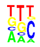	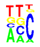	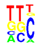	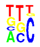	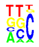	
-10	CCC (1.09%)	GGA (0.96%)	TTA (0.92%)	CCT (0.99%)	ACT (0.96%)	TCT (0.88%)	AGT (0.97%)	TAA (0.93%)	TGT (0.88%)	Least common
-9	GAG (0.94%)	TAT (0.95%)	CAT (0.91%)	AGA (0.90%)	CCC (0.89%)	GAG (0.88%)	TAC (0.96%)	ACA (0.90%)	ACT (0.82%)	
-8	AGT (0.93%)	GTA (0.87%)	CCT (0.85%)	CCC (0.85%)	CCT (0.81%)	GTA (0.84%)	GTA (0.91%)	TAT (0.84%)	GTA (0.82%)	
-7	TAA (0.89%)	AGT (0.81%)	ACT (0.81%)	AGG (0.81%)	GAG (0.77%)	CCT (0.70%)	GAG (0.88%)	AGA (0.82%)	TAT (0.81%)	
-6	GGA (0.84%)	CCT (0.80%)	GGG (0.67%)	AGT (0.77%)	AGG (0.75%)	ACT (0.67%)	GGA (0.86%)	AGG (0.80%)	AGA (0.72%)	
-5	AGA (0.83%)	AGG (0.79%)	AGA (0.67%)	GGA (0.75%)	AGT (0.73%)	GGA (0.62%)	AGA (0.85%)	GTA (0.75%)	GGG (0.69%)	
-4	AGG (0.78%)	TAA (0.77%)	AGT (0.65%)	GTA (0.73%)	GTA (0.67%)	AGT (0.58%)	AGG (0.84%)	AGT (0.74%)	GGA (0.68%)	
-3	GGG (0.53%)	GGG (0.62%)	GGA (0.63%)	GGG (0.47%)	GGG (0.46%)	GGG (0.54%)	GGG (0.58%)	GGG (0.57%)	AGT (0.66%)	
-2	CTA (0.47%)	TAG (0.41%)	TAG (0.43%)	TAG (0.40%)	TAG (0.34%)	TAG (0.42%)	TAG (0.39%)	TAG (0.35%)	TAG (0.38%)	
-1	TAG (0.46%)	CTA (0.35%)	CTA (0.30%)	CTA (0.26%)	CTA (0.24%)	CTA (0.25%)	CTA (0.34%)	CTA (0.34%)	CTA (0.27%)	
	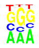	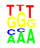	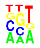	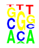	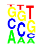	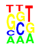	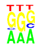	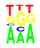	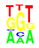	

Kmer space for 3-mers: 64   Random chance for any given 3-mer: 1.56%

4-mer error motif analysis

Rank	Template Insertion	Template Deletion	Template Substitution	Complement Insertion	Complement Deletion	Complement Substitution	2D Insertion	2D Deletion	2D Substitution	
1	TTTC (1.11%)	CGCC (1.20%)	CGCC (1.49%)	TTTT (1.19%)	TTTC (1.09%)	CGCC (1.17%)	TTTT (1.11%)	TTTT (1.27%)	TTTT (1.25%)	
2	CGCC (1.08%)	GCGC (1.14%)	GCGC (1.25%)	ATTT (0.98%)	TTTT (1.07%)	TTTT (1.10%)	TTTC (0.99%)	TATT (1.11%)	CGCC (1.18%)	
3	GCGC (0.94%)	TTTC (1.05%)	CAGC (1.12%)	TTTC (0.95%)	CAGC (0.97%)	CATC (0.97%)	CGCC (0.94%)	TTTC (1.10%)	GCGC (1.08%)	
4	CTTC (0.89%)	CGGC (1.05%)	CGGC (1.07%)	GCGC (0.93%)	ATGC (0.93%)	AAAC (0.97%)	GCGC (0.88%)	ATGC (1.05%)	AAAA (1.00%)	
5	CAGC (0.85%)	CAGC (0.98%)	CATC (0.99%)	CGCC (0.91%)	GCGC (0.92%)	GATG (0.96%)	ATTT (0.87%)	CGCC (0.93%)	TTTC (0.93%)	Most common
6	TTTT (0.82%)	GGCG (0.92%)	CACC (0.94%)	CAGC (0.87%)	CGCC (0.92%)	GCGC (0.94%)	CTTC (0.85%)	GCGC (0.91%)	ATTT (0.92%)	
7	GCTG (0.82%)	CATC (0.88%)	ACGC (0.93%)	GATG (0.85%)	TGAT (0.90%)	ATCA (0.94%)	CAGC (0.83%)	GATG (0.88%)	CATC (0.91%)	
8	CATC (0.79%)	CCGC (0.81%)	CCGC (0.93%)	AATC (0.84%)	GATG (0.90%)	AAAA (0.93%)	GATG (0.82%)	TGAT (0.88%)	GATG (0.91%)	
9	GTTC (0.78%)	TTTT (0.80%)	CTGC (0.92%)	CATC (0.83%)	ATTT (0.88%)	CAGC (0.88%)	GCTG (0.82%)	CATC (0.86%)	CAGC (0.88%)	
10	CGGC (0.78%)	CACC (0.79%)	TGGC (0.90%)	TGAT (0.83%)	TATT (0.86%)	TGCC (0.88%)	AATC (0.78%)	TGCT (0.83%)	GTTT (0.88%)	
	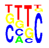	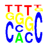	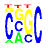	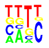	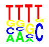	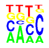	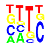	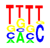	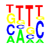	
-10	GGGA (0.12%)	CCCT (0.10%)	CTAA (0.10%)	CTTA (0.09%)	CTTG (0.09%)	GGGG (0.08%)	GGGG (0.10%)	TTAG (0.09%)	GAGT (0.09%)	
-9	TCTA (0.11%)	ACTA (0.10%)	GGGA (0.10%)	CTAA (0.09%)	CTAA (0.09%)	TAGT (0.07%)	CTTA (0.10%)	ACTA (0.08%)	GCTA (0.09%)	
-8	CTTA (0.11%)	TTAG (0.09%)	GGGG (0.09%)	GGGA (0.08%)	CTTA (0.08%)	CCCT (0.07%)	TTAG (0.10%)	GGGG (0.08%)	CTAT (0.09%)	
-7	AGGG (0.10%)	CTTA (0.09%)	GAGT (0.08%)	TAGA (0.07%)	ACTA (0.07%)	GGGA (0.07%)	ACTA (0.09%)	TCTA (0.07%)	TAGT (0.09%)	
-6	TAGA (0.09%)	TCTA (0.09%)	ACTA (0.08%)	TCTA (0.07%)	TAGA (0.07%)	TAGA (0.06%)	CTAA (0.08%)	CTAA (0.07%)	ACTA (0.07%)	Least common
-5	CTAA (0.09%)	GGGG (0.09%)	TAGG (0.07%)	ACTA (0.06%)	GGGG (0.05%)	ACTA (0.05%)	TCTA (0.08%)	TAGA (0.06%)	CCTA (0.07%)	
-4	CCTA (0.09%)	CTAA (0.08%)	TCTA (0.07%)	GGGG (0.06%)	TCTA (0.05%)	TCTA (0.05%)	TAGG (0.08%)	CTTA (0.06%)	TCTA (0.06%)	
-3	GGGG (0.08%)	TAGG (0.06%)	CCTA (0.06%)	TAGG (0.06%)	TAGG (0.04%)	TAGG (0.05%)	CCTA (0.07%)	CCTA (0.06%)	TAGG (0.05%)	
-2	TAGG (0.07%)	CCTA (0.05%)	TAGA (0.06%)	CCTA (0.03%)	CCTA (0.04%)	CCTA (0.05%)	TAGA (0.07%)	TAGG (0.05%)	TAGA (0.05%)	
-1	CTAG (0.02%)	CTAG (0.01%)	CTAG (0.01%)	CTAG (0.00%)	CTAG (0.00%)	CTAG (0.00%)	CTAG (0.01%)	CTAG (0.01%)	CTAG (0.01%)	
	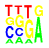	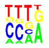	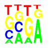	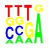	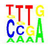	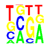	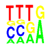	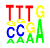	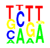	

Kmer space for 4-mers: 256   Random chance for any given 4-mer: 0.39%

5-mer error motif analysis

Rank	Template Insertion	Template Deletion	Template Substitution	Complement Insertion	Complement Deletion	Complement Substitution	2D Insertion	2D Deletion	2D Substitution	
1	ATTTC (0.36%)	GCGGC (0.41%)	CCAGC (0.44%)	TGATG (0.38%)	TTATT (0.47%)	TGATG (0.45%)	TTTTT (0.38%)	TTATT (0.86%)	TGATG (0.38%)	
2	CGCCA (0.36%)	GGCGC (0.36%)	CCGCC (0.41%)	TTTTT (0.38%)	GATGC (0.41%)	TTATC (0.42%)	CGCCA (0.37%)	GATGC (0.59%)	CCAGC (0.36%)	
3	GTTTC (0.33%)	CCAGC (0.35%)	TCGCC (0.39%)	GTTTT (0.35%)	ATGAT (0.40%)	TCGCC (0.38%)	CCAGC (0.34%)	CTGCT (0.59%)	TCGCC (0.35%)	
4	CCAGC (0.32%)	TTTTC (0.35%)	GCGGC (0.39%)	TTATC (0.35%)	TTTTC (0.40%)	CCAGC (0.36%)	TGATG (0.33%)	TCGGA (0.56%)	TTATC (0.34%)	
5	GCGGC (0.29%)	TTATT (0.33%)	CTGGC (0.39%)	CCAGC (0.33%)	TGATG (0.38%)	CTGGC (0.35%)	ATTTC (0.33%)	AGCCT (0.53%)	CTGGC (0.34%)	Most common
6	CCGCC (0.28%)	TCGCC (0.32%)	GCGCC (0.37%)	ATTTT (0.32%)	ATTTT (0.38%)	AATCA (0.34%)	GATGC (0.33%)	TAGCA (0.53%)	GATGC (0.33%)	
7	TGATG (0.28%)	CCGCC (0.31%)	GCCGC (0.35%)	CTGGC (0.32%)	TTATC (0.37%)	TTCGC (0.34%)	AAATC (0.32%)	TGGAA (0.51%)	TTTTT (0.33%)	
8	CTGGC (0.28%)	GATGC (0.31%)	GGCGC (0.34%)	CGCCA (0.31%)	CCAGC (0.37%)	GATGC (0.34%)	GTTTT (0.31%)	ATGAT (0.51%)	GCATC (0.32%)	
9	TTTTC (0.27%)	TGGCG (0.31%)	GATGC (0.33%)	ACGTT (0.30%)	GCGGC (0.36%)	TCATC (0.33%)	CTGGC (0.30%)	TTTTT (0.48%)	AAAAA (0.32%)	
10	TCGCC (0.27%)	CTGGC (0.30%)	ACGCC (0.33%)	TGGCG (0.30%)	TATCG (0.32%)	AAAAC (0.32%)	GCTTC (0.29%)	CGTGT (0.46%)	TTTTC (0.32%)	
	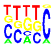	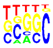	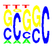	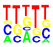	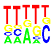	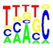	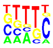	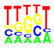	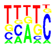	
-10	GCTAG (0.01%)	GGGGG (0.01%)	TAGGA (0.01%)	CCCCC (0.00%)	TAGGG (0.00%)	CCCCC (0.00%)	GCTAG (0.01%)	CCTAG (0.01%)	TAGGG (0.01%)	
-9	CCTGG (0.01%)	TAGGA (0.01%)	CCCTA (0.01%)	CTAGA (0.00%)	GCTAG (0.00%)	GGGGG (0.00%)	CTTAG (0.01%)	TAGGG (0.01%)	GTCTA (0.01%)	
-8	CTAGA (0.01%)	GCTAG (0.00%)	GGGGG (0.01%)	GGGGG (0.00%)	GGGGG (0.00%)	CTAGA (0.00%)	TAGGG (0.01%)	TAGGA (0.00%)	CCCTA (0.00%)	
-7	CCTAG (0.01%)	CTAGT (0.00%)	CTAGC (0.01%)	GCTAG (0.00%)	CTAGA (0.00%)	GCTAG (0.00%)	CTAGA (0.01%)	CTTAG (0.00%)	GCTAG (0.00%)	
-6	CTAGT (0.00%)	CTAGC (0.00%)	GCTAG (0.00%)	CCTAG (0.00%)	CTAGC (0.00%)	CTAGC (0.00%)	CCTAG (0.01%)	GCTAG (0.00%)	CTAGA (0.00%)	Least common
-5	CTAGC (0.00%)	CTAGA (0.00%)	ACTAG (0.00%)	CTAGT (0.00%)	CTAGT (0.00%)	ACTAG (0.00%)	CTAGT (0.00%)	CTAGT (0.00%)	CTAGC (0.00%)	
-4	GGGGG (0.00%)	ACTAG (0.00%)	CTAGA (0.00%)	CTAGC (0.00%)	CCTAG (0.00%)	CTAGT (0.00%)	CTAGC (0.00%)	ACTAG (0.00%)	TCTAG (0.00%)	
-3	ACTAG (0.00%)	TCTAG (0.00%)	TCTAG (0.00%)	ACTAG (0.00%)	ACTAG (0.00%)	TCTAG (0.00%)	ACTAG (0.00%)	CTAGC (0.00%)	ACTAG (0.00%)	
-2	TCTAG (0.00%)	CCTAG (0.00%)	CCTAG (0.00%)	CTAGG (0.00%)	TCTAG (0.00%)	CCTAG (0.00%)	TCTAG (0.00%)	TCTAG (0.00%)	CCTAG (0.00%)	
-1	CTAGG (0.00%)	CTAGG (0.00%)	CTAGG (0.00%)	TCTAG (0.00%)	CTAGG (0.00%)	CTAGG (0.00%)	CTAGG (0.00%)	CTAGG (0.00%)	CTAGG (0.00%)	
	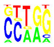	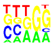	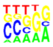	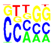	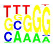	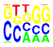	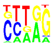	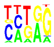	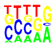	

Kmer space for 5-mers: 1024   Random chance for any given 5-mer: 0.10%
