# Supplementary material for: MinION Analysis and Reference Consortium: Phase 2 data release and analysis of R9.0 chemistry
Source: F1000Res. 2017 May 31;6:760. [Version 1] doi: 10.12688/f1000research.11354.1 (PMC5538040; doi:10.12688/f1000research.11354.1)
Supplement: Supplementary file 3 [file f1000research-6-12257-s0002.tgz › 5d2560fe-dd7a-44d1-b265-e3522e742149.zip!/SI File 3. NanoOK experiment reports/P2-Lab7-R1-2D_nanook_bwa_passfail.pdf]

NanoOK report for Nott_R92_2D

Pass and fail counts

Type	Pass	Fail
Template	80635	135818
Complement	80635	65961
2D	80635	30811

Read lengths

Type	NumReads	TotalBases	Mean	Longest	Shortest	N50	N50Count	N90	N90Count
Template	216453	1268807440	5861.81	110841	5	7596	60273	3436	154446
Complement	146596	681069444	4645.89	253028	5	6538	38113	2955	97804
2D	111446	665635361	5972.72	40111	87	7359	34420	3483	84281

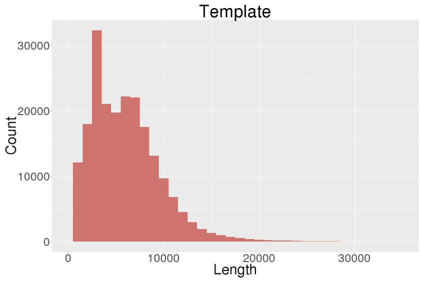

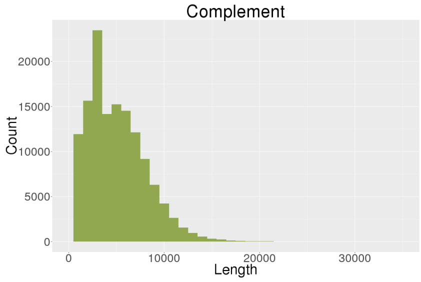

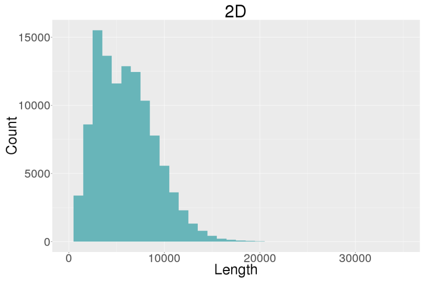

Template alignments

Number of reads	216453	
Number of reads with alignments	185334	(85.62%)
Number of reads without alignments	31119	(14.38%)

ID	Size	Number of Reads	% of Reads	Mean read length	Aligned bases	Mean coverage	Longest Perf Kmer
Control sequence	3560	26814	12.39	3460.89	86375264	24262.71	279
Escherichia coli	4641652	158520	73.24	6886.52	1029053306	221.70	274

Complement alignments

Number of reads	146596	
Number of reads with alignments	121919	(83.17%)
Number of reads without alignments	24677	(16.83%)

ID	Size	Number of Reads	% of Reads	Mean read length	Aligned bases	Mean coverage	Longest Perf Kmer
Control sequence	3560	15341	10.46	2735.06	42957649	12066.76	191
Escherichia coli	4641652	106578	72.70	5676.32	601969976	129.69	203

2D alignments

Number of reads	111446	
Number of reads with alignments	105963	(95.08%)
Number of reads without alignments	5483	(4.92%)

ID	Size	Number of Reads	% of Reads	Mean read length	Aligned bases	Mean coverage	Longest Perf Kmer
Control sequence	3560	13939	12.51	3166.22	45088110	12665.20	611
Escherichia coli	4641652	92024	82.57	6560.75	615735604	132.65	750

Control sequence error analysis

	Template	Complement	2D
Overall base identity (excluding indels)	76.81%	79.67%	90.29%
Aligned base identity (excluding indels)	91.47%	91.00%	95.40%
Identical bases per 100 aligned bases (including indels)	82.52%	77.81%	88.38%
Inserted bases per 100 aligned bases (including indels)	2.63%	2.15%	2.64%
Deleted bases per 100 aligned bases (including indels)	7.14%	12.34%	4.72%
Substitutions per 100 aligned bases (including indels)	7.70%	7.69%	4.26%
Mean insertion size	1.51	1.41	1.68
Mean deletion size	1.69	2.22	1.80

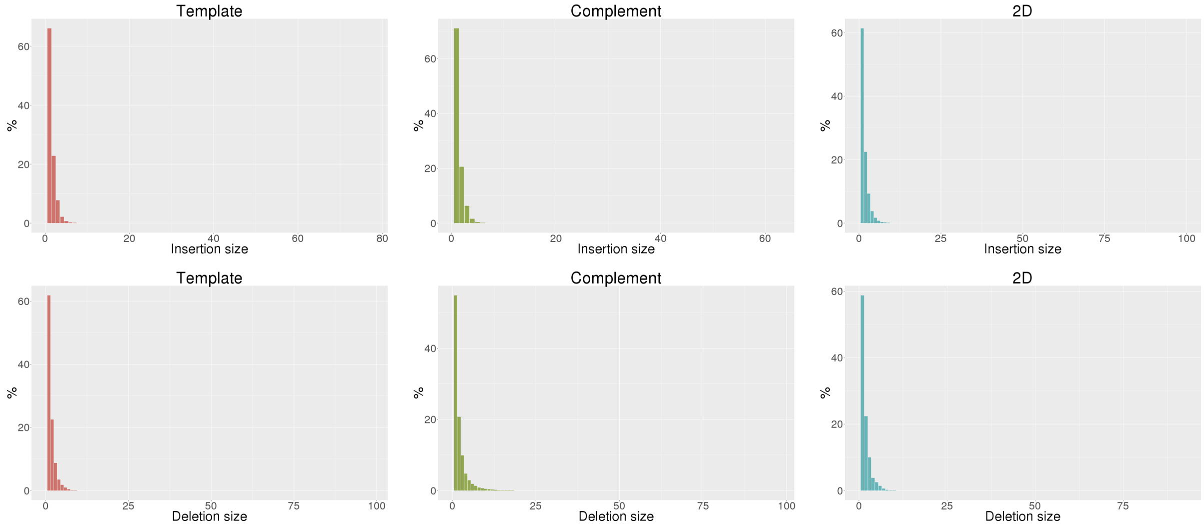

Control sequence read identity

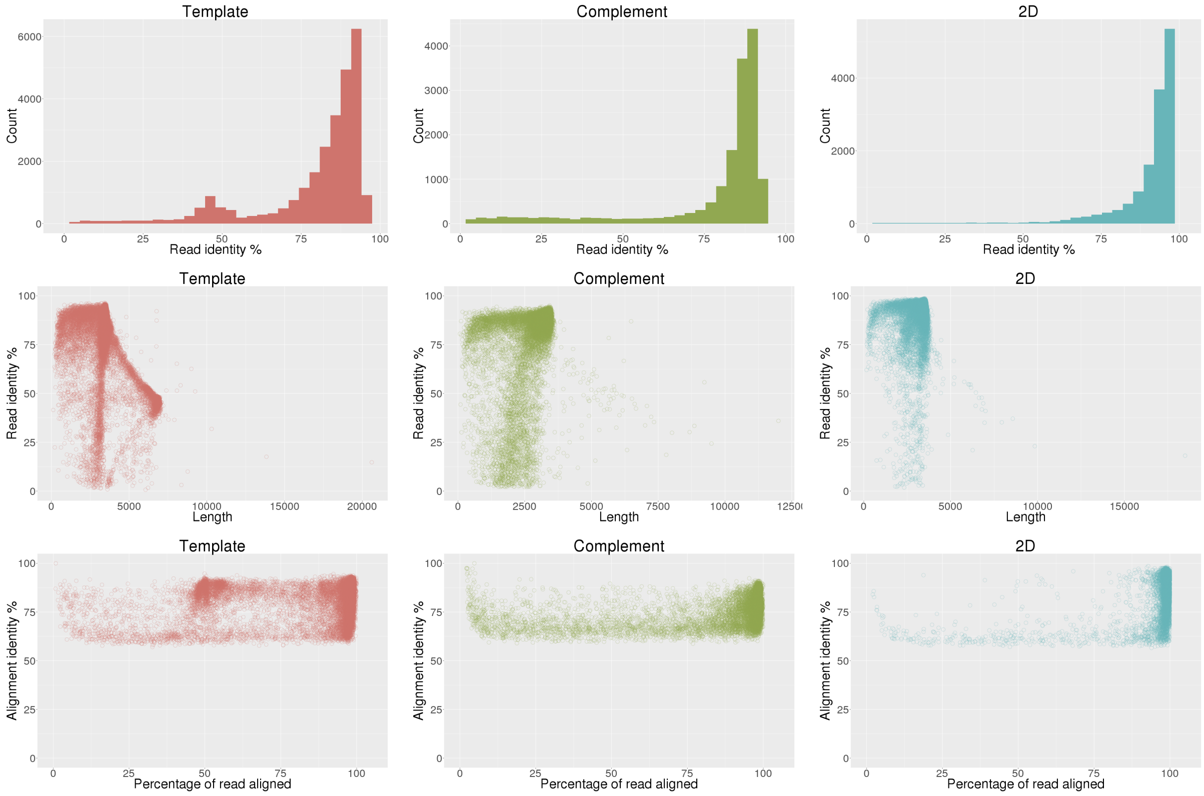

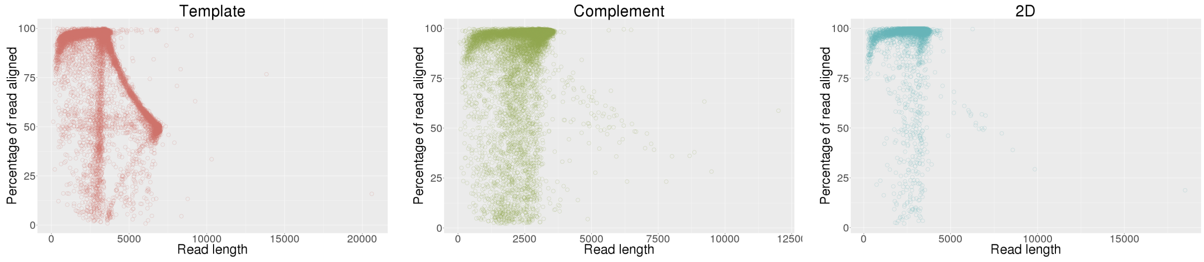

Control sequence perfect kmers

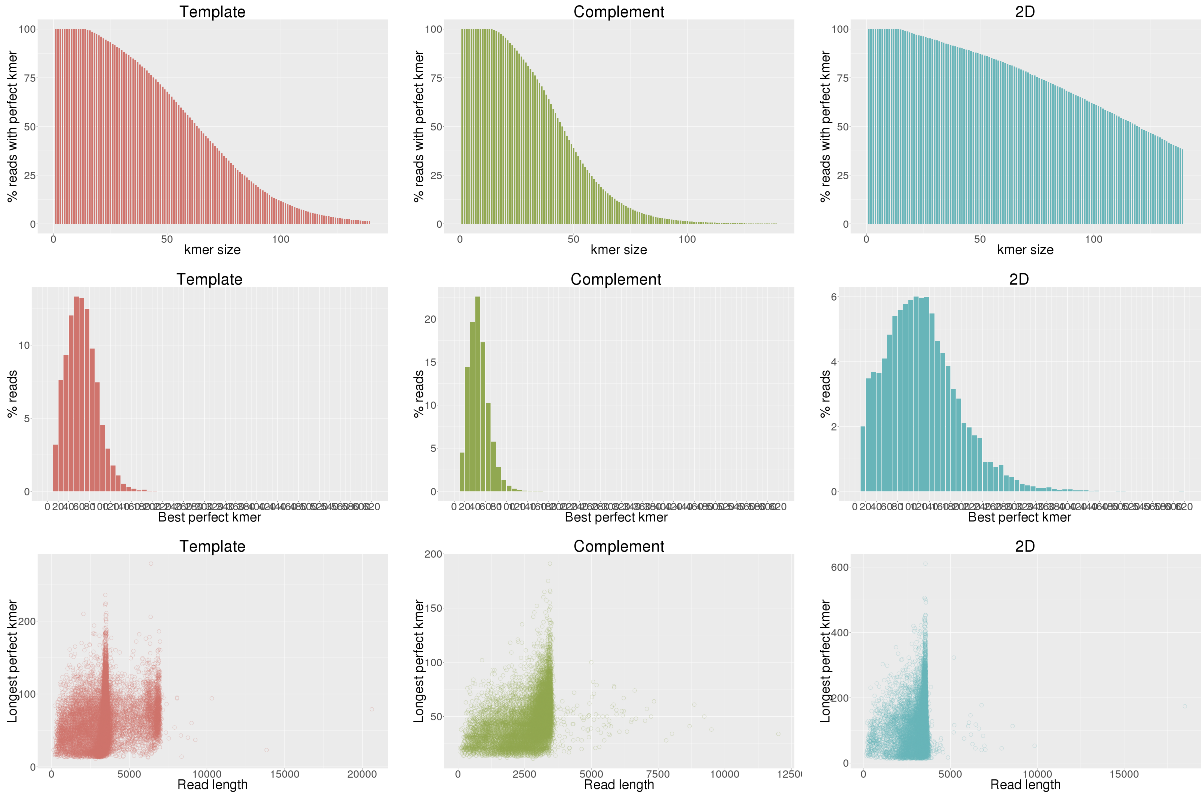

Control sequence coverage

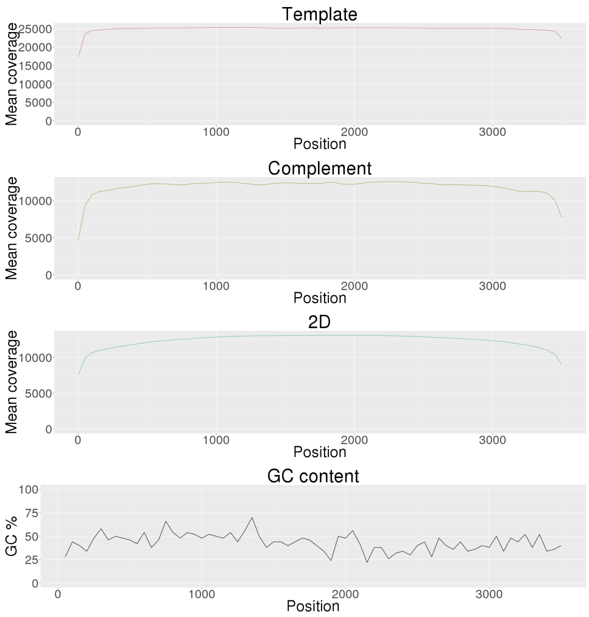

Control sequence 5-mer analysis

Under-represented 5-mers

	Template				Complement				2D			
Rank	kmer	Ref %	Read %	Diff %	kmer	Ref %	Read %	Diff %	kmer	Ref %	Read %	Diff %
1	TTTTT	0.759	0.099	-0.660	TTTTT	0.759	0.134	-0.625	TTTTT	0.759	0.212	-0.546
2	AAAAA	0.478	0.169	-0.308	AAAAA	0.478	0.175	-0.303	AAAAA	0.478	0.225	-0.253
3	TGATG	0.393	0.205	-0.188	TGATG	0.393	0.257	-0.136	TGATG	0.393	0.235	-0.159
4	CTGAT	0.309	0.155	-0.154	GAGCA	0.253	0.118	-0.135	CTGAT	0.309	0.170	-0.139
5	GCAAT	0.309	0.156	-0.154	AGTAG	0.169	0.035	-0.134	GATGT	0.309	0.179	-0.130
6	GATGC	0.281	0.139	-0.142	CTGAT	0.309	0.178	-0.132	GCAAT	0.309	0.183	-0.126
7	GATGT	0.309	0.169	-0.140	CTTTT	0.253	0.126	-0.127	GCTGA	0.281	0.157	-0.124
8	TTATC	0.309	0.170	-0.139	GATGT	0.309	0.183	-0.126	TGTGA	0.225	0.102	-0.123
9	GTTTT	0.281	0.148	-0.133	AGTAA	0.253	0.127	-0.125	TGGTT	0.253	0.140	-0.113
10	GTCAG	0.281	0.152	-0.129	GATGC	0.281	0.167	-0.114	GATGC	0.281	0.172	-0.109

Over-represented 5-mers

	Template				Complement				2D			
Rank	kmer	Ref %	Read %	Diff %	kmer	Ref %	Read %	Diff %	kmer	Ref %	Read %	Diff %
1	CATCA	0.112	0.271	0.159	CATCA	0.112	0.280	0.168	CATCA	0.112	0.272	0.160
2	CGGCG	0.028	0.173	0.145	CAGCA	0.056	0.217	0.161	TCAGC	0.028	0.171	0.143
3	CAGCA	0.056	0.189	0.133	ATCAG	0.056	0.201	0.145	ATCAG	0.056	0.195	0.139
4	ATCAG	0.056	0.183	0.127	AACCA	0.028	0.166	0.137	AACCA	0.028	0.156	0.128
5	CATCT	0.000	0.121	0.121	GCGAA	0.028	0.160	0.132	CAGCA	0.056	0.172	0.116
6	TCAGC	0.028	0.146	0.118	TCAGC	0.028	0.154	0.126	GCATC	0.084	0.200	0.116
7	GCATC	0.084	0.200	0.116	ATAAA	0.112	0.233	0.120	CATCT	0.000	0.115	0.115
8	CCGGC	0.028	0.140	0.112	AATAA	0.197	0.314	0.117	GACGA	0.000	0.098	0.098
9	GACGA	0.000	0.109	0.109	AAACG	0.112	0.223	0.111	AAAAG	0.084	0.178	0.094
10	GCGAA	0.028	0.136	0.108	GCATC	0.084	0.191	0.107	TCACA	0.028	0.120	0.092

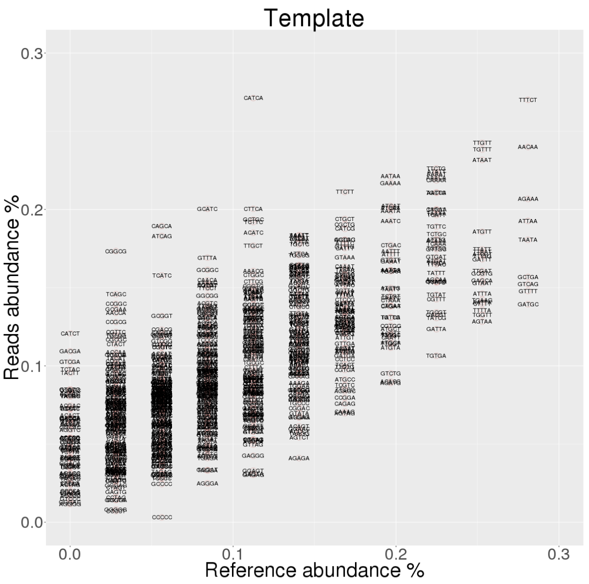

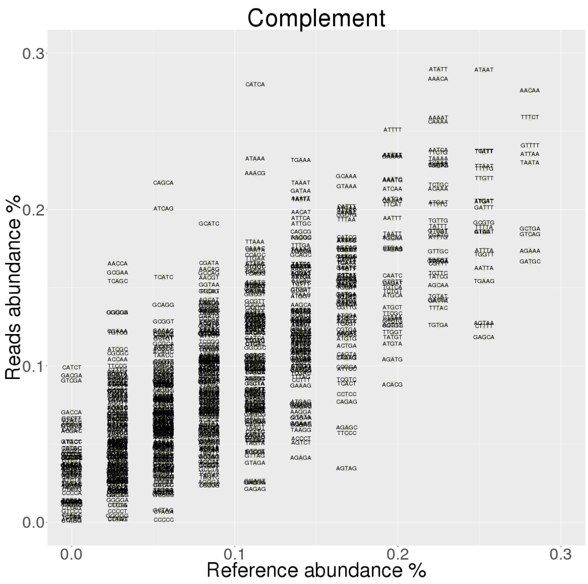

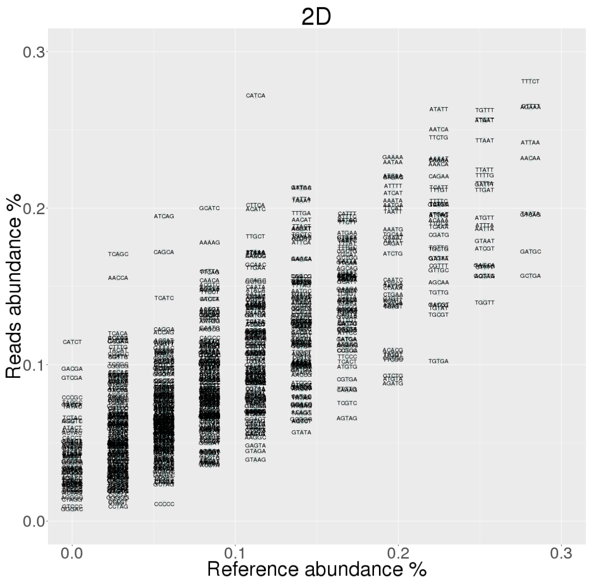

Control sequence GC content

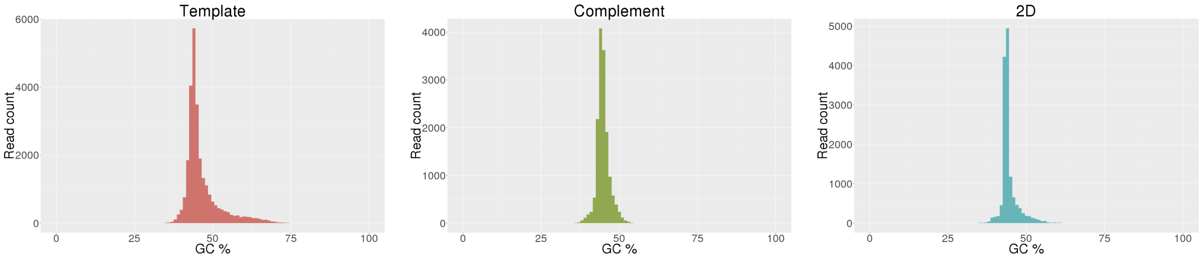

Escherichia coli error analysis

	Template	Complement	2D
Overall base identity (excluding indels)	76.92%	76.34%	89.22%
Aligned base identity (excluding indels)	90.75%	90.82%	94.80%
Identical bases per 100 aligned bases (including indels)	81.60%	76.72%	87.48%
Inserted bases per 100 aligned bases (including indels)	2.56%	2.09%	2.85%
Deleted bases per 100 aligned bases (including indels)	7.52%	13.44%	4.86%
Substitutions per 100 aligned bases (including indels)	8.32%	7.76%	4.80%
Mean insertion size	1.50	1.42	1.72
Mean deletion size	1.67	2.33	1.75

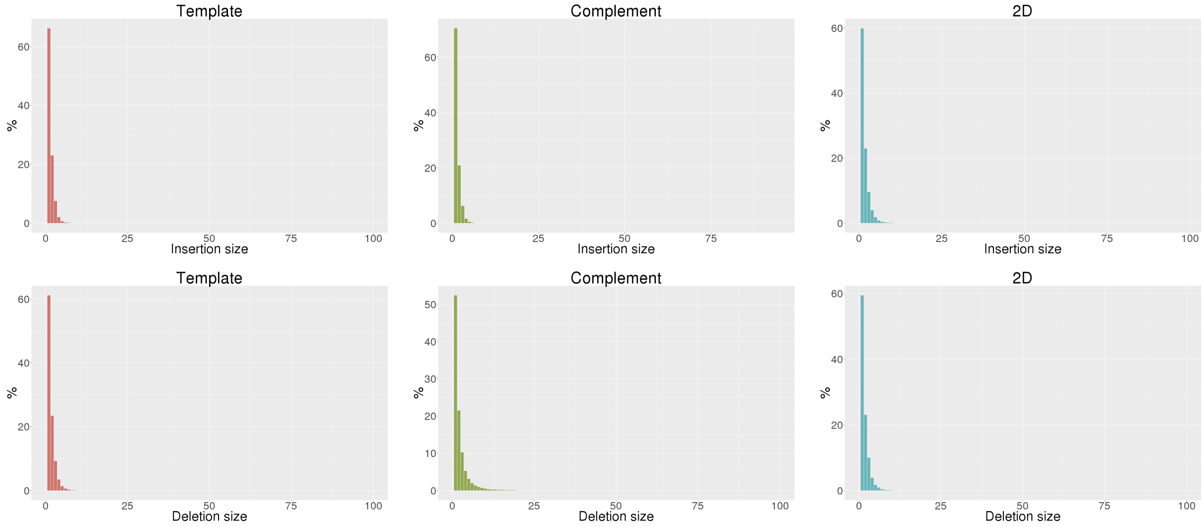

Escherichia coli read identity

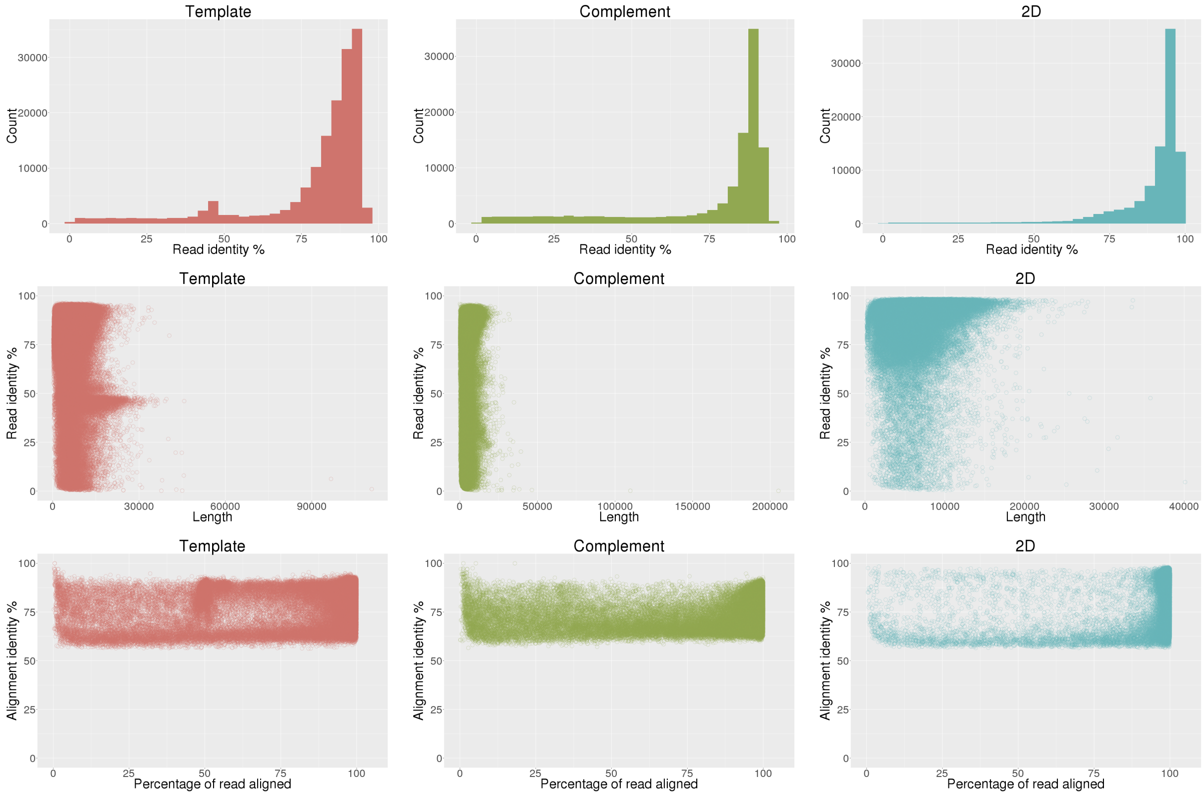

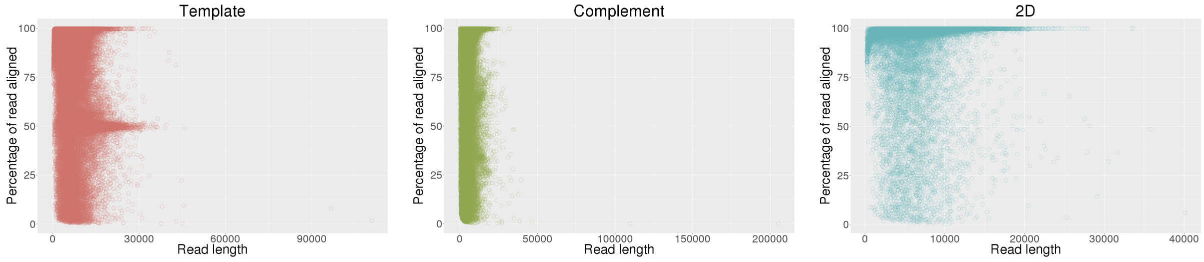

Escherichia coli perfect kmers

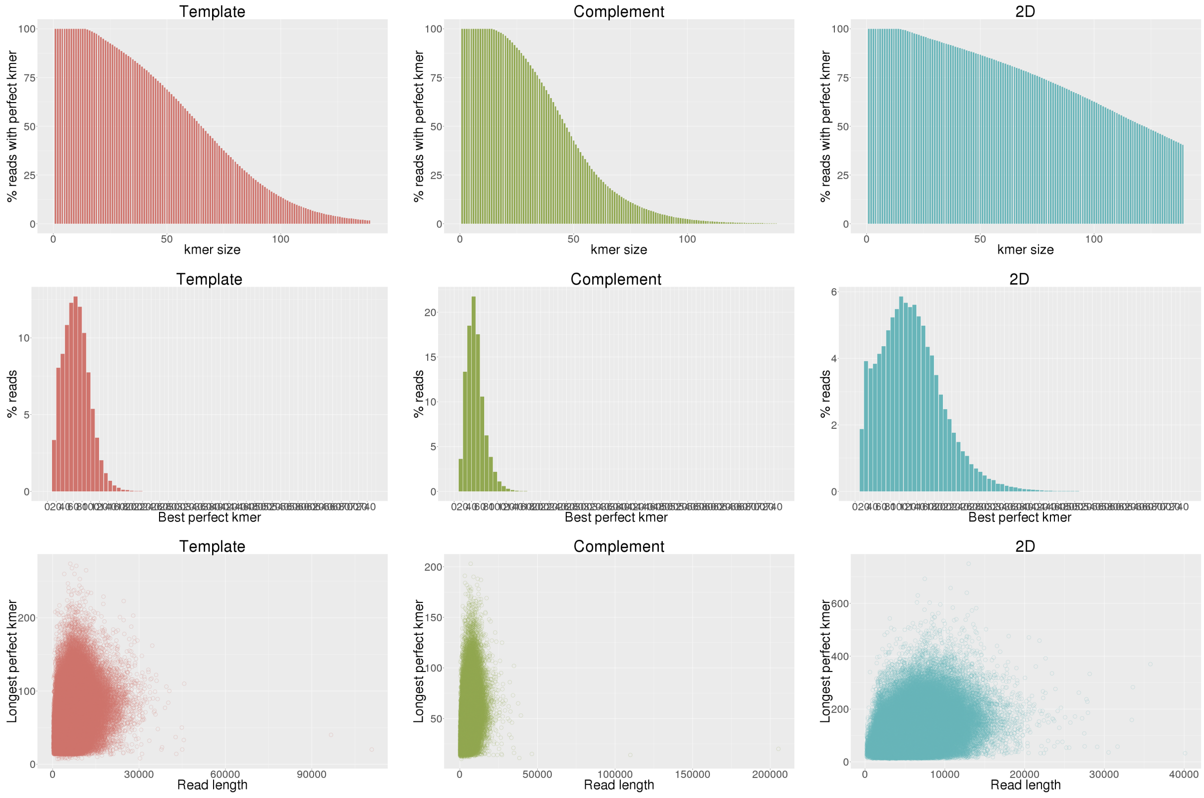

Escherichia coli coverage

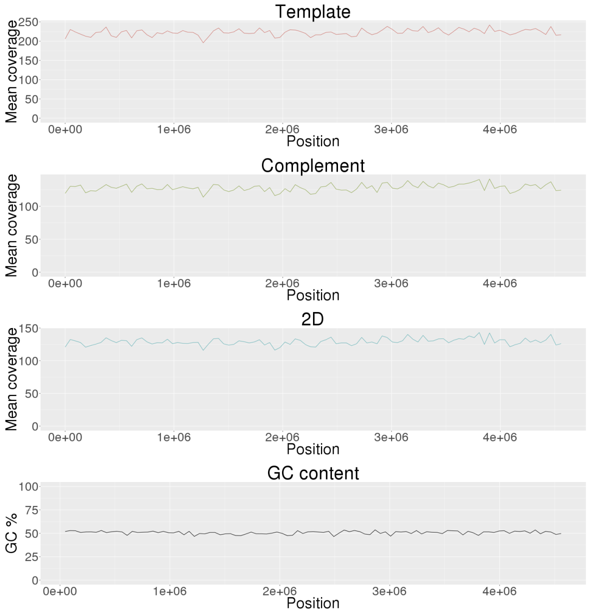

Escherichia coli 5-mer analysis

Under-represented 5-mers

	Template				Complement				2D			
Rank	kmer	Ref %	Read %	Diff %	kmer	Ref %	Read %	Diff %	kmer	Ref %	Read %	Diff %
1	TTTTT	0.251	0.038	-0.213	TTTTT	0.251	0.073	-0.178	TTTTT	0.251	0.135	-0.116
2	AAAAA	0.247	0.095	-0.152	AAAAA	0.247	0.110	-0.137	AAAAA	0.247	0.150	-0.098
3	GTTTT	0.186	0.089	-0.097	CCTGG	0.130	0.065	-0.065	CCTGG	0.130	0.050	-0.081
4	GCCAG	0.280	0.187	-0.093	CCAGG	0.129	0.066	-0.063	CCAGG	0.129	0.054	-0.076
5	TTTTC	0.198	0.130	-0.068	CCCAG	0.111	0.050	-0.060	GCCAG	0.280	0.230	-0.050
6	TTTTG	0.172	0.105	-0.067	TCCAG	0.171	0.117	-0.055	CAGGC	0.183	0.150	-0.033
7	TTTTA	0.147	0.079	-0.067	AAAGA	0.132	0.083	-0.049	GCCTG	0.185	0.155	-0.030
8	ATTTT	0.194	0.127	-0.066	GAAAG	0.130	0.083	-0.047	GTTTT	0.186	0.159	-0.027
9	CCAGG	0.129	0.066	-0.063	ATCCC	0.086	0.041	-0.045	TGCCA	0.182	0.157	-0.025
10	AAAGA	0.132	0.069	-0.063	AGAAA	0.140	0.096	-0.044	CGCCA	0.288	0.263	-0.025

Over-represented 5-mers

	Template				Complement				2D			
Rank	kmer	Ref %	Read %	Diff %	kmer	Ref %	Read %	Diff %	kmer	Ref %	Read %	Diff %
1	CGGCG	0.221	0.419	0.198	CGGCG	0.221	0.294	0.073	CGGCG	0.221	0.278	0.056
2	GCGGC	0.210	0.370	0.161	GCGGC	0.210	0.273	0.063	CGGTG	0.160	0.211	0.051
3	GGCGG	0.190	0.334	0.144	TGCCG	0.220	0.276	0.055	CACGG	0.089	0.134	0.045
4	CGGTG	0.160	0.279	0.119	CGCCG	0.219	0.271	0.052	GGCGG	0.190	0.229	0.039
5	GGCCG	0.069	0.187	0.118	GGCGG	0.190	0.240	0.050	CGCCG	0.219	0.259	0.039
6	CGCCG	0.219	0.337	0.117	AACAA	0.132	0.181	0.049	GCTGG	0.279	0.319	0.039
7	CCGGC	0.141	0.244	0.103	GCAAA	0.196	0.244	0.048	CCGGC	0.141	0.179	0.039
8	GCGGG	0.137	0.240	0.102	AAACA	0.138	0.184	0.046	GCGGC	0.210	0.246	0.037
9	GCCGC	0.209	0.308	0.100	TGCTG	0.250	0.294	0.045	CACCG	0.166	0.202	0.036
10	CGGCC	0.072	0.168	0.096	CAAAC	0.130	0.174	0.044	GGCTG	0.161	0.193	0.032

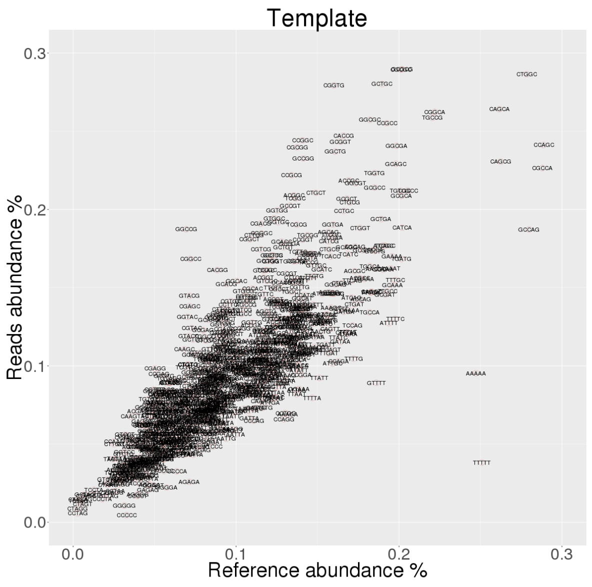

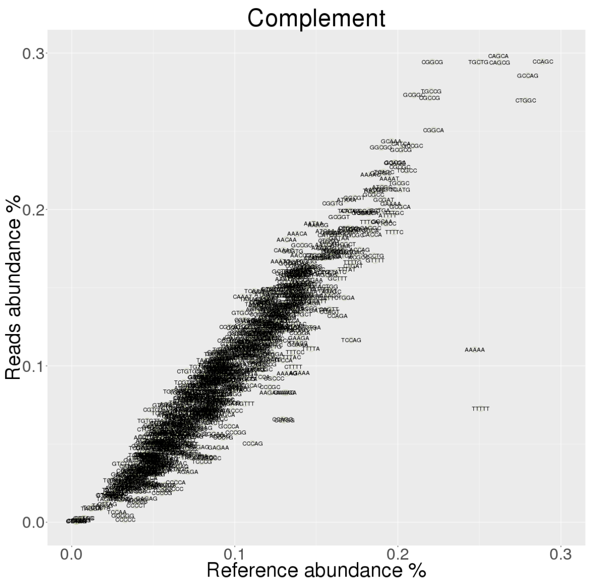

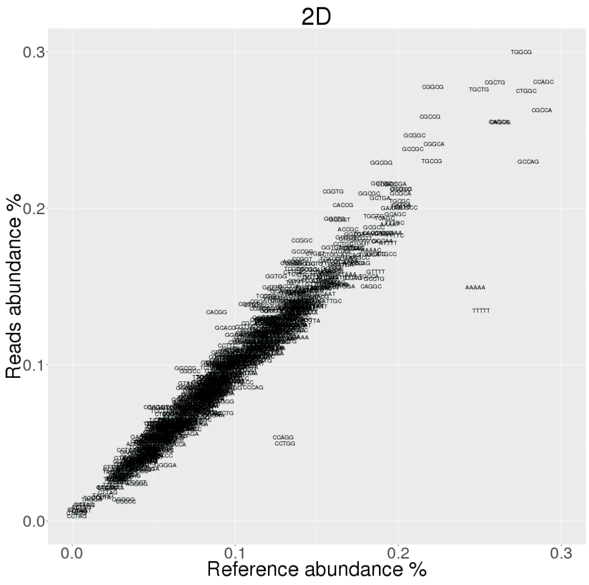

Escherichia coli GC content

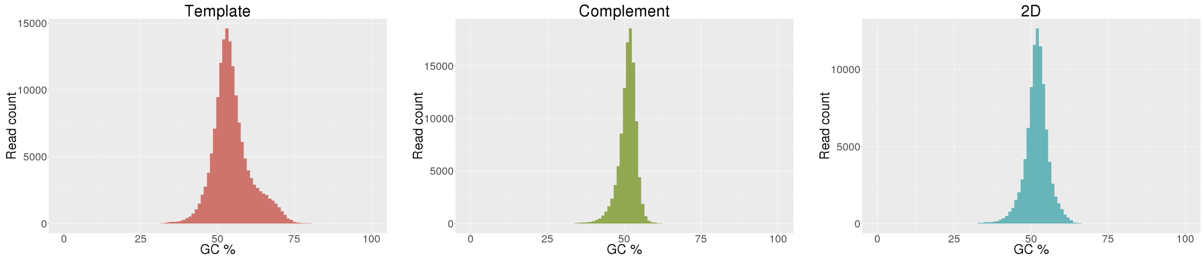

All reference 21mer analysis

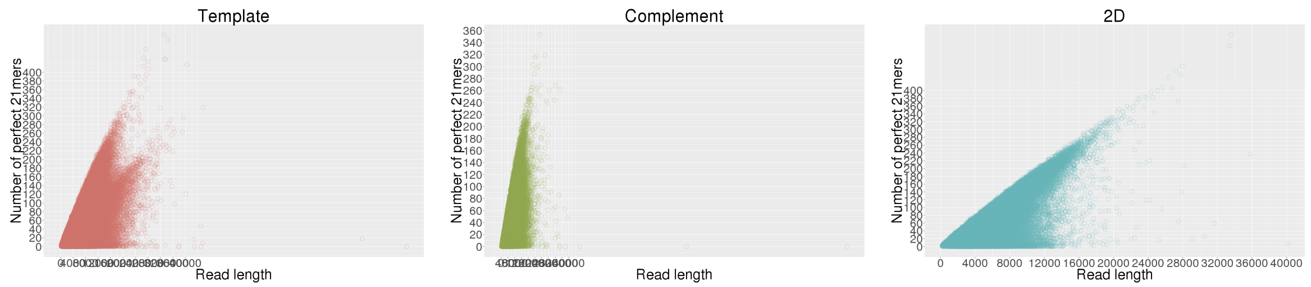

All reference substitutions

		Template substituted %				Complement substituted %				2D substituted %			
		a	c	g	t	a	c	g	t	a	c	g	t
Reference	A	0.00	8.33	17.81	4.59	0.00	5.11	14.98	5.31	0.00	7.55	15.11	5.73
	C	3.36	0.00	3.97	11.43	5.41	0.00	3.89	14.95	5.20	0.00	4.70	11.65
	G	11.17	4.26	0.00	3.36	14.77	4.03	0.00	5.35	11.36	4.85	0.00	5.07
	T	4.62	18.59	8.52	0.00	5.36	15.56	5.29	0.00	5.81	15.57	7.40	0.00

Kmer motifs before errors

3-mer error motif analysis

	Template			Complement			2D			
Rank	Insertion	Deletion	Substitution	Insertion	Deletion	Substitution	Insertion	Deletion	Substitution	
1	TTC (3.33%)	CGC (3.36%)	CGC (3.96%)	CGC (3.22%)	CGC (3.06%)	GCC (3.33%)	TTC (3.02%)	TTT (3.02%)	GCC (4.20%)	Most common
2	CGC (2.90%)	TTC (3.04%)	GCC (3.59%)	ATC (2.99%)	TTT (2.93%)	CGC (3.23%)	CGC (3.00%)	CGC (2.95%)	CGC (3.54%)	
3	ATC (2.81%)	GCC (2.87%)	ATC (3.14%)	TTT (2.83%)	ATC (2.84%)	ATC (3.19%)	TTT (2.81%)	ATC (2.83%)	ACC (3.39%)	
4	TTT (2.71%)	ATC (2.79%)	TGC (3.12%)	TTC (2.74%)	TTC (2.53%)	AAC (2.83%)	GCC (2.69%)	GCC (2.83%)	TTT (3.18%)	
5	GCC (2.61%)	TTT (2.73%)	ACC (3.10%)	GCG (2.55%)	GGC (2.53%)	TGC (2.82%)	ATC (2.68%)	TTC (2.70%)	ATC (3.14%)	
6	TGC (2.35%)	GCG (2.73%)	GGC (2.98%)	TGC (2.48%)	GCC (2.52%)	TTT (2.80%)	TGC (2.61%)	ACC (2.60%)	TGC (3.06%)	
7	ACC (2.31%)	GGC (2.72%)	TTC (2.93%)	GGC (2.48%)	GCG (2.49%)	ACC (2.66%)	GGC (2.40%)	GGC (2.54%)	TTC (2.91%)	
8	GCG (2.25%)	ACC (2.55%)	AAC (2.80%)	GCC (2.41%)	TGC (2.34%)	TTC (2.64%)	ACC (2.27%)	ATT (2.32%)	TCC (2.78%)	
9	GGC (2.19%)	TGC (2.29%)	AGC (2.59%)	AAC (2.33%)	ATT (2.33%)	GGC (2.45%)	AGC (2.18%)	GCG (2.27%)	GGC (2.67%)	
10	AAC (2.08%)	AGC (2.13%)	TTT (2.49%)	ATT (2.26%)	AAA (2.30%)	AAA (2.44%)	GCG (2.13%)	TGC (2.27%)	AAC (2.52%)	
	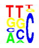	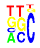	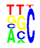	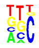	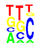	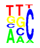	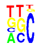	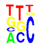	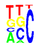	
-10	TTA (1.10%)	AGA (0.97%)	GTA (0.89%)	CCT (0.95%)	GGA (0.95%)	TGT (0.84%)	TAA (1.02%)	GGA (0.95%)	ACT (0.85%)	Least common
-9	AGT (0.92%)	GGA (0.90%)	GAG (0.87%)	AGA (0.94%)	CCC (0.90%)	TCT (0.84%)	GTA (0.98%)	AGA (0.94%)	GAG (0.83%)	
-8	GAG (0.91%)	GTA (0.87%)	CCT (0.84%)	CCC (0.94%)	GAG (0.79%)	GAG (0.81%)	AGT (0.95%)	GAG (0.93%)	GTA (0.82%)	
-7	TAA (0.90%)	AGT (0.82%)	ACT (0.82%)	GTA (0.81%)	GTA (0.78%)	ACT (0.67%)	GAG (0.94%)	AGT (0.87%)	TAT (0.81%)	
-6	AGA (0.82%)	TAA (0.81%)	GGG (0.65%)	AGG (0.79%)	AGG (0.77%)	GGA (0.64%)	AGA (0.80%)	GTA (0.84%)	GGG (0.73%)	
-5	GGA (0.82%)	AGG (0.78%)	AGA (0.64%)	GGA (0.78%)	AGT (0.73%)	GGG (0.63%)	AGG (0.77%)	CCT (0.83%)	AGA (0.65%)	
-4	AGG (0.76%)	CCT (0.74%)	AGT (0.62%)	AGT (0.76%)	CCT (0.68%)	CCT (0.60%)	GGA (0.76%)	AGG (0.75%)	AGT (0.57%)	
-3	GGG (0.52%)	GGG (0.62%)	GGA (0.60%)	GGG (0.52%)	GGG (0.55%)	AGT (0.50%)	GGG (0.59%)	GGG (0.61%)	GGA (0.55%)	
-2	CTA (0.47%)	TAG (0.41%)	TAG (0.42%)	TAG (0.37%)	TAG (0.33%)	TAG (0.40%)	TAG (0.43%)	CTA (0.40%)	TAG (0.39%)	
-1	TAG (0.45%)	CTA (0.36%)	CTA (0.30%)	CTA (0.27%)	CTA (0.27%)	CTA (0.26%)	CTA (0.40%)	TAG (0.39%)	CTA (0.30%)	
	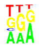	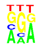	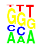	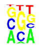	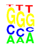	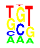	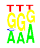	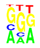	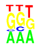	

Kmer space for 3-mers: 64 Random chance for any given 3-mer: 1.56%

4-mer error motif analysis

Rank	Template			Complement			2D			
	Insertion	Deletion	Substitution	Insertion	Deletion	Substitution	Insertion	Deletion	Substitution	
1	TTTC (1.14%)	CGCC (1.22%)	CGCC (1.55%)	CAGC (1.06%)	CAGC (1.11%)	CGCC (1.40%)	CGCC (1.09%)	CGCC (1.15%)	CGCC (1.74%)	Most common
2	CGCC (1.11%)	GCGC (1.15%)	GCGC (1.26%)	GCGC (1.05%)	CGCC (1.08%)	CAGC (1.09%)	TTTC (1.00%)	GCGC (1.00%)	TGCC (1.16%)	
3	GCGC (0.96%)	CGGC (1.04%)	CAGC (1.18%)	CGCC (1.03%)	GCGC (1.03%)	TGCC (1.01%)	GCGC (0.97%)	CAGC (0.98%)	GCGC (1.12%)	
4	CAGC (0.88%)	TTTC (1.04%)	CGGC (1.09%)	CGGC (0.91%)	CGGC (0.93%)	GCGC (1.00%)	CAGC (0.96%)	CGGC (0.91%)	TTTT (1.07%)	
5	CTTC (0.88%)	CAGC (1.02%)	CATC (1.01%)	TTTC (0.89%)	TTTC (0.90%)	CATC (0.96%)	TTTT (0.84%)	TTTT (0.89%)	CAGC (1.05%)	
6	GCTG (0.82%)	GGCG (0.91%)	CACC (0.99%)	CATC (0.86%)	TGGC (0.87%)	AAAC (0.93%)	TGGC (0.82%)	TTTC (0.88%)	CACC (1.05%)	
7	CATC (0.82%)	CATC (0.89%)	ACGC (0.95%)	TGGC (0.86%)	TTTT (0.85%)	TGGC (0.88%)	CGGC (0.81%)	CACC (0.83%)	AACC (1.03%)	
8	CGGC (0.79%)	CACC (0.83%)	CCGC (0.94%)	TTTT (0.85%)	CATC (0.85%)	ATCA (0.86%)	CTTC (0.80%)	CATC (0.83%)	TTCC (1.01%)	
9	TTTT (0.78%)	CCGC (0.80%)	TGGC (0.94%)	ACGC (0.82%)	ATTT (0.81%)	AACC (0.85%)	CTGC (0.77%)	ATTT (0.82%)	ATCC (0.99%)	
10	CACC (0.76%)	TGGC (0.79%)	CTGC (0.92%)	GGCG (0.80%)	GGCG (0.80%)	CGGC (0.85%)	TGCC (0.76%)	GTTT (0.79%)	TTTC (0.91%)	
	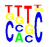	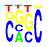	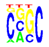	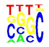	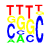	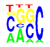	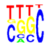	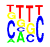	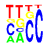	
-10	CTTA (0.12%)	CTTA (0.10%)	TAGT (0.10%)	TAGT (0.10%)	TAGT (0.09%)	GAGT (0.08%)	TTAG (0.11%)	CCCT (0.11%)	CTAA (0.09%)	Least common
-9	GGGA (0.11%)	ACTA (0.10%)	GGGA (0.09%)	GGGA (0.09%)	CCCT (0.09%)	CCCT (0.08%)	GGGA (0.11%)	TTAG (0.10%)	GGGA (0.09%)	
-8	TCTA (0.11%)	TTAG (0.10%)	GGGG (0.09%)	CTAA (0.09%)	CTAA (0.09%)	GGGG (0.07%)	ACTA (0.11%)	ACTA (0.10%)	GAGT (0.09%)	
-7	AGGG (0.10%)	CCCT (0.09%)	ACTA (0.08%)	TAGA (0.08%)	TAGA (0.09%)	ACTA (0.07%)	GGGG (0.10%)	TAGA (0.10%)	ACTA (0.08%)	
-6	CTAA (0.09%)	TCTA (0.09%)	GAGT (0.08%)	TCTA (0.07%)	ACTA (0.07%)	TAGA (0.07%)	CTAA (0.09%)	CTAA (0.10%)	TAGT (0.07%)	
-5	TAGA (0.08%)	GGGG (0.09%)	TAGG (0.07%)	ACTA (0.07%)	GGGG (0.07%)	TAGG (0.06%)	TCTA (0.09%)	GGGG (0.10%)	TCTA (0.06%)	
-4	CCTA (0.08%)	CTAA (0.08%)	TCTA (0.06%)	GGGG (0.07%)	TCTA (0.06%)	TAGT (0.06%)	TAGA (0.08%)	TCTA (0.09%)	TAGG (0.06%)	
-3	GGGG (0.08%)	TAGG (0.06%)	TAGA (0.06%)	TAGG (0.05%)	TAGG (0.05%)	TCTA (0.05%)	TAGG (0.06%)	CCTA (0.06%)	TAGA (0.05%)	
-2	TAGG (0.07%)	CCTA (0.05%)	CCTA (0.05%)	CCTA (0.03%)	CCTA (0.03%)	CCTA (0.04%)	CCTA (0.06%)	TAGG (0.06%)	CCTA (0.05%)	
-1	CTAG (0.02%)	CTAG (0.01%)	CTAG (0.01%)	CTAG (0.00%)	CTAG (0.00%)	CTAG (0.00%)	CTAG (0.01%)	CTAG (0.01%)	CTAG (0.01%)	
	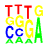	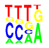	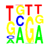	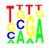	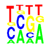	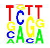	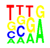	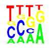	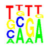	

Kmer space for 4-mers: 256 Random chance for any given 4-mer: 0.39%

5-mer error motif analysis

Rank	Template			Complement			2D			
	Insertion	Deletion	Substitution	Insertion	Deletion	Substitution	Insertion	Deletion	Substitution	
1	ATTTC (0.37%)	GCGGC (0.39%)	CCAGC (0.46%)	CCAGC (0.40%)	CCAGC (0.42%)	CCAGC (0.42%)	CCAGC (0.35%)	CCAGC (0.35%)	CCGCC (0.49%)	Most common
2	CGCCA (0.36%)	CCAGC (0.36%)	CCGCC (0.42%)	CTGGC (0.36%)	CTGGC (0.36%)	TCGCC (0.42%)	CGCCA (0.34%)	GCGGC (0.34%)	TCGCC (0.49%)	
3	GTTTC (0.33%)	GGCGC (0.35%)	TCGCC (0.41%)	GCGGC (0.32%)	GCGGC (0.36%)	CTGGC (0.38%)	CTGGC (0.32%)	TCGCC (0.33%)	CAGCC (0.44%)	
4	CCAGC (0.33%)	TCGCC (0.33%)	CTGGC (0.40%)	TGGCG (0.32%)	CGCCA (0.33%)	CCGCC (0.35%)	TCGCC (0.30%)	CGCCA (0.33%)	GCGCC (0.42%)	
5	TCGCC (0.29%)	CCGCC (0.32%)	GCGGC (0.40%)	CGCCA (0.31%)	TGGCG (0.32%)	GCGCC (0.35%)	ATTTC (0.29%)	GGCGC (0.32%)	CCAGC (0.40%)	
6	CCGCC (0.29%)	TTTTC (0.32%)	GCGCC (0.38%)	CAGCG (0.31%)	TCGCC (0.31%)	CGCCA (0.33%)	GCGGC (0.29%)	CTGGC (0.31%)	ACGCC (0.39%)	
7	GCGGC (0.29%)	TGGCG (0.32%)	GCCGC (0.36%)	AACGC (0.30%)	ATTTT (0.30%)	GCGGC (0.32%)	CCGCC (0.29%)	CCGCC (0.30%)	TTGCC (0.37%)	
8	CGCGC (0.28%)	CTGGC (0.31%)	ACGCC (0.35%)	TGATG (0.29%)	TGATG (0.29%)	ACGCC (0.32%)	GTTTC (0.27%)	ATTTT (0.28%)	CTGGC (0.36%)	
9	CTGGC (0.28%)	CGCGC (0.29%)	CAGCC (0.34%)	CGCGC (0.29%)	GGCGC (0.29%)	TTGCC (0.32%)	CAGCA (0.26%)	TTATT (0.28%)	TCACC (0.34%)	
10	TTTTC (0.27%)	CGGCG (0.29%)	CGCGC (0.34%)	ATTTT (0.29%)	GCAGC (0.28%)	TCATC (0.31%)	ACGCC (0.26%)	TCACC (0.27%)	AAACC (0.34%)	
	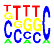	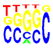	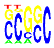	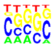	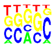	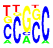	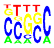	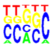	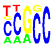	
-10	CCTGG (0.01%)	CCAGG (0.01%)	CTAGC (0.01%)	CCCCC (0.00%)	TCCTA (0.00%)	CCCCC (0.00%)	TAGGG (0.01%)	TCCTA (0.01%)	TCCTA (0.01%)	Least common
-9	GCTAG (0.01%)	GGGGG (0.01%)	TAGGA (0.01%)	GGGGG (0.00%)	GGGGG (0.00%)	GGGGG (0.00%)	TCCTA (0.01%)	TAGGA (0.01%)	CTAGC (0.01%)	
-8	CTAGC (0.01%)	CTAGT (0.00%)	CTAGT (0.01%)	GCTAG (0.00%)	CTAGC (0.00%)	GCTAG (0.00%)	CTAGC (0.01%)	CTAGC (0.00%)	TAGGA (0.00%)	
-7	GGGGG (0.00%)	CTAGC (0.00%)	GGGGG (0.01%)	CTAGC (0.00%)	GCTAG (0.00%)	CTAGC (0.00%)	GCTAG (0.00%)	CTAGT (0.00%)	GCTAG (0.00%)	
-6	CTAGT (0.00%)	GCTAG (0.00%)	ACTAG (0.00%)	CTAGT (0.00%)	CTAGT (0.00%)	ACTAG (0.00%)	CTAGT (0.00%)	GCTAG (0.00%)	CTAGT (0.00%)	
-5	ACTAG (0.00%)	ACTAG (0.00%)	GCTAG (0.00%)	CTAGA (0.00%)	CTAGA (0.00%)	CTAGA (0.00%)	ACTAG (0.00%)	ACTAG (0.00%)	ACTAG (0.00%)	
-4	CTAGA (0.00%)	CTAGA (0.00%)	TCTAG (0.00%)	ACTAG (0.00%)	ACTAG (0.00%)	TCTAG (0.00%)	TCTAG (0.00%)	CTAGA (0.00%)	TCTAG (0.00%)	
-3	CCTAG (0.00%)	TCTAG (0.00%)	CTAGA (0.00%)	TCTAG (0.00%)	TCTAG (0.00%)	CTAGT (0.00%)	CTAGA (0.00%)	TCTAG (0.00%)	CTAGA (0.00%)	
-2	TCTAG (0.00%)	CCTAG (0.00%)	CCTAG (0.00%)	CTAGG (0.00%)	CTAGG (0.00%)	CTAGG (0.00%)	CCTAG (0.00%)	CTAGG (0.00%)	CCTAG (0.00%)	
-1	CTAGG (0.00%)	CTAGG (0.00%)	CTAGG (0.00%)	CCTAG (0.00%)	CCTAG (0.00%)	CCTAG (0.00%)	CTAGG (0.00%)	CCTAG (0.00%)	CTAGG (0.00%)	
	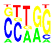	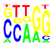	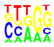	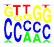	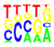	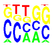	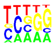	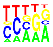	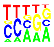	

Kmer space for 5-mers: 1024 Random chance for any given 5-mer: 0.10%
