# Supplementary material for: MinION Analysis and Reference Consortium: Phase 2 data release and analysis of R9.0 chemistry
Source: F1000Res. 2017 May 31;6:760. [Version 1] doi: 10.12688/f1000research.11354.1 (PMC5538040; doi:10.12688/f1000research.11354.1)
Supplement: Supplementary file 3 [file f1000research-6-12257-s0002.tgz › 5d2560fe-dd7a-44d1-b265-e3522e742149.zip!/SI File 3. NanoOK experiment reports/P2-Lab7-R1-2D_nanook_bwa_passonly.pdf]

NanoOK report for Nott_R92_2D

Pass and fail counts

Type	Pass	Fail
Template	80635	0
Complement	80635	0
2D	80635	0

Read lengths

Type	NumReads	TotalBases	Mean	Longest	Shortest	N50	N50Count	N90	N90Count
Template	80635	546284665	6774.78	34594	208	7948	26850	4532	61461
Complement	80635	481298865	5968.86	34884	152	7074	26156	3903	61018
2D	80635	533824143	6620.25	35764	158	7821	26523	4383	61210

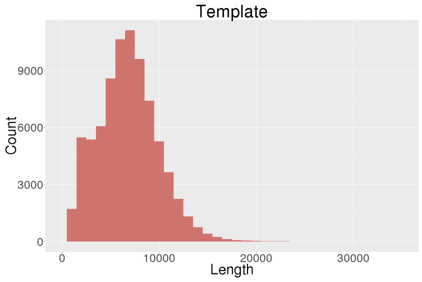

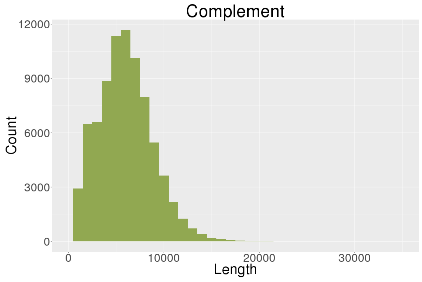

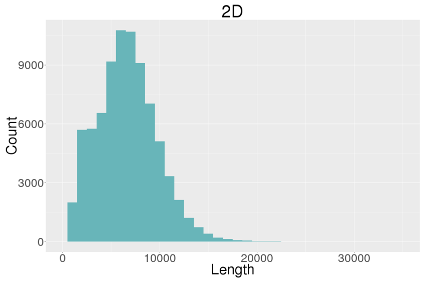

Template alignments

Number of reads	80635	
Number of reads with alignments	80543	(99.89%)
Number of reads without alignments	92	(0.11%)

ID	Size	Number of Reads	% of Reads	Mean read length	Aligned bases	Mean coverage	Longest Perf Kmer
Control sequence	3560	1503	1.86	2160.01	3061759	860.04	194
Escherichia coli	4641652	79040	98.02	6861.54	560109410	120.67	271

Complement alignments

Number of reads	80635	
Number of reads with alignments	80361	(99.66%)
Number of reads without alignments	274	(0.34%)

ID	Size	Number of Reads	% of Reads	Mean read length	Aligned bases	Mean coverage	Longest Perf Kmer
Control sequence	3560	1486	1.84	1799.80	2737433	768.94	146
Escherichia coli	4641652	78875	97.82	6047.57	509573939	109.78	203

2D alignments

Number of reads	80635	
Number of reads with alignments	80561	(99.91%)
Number of reads without alignments	74	(0.09%)

ID	Size	Number of Reads	% of Reads	Mean read length	Aligned bases	Mean coverage	Longest Perf Kmer
Control sequence	3560	1509	1.87	1922.41	2926334	822.00	403
Escherichia coli	4641652	79052	98.04	6709.04	547589559	117.97	750

Control sequence error analysis

	Template	Complement	2D
Overall base identity (excluding indels)	79.65%	79.62%	91.35%
Aligned base identity (excluding indels)	92.71%	91.47%	96.76%
Identical bases per 100 aligned bases (including indels)	84.46%	77.79%	90.55%
Inserted bases per 100 aligned bases (including indels)	2.52%	1.97%	2.04%
Deleted bases per 100 aligned bases (including indels)	6.39%	12.98%	4.37%
Substitutions per 100 aligned bases (including indels)	6.64%	7.25%	3.03%
Mean insertion size	1.50	1.39	1.62
Mean deletion size	1.65	2.29	1.81

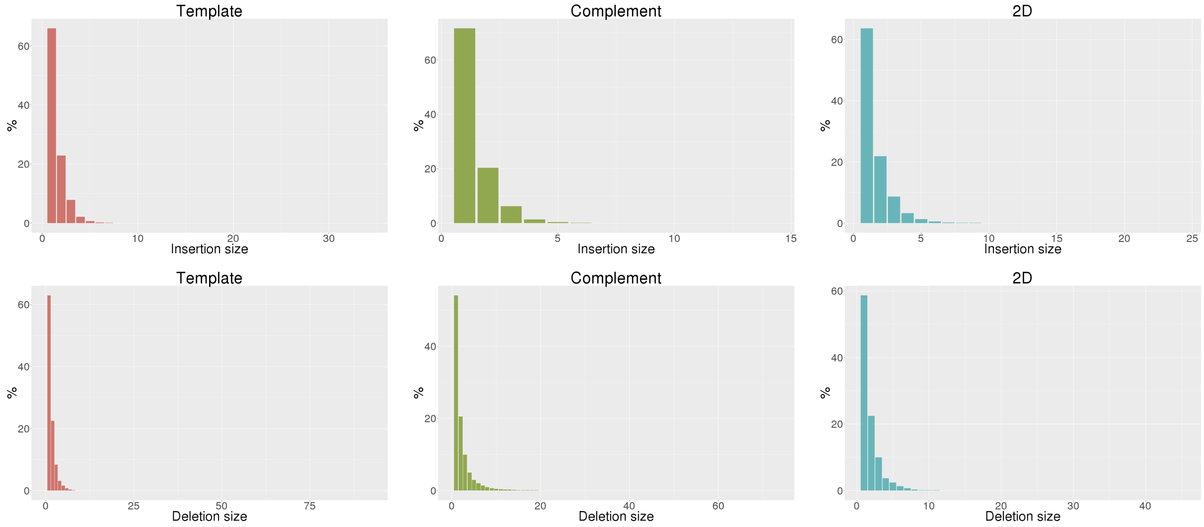

Control sequence read identity

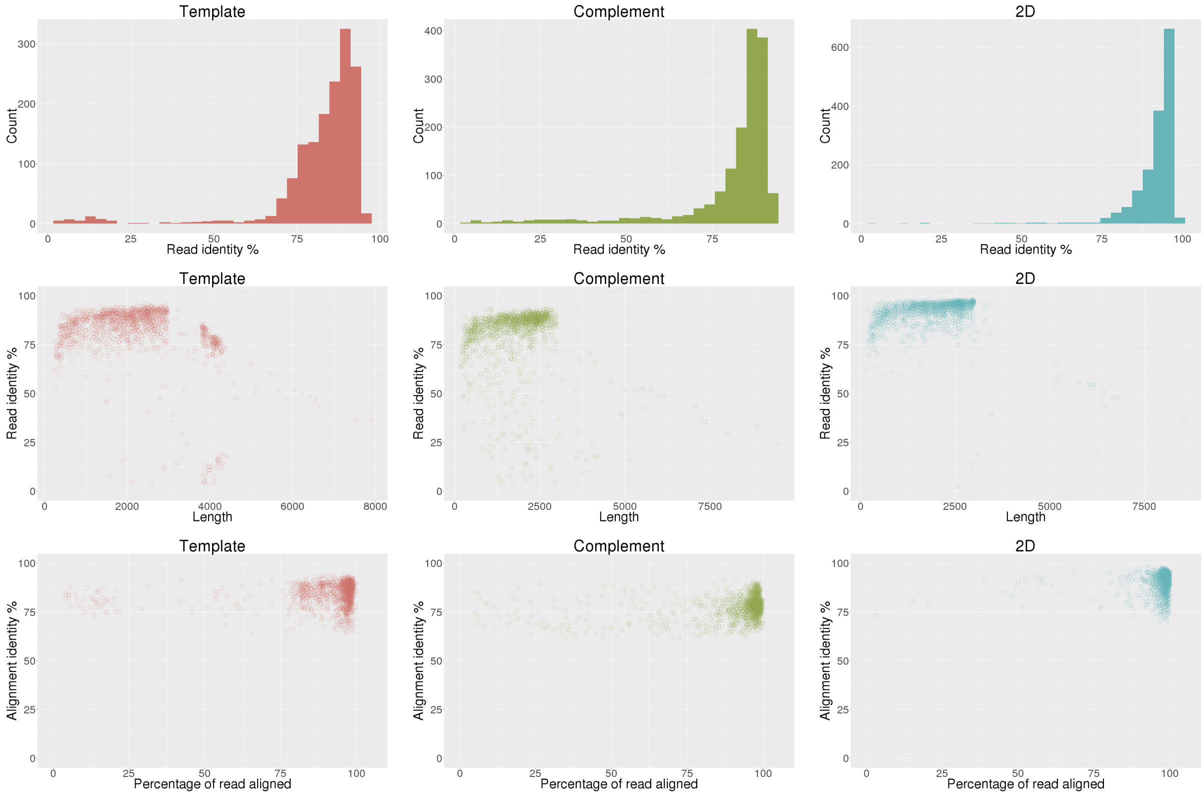

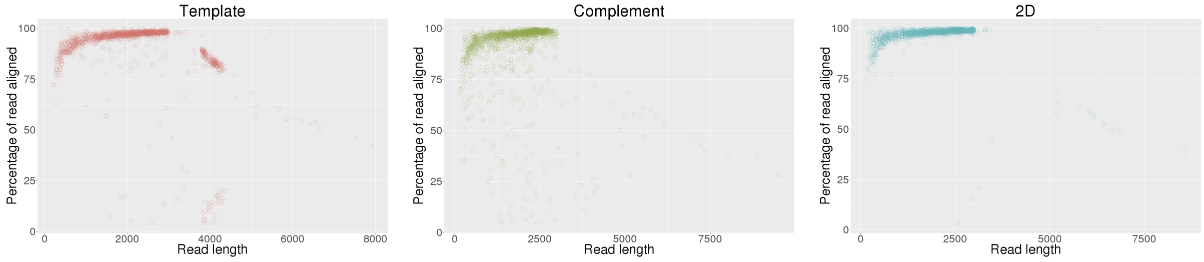

Control sequence perfect kmers

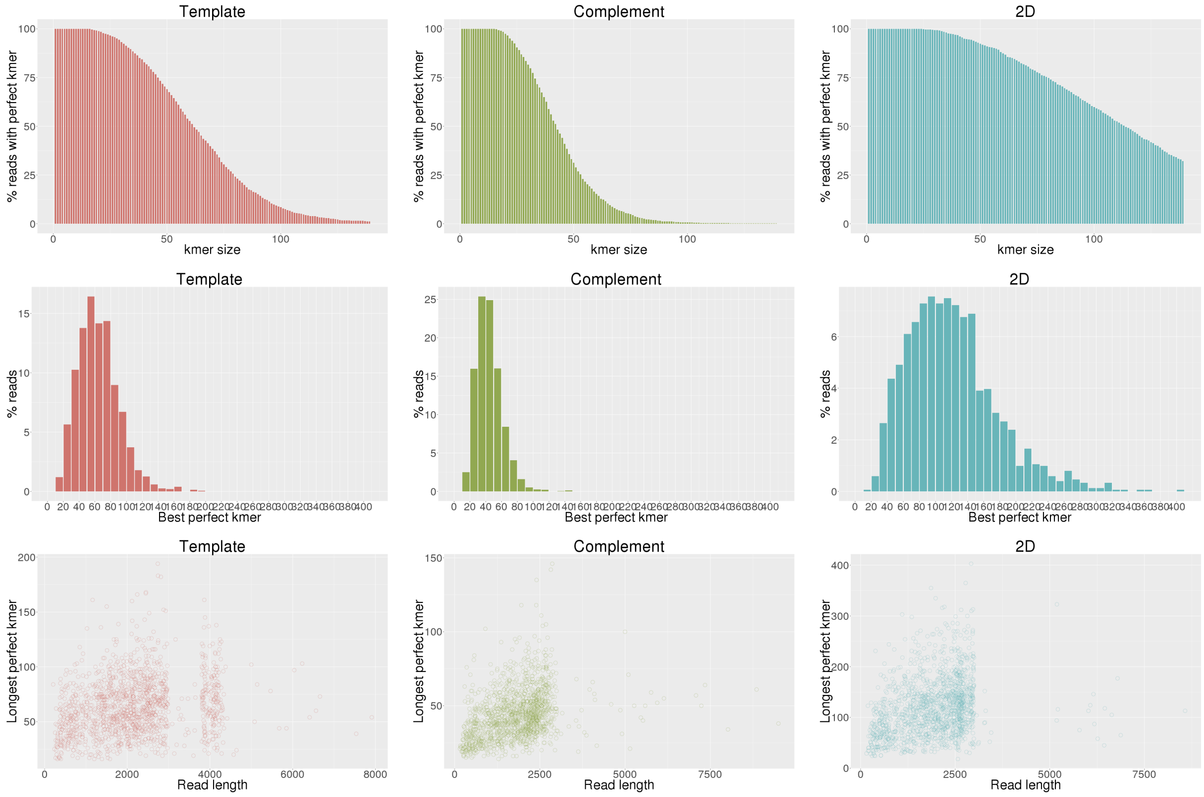

Control sequence coverage

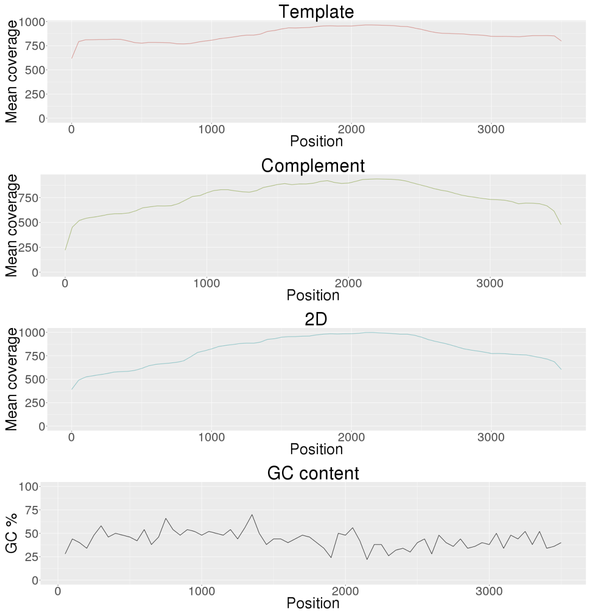

Control sequence 5-mer analysis

Under-represented 5-mers

	Template				Complement				2D			
Rank	kmer	Ref %	Read %	Diff %	kmer	Ref %	Read %	Diff %	kmer	Ref %	Read %	Diff %
1	TTTTT	0.759	0.103	-0.656	TTTTT	0.759	0.116	-0.643	TTTTT	0.759	0.216	-0.543
2	AAAAA	0.478	0.176	-0.301	AAAAA	0.478	0.170	-0.308	AAAAA	0.478	0.219	-0.258
3	TGATG	0.393	0.204	-0.189	CTGAT	0.309	0.171	-0.138	TGATG	0.393	0.226	-0.167
4	GCAAT	0.309	0.155	-0.154	TGATG	0.393	0.257	-0.136	CTGAT	0.309	0.147	-0.162
5	CTGAT	0.309	0.160	-0.149	AGTAG	0.169	0.036	-0.133	GATGT	0.309	0.172	-0.137
6	GATGC	0.281	0.137	-0.144	GAGCA	0.253	0.121	-0.132	GCTGA	0.281	0.148	-0.133
7	GATGT	0.309	0.166	-0.143	CTTTT	0.253	0.124	-0.128	TGGTT	0.253	0.127	-0.126
8	GCTGA	0.281	0.145	-0.136	GATGT	0.309	0.185	-0.124	GCAAT	0.309	0.185	-0.124
9	TTATC	0.309	0.178	-0.131	AGTAA	0.253	0.136	-0.117	GATGC	0.281	0.166	-0.114
10	TGGTT	0.253	0.125	-0.128	GATGC	0.281	0.169	-0.112	TGTGA	0.225	0.111	-0.114

Over-represented 5-mers

	Template				Complement				2D			
Rank	kmer	Ref %	Read %	Diff %	kmer	Ref %	Read %	Diff %	kmer	Ref %	Read %	Diff %
1	CATCA	0.112	0.287	0.175	CAGCA	0.056	0.225	0.169	CATCA	0.112	0.291	0.179
2	ATCAG	0.056	0.197	0.141	CATCA	0.112	0.262	0.149	TCAGC	0.028	0.186	0.158
3	CAGCA	0.056	0.185	0.129	AACCA	0.028	0.164	0.136	ATCAG	0.056	0.194	0.137
4	TCAGC	0.028	0.156	0.128	GCGAA	0.028	0.156	0.128	AACCA	0.028	0.159	0.131
5	CATCT	0.000	0.122	0.122	TCAGC	0.028	0.153	0.125	GCATC	0.084	0.206	0.122
6	GCATC	0.084	0.202	0.118	ATAAA	0.112	0.236	0.124	CAGCA	0.056	0.177	0.121
7	CGTTC	0.028	0.146	0.117	ATCAG	0.056	0.179	0.123	CATCT	0.000	0.115	0.115
8	GTTTA	0.084	0.199	0.114	AATAA	0.197	0.312	0.116	TCACA	0.028	0.141	0.113
9	AACCA	0.028	0.140	0.112	AAACG	0.112	0.223	0.110	GACGA	0.000	0.103	0.103
10	TACTT	0.000	0.109	0.109	GGCGA	0.028	0.130	0.102	TATTA	0.140	0.242	0.102

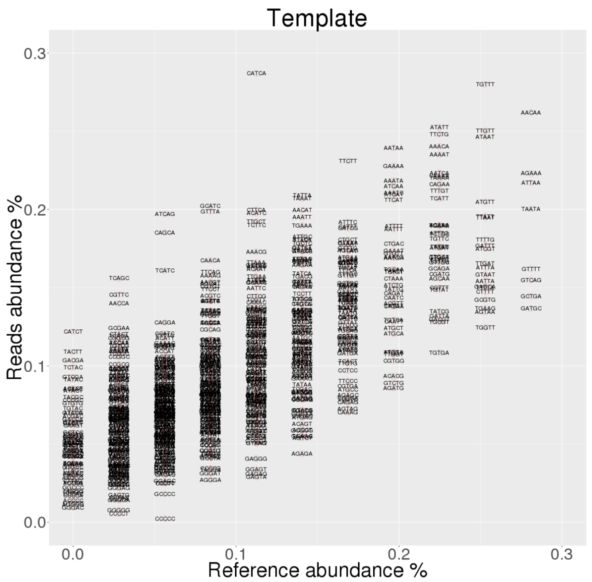

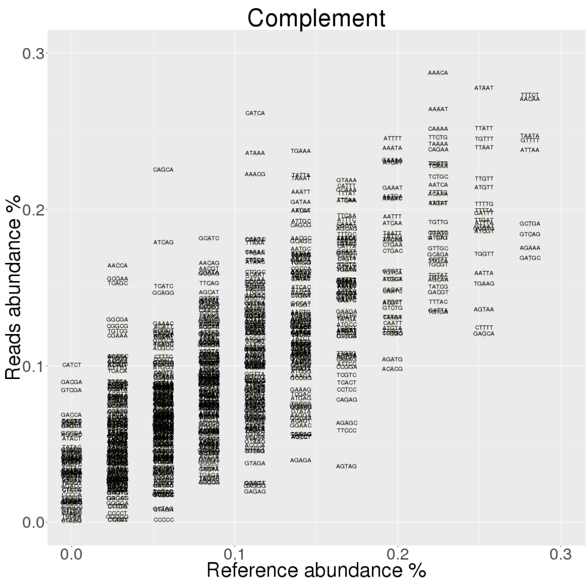

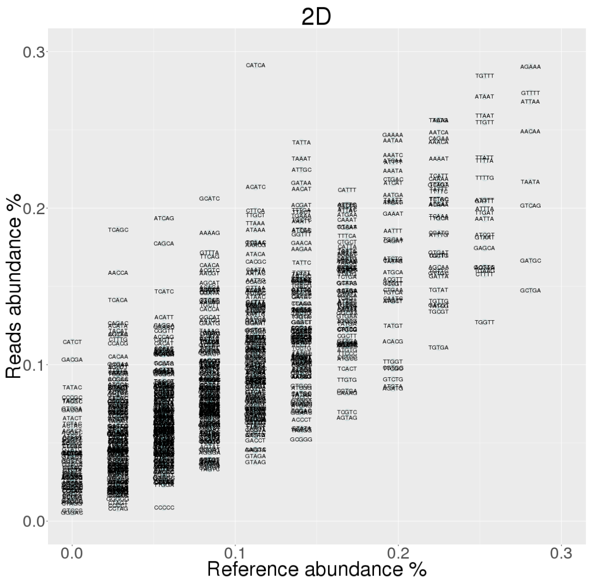

Control sequence GC content

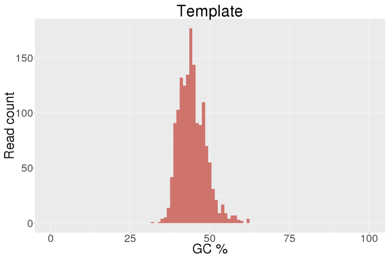

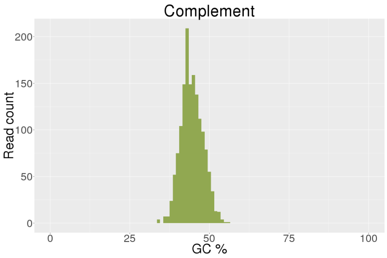

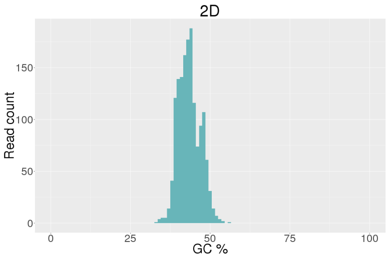

Escherichia coli error analysis

	Template	Complement	2D
Overall base identity (excluding indels)	86.02%	82.79%	92.88%
Aligned base identity (excluding indels)	91.80%	91.41%	96.28%
Identical bases per 100 aligned bases (including indels)	83.29%	77.50%	89.96%
Inserted bases per 100 aligned bases (including indels)	2.40%	1.98%	2.15%
Deleted bases per 100 aligned bases (including indels)	6.87%	13.23%	4.42%
Substitutions per 100 aligned bases (including indels)	7.44%	7.29%	3.48%
Mean insertion size	1.49	1.41	1.63
Mean deletion size	1.62	2.34	1.74

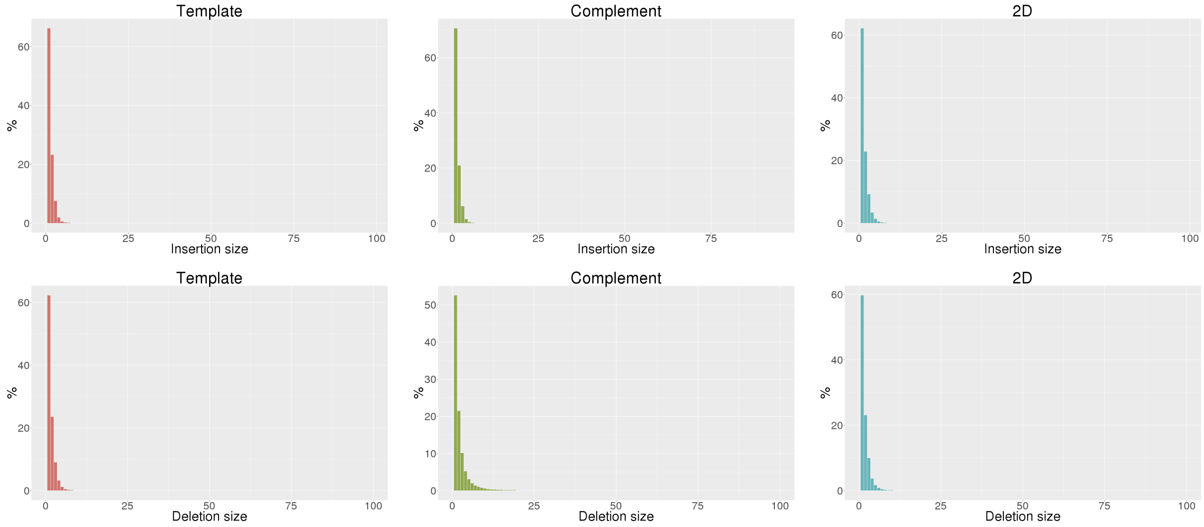

Escherichia coli read identity

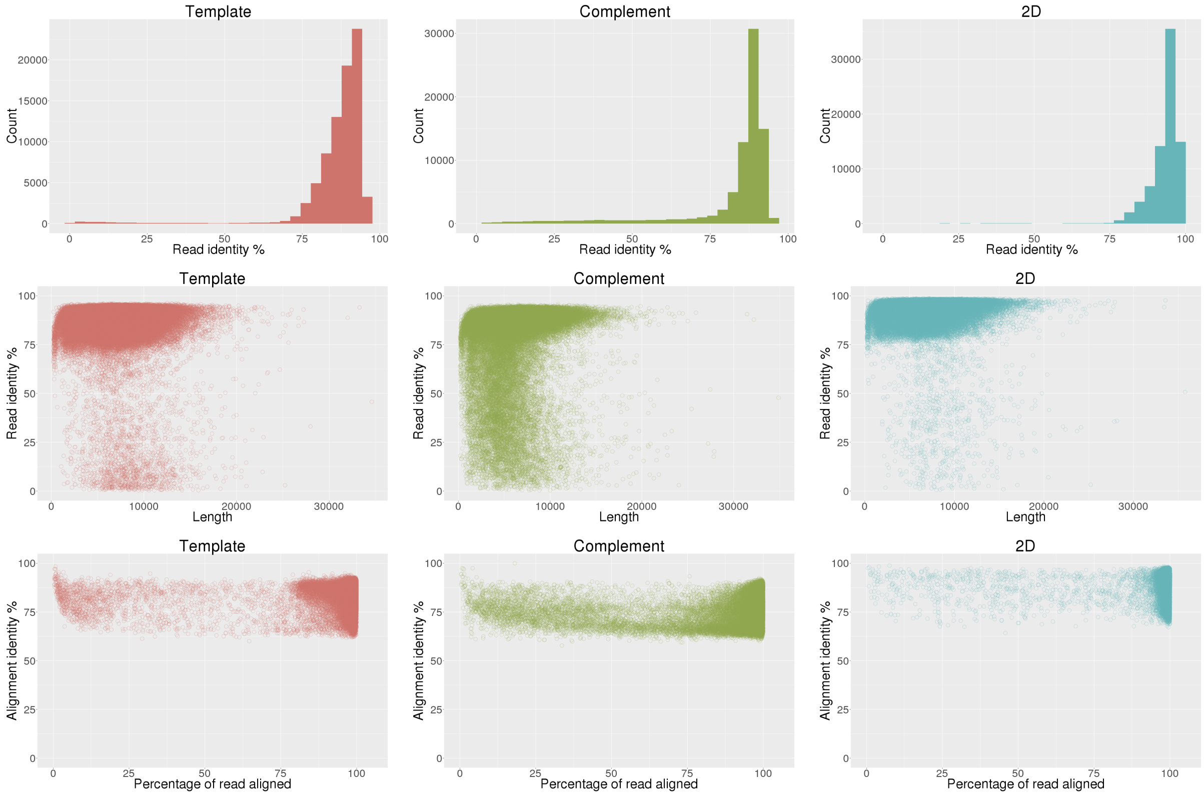

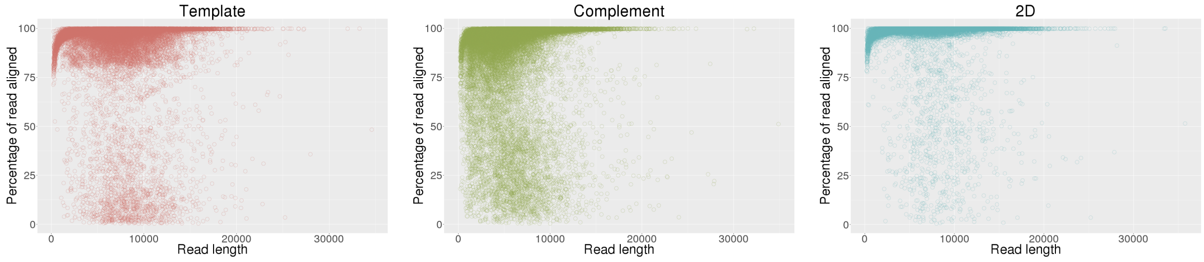

Escherichia coli perfect kmers

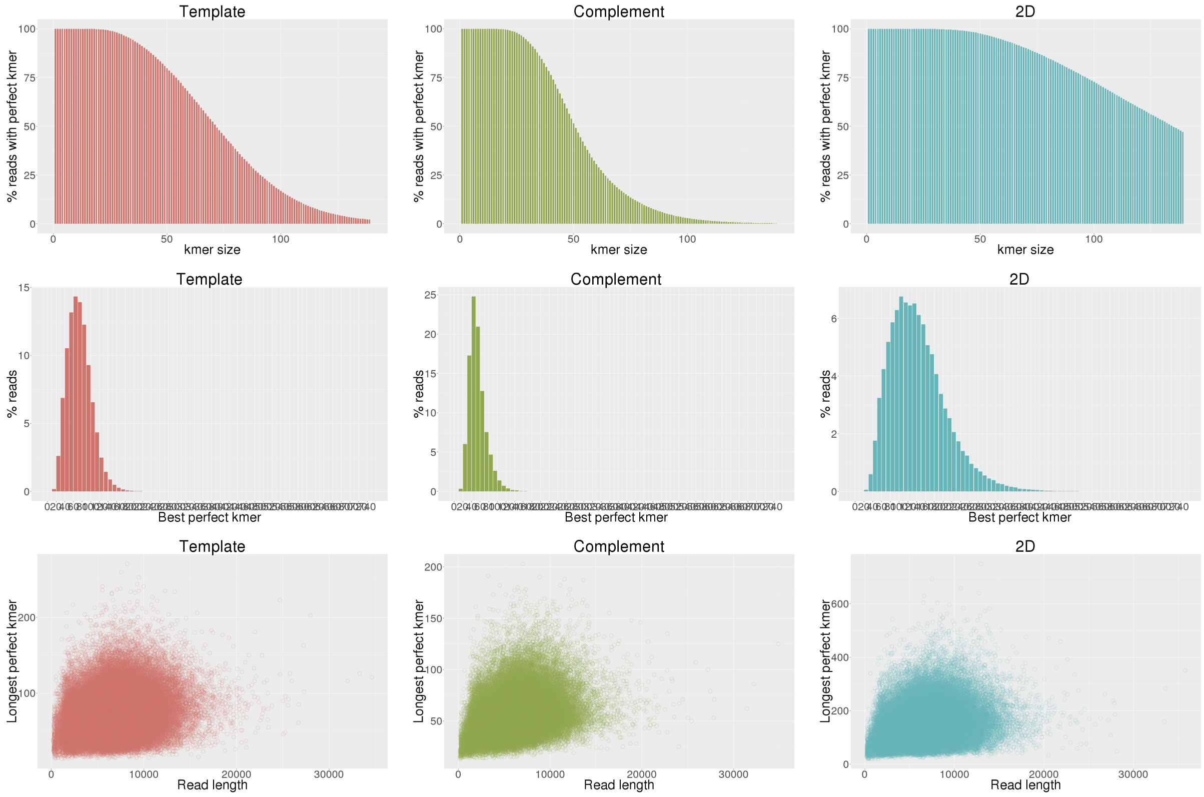

Escherichia coli coverage

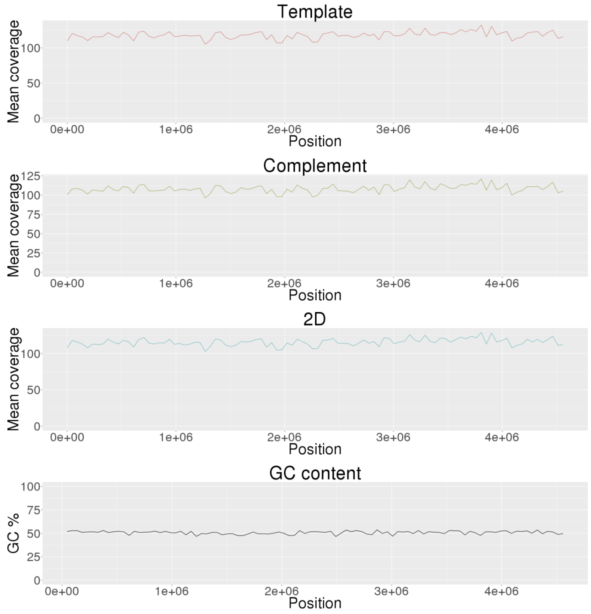

Escherichia coli 5-mer analysis

Under-represented 5-mers

	Template				Complement				2D			
Rank	kmer	Ref %	Read %	Diff %	kmer	Ref %	Read %	Diff %	kmer	Ref %	Read %	Diff %
1	TTTTT	0.251	0.041	-0.210	TTTTT	0.251	0.066	-0.185	TTTTT	0.251	0.139	-0.112
2	AAAAA	0.247	0.102	-0.145	AAAAA	0.247	0.105	-0.142	AAAAA	0.247	0.154	-0.093
3	GTTTT	0.186	0.096	-0.090	CCTGG	0.130	0.061	-0.070	CCTGG	0.130	0.038	-0.093
4	GCCAG	0.280	0.196	-0.084	CCAGG	0.129	0.065	-0.064	CCAGG	0.129	0.044	-0.085
5	CCTGG	0.130	0.057	-0.074	CCCAG	0.111	0.054	-0.057	GCCAG	0.280	0.233	-0.047
6	CCAGG	0.129	0.063	-0.067	TCCAG	0.171	0.120	-0.052	GCCTG	0.185	0.149	-0.036
7	TTTTA	0.147	0.087	-0.060	AAAGA	0.132	0.083	-0.048	CAGGC	0.183	0.149	-0.034
8	TTTTG	0.172	0.112	-0.060	GAAAG	0.130	0.083	-0.048	CCCAG	0.111	0.084	-0.027
9	TTTTC	0.198	0.140	-0.058	AGAAA	0.140	0.096	-0.044	CCCTG	0.093	0.067	-0.026
10	AAAGA	0.132	0.074	-0.057	ATCCC	0.086	0.042	-0.044	GTTTT	0.186	0.161	-0.025

Over-represented 5-mers

	Template				Complement				2D			
Rank	kmer	Ref %	Read %	Diff %	kmer	Ref %	Read %	Diff %	kmer	Ref %	Read %	Diff %
1	CGGCG	0.221	0.348	0.127	CGGCG	0.221	0.284	0.063	CGGTG	0.160	0.202	0.042
2	GCGGC	0.210	0.309	0.099	GCGGC	0.210	0.265	0.056	CACGG	0.089	0.130	0.041
3	CGGTG	0.160	0.252	0.092	GGCGG	0.190	0.234	0.045	CGGCG	0.221	0.260	0.039
4	GGCGG	0.190	0.279	0.089	TGCCG	0.220	0.265	0.045	CGCCG	0.219	0.249	0.030
5	CGCCG	0.219	0.294	0.075	AACAA	0.132	0.176	0.044	GCTGG	0.279	0.309	0.029
6	GGCCG	0.069	0.141	0.071	CGGTG	0.160	0.202	0.042	GGCGG	0.190	0.216	0.026
7	CACGG	0.089	0.156	0.067	CGCCG	0.219	0.261	0.042	CACCG	0.166	0.192	0.026
8	CGCGG	0.137	0.203	0.065	AAACA	0.138	0.180	0.042	CCGGC	0.141	0.167	0.026
9	CGCGC	0.201	0.264	0.063	GCAAA	0.196	0.236	0.040	ACGGC	0.136	0.160	0.025
10	GTACG	0.071	0.135	0.063	AATAA	0.148	0.187	0.039	TGCTG	0.250	0.274	0.025

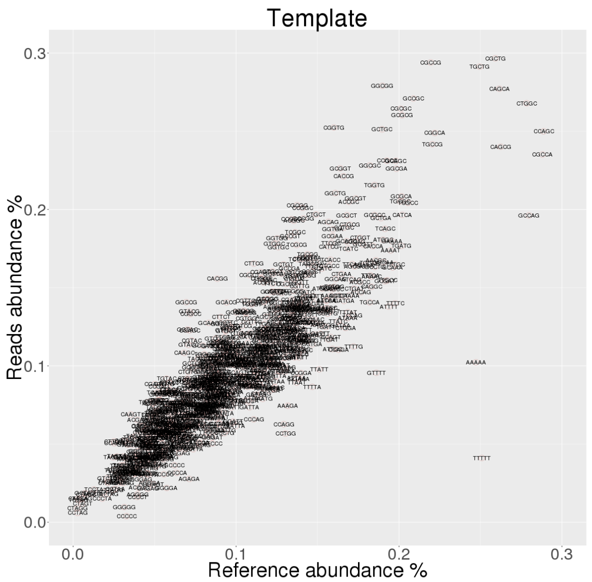

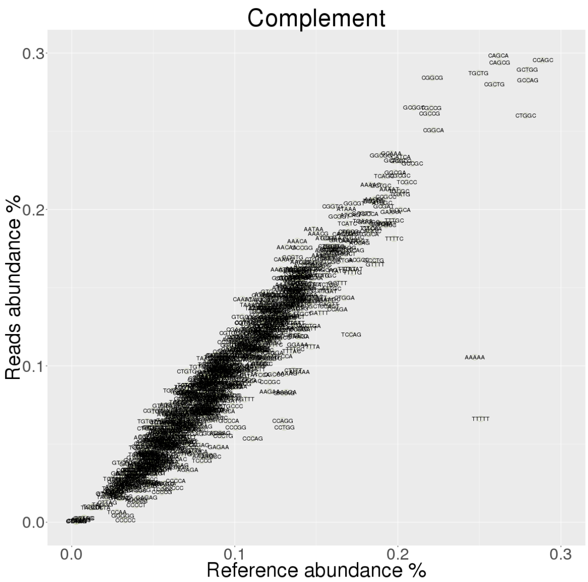

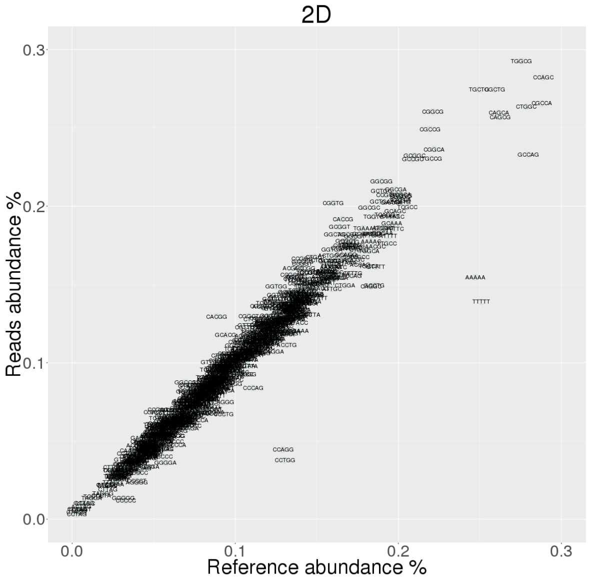

Escherichia coli GC content

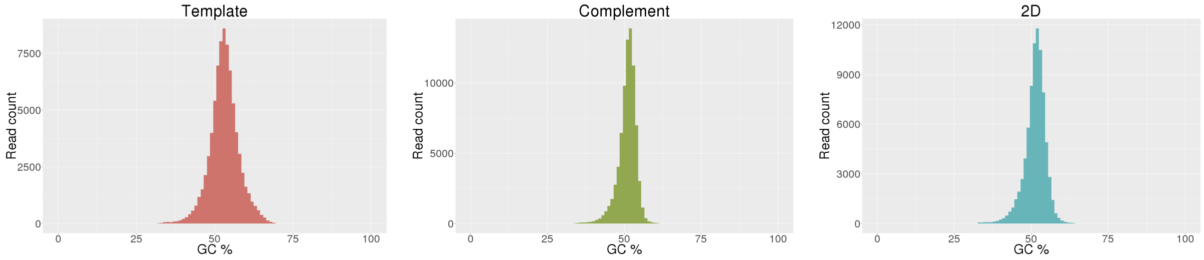

All reference 21mer analysis

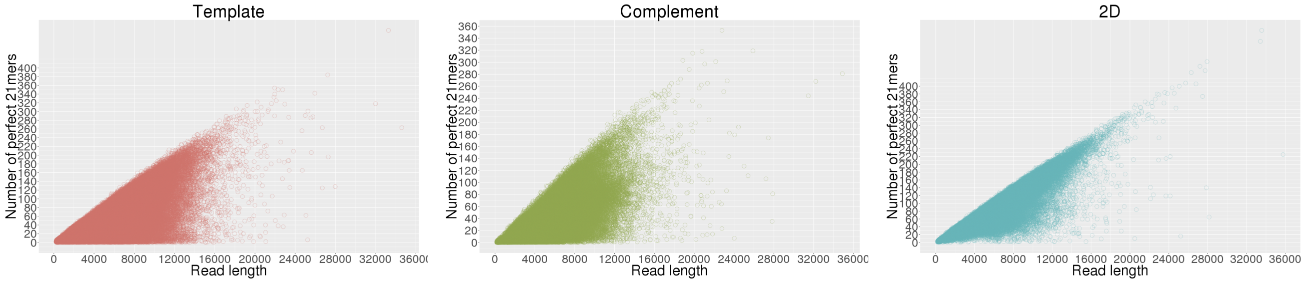

All reference substitutions

		Template substituted %				Complement substituted %				2D substituted %			
		a	c	g	t	a	c	g	t	a	c	g	t
Reference	A	0.00	7.71	17.69	4.51	0.00	4.87	15.16	5.18	0.00	6.76	15.77	5.23
	C	3.54	0.00	3.78	12.36	5.35	0.00	3.80	15.55	5.13	0.00	4.11	13.11
	G	12.18	4.07	0.00	3.51	15.02	3.91	0.00	5.26	12.66	4.25	0.00	4.92
	T	4.51	18.20	7.94	0.00	5.18	15.65	5.09	0.00	5.27	16.23	6.58	0.00

Kmer motifs before errors

3-mer error motif analysis

	Template			Complement			2D			
Rank	Insertion	Deletion	Substitution	Insertion	Deletion	Substitution	Insertion	Deletion	Substitution	
1	TTC (3.36%)	CGC (3.32%)	CGC (4.01%)	CGC (3.31%)	CGC (3.11%)	GCC (3.50%)	CGC (3.08%)	TTT (2.99%)	GCC (4.67%)	Most common
2	CGC (2.95%)	TTC (3.03%)	GCC (3.75%)	ATC (2.95%)	TTT (2.84%)	CGC (3.25%)	TTC (3.00%)	CGC (2.95%)	ACC (3.70%)	
3	ATC (2.85%)	GCC (2.94%)	ACC (3.27%)	TTC (2.68%)	ATC (2.79%)	ATC (3.15%)	GCC (2.82%)	GCC (2.94%)	CGC (3.61%)	
4	TTT (2.70%)	ATC (2.83%)	ATC (3.23%)	TTT (2.67%)	GCC (2.61%)	AAC (2.83%)	TTT (2.71%)	ATC (2.77%)	TGC (3.19%)	
5	GCC (2.67%)	TTT (2.78%)	TGC (3.19%)	GGC (2.57%)	GGC (2.59%)	TGC (2.83%)	TGC (2.68%)	TTC (2.63%)	ATC (3.16%)	
6	ACC (2.37%)	GGC (2.73%)	GGC (3.02%)	GCG (2.56%)	GCG (2.54%)	ACC (2.79%)	ATC (2.63%)	ACC (2.62%)	TTT (3.06%)	
7	TGC (2.36%)	GCG (2.70%)	TTC (2.98%)	TGC (2.50%)	TTC (2.47%)	TTC (2.64%)	GGC (2.47%)	GGC (2.61%)	TCC (3.00%)	
8	GCG (2.25%)	ACC (2.55%)	AAC (2.89%)	GCC (2.49%)	TGC (2.30%)	TTT (2.62%)	ACC (2.37%)	GCG (2.26%)	TTC (2.92%)	
9	GGC (2.21%)	TGC (2.23%)	AGC (2.65%)	AAC (2.33%)	GAT (2.29%)	GGC (2.53%)	AGC (2.22%)	TGC (2.22%)	GGC (2.72%)	
10	AAC (2.09%)	AGC (2.15%)	TCC (2.48%)	GAT (2.24%)	AAA (2.24%)	GCG (2.41%)	ATT (2.09%)	ATT (2.21%)	AAC (2.59%)	
	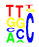	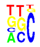	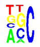	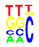	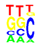	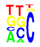	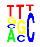	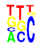	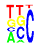	
-10	TTA (1.09%)	AGA (0.97%)	GTA (0.85%)	CCC (0.96%)	GGA (0.93%)	TGT (0.82%)	TAA (1.02%)	AGA (0.97%)	GTA (0.82%)	Least common
-9	TAA (0.92%)	GTA (0.87%)	ACT (0.84%)	AGA (0.95%)	CCC (0.91%)	TCT (0.82%)	GTA (1.00%)	GAG (0.92%)	CGA (0.81%)	
-8	AGT (0.91%)	AGT (0.84%)	CCT (0.83%)	CCT (0.94%)	GTA (0.81%)	GAG (0.79%)	GAG (0.96%)	AGT (0.90%)	TAT (0.80%)	
-7	GAG (0.87%)	GGA (0.84%)	GAG (0.81%)	GTA (0.83%)	GAG (0.80%)	ACT (0.67%)	AGT (0.95%)	GGA (0.87%)	GAG (0.78%)	
-6	AGA (0.81%)	TAA (0.83%)	GGG (0.64%)	AGG (0.79%)	AGG (0.78%)	GGG (0.66%)	AGA (0.79%)	GTA (0.86%)	GGG (0.75%)	
-5	GGA (0.79%)	AGG (0.77%)	AGA (0.61%)	GGA (0.79%)	AGT (0.73%)	GGA (0.65%)	AGG (0.75%)	CCT (0.74%)	AGA (0.62%)	
-4	AGG (0.75%)	CCT (0.67%)	AGT (0.59%)	AGT (0.76%)	CCT (0.64%)	CCT (0.57%)	GGA (0.73%)	AGG (0.74%)	AGT (0.53%)	
-3	GGG (0.51%)	GGG (0.62%)	GGA (0.56%)	GGG (0.53%)	GGG (0.57%)	AGT (0.48%)	GGG (0.59%)	GGG (0.62%)	GGA (0.51%)	
-2	CTA (0.47%)	TAG (0.41%)	TAG (0.42%)	TAG (0.36%)	TAG (0.33%)	TAG (0.40%)	TAG (0.44%)	CTA (0.41%)	TAG (0.39%)	
-1	TAG (0.45%)	CTA (0.37%)	CTA (0.29%)	CTA (0.28%)	CTA (0.27%)	CTA (0.27%)	CTA (0.42%)	TAG (0.40%)	CTA (0.32%)	
	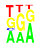	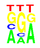	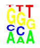	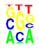	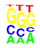	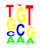	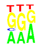	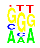	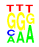	

Kmer space for 3-mers: 64 Random chance for any given 3-mer: 1.56%

4-mer error motif analysis

Rank	Template			Complement			2D			
	Insertion	Deletion	Substitution	Insertion	Deletion	Substitution	Insertion	Deletion	Substitution	
1	TTTC (1.18%)	CGCC (1.24%)	CGCC (1.60%)	CAGC (1.12%)	CAGC (1.14%)	CGCC (1.47%)	CGCC (1.13%)	CGCC (1.20%)	CGCC (1.90%)	Most common
2	CGCC (1.14%)	GCGC (1.15%)	GCGC (1.26%)	GCGC (1.08%)	CGCC (1.12%)	CAGC (1.15%)	CAGC (1.00%)	GCGC (1.02%)	TGCC (1.28%)	
3	GCGC (0.99%)	CAGC (1.06%)	CAGC (1.25%)	CGCC (1.07%)	GCGC (1.06%)	TGCC (1.05%)	TTTC (1.00%)	CAGC (1.01%)	CACC (1.13%)	
4	CAGC (0.92%)	CGGC (1.03%)	CGGC (1.10%)	CGGC (0.95%)	CGGC (0.96%)	GCGC (1.01%)	GCGC (0.99%)	CGGC (0.93%)	GCGC (1.13%)	
5	CTTC (0.87%)	TTTC (1.02%)	CACC (1.04%)	TGGC (0.89%)	TGGC (0.90%)	CATC (0.95%)	TGGC (0.86%)	CACC (0.87%)	AACC (1.10%)	
6	CATC (0.84%)	GGCG (0.91%)	CATC (1.03%)	CATC (0.87%)	CATC (0.86%)	AAAC (0.93%)	CGGC (0.84%)	TTTC (0.84%)	CAGC (1.09%)	
7	GCTG (0.82%)	CATC (0.90%)	TGGC (0.98%)	TTTC (0.87%)	TTTC (0.85%)	TGGC (0.90%)	TGCC (0.80%)	ATTT (0.84%)	ATCC (1.08%)	
8	CGGC (0.80%)	CACC (0.86%)	ACGC (0.96%)	ACGC (0.84%)	GGCG (0.82%)	CGGC (0.89%)	CTGC (0.79%)	CATC (0.82%)	TTCC (1.05%)	
9	CACC (0.79%)	TGGC (0.83%)	CCGC (0.96%)	GGCG (0.82%)	TTTT (0.80%)	AACC (0.88%)	CTTC (0.79%)	TTTT (0.82%)	TTTT (1.01%)	
10	GCCA (0.76%)	GTTT (0.80%)	TGCC (0.95%)	GCTG (0.78%)	ATTT (0.79%)	CACC (0.85%)	TTGC (0.78%)	TGGC (0.81%)	AGCC (0.98%)	
	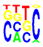	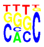	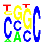	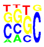	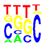	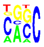	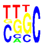	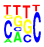	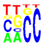	
-10	TTAG (0.11%)	CTTA (0.11%)	TAGT (0.09%)	GGGA (0.10%)	TAGA (0.09%)	CCCT (0.08%)	AGGG (0.11%)	TTAG (0.11%)	CTAA (0.09%)	Least common
-9	TCTA (0.11%)	TTAG (0.10%)	GGGA (0.09%)	TAGT (0.09%)	CTAA (0.09%)	GAGT (0.07%)	ACTA (0.11%)	CCCT (0.11%)	ACTA (0.09%)	
-8	GGGA (0.11%)	ACTA (0.10%)	GGGG (0.09%)	CTAA (0.09%)	CCCT (0.09%)	ACTA (0.07%)	GGGA (0.11%)	TAGA (0.11%)	GGGA (0.08%)	
-7	AGGG (0.10%)	TCTA (0.09%)	ACTA (0.08%)	TAGA (0.08%)	TAGT (0.08%)	TAGG (0.07%)	GGGG (0.10%)	ACTA (0.10%)	GAGT (0.08%)	
-6	CTAA (0.09%)	GGGG (0.09%)	GAGT (0.08%)	ACTA (0.07%)	GGGG (0.07%)	GGGG (0.07%)	CTAA (0.10%)	CTAA (0.10%)	TAGG (0.07%)	
-5	TAGA (0.08%)	CCCT (0.09%)	TAGG (0.08%)	TCTA (0.07%)	ACTA (0.07%)	TAGA (0.07%)	TCTA (0.09%)	GGGG (0.10%)	TAGT (0.06%)	
-4	GGGG (0.07%)	CTAA (0.08%)	TCTA (0.06%)	GGGG (0.07%)	TCTA (0.06%)	TAGT (0.05%)	TAGA (0.08%)	TCTA (0.10%)	TCTA (0.06%)	
-3	CCTA (0.07%)	TAGG (0.06%)	TAGA (0.06%)	TAGG (0.05%)	TAGG (0.05%)	TCTA (0.05%)	TAGG (0.06%)	TAGG (0.06%)	TAGA (0.05%)	
-2	TAGG (0.06%)	CCTA (0.05%)	CCTA (0.04%)	CCTA (0.03%)	CCTA (0.03%)	CCTA (0.03%)	CCTA (0.06%)	CCTA (0.06%)	CCTA (0.04%)	
-1	CTAG (0.01%)	CTAG (0.01%)	CTAG (0.01%)	CTAG (0.00%)	CTAG (0.00%)	CTAG (0.00%)	CTAG (0.01%)	CTAG (0.01%)	CTAG (0.01%)	
	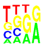	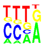	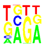	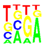	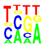	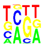	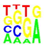	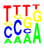	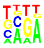	

Kmer space for 4-mers: 256 Random chance for any given 4-mer: 0.39%

5-mer error motif analysis

Rank	Template			Complement			2D			
	Insertion	Deletion	Substitution	Insertion	Deletion	Substitution	Insertion	Deletion	Substitution	
1	ATTTC (0.37%)	GCGGC (0.38%)	CCAGC (0.48%)	CCAGC (0.42%)	CCAGC (0.43%)	CCAGC (0.44%)	CCAGC (0.36%)	CCAGC (0.36%)	CCGCC (0.54%)	Most common
2	CGCCA (0.36%)	CCAGC (0.38%)	CCGCC (0.44%)	CTGGC (0.37%)	CTGGC (0.38%)	TCGCC (0.43%)	CGCCA (0.34%)	GCGGC (0.34%)	TCGCC (0.52%)	
3	GTTTC (0.34%)	GGCGC (0.35%)	TCGCC (0.43%)	GCGGC (0.34%)	GCGGC (0.36%)	CTGGC (0.38%)	CTGGC (0.33%)	TCGCC (0.34%)	CAGCC (0.49%)	
4	CCAGC (0.34%)	TCGCC (0.34%)	CTGGC (0.41%)	TGGCG (0.32%)	CGCCA (0.34%)	CCGCC (0.37%)	TCGCC (0.31%)	CGCCA (0.34%)	GCGCC (0.45%)	
5	TCGCC (0.31%)	CCGCC (0.33%)	GCGGC (0.40%)	CGCCA (0.31%)	TGGCG (0.33%)	GCGCC (0.36%)	GCGGC (0.30%)	CTGGC (0.32%)	ACGCC (0.43%)	
6	CGCGC (0.30%)	TGGCG (0.32%)	GCGCC (0.40%)	CAGCG (0.31%)	TCGCC (0.32%)	CGCCA (0.34%)	CCGCC (0.30%)	GGCGC (0.31%)	TTGCC (0.41%)	
7	CCGCC (0.30%)	CTGGC (0.32%)	ACGCC (0.37%)	CGCGC (0.31%)	CGCGC (0.30%)	ACGCC (0.34%)	ATTTC (0.28%)	CCGCC (0.31%)	CCAGC (0.41%)	
8	GCGGC (0.29%)	CGCGC (0.30%)	CAGCC (0.36%)	AACGC (0.30%)	GCTGG (0.29%)	GCGGC (0.33%)	CAGCA (0.28%)	TCACC (0.28%)	CTGCC (0.36%)	
9	CAGCA (0.28%)	TTTTC (0.29%)	GCCGC (0.36%)	TCGCC (0.30%)	GGCGC (0.29%)	TTGCC (0.33%)	ACGCC (0.27%)	ACGCC (0.28%)	CTGGC (0.36%)	
10	CTGGC (0.28%)	ACGCC (0.29%)	CGCGC (0.35%)	TCAGC (0.28%)	CAGCG (0.29%)	CAGCC (0.32%)	TGCGC (0.27%)	GCGCC (0.28%)	TCACC (0.35%)	
	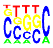	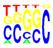	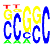	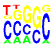	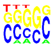	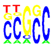	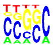	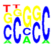	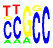	
-10	CCTGG (0.01%)	CCAGG (0.01%)	TCCTA (0.01%)	CCCCC (0.00%)	TCCTA (0.00%)	CCCCC (0.00%)	TCCTA (0.01%)	TCCTA (0.01%)	CTAGC (0.01%)	Least common
-9	CTAGC (0.01%)	GGGGG (0.01%)	TAGGA (0.00%)	GGGGG (0.00%)	GGGGG (0.00%)	CTAGC (0.00%)	TAGGA (0.01%)	TAGGA (0.01%)	TCCTA (0.01%)	
-8	ACTAG (0.00%)	CTAGT (0.00%)	GGGGG (0.00%)	CTAGC (0.00%)	CTAGC (0.00%)	GGGGG (0.00%)	CTAGC (0.01%)	CTAGC (0.00%)	TAGGA (0.00%)	
-7	GCTAG (0.00%)	CTAGC (0.00%)	ACTAG (0.00%)	GCTAG (0.00%)	GCTAG (0.00%)	GCTAG (0.00%)	GCTAG (0.00%)	CTAGT (0.00%)	GCTAG (0.00%)	
-6	GGGGG (0.00%)	ACTAG (0.00%)	CTAGT (0.00%)	CTAGT (0.00%)	CTAGT (0.00%)	ACTAG (0.00%)	CTAGT (0.00%)	ACTAG (0.00%)	ACTAG (0.00%)	
-5	CTAGT (0.00%)	GCTAG (0.00%)	GCTAG (0.00%)	ACTAG (0.00%)	ACTAG (0.00%)	TCTAG (0.00%)	ACTAG (0.00%)	GCTAG (0.00%)	CTAGT (0.00%)	
-4	TCTAG (0.00%)	TCTAG (0.00%)	TCTAG (0.00%)	TCTAG (0.00%)	CTAGA (0.00%)	CTAGT (0.00%)	TCTAG (0.00%)	TCTAG (0.00%)	TCTAG (0.00%)	
-3	CCTAG (0.00%)	CTAGA (0.00%)	CTAGG (0.00%)	CTAGA (0.00%)	TCTAG (0.00%)	CTAGG (0.00%)	CTAGA (0.00%)	CTAGA (0.00%)	CTAGG (0.00%)	
-2	CTAGA (0.00%)	CTAGG (0.00%)	CCTAG (0.00%)	CTAGG (0.00%)	CTAGG (0.00%)	CCTAG (0.00%)	CCTAG (0.00%)	CTAGG (0.00%)	CCTAG (0.00%)	
-1	CTAGG (0.00%)	CCTAG (0.00%)	CTAGA (0.00%)	CCTAG (0.00%)	CCTAG (0.00%)	CTAGA (0.00%)	CTAGG (0.00%)	CCTAG (0.00%)	CTAGA (0.00%)	
	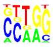	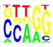	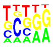	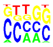	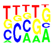	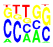	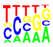	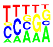	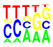	

Kmer space for 5-mers: 1024 Random chance for any given 5-mer: 0.10%
