# Supplementary material for: MinION Analysis and Reference Consortium: Phase 2 data release and analysis of R9.0 chemistry
Source: F1000Res. 2017 May 31;6:760. [Version 1] doi: 10.12688/f1000research.11354.1 (PMC5538040; doi:10.12688/f1000research.11354.1)
Supplement: Peer review history

# Peer review: Referee response for version 1

**DOI:** 10.5256/f1000research.12257.r23369

**Published:** 2017-07-28

**Authors:** Titus Brown, Lisa Cohen

**Affiliations:** 1 Department of Population Health and Reproduction, University of California, Davis, Davis, CA, USA; 2 Department of Population Health and Reproduction, University of California, Davis, CA, USA

This is a followup data release and analysis by the MinION Analysis and Reference Consortium (MARC) to the Phase 1 release by Ip et al. in 2015 ( https://f1000research.com/articles/4-1075/v1), who compared the consistency, rate, volume and quality of E. coli K-12 data produced by 5 labs from 2 R7.3 flow cell runs each. In this Phase 2 data release and analysis, the MARC characterized the throughput, read quality, and accuracy of one run each of 1D and 2D library preps of R9.0 chemistry. 

 Aside from a fundamental issue with this study that it would have been nice to have data from more than 2 labs, and more than 1 flowcell run of each 1D and 2D (only 4 total flowcells were used in this study), there were several really nice features:

 Great idea to compare!

 "Sequencing was performed using both the 2-dimensional (“2D”) “ligation” kit and the newer 1D “rapid” kit."

 Excellent, thank you for providing these data!

 "It is a resource to aid further developments in nanopore informatics as well as the development of biological applications using the MinION."

 Cool!

 "overall, the R9.0 chemistry showed an increase in data yield and read length when

 compared with a typical Phase 1 R7.3 experiment"

 A few criticisms and questions -

1. Would have liked more robust comparison and discussion on differences between 2D and 1D sequencing since the consensus in the community seems to be now that 1D sequencing libraries are fine (nobody uses 2D anymore). "The higher total error of 15.3% for “pass” template base-calls, compared with 7.5% for “pass” 2D base-calls (Table 4), is an acceptable trade off."
2. ENA accessions could be more clear, such as Table S10 from MARC Phase 1 paper (Ip et al. 2015).
  * These data are clearly generated by experts (some of whom are long-term experts and paid consultants supported by ONT), with available pore numbers and sequencing yields representing best case scenarios. While perhaps beyond the scope of this benchmark, it would be nice to see similar data comparisons by novice labs trying to figure this technology out.
3. Why are 1D and 2D library preparation modifications made in this study not part of standard ONT protocols? What was reasoning behind making these changes? One of the hardest parts of figuring out ONT is troubleshooting the little modifications like the ones mentioned in this study. Modifications indicated in the manuscript: genomic DNA was sheared to ~10 kb and 0.4x AMPureXP cleanup treatment. And 1D: 0.4x AMPureXP cleanup prior to prep, unsheared DNA input of 400ng, 0.4ul blunt/TA ligase; 10 min incubation used in final step. 
4. This might be obvious, but I'm not sure: why was the lambda control DNA not included in the 1D runs?
5. Why does the % of active pores decrease from g1 to g2? It is difficult to compare the percentage of active pores between flowcells since, as the manuscript states, the computer from Lab7 crashed in the middle of the experiment and these numbers were not available. And there were only 4 flowcells used in this study. What are some of the reasons why the number of active pores fluctuates between flow cells? 
6. Were these spot on flowcells? This feature was added recently sometime after R9.0 was released, and I am curious what effect this had on sequencing yield.
7. Perhaps include data analyses software versions?
  "we computed alignments and error-rate measurements using BWA-MEM and LAST, followed by re-alignment using marginAlign. Real-time evaluation of the runs was performed by minoTour (more information available from: http://minotour.github.io/minoTour), run locally at the two experimental laboratories. The “pass” and “fail” reads from each experiment were evaluated with NanoOK (version 0.95) using bwa alignments. Additional metrics and analyses were performed with bespoke Python and R scripts, (available at https://github.com/camilla-ip/marcp2).

We have read this submission. We believe that we have an appropriate level of expertise to confirm that it is of an acceptable scientific standard, however we have significant reservations, as outlined above.

# Peer review: Referee response for version 1

**DOI:** 10.5256/f1000research.12257.r24143

**Published:** 2017-07-27

**Authors:** Martin C. Frith

**Affiliations:** 1 Artificial Intelligence Research Center, National Institute of Advanced Industrial Science and Technology (AIST), Tokyo, Japan; 2 Department of Computational Biology and Medical Sciences, Graduate School of Frontier Sciences, The University of Tokyo, Kashiwa, Japan; 3 Computational Bio Big-Data Open Innovation Laboratory (CBBD-OIL), National Institute of Advanced Industrial Science and Technology (AIST), Tokyo, Japan

This study tests version R9.0 of the MinION nanopore sequencer, and describes its error rate, read lengths, throughput, and other characteristics. The article's timing is unfortunate, because I believe R9.0 had already been superceded at the time of publication, but this does not affect the study's soundness.  As far as I can tell, this study is basically sound, but there are some careless mistakes:

- This text does not match Table 2: maybe pass -> total? "64–111 K 2D pass reads (compared with 21 K for the typical R7.3 experiment)".
- Is this really an "increase"? "increase in N50 read length to 7.3–9.1 Kb for all 2D reads in the R9.0 experiments (compared with 7.4 Kb". The abstract says: "The 2-dimensional (“2D”) N50 read length was unchanged".
- This text does not match Table 3, maybe pass/fail -> total/pass? "mean template length for “pass” reads increasing to 7.2–8.6 Kb (from 5.0 for R7.3) and increasing to 7.8–9.4 for “fail” reads (from 6.2":
- This text also does not match Table 3: "Read length N50 increased to 13.1–15.7 Kb for “pass” reads (compared with 6.9 Kb for the typical R7.3) and 13.6–16.2 Kb"
- This is true only for 1D reads: "For template reads from both 1D and 2D experiments, 99.9% of “pass” reads were alignable from both 1D and 2D".
- 88% is not similar to 78%: "The median identity of reads from 1D and 2D experiments (Table 4) was similar...  The median identity for 1D template reads was ~88% and ~76%, for “pass” and “fail”, respectively (compared with 78% and 75%".
- This is not comparing like with like (R9.0 template versus R7.3 2D): "For the 2D experiments, the read identity was ~89% and ~85%, for “pass” and “fail”, respectively (compared with ~92% and ~82%, respectively, for the typical R7.3 experiment)."

A few things should be clarified:

- Why do "Identity %" and "Total error %" not sum to 100?
- Fig 2:
  - what is the difference between Q-score and BQ?
  - what is "(Temp)"?
  - what is the difference between "GC" and "GC (Temp)"?
  - what is "throughput": count of what per what?
- What is "1D consensus follow through"?
- What does "bridging experiment" mean?
- What is a "run script"?

Other minor comments:

- The title should be shortened to something like "Analysis of MinION R9.0 chemistry". The rest is not scientifically meaningful, and might be perceived as "appeal to authority".
- The abstract "methods" section is incorrectly brief.
- The abstract "conclusions" section should probably not say "new" R9.0 chemistry.
- Is this really "higher"? "higher at... 6.0–6.5 Kb (compared with 6.0 Kb".
- Page 4: "were statistics computed" -> "statistics were computed".
- The LAST usage is likely suboptimal (though I guess it matters little here).  The currently-recommended usage has been here since 2016-11-22: https://github.com/mcfrith/last-rna/blob/master/last-long-reads.md

Data availability:

 I found it excessively hard to obtain the data. The PRJEB18053 link leads to three "component projects", each of which has numerous files. Which file is which dataset?  This should be better organized, or at least described. The best I could do was to mouse-over the links: a name like "Nott_R9_run2_1D_pass_f74a133aa1ac903384a928a51051582db2cc412b_0.fastq" gives me a clue, but a name like "ERR2025969.fastq" is hopeless.

 For example, what is the difference between these files?

 Nott_R9_run2_1D.pass.1D.fastq

 Nott_R9_run2_1D_pass_f74a133aa1ac903384a928a51051582db2cc412b_0.fastq

 Suggestions for future studies of this type:

- Characterize the substitution errors further, e.g. is A->G more frequent?
- Are the insertions and deletions long-and-rare, or short-and-numerous?
- Are the base quality scores accurate/useful?  (And what do they even mean when indels are the main error?)
- Characterize context-dependence of errors, e.g. homopolymers, CCXGG context (http://www.biorxiv.org/content/early/2017/06/29/157040).
- Can rearrangement errors be characterized? Long reads are promising for finding rearrangements (e.g. inversions, translocations), but do artifactual rearrangements occur?

I have read this submission. I believe that I have an appropriate level of expertise to confirm that it is of an acceptable scientific standard.

# Peer review: Referee response for version 1

**DOI:** 10.5256/f1000research.12257.r23414

**Published:** 2017-06-28

**Authors:** Wigard P. Kloosterman, Jose Espejo Valle Inclan, Mircea Cretu Stancu

**Affiliations:** 1 Department of Genetics, University Medical Center Utrecht, Utrecht, Netherlands

In this work the Minion Analysis and Reference Consortium describes the analysis of Oxford Nanopore sequencing data generated from *E.coli* using the R9.0 sequencing chemistry. The R9.0 data characteristics were benchmarked against previous R7.3 data. The work is of much interest to (new) users of the Oxford Nanopore sequencing technology, as it provides a realistic overview of how the technology has developed over the course of 2016 and what can be expected in terms of data throughput and quality.

 I have the following remarks:

 Major point

- Oxford Nanopore sequencing technology has developed rapidly over the last year. Yet, the data presented in the paper are derived from older R9.0 and R7.3 chemistries. The value of the data analysis and comparisons would much increase if one or two runs of the more recent R9.4 data will be added as an extra column to each of the plots/tables. R9.4 chemistry is mentioned several times in the manuscript, but unfortunately no data are shown.

Other points

- Materials & methods:
  - The authors mention that DNA extraction procedures are described in MARC Phase 1, but no reference is given. Instead, the authors do refer to Supplementary File 1, but this file only contains a list of affiliations. I would suggest that the authors provide the appropriate reference here and/or refer to a Supplementary file that describes the DNA extraction procedures (or add this in the methods).
  - 1D/2D library preparation: The authors list some modifications with respect to ONT protocols. For the more ignorant reader, the authors could spell out why these modifications were added.
  - Sentence page 4: “Both alignments were then improved with marginAlign.” What does ‘improved’ mean in this case? If specific marginAlign settings were used, then these should be listed as well.
- Results:
  - Base yield and read lengths:
    - There appear some inconsistencies regarding claims about read lengths for R7.3 vs R9.0. The abstract states that read length N50 was not different, while page 4 states that “R9.0 chemistry showed an increase in [...] read length” compare to R7.3. Are differences in yield and read lengths statistically significant between R7.3 and R9.0?
    - Related to this: the authors mention that median read length is longer for R9.0 compared to R7.3 (6.6kb - 7.7kb and 6.4kb), yet they mention that the maximum read length is comparable (50.9kb and 59.7 kb). Why are median values regarded as different, while maximum values are regarded as comparable?
  - Base quality:
    - “The proportion of “pass” reads with a Q-value threshold of 9 was 66% to 72%, about the same as that observed for the typical R7.3 experiment, with a base quality threshold of 9.0.” Is Q-value equivalent to base quality value here? Or do the authors mean Q-value instead of base quality? A similar statement is made later in this paragraph.
  - Alignment identity and accuracy:
    - From the Table, it appears that R9.0 “fail” reads are worse than R7 failed reads. Could the authors comment why this is the case?
    - Page 7, second column: There appears to be a mistake in the given read identities for 2D R9.0 experiments (89% and 85% given, while these numbers appear for R9.0 template reads; should be ~94% and ~70%).
    - The authors make a point about read quality and mention that the longest subsequence that perfectly aligns increases around 2-4 times for 1D runs, going from R7.3 to R9. Does this mean that the errors are less randomly distributed in R9 data, given that the median percent identity does not change substantially? The authors could improve this analysis, by evaluating the randomness of the error distribution within reads, or across the genome and how this relates to genome sequence context.
  - Performance over time (Figure 2):
    - Final sentence of results: “The current report is for the initial R9.0 chemistry, and the GC-bias seems to be less pronounced with the improved version of the R9 pore (R9.4 data not shown).” It would be better if the authors draw a clear conclusion whether this bias is present or not, and include data to support this.
    - Page 9: the authors mention that GC content differs for different run and read types. It would be good if the authors quantify these differences and provide the numbers in the text.
    - Figure 2: What does count mean here? Read counts or event counts?
    - Figure 2 legend: Read length is referred to as ‘kb’, but probably the authors mean ‘b’ (looking at the y-axis of the length plot).
    - Page 8: “the quantity and quality of data produced during an experiment varied as material passed from one side of the membrane to the other.” What does this mean exactly? This could be replaced by a more precise statement.

We have read this submission. We believe that we have an appropriate level of expertise to confirm that it is of an acceptable scientific standard, however we have significant reservations, as outlined above.
